# Discovery of Candidate Disease Genes in ENU–Induced Mouse Mutants by Large-Scale Sequencing, Including a Splice-Site Mutation in Nucleoredoxin

**DOI:** 10.1371/journal.pgen.1000759

**Published:** 2009-12-11

**Authors:** Melissa K. Boles, Bonney M. Wilkinson, Laurens G. Wilming, Bin Liu, Frank J. Probst, Jennifer Harrow, Darren Grafham, Kathryn E. Hentges, Lanette P. Woodward, Andrea Maxwell, Karen Mitchell, Michael D. Risley, Randy Johnson, Karen Hirschi, James R. Lupski, Yosuke Funato, Hiroaki Miki, Pablo Marin-Garcia, Lucy Matthews, Alison J. Coffey, Anne Parker, Tim J. Hubbard, Jane Rogers, Allan Bradley, David J. Adams, Monica J. Justice

**Affiliations:** 1 Department of Molecular and Human Genetics, Baylor College of Medicine, Houston, Texas, United States of America; 2 The Wellcome Trust Sanger Institute, Hinxton, Cambridgeshire, United Kingdom; 3 Faculty of Life Sciences, University of Manchester, Manchester, United Kingdom; 4 The University of Texas MD Anderson Cancer Center, Houston, Texas, United States of America; 5 Department of Pediatrics, Baylor College of Medicine, Houston, Texas, United States of America; 6 Texas Children's Hospital, Houston, Texas, United States of America; 7 Laboratory of Intracellular Signaling, Institute for Protein Research, Osaka University, Osaka, Japan; Harvard Medical School, United States of America

## Abstract

An accurate and precisely annotated genome assembly is a fundamental requirement for functional genomic analysis. Here, the complete DNA sequence and gene annotation of mouse Chromosome 11 was used to test the efficacy of large-scale sequencing for mutation identification. We re-sequenced the 14,000 annotated exons and boundaries from over 900 genes in 41 recessive mutant mouse lines that were isolated in an *N*-ethyl-*N*-nitrosourea (ENU) mutation screen targeted to mouse Chromosome 11. Fifty-nine sequence variants were identified in 55 genes from 31 mutant lines. 39% of the lesions lie in coding sequences and create primarily missense mutations. The other 61% lie in noncoding regions, many of them in highly conserved sequences. A lesion in the perinatal lethal line *l11Jus13* alters a consensus splice site of nucleoredoxin (*Nxn*), inserting 10 amino acids into the resulting protein. We conclude that point mutations can be accurately and sensitively recovered by large-scale sequencing, and that conserved noncoding regions should be included for disease mutation identification. Only seven of the candidate genes we report have been previously targeted by mutation in mice or rats, showing that despite ongoing efforts to functionally annotate genes in the mammalian genome, an enormous gap remains between phenotype and function. Our data show that the classical positional mapping approach of disease mutation identification can be extended to large target regions using high-throughput sequencing.

## Author Summary

Here we show that tiny DNA lesions can be found in huge amounts of DNA sequence data, similar to finding a needle in a haystack. These lesions identify many new candidates for disease genes associated with birth defects, infertility, and growth. Further, our data suggest that we know very little about what mammalian genes do. Sequencing methods are becoming cheaper and faster. Therefore, our strategy, shown here for the first time, will become commonplace.

## Introduction

Genome sequences are essential tools for comparative and mutational analyses [1],[2]. As a part of the Mouse Genome Sequencing Consortium, we sequenced mouse Chromosome 11 in the C57BL/6J mouse strain. The 2002 draft mouse genome reported 8681 gaps in mouse Chromosome 11, and was estimated to lack 4.3 Mb of sequence, despite a good shotgun assembly and a physical map [3]. Using clone-by-clone sequencing of the physical map of mouse Chromosome 11, we have assembled this chromosome into a near contiguous sequence with the highest quality of any mouse chromosome assembly (completed sequence is available in the NCBI assembly of the mouse genome at www.ensembl.org).

The sequence of Chromosome 11 from C57BL/6J now provides a basis for understanding the trait characteristics of this strain, for cross-strain comparative analysis, for identifying modifiers and quantitative trait loci (QTLs), and for the precise design of knockout alleles. Accurate DNA sequence also facilitates the discovery of disease associated genes from forward genetic screens, as well as the engineering of mutations and rearrangements in the mouse to model human diseases.

We used the completed sequence to design primers to re-sequence DNA from mutants isolated in a phenotype-driven regional recessive *N*-ethyl-*N*-nitrosourea (ENU) genetic screen targeted to mouse Chromosome 11 through the use of a balancer chromosome [4],[5]. ENU primarily induces point mutations, which have been a challenge to fine map and identify [6]. Classical positional cloning involves narrowing the molecular interval by meiotic mapping, followed by candidate gene sequence analysis, which is time consuming and expensive. Further, in many organisms, including *Caenorhabditis elegans*, *Drosophila melanogaster*, and *Escherichia coli*, chemical point mutagens often generate a multitude of linked lesions [7], which can confound the designation of a candidate gene to the mutant phenotype. In the mouse, genes are dispersed, chromatin contains large stretches of noncoding sequence, and inbred strains with fixed genetic backgrounds are readily available. We reasoned that mutations isolated using the regional screen were ideal to test the efficacy of mutation identification by sequencing after ENU point mutagenesis, because mutations were induced on a C57BL/6J chromosome, and because mutant recovery was targeted to an interval between *Trp53* and *Wnt3*, restricting the analysis to a 34 megabase (Mb) region [5]. Mouse Chromosome 11 and its conserved counterpart human Chromosome 17 are very gene dense, making classical positional cloning efforts unrealistic for large numbers of mutants. Identifying DNA lesions in mutant lines should assign candidate genes, while it defines the numbers and nature of DNA lesions detected after ENU treatment. We illustrate the value of finished sequence by recovering a rich catalogue of candidate genes in ENU-induced mutant lines. No mutations have been reported previously for the majority of the candidate genes. Here, we report for the first time that a splice-site mutation in nucleoredoxin causes craniofacial defects in the perinatal lethal line *l11Jus13*.

## Results

### The sequence of mouse Chromosome 11

**Figure 1:**
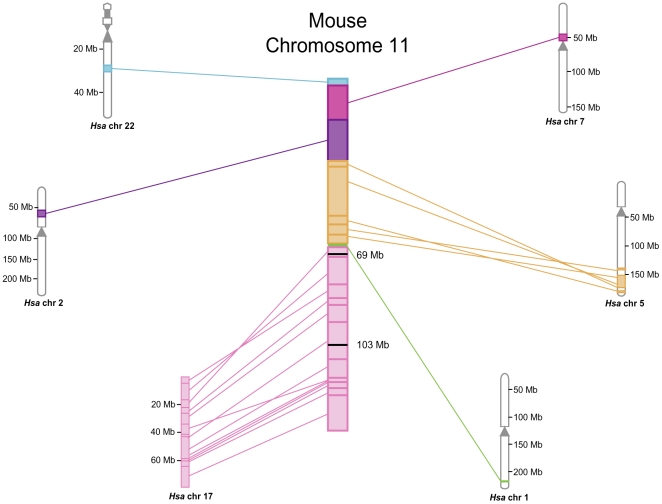
Synteny map of mouse Chromosome 11. Mouse Chromosome 11 is aligned with human chromosomes 22, 7, 2, 5, 1, and 17. Syntenic regions are represented in the same color and lines indicate breaks of synteny. The mouse Chromosome 11 ENU mutagenesis screen was targeted to the *Trp53-Wnt3* (69 Mb–103.5 Mb) interval within the region most conserved with human Chromosome 17, indicated by the black lines.

The sequence of mouse Chromosome 11 (∼4.6% of the mouse genome) was generated by sequencing 910 bacterial artificial chromosomes (BACs) from a linear physical array [8],[9]. The clone-by-clone sequencing of this chromosome generated over 4 million raw sequence reads, which were collapsed into 118.8 Mb of finished sequence. The current assembly contains three main contiguous sequences of approximately 31.4, 54.0 and 33.4 Mb separated by two gaps, which were found by fiber-FISH to be 91 and 6kb, respectively (Table S1). An additional gap represents the acrocentric heterochromatin. Thus, the physical length of the chromosome is estimated to be 121.8 Mb. The telomeric half of mouse Chromosome 11 (Mmu11) contains the equivalent of the entire euchromatic region of human Chromosome 17 (Hsa17) (Figure 1), with the exception of a small number of genes that differ between the two organisms (Table S2).

### Identifying ENU–induced lesions in the Mmu11/Hsa17 region

We previously described a screen for autosomal recessive mouse mutants using the point mutagen ENU, which was targeted to the Mmu11 region most highly conserved with Hsa17, a 34 Mb interval between *Trp53* and *Wnt3* (Figure 1) [5]. A large number of recessive mutant lines with a wide range of phenotypes, including craniofacial abnormalities, neurological defects, infertility, impaired growth, and lethality, were isolated in the screen. Each of the mutants was identified by its phenotype after breeding ENU-treated C57BL/6J male mice, and was maintained *in trans* to a balancer chromosome, which was derived from 129S5/SvEv embryonic stem (ES) cells. To identify the DNA lesions responsible for the phenotypes in these lines, we designed primers for the 14,000 annotated exons and intron/exon boundaries from all of the protein coding genes in the 34 Mb interval (Table S3), representing approximately 17,000 sequence tags. We then carried out bi-directional sequencing of PCR amplicons from DNA of either a homozygous or heterozygous individual from each of 41 mutant lines, a total of over 14 Mb of sequence covering 7.8 Mb of transcribed DNA from each line, which represents approximately one-fourth of the total DNA content of the 34 Mb balancer region. A total of 1727 sequence variants were identified, but most occurred in multiple heterozygous mutant lines and were previously published single nucleotide polymorphisms (SNPs) between the C57BL/6J and 129S5/SvEv strains [10].

Eighty-one unique potentially causative base pair changes were identified in the mutant lines. Each lesion was confirmed by re-sequencing of DNA PCR products amplified from 2–4 independent phenotype-true animals and all appropriate controls (Figure S1). However, five base changes in three mutant lines were confirmed in only a subset of DNAs. This indicates that the ENU-induced base change was present in the original DNA that was sequenced, but did not segregate with the phenotype in other animals and was therefore not causative of the phenotype (Table S4). The remaining changes included five new SNPs, which may be unique to our 129 substrain, or may not have been previously reported. That these occurred in only one line each is possibly due to the randomness of crossovers in each line. Twelve of the lesions initially identified by sequencing were not confirmed in any mutant animal whose DNA was re-sequenced.

The most common DNA base changes identified were AT-GC transitions (42.2%). AT-TA transversions represented 29.7% of the bases changes followed by GC-AT (10.9%), GC-TA (10.9%), and AT-CG (6.3%). These data are similar to the most predominant lesions reported for ENU-induced mutations after treatment of mouse spermatogonia [11]. The numbers of confirmed ENU-induced lesions per mutant mouse line fits a Poisson distribution: no lesions were detected in eight mutant lines, one lesion was confirmed in each of seventeen mutant lines, two lesions in seven mutant lines, three lesions in five mutant lines, four lesions in two mutant lines, and five lesions in two mutant lines (X^2(5df)^ = 3.99; p<0.001; see calculations in Materials and Methods). This finding indicates that some mutant lines could carry more than one nucleotide variant that either individually or together produce a phenotype.

Of the 23 coding base changes identified in 19 different mutant lines, only 4 were synonymous. The non-synonymous base pair changes provide a valuable list of candidate genes for the ENU-induced mutations. The genetic code assists in the interpretation of these lesions, since such base changes cause obvious defects in protein coding sequences. Three of the non-synonymous lesions result in the insertion of a stop codon, which may cause protein truncation or elicit nonsense-mediated decay. One of these mutations, C4228T, which generates Q106X in *l11Jus15*, is in a member of the mediator complex that directs transcription, mediator complex 31 (*Med31*). Sixteen non-synonymous missense mutations were identified, most of which occur as the sole lesion within the DNA that was sequenced in a line (Table 1, Table S4). Notably, we previously reported a missense mutation in the postnatal lethal line *l11Jus51*, and this line was re-sequenced as a positive control. The mutation E541V in *Slc4a1* was the only confirmed lesion identified in 6.27×10^6^ bp sequenced from this line, showing the sensitivity of detection, as well as demonstrating the relatively low frequency of ENU lesions. Two mutant lines, craniofacial08 *(crf08)* and the lethal line *l11Jus52*, contain two missense mutations each. *Crf08* has an isoleucine to valine substitution (I207V) in olfactory receptor 394 (*Olfr394*) as well as a lysine to glutamic acid substitution (K663E) in mediator complex 13 (*Med13*). *L11Jus52* has a tyrosine to cysteine substitution (Y340C) in the frizzled 2 homolog (*Fzd2*) and a glutamine to proline substitution (Q341P) in the plexin domain containing 1 gene (*Plxdc1*).

**Table 1:** Confirmed ENU–induced mutations that are potentially causative for the abnormal phenotype in each line.

Mutant Line	Phenotype of Mutant Line	Gene name	Base Change*	Lesion**	Classification†
*craniofacial* (*crf*) *02*	abnormal craniofacial morphology^1^	*Hspb9*	G653A^e^	G11R	Non-Neutral 3/78%
*crf08*	abnormal craniofacial morphology^1^	*Med13*	A49310G^e^	K663E	Non-Neutral 3/78%
		*Olfr394*	A1219G^e^	I207V	Neutral 2/69%
*crf12*	abnormal craniofacial morphology^1^	*Centb1*	T10645A^e^	V414E	Non-Neutral 2/70%
		*Med13*	C87859A^3U^	noncoding	
		*Gip*	A4968C^i^	noncoding	
		*Mpdu1* 5	T5105A^i^	noncoding	
*crf18*	abnormal craniofacial morphology^1^	*Klhl10*	A4098G^e^	K55R	Neutral 3/78%
*crf26*	abnormal craniofacial morphology^1^	*Ccdc55*	T33214A^e^	V516E	Non-Neutral 2/70%
*growth (gro) 01*	decreased body size^1^	*Dvl2* 6	T8151G^e^	F453V	Non-Neutral 2/78%
		*Traf4*	T5526A^i^	noncoding	
		*Nos2*	A29970G^i^	noncoding	
*gro22*	decreased body size	*Abr*	G51912T^i^	noncoding	
	decreased circulating cholesterol^1^	*Abcc3*	A29323G^i^	noncoding	
		*Mbtd1*	T51696C^i^	noncoding	
		*Mett10d*	T13811A^i^	noncoding	
*gro41*	decreased body size^1^	*Stac2*	C14382A^e^	R354R	
*gro42*	decreased body size^1^	*Nsf*	C106442A^i^	noncoding	
*infertile (inf)03*	female infertility^1^	*Sp6*	T10900A^3U^	noncoding	
*inf07*	female infertility^1^	*Plekhm1* 7	T18282C^e^	S209P	Neutral 1/60%
		*RP23-263M10.5*	T33177C^d^	noncoding	
		*RP23-350G1.1*	G4223A^i^	noncoding	
*lethal Chr11(l11Jus)03*	embryonic lethality (5.5–8.5 dpc^3^)	*Git1*	A10267C^i^	noncoding	
*l11Jus05*	embryonic lethality (9.5–12.5 dpc^3^)	*Med13*	T87481A^3U^	noncoding	
*l11Jus06*	embryonic lethality (9.5–12.5 dpc^3^)	*RP23-185A18.9*	C8461A^e^	C632stop	
		*Tmigd1*	T5212C^i^	noncoding	
*l11Jus08*	embryonic lethality (9.5–12.5 dpc^3^)	*Zzef1*	T95354A^i^	noncoding	
*l11Jus12*	embryonic lethality (5.5–8.5 dpc^3^)	*Map3k14* 8	A46941T^e^	M908L	Non-Neutral 2/70%
		*P140*	T39151C^i^	noncoding	
		*Tlk2*	T92972C^e^	D568D	
*l11Jus13*	perinatal lethality^3^	*Nxn*	T136735A^i^	Splice site	
		*Mlx*	T4599C^3U^	noncoding	
		*Mrpl27*	C1086A^i^	noncoding	
		*Nbr1*	A17277G^i^	noncoding	
		*RP23-396N4.2*	T91284A^d^	noncoding	
*l11Jus14*	embryonic lethality (9.5–12.5 dpc^3^)	*Stat3* 4 ^,^ 9	G43695T^i^	noncoding	
*l11Jus15*	embryonic lethality (13.5–18.5 dpc^3^)	*Med31*	C4228T^e^	Q106stop	
		*Car10*	A2624G^5U^	noncoding	
*l11Jus22*	perinatal lethality^3^	*Scpep1*	T20167C^e^	V237A	Neutral 0/53%
		*Stat5a*	T22219C^i^	noncoding	
*l11Jus27*	embryonic lethality (9.5–12.5 dpc^3^)	*Usp32*	T107950C^i^	noncoding	
		*Dhx58*	T5507C^e^	S364S	
*l11Jus48*	embryonic lethality (5.5–8.5 dpc^3^)	*Hes7* 10	A1728G^e^	K22E	Non-Neutral 3/78%
*l11Jus49*	embryonic lethality (5.5–8.5 dpc^3^)	*Acaca*	T165383C^i^	noncoding	
*l11Jus51*	postnatal lethality with anemia^1^	*Slc4a1* 11	A9600T^e^	E541V	Non-Neutral 3/78%
*l11Jus52*	postnatal lethality^3^	*Fzd2*	A1955G^e^	Y340C	Non-Neutral 4/82%
		*Plxdc1*	A53039C^e^	Q341P	Non-Neutral 3/78%
		*Aipl1* 12	A630T^5U^	noncoding	
		*Atp6v0a1*	T1610A^i^	noncoding	
		*Socs7*	T31367C^i^	noncoding	
*l11Jus54*	postnatal lethality^3^	*Fzd2*	G2447A^e^	C504Y	Non-Neutral 4/82%
*l11Jus55*	postnatal lethality^3^	*Nf1* 13	A5492G^3U^	noncoding	
*l11Jus58*	embryonic lethality (after 12.5dpc^3^)	*Cntnap1*	C17008T^i^	noncoding	
		*Mrps23*	G1465A^e^	K36K	
*neurological (nur) 07*	decreased body size,	*Aspa* 14	C13844T^e^	Q193stop	
	late onset tremors^1^	*RP23-467J12.1*	A14611G^3U^	noncoding	
		*RP23-352L3.2*	T7179A^e^	D916E	Neutral 4/85%
*nur08*	hyperactive, seizures, craniofacial^1^	*RP23-185A18.9*	T4392A^i^	noncoding	
*nur09*	hyperactive, jerky, hearing loss^1^	*RP23-136D4.2*	T134653A^3U^	noncoding	

The average amount of sequence obtained for each mutant was 80% of the 7.8Mb (one-fourth) of the 34 Mb region containing exons of annotated genes. The mouse gene symbol is shown. Location of lesion indicated by ^e^ (exon), ^i^ (intron), ^5U^ (5′ UTR), ^3U^ (3′ UTR), ^u^ (upstream), or ^d^ (downstream). No lesions were found in the mutant lines *crf05*, *inf4*, *gro40*, *l11Jus38*, *l11Jus45*, *nur01*, *nur05*, and *skc1*. Lesions were identified in the mutant lines *crf06* and *l11Jus39* that confirmed in some, but not all of the samples, indicating that the lesion was not causative of the phenotype, so these mutants are not included in the table.

*: Number refers to nucleotide position within entire genomic sequence from Ensembl v52.

**: Number refers to amino acid position within the first protein coding transcript in Ensembl v52.

**†:** SNAP analysis was used to determine the likelihood of an amino acid change being deleterious to the protein. The resulting classification is shown as Neutral or non-Neutral, along with the reliability index on a scale from 0 to 9, with 9 being the most reliable prediction, and finally, the predicted accuracy, shown as a percentage. This analysis is based on human sequence, so may not be as reliable for the mouse.

1 Kile et al. [5].

2 Clark et al. [56].

3 Hentges et al. [16].

4 *Stat3* is not the causative lesion because *l11Jus14* maps to the *Mpo-Chad* interval and *Stat3* is outside this interval.

5 Associated with mutation in human gene *MPDU1*, OMIM 6040410.

6 Associated with mouse mutant MGI 106613.

7 Associated with mutation in human gene *PLEKHM1*, OMIM 611466, and a rat mutation, MGI 2443207.

8 Associated with mouse mutant MGI 1858204.

9 Associated with mutations in human gene *STAT3*, OMIM 147060, and mouse mutant MGI 103038.

10 Associated with mouse mutation MGI 2135679.

11 Associated with mutations in human gene *SLC4A1*, OMIM109240, and mouse mutant, MGI 109393.

12 Associated with mutation in human gene *AIPL1* OMIM 604393.

13 Associated with mutation of human gene *NF1*, OMIM 601321, OMIM 607785, and mouse mutant MGI 97306.

14 Associated with mutation in human gene *ASPA*, OMIM 271900.

To predict the likelihood that an amino acid change is deleterious, we employed the SNAP (screening for non-acceptable polymorphisms) algorithm using default parameters and full-length protein coding sequences [12]. It predicts the neutrality of the mutation, calculates the percent accuracy of this analysis, and provides a reliability index (Table 1). The SNAP analysis revealed that the I207V transition in *Olfr394* is a neutral amino acid change, while K663E in the transcriptional regulator *Med13* is non-neutral, suggesting that the mutation causes a functional change. Both of the lesions in *l11Jus52*, Y340C in *Fzd2* and Q341P in *Plxdc1*, are predicted to cause a functional change. Ultimately, confirmation of candidate genes will require transgenic rescue or crosses with additional alleles.

Thirty-six of the 59 potentially causative base pair changes were found in non-coding regions of genes, including 25 within introns, 2 in 5′ untranslated (UTR) elements, 7 in 3′ UTRs, and 2 downstream of a gene (Table 1). We did not sequence conserved microRNAs in this project. However, a search of miRNA information databases revealed that none of the ENU-induced mutations lie within known miRNA sequences that may fall within or near genes. Further, no consensus splice sites or start codons were created by the lesions, though one consensus splice site was destroyed. Therefore, the noncoding lesions present more of a challenge to link cause and effect, since most of them occur within introns or 3′UTRs, and may affect gene regulation, splicing, transcript stability, translational efficacy, or may have no effect at all.

**Figure 2:**
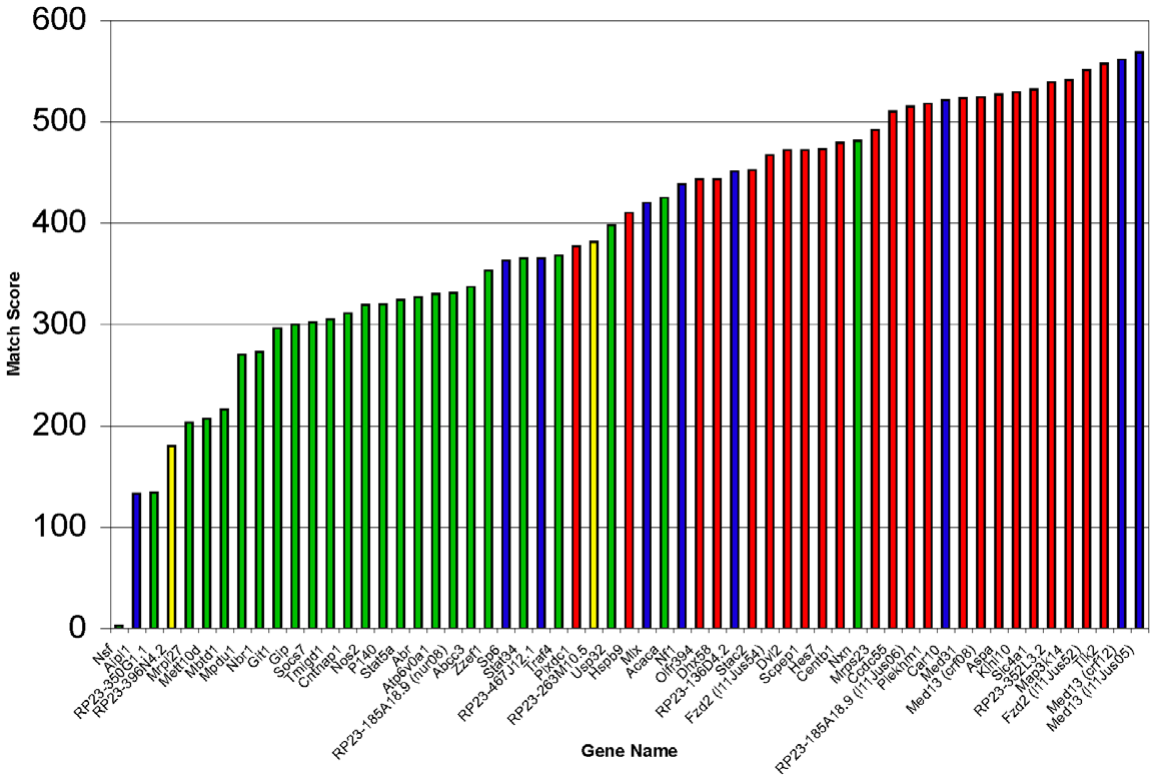
Conservation analysis of sequence surrounding ENU–induced lesions. A graph of match scores shows that exons and some non-coding elements are highly conserved. The match score is based on a 100 base pair comparison across seven vertebrates: mouse, human, rat, Rhesus monkey, horse, dog, and chicken. Red = lesion occurred in an exon, blue = lesion occurred in a 5′ or 3′ UTR, green = lesion occurred in an intron, and yellow = lesion occurred downstream of a gene.

Because multi-species sequence conservation is often a predictor of function, we compared a 100 base pair mouse sequence surrounding each ENU-induced base change to that of six other vertebrate organisms: human, rat, Rhesus monkey, horse, dog, and chicken. The comparisons produce a number based on the percent identity, which we have arbitrarily designated a “match score”. This analysis showed that the coding region mutations are conserved, as expected. However, it also showed that many of the noncoding mutations are located within highly conserved regions as well (Figure 2). All of the non-coding regions were chosen for re-sequencing because they lie near exons or are non-coding UTRs, so one may expect them to be well-conserved, similar to the coding regions. However, the coding region lesion match score averaged 493, whereas the noncoding lesion match score averaged 304. The 100 bases surrounding the T to A lesion in *l11Jus13* within a consensus splice site of nucleoredoxin (*Nxn*) produced a match score of 481. Both *l11Jus05* and *crf12* have independent mutations, T to A and C to A, respectively, in the 3′UTR of *Med13* (the same gene with a coding mutation in *crf08*), which occur in regions that produced match scores of 568 and 561, respectively (Figure 2 and Table S5). The regions surrounding these two noncoding lesions had the highest match scores in our conservation analysis. Either of these mutations may cause a change in message stability or translation, perhaps through perturbations of interactions with microRNA. Indeed, an analysis of TargetScan revealed that the lesion in *l11Jus05* lies within a seed sequence for the microRNA *miR200* [13],[14].

### A consensus splice site mutation in nucleoredoxin in the perinatal lethal line *l11Jus13*

**Figure 3:**
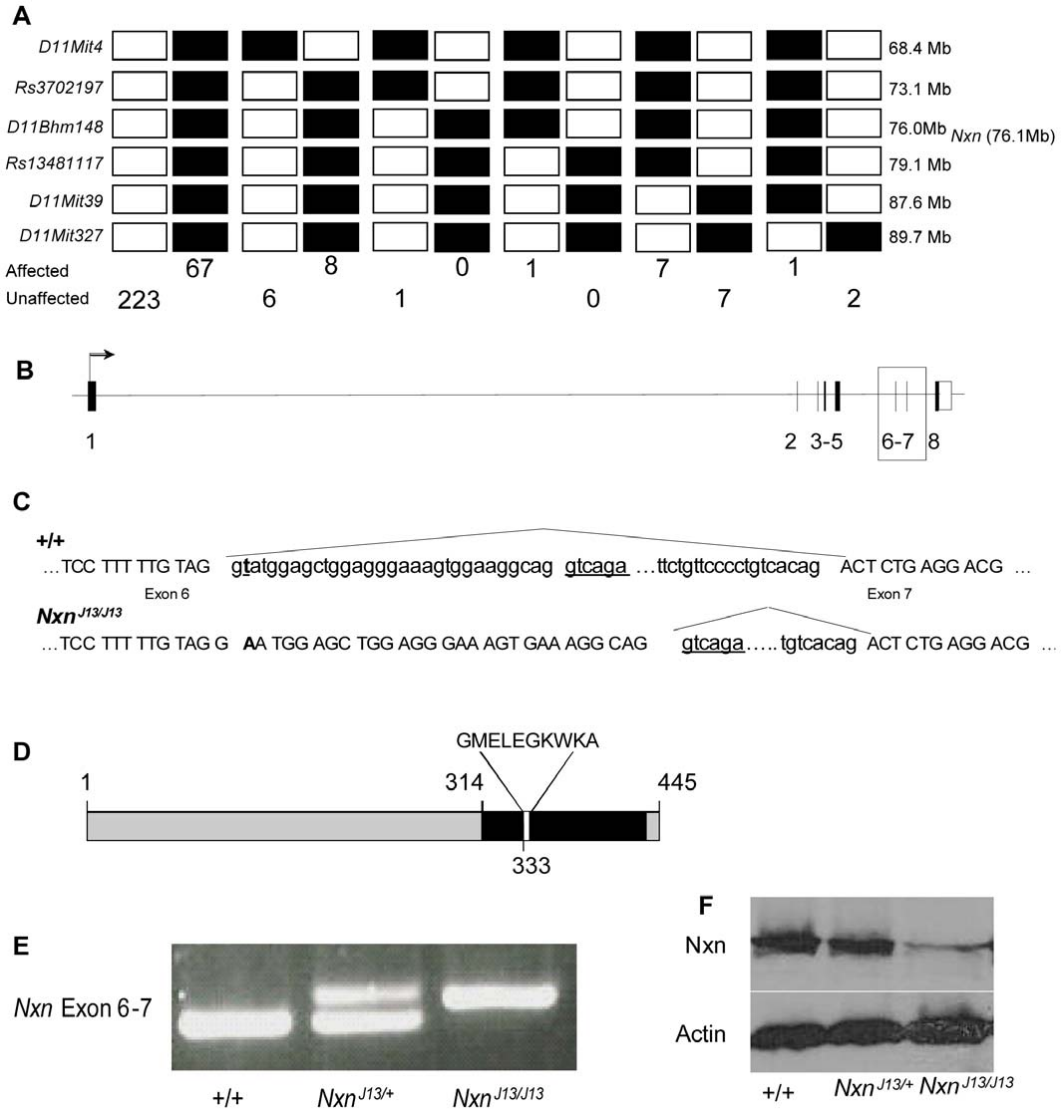
Mutation of nucleoredoxin in *l11Jus13 (Nxn^J13/J13^)*. (A) Haplotype map of 239 unaffected and 83 affected mice used for meiotic mapping. The location of each marker and *Nxn* is displayed (Ensembl v52). The mutation lies in a 6Mb region located between *rs3702197* and *rs13481117*. (B) The *Nxn* locus is depicted with introns 6 and 7 boxed. (C) The boxed region is expanded to illustrate the consequence of splicing in the wild type and mutant. A transversion (T to A) abolishes a consensus splice donor site leading to aberrant RNA splicing. The six base pair cryptic splice site used in the mutant is underlined. (D) Aberrant splicing in *Nxn^J13/J13^* predicts an in frame insertion of 10 amino acids, GMELEGKWKA, (white), which occurs within the acidic region (black) of Nxn. (E) RT–PCR using primers flanking exon 6–7 from pools of three E14.5 heads of wild-type, heterozygous, and homozygous mutants demonstrates aberrant splicing in the mutant allele. (F) Western blots of Nxn and Actin from wild-type, heterozygous and homozygous mutants at E12.5 show reduced protein in the homozygous mutant. A reduction in protein was also observed at E15.5 and E18.5, and a polyclonal antibody against the N-terminus gave similar results (data not shown).

We examined the lesions in *l11Jus13*, a line that carried five confirmed noncoding mutations in the novel Riken Protein *RP23-396N4.2*, 39S ribosomal protein L27 *(Mrpl27)*, next to *Brca1* gene (*Nbr1*), max-like protein X (*Mlx*), and nucleoredoxin *(Nxn)*. These lesions give match scores in our conservation analysis of 180, 202, 260, 410, and 481, respectively. The critical interval for the *l11Jus13* perinatal lethal phenotype was narrowed by meiotic mapping to 6 Mb of DNA extending from the SNP r*s3702197* to the SNP r*s13481117* (Figure 3A). All lesions other than that in *Nxn* are excluded from this region. The T to A lesion in *Nxn* occurs two base pairs after exon 6 to alter a consensus splice donor sequence. RT-PCR and sequencing confirmed that the mutation leads to aberrant splicing of the transcript in homozygous *l11Jus13* (*Nxn^J13/J13^*) embryos. Thirty base pairs of intronic sequence are included in the transcript, predicting an in-frame insertion of 10 amino acids into the protein, which was present in E12.5 homozygous mutants at about 30% of wild-type levels (Figure 3B–3F) [15]. A null allele of Nxn was obtained from the European Conditional Mouse Mutagenesis Program (EUCOMM), *Nxn^tm1Eucomm/+^* (*Nxn^+/−^*), and a complementation test was carried out. Thirty-four mice from crosses between *Nxn^+/−^* and *Nxn^J13/+^* were examined at weaning and none (Expected = 8) were *Nxn^J13/−^* (p<0.001).

**Figure 4:**
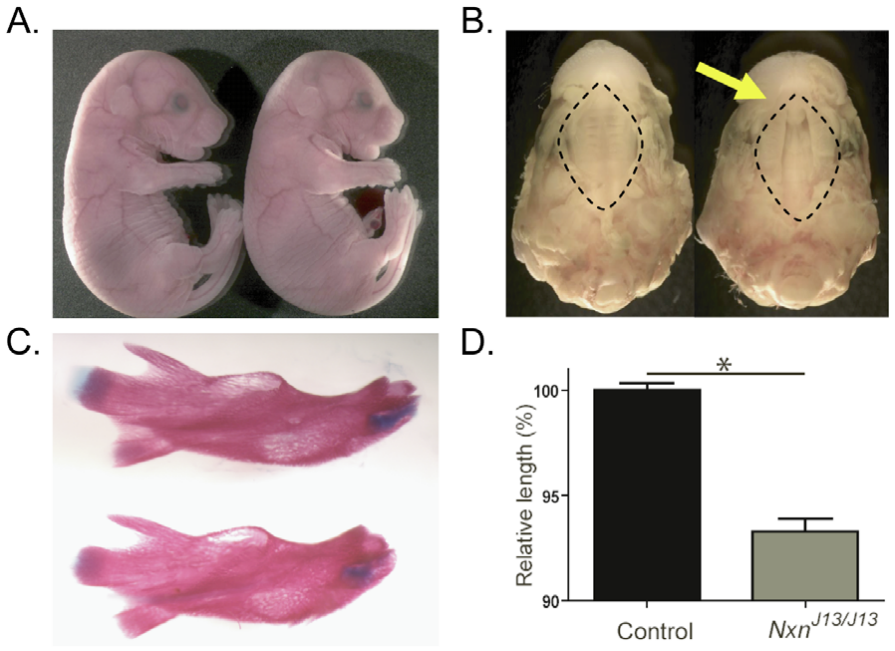
*Nxn^J13/J13^* mutants have cleft palates and small mandibles. Homozygous *Nxn^J13/J13^* embryos were compared to *Nxn^J13^*+/+*Inv* control littermates. (A) Gross morphology at E18.5. *Nxn^J13/J13^* mutants (right) have a shortened snout compared to control littermates (left). (B) The palate (inside the dotted lines) was examined at E18.5, and a cleft palate (yellow arrow) occurred in *Nxn^J13/J13^* embryos (right) but not in control littermates (left). (C) Skeletal preparations were carried out at E18.5. The mandible is shorter in length in *Nxn^J13/J13^* embryos (bottom) than in control littermates (top). (D) The mean and standard error are plotted to show the difference in mandible length. Mutant mandibles are significantly (p<0.001) shorter than controls (n = 10 mandibles per genotype).

Ninety-seven percent of the *Nxn^J13/J13^* mutants die perinatally by postnatal day 1 (P1) (Figure S2) [16]. All *Nxn^J13/J13^* embryos had craniofacial dysmorphology (Figure 4A and 4B), and most had cleft palates. Skeletal preparations at E18.5 showed that *Nxn^J13/J13^* embryos had a seven percent decrease in mandible length (p<0.001) when compared to their control littermates (Figure 4A and 4B). The decrease in bone length was not found in femurs and body mass was not significantly different at this time point, showing that this decrease was not due to a body size difference in the mutants (Figure S2).

## Discussion

Mouse Chromosome 11 is the first full mouse chromosome to be completely finished. Here, we analyzed the most conserved region between mouse Chromosome 11 and human Chromosome 17 by mutagenesis and sequencing in the largest ENU mutation identification project to date. This chromosome contains 4.6% of the total mouse genome sequence, yet contains nearly 10% of the estimated total number of transcribed genes, showing that mouse Chromosome 11 is a gene dense region. Previous data suggested that this region contains more than the average number of essential genes [17], consistent with a high recovery of mutations in the Chromosome 11 screen [5]. Classical positional cloning efforts would have required many crosses in each mutant line to narrow the critical intervals to a region small enough to re-sequence, which would have been a massive effort for 41 mutant lines.

Fifty-nine unique lesions were confirmed in 55 genes in 31 of the mutant lines. The simplest of these to associate with candidate genes are those that alter the coding sequence of a gene, although any of the lesions reported here could be predicted to be causal until proven otherwise. An F453V mutation in *Dvl2*, an activator of Wnt signalling, occurred in the growth mutant *gro01*. The knockout of *Dvl2* dies perinatally of heart defects [18], so *gro01* may be a hypomorphic allele of *Dvl2*. Two independent mutations in the Wnt receptor *Fzd2* occurred in the mutant lines *l11Jus52* (Y340C) and *l11Jus54* (C504Y). *Fzd2* has not been targeted by mutation previously. The aspartoacylase (*Aspa*) mutation in *nur07* (Q193X) represents a model of human Canavan disease, a progressive disorder of myelination [19]. By far, the majority of ENU-induced base changes that were identified occur in genes that have no known function or for which no mutations have been reported, including many full-length RIKEN cDNA sequences, a plekstrin homology domain protein (*Plekhm1*), and the mediator complex components *Med13* and *Med31*. The causative nature of four (K22E in *Hes7*, E541V in *Slc4a1*, Q193X in *Aspa*, and Q105X in *Med31*) of the candidate gene mutations has been shown by complementation tests or protein studies prior to this publication [5],[19] (and data not shown), and one in *Nxn* reported here; however, the causal nature of others remains to be demonstrated.

A large proportion of the lesions occurred in noncoding regions. Multi-species conservation analysis was carried out to predict whether these lesions lie in a significantly conserved region (Figure 4). In the lethal line *l11Jus13*, which carries 5 independent noncoding mutations, a splice-site lesion in *Nxn*, which lies in a highly conserved region, is responsible for the mutant phenotypes. Of note, the five lesions in this line were well-spaced (at 76 Mb, 94.5 Mb, 101 Mb, 101.5 Mb, and 104.5 Mb), and none other than that in *Nxn* was found in a 6 Mb critical interval that contains over 180 genes. Although the *l11Jus13* line had five base changes, it was the exception. Seventeen of the lines had only one base change in the entire 7.8 Mb of DNA that was sequenced. Together, our data suggest that in the majority of ENU-induced mouse mutant lines, accessory DNA lesions will not complicate assigning mutations to phenotypes.

*Nxn^J13^* homozygous mutants likely die as a consequence of cleft palate, which causes an inability to suckle at birth (Figure S2). Cleft lip and/or cleft palate is a common birth defect, which can be caused by the tongue protruding into the space where the palate should close during embryogenesis [20],[21]. Therefore, the cleft palate in *Nxn^J13^* homozygous mutants could be due to a physical failure of the palate to fuse as a consequence of the small mandibles causing a protrusion of the tongue. *Nxn* lies within the region commonly deleted in Miller-Dieker Lissencephaly Syndrome (MDLS—OMIM #247200). Patients with this disorder sometimes have micrognathia (small jaw), cleft palate, and heart defects, raising the possibility that *Nxn* plays a role in the pathogenesis of this disease [22]. In addition, the triad of glossoptosis (displacement of the tongue), micrognathia, and cleft palate is seen in Pierre Robin syndrome (OMIM #261800). Recently, an autosomal dominant form of this disorder was associated with deletions around the *SOX9* gene on 17q (OMIM #608160); however, there are clearly still autosomal recessive forms of this disease for which the underlying genetic lesion has yet to be identified [23], and *NXN* is a promising candidate gene for these cases. *Nxn* has been previously implicated as a negative regulator of the canonical Wnt pathway and the noncanonical PCP pathway in cell culture and in *Xenopus* [24],[25]. Disrupting the signalling of either or both of these pathways could result in the craniofacial abnormalities observed in the *Nxn^J13/J13^* mutants.

In spite of the large number of candidate genes reported here, we know that our mutation detection is incomplete. Our overall mutation rate was 2.6×10^−7^, which is within the published range for mutation rates defined after ENU treatment and sequencing (1.04×10^−6^ to 3.1×10^−7^) [26],[27]. We predict that we have identified 36–100% of the possible lesions in our mutants. Prior searches for point mutations in an allelic series at myosin light chain 5a (*Myo5a*; dilute) and bone morphogenetic protein 5 (*Bmp5*; short ears), failed to identify approximately 1/3 of the lesions within annotated coding sequences [28]–[30]. Further, two of five ENU-induced alleles of quaking (*qk*) lie outside the coding region or the 5′ and 3′ UTRs [31],[32]. Despite extensive efforts to catalogue the coding component of mouse Chromosome 11, it is likely that there are genes, particularly those expressed at discrete developmental time points or in rare cell types that have not been annotated. However, some of the missing *Bmp5* lesions were later shown to lie in regulatory regions [33]. Here, we show that many of the ENU-induced lesions lie in non-coding regions, even though our exon-based sequencing strategy targeted only 125 base pairs outside each exon along with the 5′ and 3′ UTRs. We would predict that the mutants reported here have additional lesions in non-coding regions that were not sequenced in this project or in the exon sequences that were not obtained for each mutant. Some of these may lie in regulatory regions.

The first ENU-induced mutation discovered to disrupt the sequence of a microRNA was recently reported to cause deafness [34]. MicroRNAs were not previously included in re-sequencing strategies for candidate genes, because they were not annotated. We report the first lesion in a *miR200* seed sequence in the 3′ UTR of *Med13* in the lethal line *l11Jus05*, which dies at E 8.5 with cardiovascular and neural tube defects [16]. *Med13* is a component of the mediator complex, which associates with RNA Polymerase II to direct transcription. It is expressed during embryonic development and throughout the brain and skeleton in the adult [35]. The mediator complex is required during development as evidenced by the fact that deletion of the components *Med1* and *Med21* produce embryonic lethal phenotypes due to cardiac defects [36],[37]. *MiR200* is required for the mesenchymal/epithelial transition during embryonic development and is involved in cancer metastasis [38],[39]. Further studies of this mutation will help us to determine how *miR200* regulates *Med13*. Future sequencing efforts in any project designed to identify causes of mutation or disease should include microRNAs, 3′ and 5′UTRs, and any other highly-conserved noncoding regions. Although the functional nature of many conserved noncoding regions is not apparent, perhaps some of our mutations will allow us to determine the “genomic code” in non-coding conserved sequences.

As polymorphisms are detected in Genome Wide Association Studies (GWAS), the correlation of SNPs or copy number variation with disease will require knowledge of the biological function of each gene. Of the over 900 annotated transcripts in the *Trp53-Wnt3* interval, 27% are associated with mutant phenotypes from gene targeting or spontaneous mutation (Table S6). However, only six of the candidate genes reported here have been targeted by mutation in the mouse (*Mapk14* [40],[41], *Hes7* [42], *Slc4a1* [43], *Stat3* [44], *Nf1* [45], *and Dvl2* [18]), and only one in the rat (*Plekhm1*) [46]. Seven genes are associated with disease mutations in the Online Mendelian Inheritance in Man database (OMIM) (*MPDU1* [47], *PLEKHM1* [46], *STAT3* [48], *SLC4A1* [49], *NF1* [50], *AIPL1* [51] and *ASPA* [52]), of which five are associated with mutations in mouse or rat (Table 1). Therefore, prior to this study, function was associated with only nine of the genes we report, showing that the majority of the lesions reveal new functions for the candidate genes. In aggregate, these data show that functional annotation of the mouse genome is still in its infancy, especially when one considers the low degree of saturation of our mutagenesis screen [17]. The mouse genome has a tremendous potential to provide biological and experimental annotation of both genes and noncoding conserved sequences relevant to GWAS, if this gap between function and phenotype is to be bridged.

Here we show that re-sequencing must no longer be restricted to a few candidate genes. Faster, cheaper, high-throughput methods for re-sequencing reduce the need for narrowing candidate gene intervals to small regions by meiotic mapping. Targeted re-sequencing using Next Generation (NextGen) technologies should be attempted for mutation detection in additional mutant lines that map to restricted molecular intervals. The classical microcapillary sequencing method has a relatively low error rate. However, comparisons of the efficacy of the various NextGen methods show that each is sequence context dependent [53],[54]. Regardless of error rate, cost or ease of use, our data show that although most sequencing methods are exon-based, strategies used for mutation detection should include conserved non-coding as well as coding sequences, also known as the “conservome” [54]. Altogether, accurate genome sequence and cheaper sequencing technologies provide a new avenue for understanding genomes, genome evolution, disease mutations and biological function.

## Materials and Methods

### Ethics statement

All animal work was approved by the Institutional Animal Care and Use Committee (IACUC). Our animal facility is accredited by the Association for Assessment and Accreditation of Laboratory Animal Care International (AAALAC).

### Mapping, sequencing, and sequence analysis

The sequence of mouse Chromosome 11 was generated using a hierarchical strategy to generate a sequence-ready physical map of the chromosome followed by clone-by-clone sequencing to generate high-quality finished sequence.

Alignments for cross species comparative analysis were performed with WU-BLASTN (http://blast.wustl.edu) using the finished sequence assembly of mouse Chromosome 11 (National Center for Biotechnology Information (NCBI) build 37) and of the human genome (NCBI 37). All sequences were repeat-masked with RepeatMasker (http://repeatmasker.genome.washington.edu) and low-quality alignments (*E*-value >10^−30^) were removed prior to analysis.

We performed manual annotation of the finished mouse Chromosome 11 following the human and vertebrate analysis and annotation (HAVANA) guidelines (http://www.sanger.ac.uk/HGP/havana/) to identify 2,545 gene structures (approximately 30% higher than previous computational predictions alone), which include 1,597 protein-coding loci, 450 processed transcripts, and 498 pseudogenes (Table S2). Before the process of manual annotation, an automated analysis pipeline for similarity searches and *ab initio* gene predictions was run, and the resulting data were manually annotated using the graphical in-house annotation tool “otterlace”. Manual gene annotation is available in Vega (http://vega.sanger.ac.uk/index.html) [55]. Protein coding loci were subcategorized into known and novel loci depending on whether the cDNA had an entry in RefSeq (human loci) or Mouse Genome Database (mouse loci). If no open reading frame could be determined the locus was classified as a transcript and labeled novel or putative, depending on level of supporting evidence.

### ENU mutagenesis balancer screen and exon sequencing

The Chromosome 11 ENU mutagenesis screen was performed as described previously using C57BL/6J ENU-treated males and the 129.*Inv(11)8Brd^Trp53-Wnt3^* balancer chromosome [4],[5]. The inversion was generated in 129S5SvEv ES cells, and restricts the recovery of viable recombination products, so the region should remain 129S5 in subsequent crosses. After isolating a line based on its phenotype, animals were mated at least four times (N4) to a 129S6/SvEv or congenic 129S6.*Rex* line to allow for recombination, and then each line was maintained *in trans* to 129.*Inv11(8)Brd*. Sequences for the exons and their 1kb flanking sequences were extracted from Vega for all known protein-coding genes, novel coding sequences, and transcripts in the target region. Repeats in the sequence were masked using RepeatMasker (http://www.repeatmasker.org/) prior to primer design. Primers were designed automatically using Primer3 (http://frodo.wi.mit.edu/) to amplify each exon and at least 125bp on either side of the exon with an optimum amplicon size of 450–550bp. A series of overlapping primer pairs was designed for each larger exon to obtain complete coverage. Any exons failing automatic primer design had primers designed manually. Primer pairs were checked for uniqueness prior to ordering and pre-screened to determine the optimum conditions for amplification.

Amplification was routinely performed on 48 DNA samples with 8 sequence tagged sites (STSs) for each sequence run. For initial large-scale sequencing, only one DNA from each mutant line was used. Because 7.8 Mb of transcribed linear DNA was sequenced per line for both strands, a total of over 560 Mb of DNA sequence was analyzed. One line reported here, *l11Jus48*, was not sequenced for the entire region because the causative lesion was found by concurrent candidate gene sequencing. The majority of exons were amplified at 60°C. After amplification, an aliquot of the product was visualized on an agarose gel. Prior to sequencing, the remaining PCR product was purified using Exonuclease 1 and Shrimp Alkaline Phosphatase. Bi-directional sequencing of amplicons was carried out using Big Dye chemistry. For more details, please refer to http://www.sanger.ac.uk/humgen/exoseq/. SNPs were called using ExoTrace (http://www.sanger.ac.uk/humgen/exoseq/analysis.shtml), a novel algorithm developed in-house for the detection of sequence variants. The program works by comparing actual peak heights with the expected peak height for a homozygous base. A base is called as homozygous if the relative peak height in a single channel exceeds a threshold and the signal in all other channels is significantly smaller than the expected peak height. A base is called as heterozygous if the signal in two channels is approximately half the expected homozygous peak height and there is no significant signal in the other channels. ExoTrace processes the sense and antisense sequence reads separately and subsequently combines the results to allow SNP scoring. Each SNP is assigned a status according to a set of pre-defined rules. All SNPs below a certain threshold were subjected to manual review using a modified version of GAP4, part of the Staden Sequence Analysis Package software (http://staden.sourceforge.net/), created for the ExoSeq project.

### Re-sequencing for confirmation of mutations

Eighty-one of 1727 sequence variants that were identified in first pass sequencing were chosen for re-sequencing. Comparisons of sequence from each mutant line against every other line provided a control for SNPs in the B6 and 129 substrains. ENU-induced lesions that are causative are expected to occur only once in a single mutant line. Primers were designed manually using Primer3 (v.0.4.0, http://frodo.wi.mit.edu) to flank the candidate mutations (Table S7). Genomic DNA was phenol-chloroform extracted from livers, embryos, or tails from homozygous or heterozygous mutants was PCR-amplified and sequenced directly using the PCR primers and BigDye Terminator v3.1 (Applied Biosystems) according to the manufacturer's instructions. The sequencing chromatograms were analyzed with Sequencher 4.7. The locations of the mutations are displayed on Ensembl v52.

### Mouse strains and genotyping

*The l11Jus13 (Nxn^J13^)* mutation is maintained *in trans* to *129.Inv(11)8Brd^Trp53-Wnt3^* [4]. The *Nxn^J13^+/+Inv* line was crossed five times to congenic 129S6.*Rex+/+Inv* mice, to recover *Rex+/+Nxn^J13^* animals and allow for recombination. Mice were genotyped with *D11Mit327* or *D11Mit132* for embryonic and neonatal studies prior to the identification of the causative lesion. The line was crossed four additional times to the 129S6/SvEvTac inbred mouse strain after the mutation was found, creating a new congenic line 129S6.*Nxn^J13^*. DNA was prepared from mouse tails, embryonic tissue, or yolk sac by either phenol/chloroform extraction or alkaline lysis (tissue is treated with 50 mM NaOH at 95°C for 20 minutes, followed by neutralization with 1/5 volume of 0.5 M Tris, pH 8.0). Genotyping of embryos, neonates, and adult mice was performed by PCR analysis of at least two STS markers flanking nucleoredoxin. Primer pairs for three different STS markers were used: 1) *D11Bhm148* (Forward primer 5′- AGGGGAAGTCCTGTATGGACA-3′ and Reverse primer 5′-ACCAACCTCGATAGAGCCATC-3′), 2) r*s13481111* (F-GTAAGGACAAAGAGGACTGCCAAG and R-AATGACAGACAGGAGGAAATCCAT), or 3) *D11Mit245* (F-ATGAGACCATGCTCCTCCAC and R-TTGTCCTCTGACCTTCACACC). The PCR mixture contained 5× Promega GoTaq PCR buffer, 0.3 mM dNTPs, 0.5 µM primer mix, 250 ng template, and 0.25 U Taq Polymerase (New England Biolabs). Cycling conditions for *D11Bhm148* were: 94°C 5 min; 40 cycles of 94°C 45 sec, 55°C 45 sec, 72°C 45 sec; then 7 min at 72°C; followed by incubation at 4°C. The PCR products (C57BL/6J - 97 bp, 129SvEvTac - 107 bp) were resolved on 4% NuSieve gels (Lonza). Cycling conditions for r*s13481111* were: 94°C 5 min; 40 cycles of 94°C 45 sec, 57°C 45 sec, 72°C 45 sec; then 7 min at 72°C; followed by incubation at 4°C. Digestion of the 236 bp PCR product with *Sau*96I (New England Biolabs) yields 2 bands for C57BL/6J (72 and 164 bp) and one for 129SvEvTac (236bp band remains uncut). These products were resolved on 2.5% SeaKem LE agarose gels (Lonza). The cycling conditions for *D11Mit245* were: 94°C 5 min; 40 cycles of 94°C 45 sec, 60°C 45 sec, 72°C 45 sec; then 7 min at 72°C; followed by incubation at 4°C. The PCR products (C57BL/6J - 152 bp, 129SvEv - 140 bp) were resolved on 5% MetaPhor gels (Lonza).

To generate the *Nxn^tm1EUCOMM^* (*Nxn^−/−^*) allele, C57BL/6N JM8 ES cells containing a multipurpose conditional “knockout-first” construct targeted to *Nxn* were obtained from the European Conditional Mouse Mutagenesis Program (EUCOMM). These ES cells were injected into C57BL/6^Brd^ *Tyr* ^−/−^ blastocysts and implanted into pseudopregnant mothers. Male chimeric offspring with black and white coats were then mated to C57BL/6^Brd^ *Tyr* ^−/−^ females, and black progeny were genotyped for the knockout-first allele with the following primers: NxnFor (TTGGGTATGCCCGACTCCCCCACC), NxnRev (CCTTCAGCCCTCTCCTTTCTGTGC), and *lox*Prev (TGAACTGATGGCGAGCTCAGACC). Two PCR reactions were set up for each sample, one with NxnFor and NxnRev, which gives a 435 bp PCR product from the mutant and a 568 bp product from wild-type, and one with NxnFor and *lox*Prev, which gives a 228 bp PCR product from the mutant, but none from the wild-type. PCR conditions were: 5× Promega GoTaq PCR buffer, 0.3 mM dNTPs, 0.5 µM primer mix, 250 ng template, 1M betaine, 0.25 U Taq Polymerase (New England Biolabs). The cycling conditions were as follows: 94°C 5 min; 40 cycles of 94°C 45 sec, 56°C 45 sec, 72°C 45 sec; then 7 min at 72°C; followed by incubation at 4°C. The PCR products were resolved on 2% agarose gels.

### Meiotic mapping

Meiotic mapping was performed by crossing *Nxn^J13^+/+Inv* to congenic 129S6.*Rex/Rex* mice, followed by intercrossing *Nxn^J13^+/+Rex* mice, and examining the progeny for recombination events. Embryos and neonates with an abnormal facial structure and a cleft palate were classified as affected progeny (n = 82), and mice that survived to weaning were classified as unaffected progeny (n = 239). Two or more polymorphic markers between the C57BL/6J and 129S6/SvEvTac strains were analyzed for every DNA sample, which was obtained from embryonic tissue or adult mouse tails by phenol/chloroform extraction. The following microsatellite markers were assessed: *D11Mit4*, *D11Mit219*, *D11Mit322*, *D11Bhm148*, *D11Mit245*, *D11Mit120*, *D11Mit324*, *D11Mit39*, *D11Mit327*, *D11Mit132*, and *D11Mit333*. The following SNPs were assessed: r*s3702197*, r*s13481111*, r*s13481113*, r*s13481117*, and r*s13481125*. Primer sequences and restriction enzymes used to digest the SNPs are shown in Table S8.

### RT–PCR and sequencing

RNA was made from pools of three E14.5 heads and livers with RNA STAT-60 (Tel-Test), and treated with DNase I (Invitrogen) following the manufacturer's instructions. Two micrograms of RNA were reverse transcribed into cDNA using SuperScript III First-Strand Synthesis System for RT-PCR (Invitrogen) with random hexamers following the manufacturer's instructions. *Gapdh* was used as a positive control using the following primers: F-CGGAGTCAACGGATTTGGTCGTAT and R-GCCTTCATGGTGGTGAAGAC. Cycling conditions were: 94°C 5 min; 30 cycles of 94°C 30 sec, 60°C 30 sec, 72°C 30 sec; then 7 min at 72°C; followed by incubation at 4°C. *Nxn* RT-PCR was carried out using the following primers: F-GGTGCTCAATGACGAGGACT and R-GCCTCCTCTTCTTTGGCTTT, which amplified the junction between exons 6–7 (213 bp wild-type product and 243 bp mutant product). Cycling conditions were: 94°C 5 min; 35 cycles of 94°C 45 sec, 60°C 45 sec, 72°C 45 sec; then 7 min at 72°C; followed by incubation at 4°C. The PCR products were gel extracted, cleaned with Zymoclean Gel DNA Recovery Kit (Zymo Research), and sequenced directly using the PCR primers and Big Dye Terminator v3.1 (Applied Biosystems). The Big Dye terminator was removed with Centri^.^Sep 8 (Princeton Separations), according to the manufacturer's instructions. Sequencing was performed by the Child Health Research Center (Baylor College of Medicine) and the sequencing chromatograms were analyzed with Sequencher 4.7.

### miRNA analysis

The miRBase Sequence Database (http://www.mirbase.org/) was used to identify 18 known miRNA sequences that lie within the Chromosome 11 balancer region. Two miRNAs, mmu-mir-324 and mmu-mir-423, lie within genes in which mutations were found, dishevelled 2 (*Dvl2*) and coiled-coil domain containing 55 (*Ccdc55*), respectively, though neither mutation identified in these genes is within the miRNA sequence itself. Target Scan 4.2 (http://www.targetscan.org/) was used to determine if any of the mutations that did not cause amino acid changes had altered a known conserved miRNA target.

### Western blot analysis

Total embryo protein extracts were prepared by grinding embryos in reducing 2× SDS sample buffer (200 mM Tris-HCl pH 6.8, 3% w/v SDS, 20% v/v glycerol, 10% v/v β–mercaptoethanol, 4% v/v saturated bromophenol blue solution) using a Tekmar electronic tissue homogenizer. Samples were resolved by SDS-PAGE on a precast 4–20% Tris-HCl polyacrylamide gel (Bio-Rad) and transferred to a Hybond ECL nitrocellulose membrane (Amersham). Nucleoredoxin was visualized using the previously described polyclonal anti-Nxn antibody [24], which was made against full-length protein and purified against a C-terminal fragment. Experiments were repeated using a polyclonal antibody made against an N-terminal fragment of Nxn as well [24]. Primary antibody (1∶1000 of 0.5 mg/mL stock) incubation was followed by an anti-rabbit secondary antibody (1∶10,000 of 0.8 mg/mL stock) linked to horseradish peroxidase (Jackson Immunoresearch) and detected on Hyperfilm ECL film using the ECL Plus Western Blotting Detection kit (Amersham) according to the manufacturer's instructions. Actin was used as a loading control and was visualized similarly using the rabbit anti-actin antibody (A2066) from Sigma.

### Embryo and neonate examination

Timed matings were carried out on intercrosses between *Nxn^J13^+/+Inv*×*Nxn^J13^+/+Inv* and *Nxn^J13/+^*×*Nxn^J13/+^*mice. The day that a vaginal plug was observed was designated E0.5. Embryos were dissected at E15.5 and E18.5, visualized by light microscopy with a Leica microscope (Diagnostic Instruments), and photographed using a SPOT digital camera. Mice at E18.5 were weighed with a laboratory balance (Mettler Toledo) and Student's t-tests were carried out to determine significance.

### Skeletal preparations

Whole-mount skeletal/cartilage preparations were carried out with solutions containing alcian blue, which stains cartilage, and alizarin red, which stains mineralized bone. The skin and the internal organs were removed from E18.5 and P0 mice, fixed overnight in 95% ethanol, stained overnight with an alcian blue solution (0.015% alcian blue 8GX from Sigma, 20% acetic acid, 80% ethanol), transferred to 95% ethanol for at least three hours, transferred to 2% KOH for at least 24 hours, stained overnight with an alizarin red solution (0.005% alizarin sodium sulfate from Sigma, 1% KOH), cleared for at least two days with 1% KOH/20% glycerol, and stored in a 1∶1 mix of glycerol and 95% ethanol. The entire procedure was carried out at room temperature. Adobe Photoshop 6.0 was used to measure the lengths of mandibles and femurs and Student's t-tests were performed to determine if significant differences in bone length occurred.

### Calculations of mutation distribution and mutation rate

1. Poisson distribution of lesions:
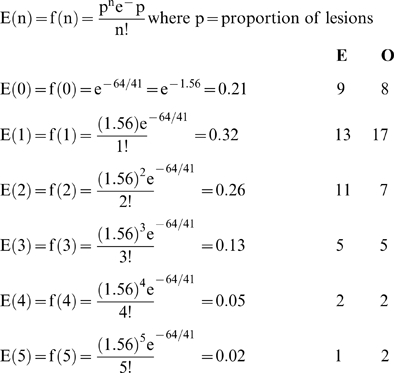
Chi-square goodness of fit X^2^ = 3.99 5 degrees of freedom
2. Mutation rate:
  17,414 STSs were designed of which 15,673 were successfully amplified.
  Average 500 bp per read = 7.8 Mb linear transcribed sequence per mutant line.
  Average 80% successful reads = 6.27 Mb per line.
  64*/6.27×10^6^×40* mutants = 2.6×10^−7^
  *The lesion in *Hes7* is not included here because the *l11Jus48* line was not sequenced for the full linear transcribed sequence.

## Supporting Information

Figure S1 Sequencing chromatograms of confirmed ENU-induced mutations. All chromatograms are shown with control strain sequence on top and heterozygous or homozygous mutant sequence on bottom. † Forward strand sequence shown in chromatogram.
(8.15 MB DOC)

Figure S2 *Nxn^J13^* mutants. (A–D) Comparison of femur length with mandible length in *Nxn^J13/J13^* mutants. At the top are the mandibles showing a seven percent difference in length (also shown in Figure 4). At the bottom are the femurs showing no significant difference in length (p = 0.29). Error bars show the 95% confidence interval around the mean (n = 10 per genotype, mandibles and femurs). Controls were all heterozygous animals. (E) Intestines in control and mutant animals at P0. Homozygous mutants have air in the intestines, indicating a suckling defect. (F) Genotypes of offspring from *Nxn^J13^*+/+Inv×*Nxn^J13^*+/+Inv matings. (A) Homozygous Inv(11)8Brd disrupts Wnt3, and no mice were obtained with this genotype, therefore the expected ratio is 2∶1. (B) Fisher exact tests with two-tailed p values.
(0.14 MB TIF)

Table S1 Sequence contigs on mouse Chromosome 11. The cumulative gap size is approximately 100 kb and centromere (3 Mb allocated in Accessioned Golden Pathy) (AGP). Note: Telmeric sequence reached. * Optical Map is a technique for generating a high resolution map of the structure of a chromosome or genome.
(0.02 MB DOC)

Table S2 Gene content differences between mouse and human.
(0.03 MB DOC)

Table S3 Structural characteristics of annotated genes structures from mouse Chromosome 11. ^1^ Of these, 450 were processed transcripts that were not likely to encode a protein.
(0.04 MB DOC)

Table S4 Variants found by resequencing exons and their flanking 125 bp in the *Trp53-Wnt3* interval. Lesions found only once in a single line are shown. Each lesion was confirmed by re-sequencing 2–4 DNAs from additional mutant individuals from each line. The B6/129 SNPs are likely to be new SNPs that arose in our substrain, or may not have been identified. No mutations were found or confirmed in 8 lines (*crf05*, *inf4*, *gro40*, *l11Jus38*, *l11Jus45*, *nur01*, *nur05*, and *skc1*). *^Hom:^* Indicates that DNA from a homozygous mutant was used for re-sequencing. *^Het:^* Indicates that DNA from a heterozygous mutant was used for re-sequencing. F: Denotes forward gene, forward strand sequence provided (coding sequence). ^R:^ Denotes reverse gene, reverse complement of forward strand sequence provided (coding sequence). ^1^ Twelve lesions did not confirm in any animals giving an error rate for re-sequencing of 15% (12/80). ^2^ Mutation was discovered independently of chr 11 resequencing effort. ^3^ Unable to successfully PCR and amplify and sequence the genomic region flanking this mutation so this mutation was not confirmed. ^4^ Kile, B.T. et. al. Nature 2003 [5]. ^5^ Traka, M. et. al. J. Neurosci. 2008 [19].
(0.22 MB DOC)

Table S5 Allelic series found. Exon number, intron numbers, and the size of the genomic loci are taken from Ensembl version 34. ^1^ Four additional lesions found in *Aipl1*, *Atp6v0a1*,*Plxdc1*, and *Socs7*. ^2^ One additional lesion found in *Tmigd1*. ^3^ Two additional lesions found in *Plekhm1*, *RP23-263M10.5*. ^4^ One additional lesion found in *Olfr394*. ^5^ Three additional lesions found in *Centb1*, *Gip*, *Mpdu1*.
(0.05 MB DOC)

Table S6 Known genes associated with mutant alleles that lie in the *Trp53-Wnt3* interval.
(1.05 MB DOC)

Table S7 Primers used to confirm Chromosome 11 ENU-induced mutations.
(0.12 MB DOC)

Table S8 Microsatellite markers and SNPs used in mapping *l11Jus13* (*Nxn^J13^*). PCR conditions are available upon request. F denotes forward primer, R denotes reverse primer.
(0.06 MB DOC)
