# Supplementary material for: Discovery of Candidate Disease Genes in ENU–Induced Mouse Mutants by Large-Scale Sequencing, Including a Splice-Site Mutation in Nucleoredoxin
Source: PLoS Genet. 2009 Dec 11;5(12):e1000759. doi: 10.1371/journal.pgen.1000759 (PMC2782131; doi:10.1371/journal.pgen.1000759)
Supplement: Figure S1 — Sequencing chromatograms of confirmed ENU-induced mutations. All chromatograms are shown with control strain sequence on top and heterozygous or homozygous mutant sequence on bottom. † Forward strand sequence shown in chromatogram. (8.15 MB DOC) [file pgen.1000759.s001.doc]

**Figure S1: Sequencing chromatograms of confirmed ENU-induced mutations**


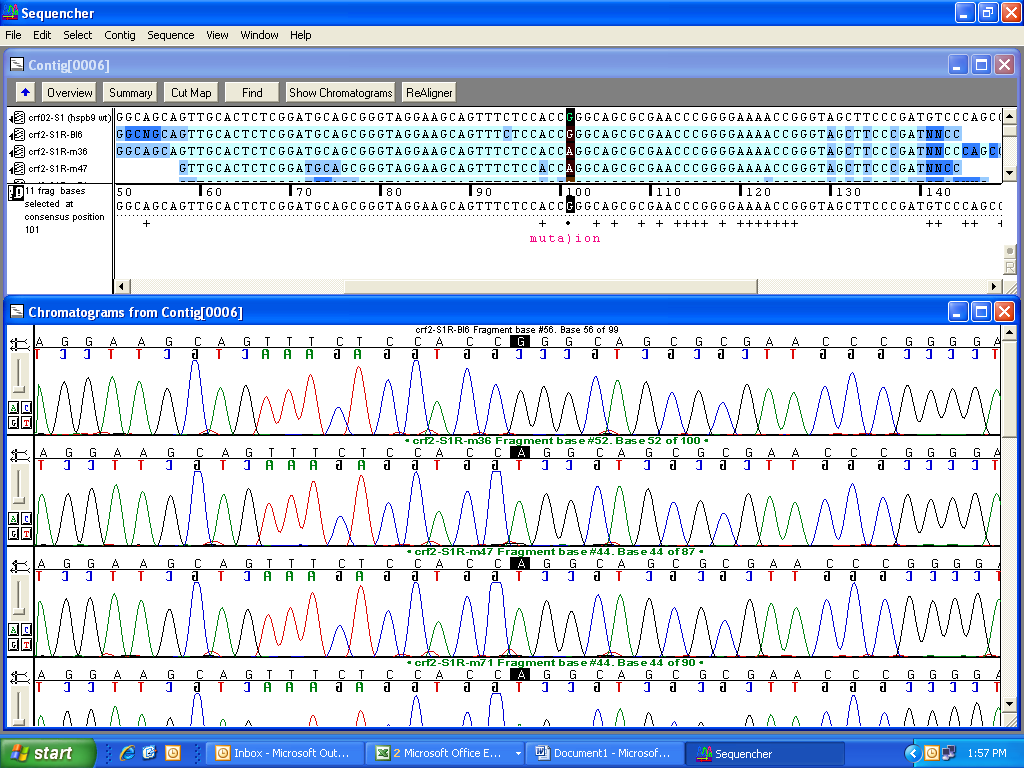
**Mutant Line:** *crf02*

**Gene Name:** *Hspb9*

**Chr. 11 base #[[1]](#endnote-2):** 100535061

**Base Change:** G to A^F^


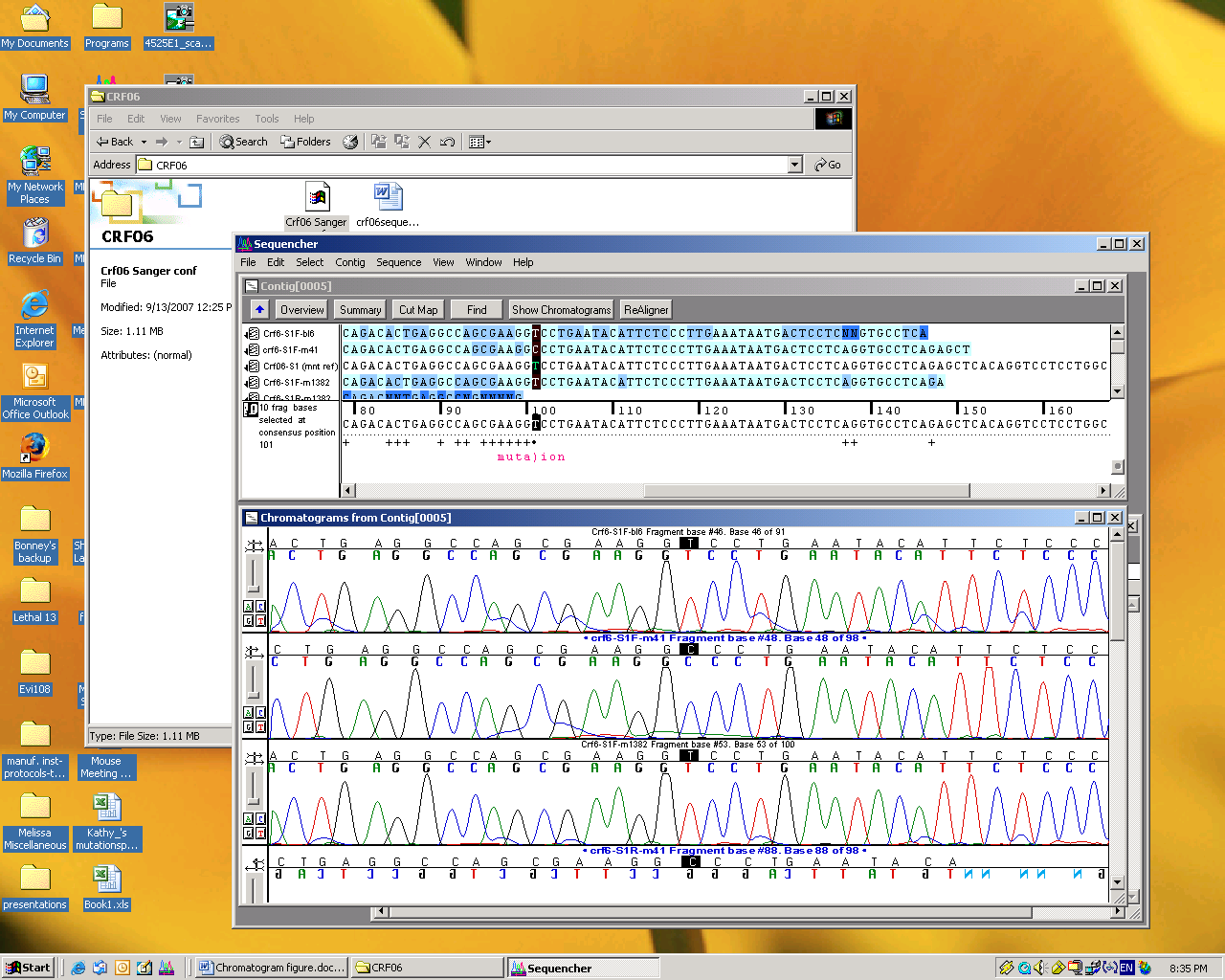


**Mutant Line:** *crf06*

**Gene Name:** *Mnt*

**Chr. 11 base #:** 74570213

**Base Change:** T to C^F^

**
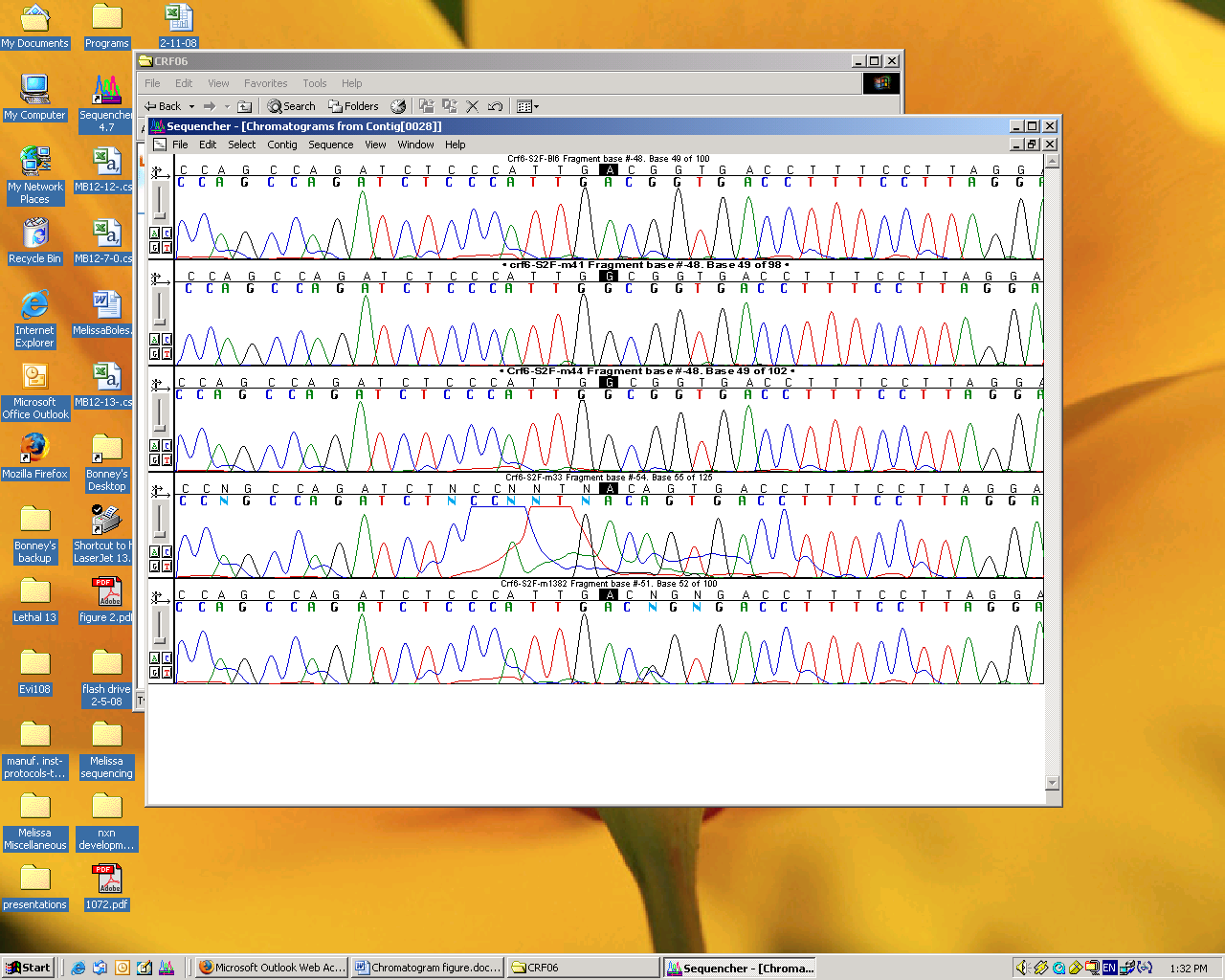
**

**Mutant Line:** *crf06*

**Gene Name:** *Plscr3*

**Chr. 11 base #:** 69575985

**Base Change:** A to G^F^

**
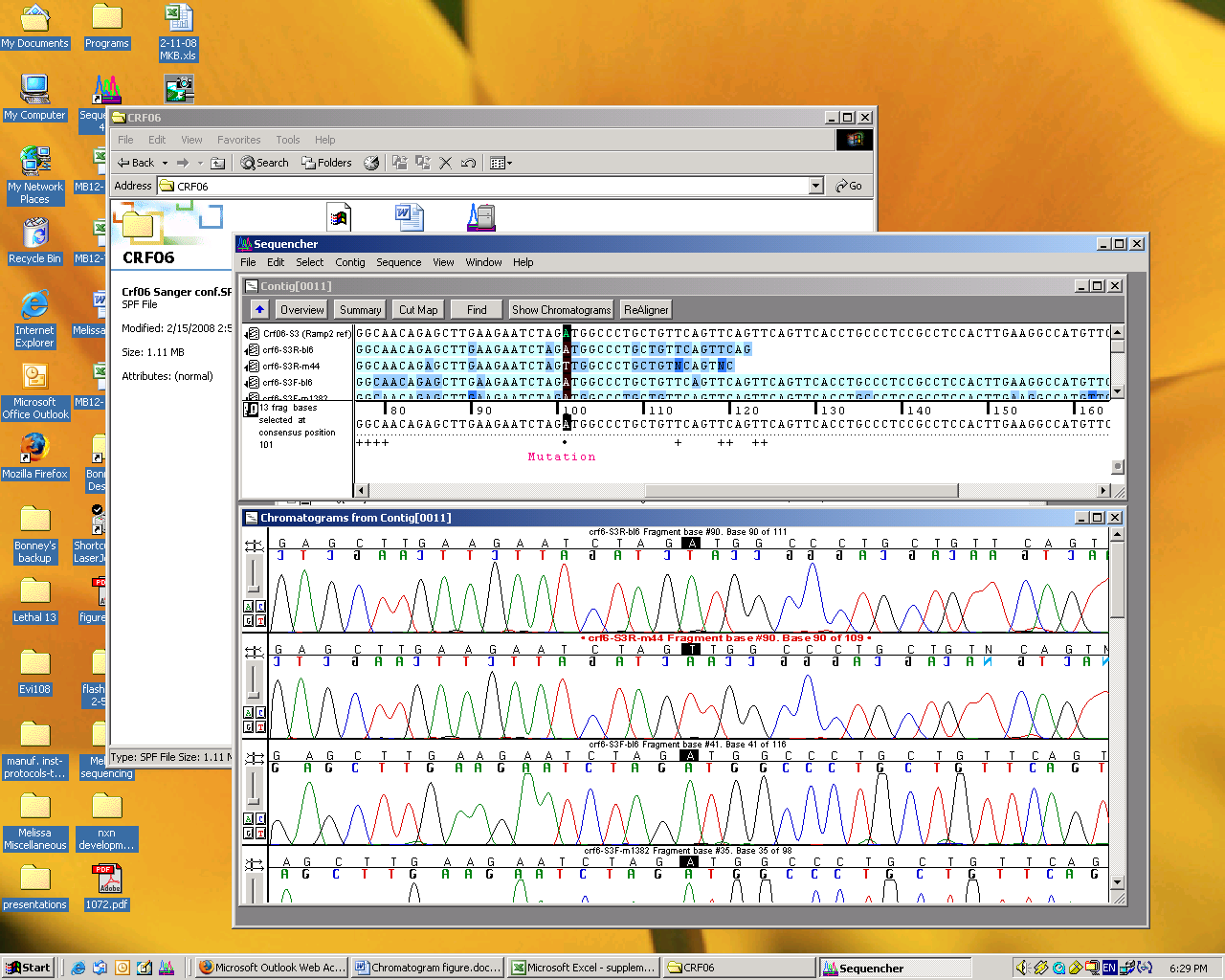
**

**Mutant Line:** *crf06*

**Gene Name:** *Ramp2*

**Chr. 11 base #:** 101069419

**Base Change:** A to T^F^

**
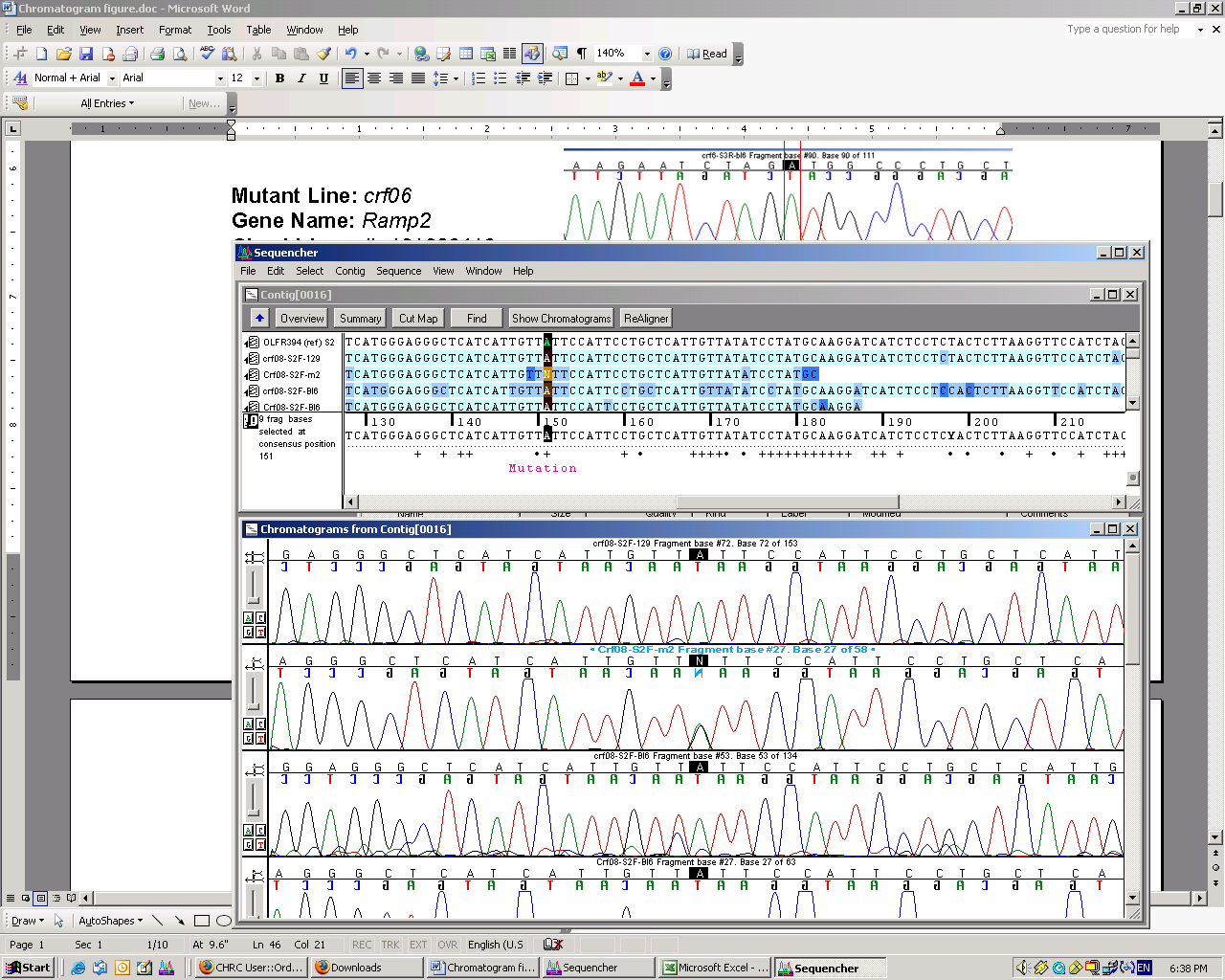
**

**Mutant Line:** *crf08*

**Gene Name:** *Olfr394*

**Chr. 11 base #:** 73613411

**Base Change:** A to G^R^


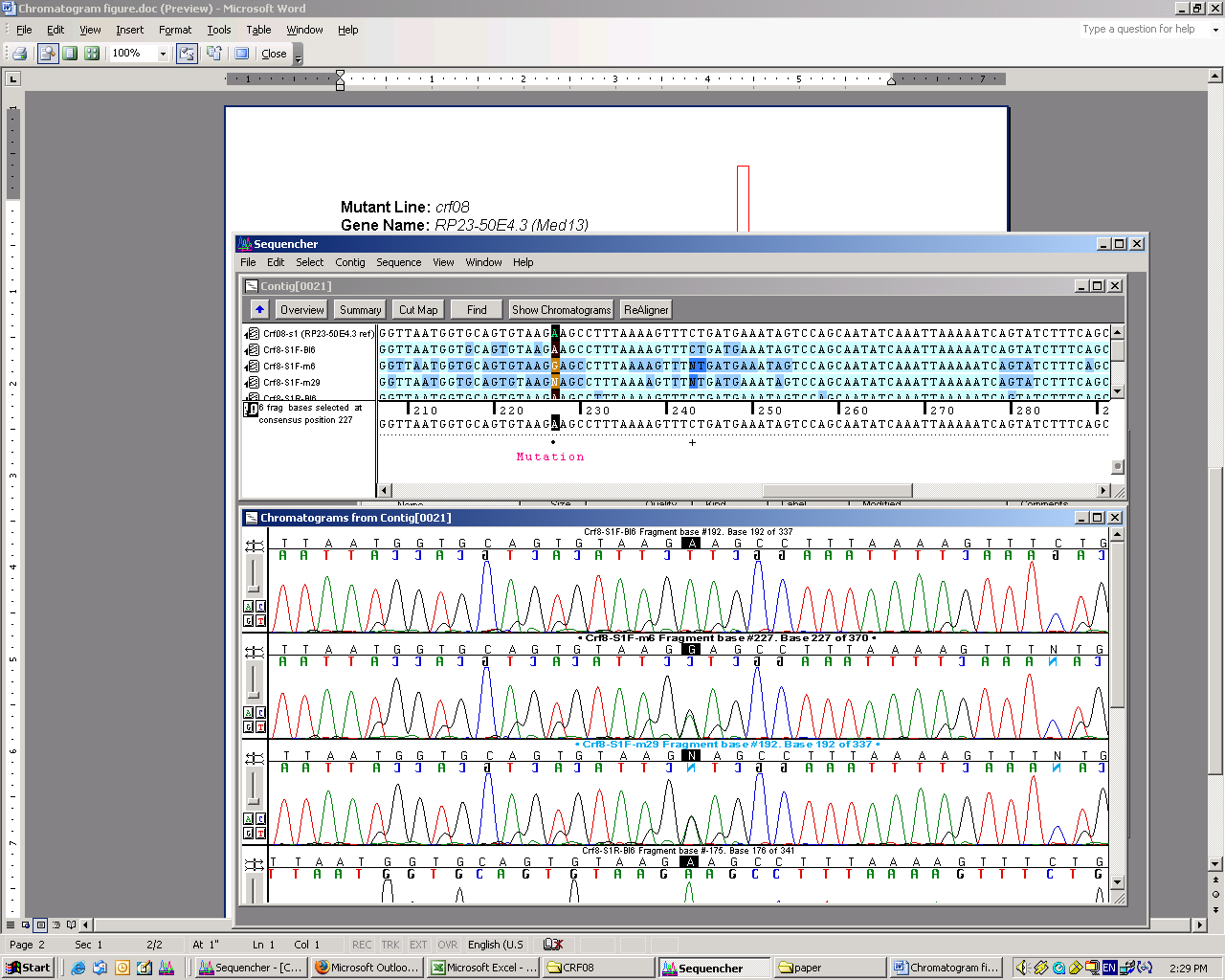
**Mutant Line:** *crf08*

**Gene Name:** *Med13*

**Chr. 11 base #:** 86036886

**Base Change:** A to G^R^

**
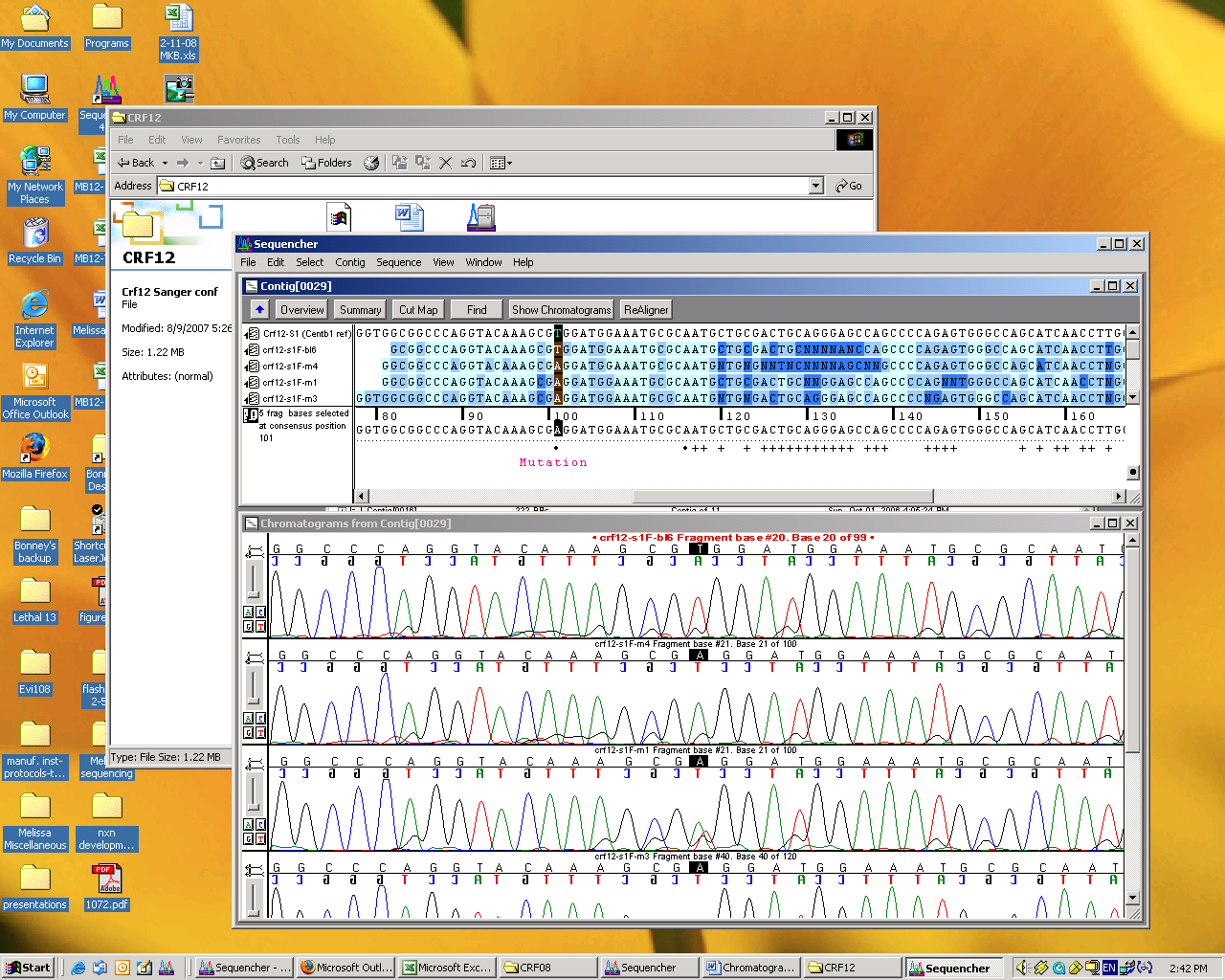
**

**Mutant Line:** *crf12*

**Gene Name:** *Centb1*

**Chr. 11 base #:** 69611154

**Base Change:** T to A^R^

**
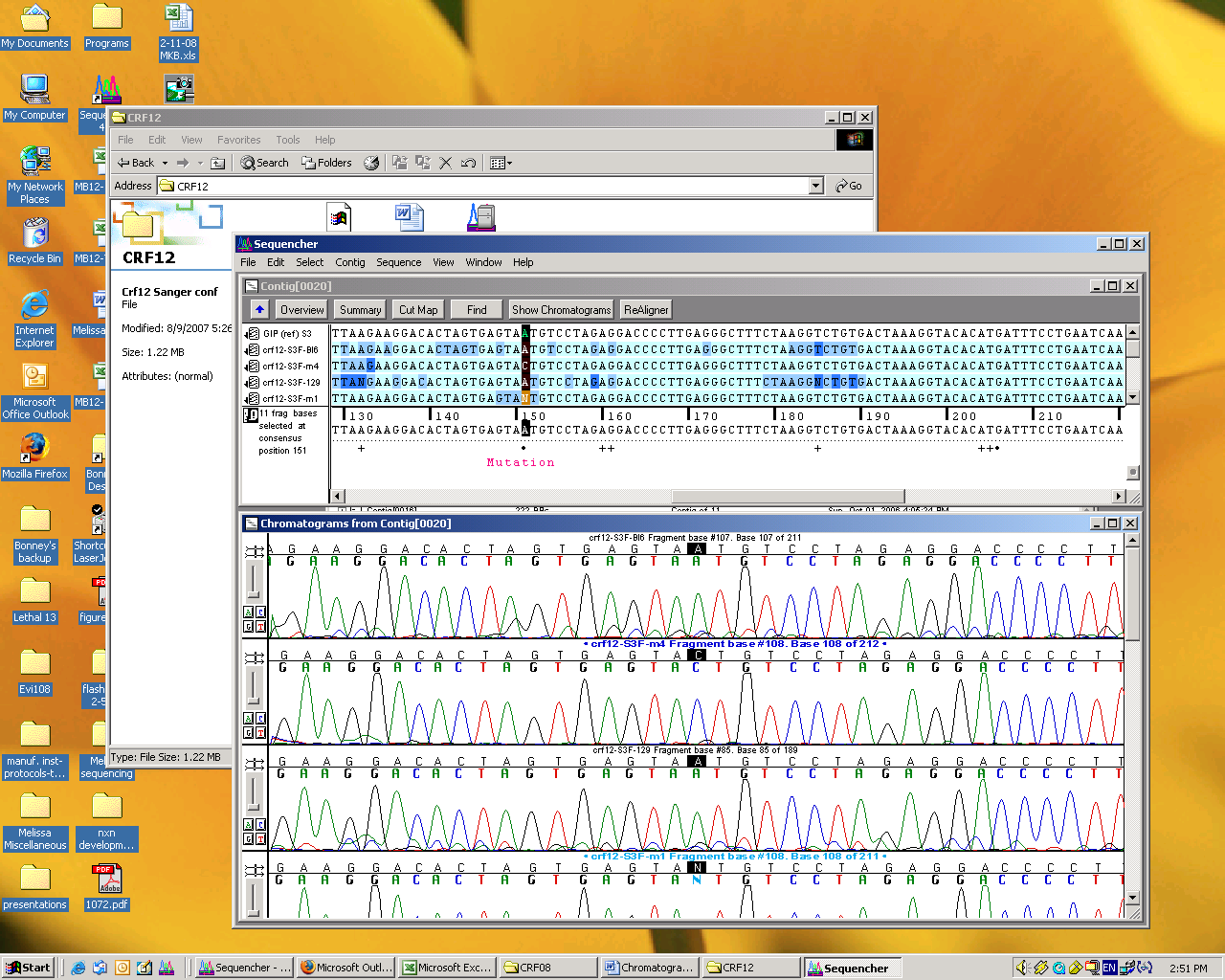
**

**Mutant Line:** *crf12*

**Gene Name:** *Gip*

**Chr. 11 base #:** 95850000

**Base Change:** A to C^F^

**
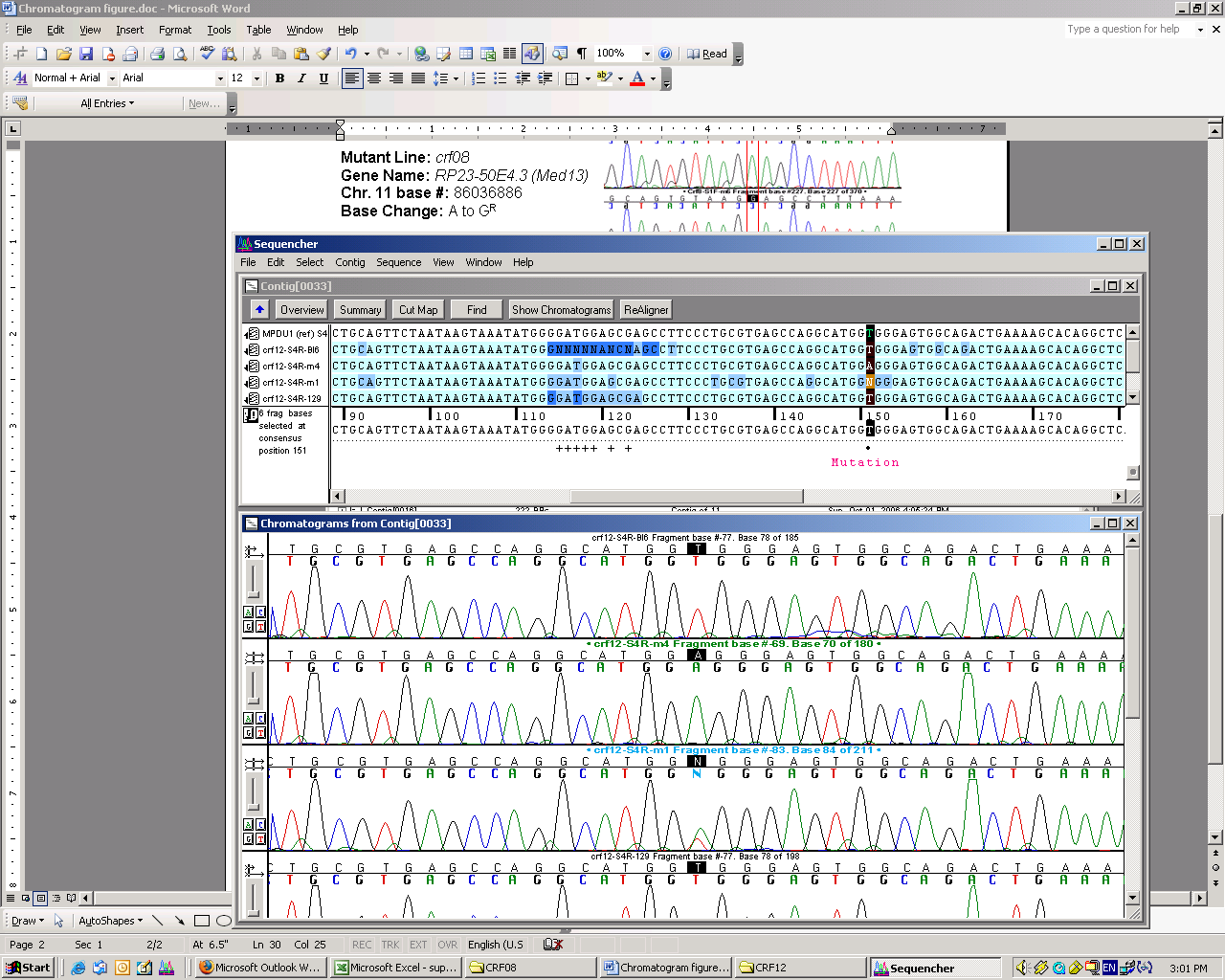
**

**Mutant Line:** *crf12*

**Gene Name:** *Mpdu1*

**Chr. 11 base #:** 69383797

**Base Change:** T to A^R^

**
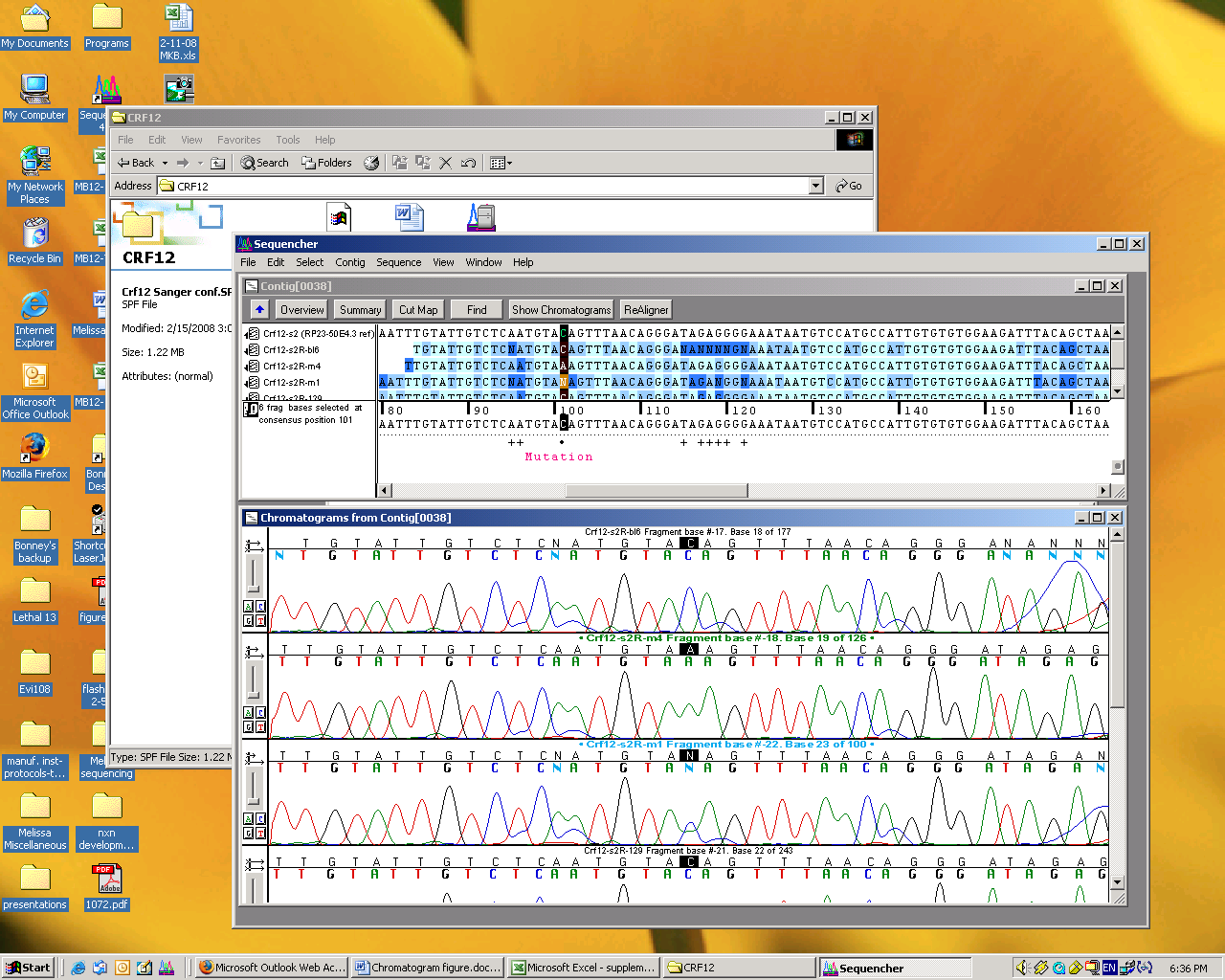
**

**Mutant Line:** *crf12*

**Gene Name:** *Med13*

**Chr. 11 base #:** 85998337

**Base Change:** C to A^R^


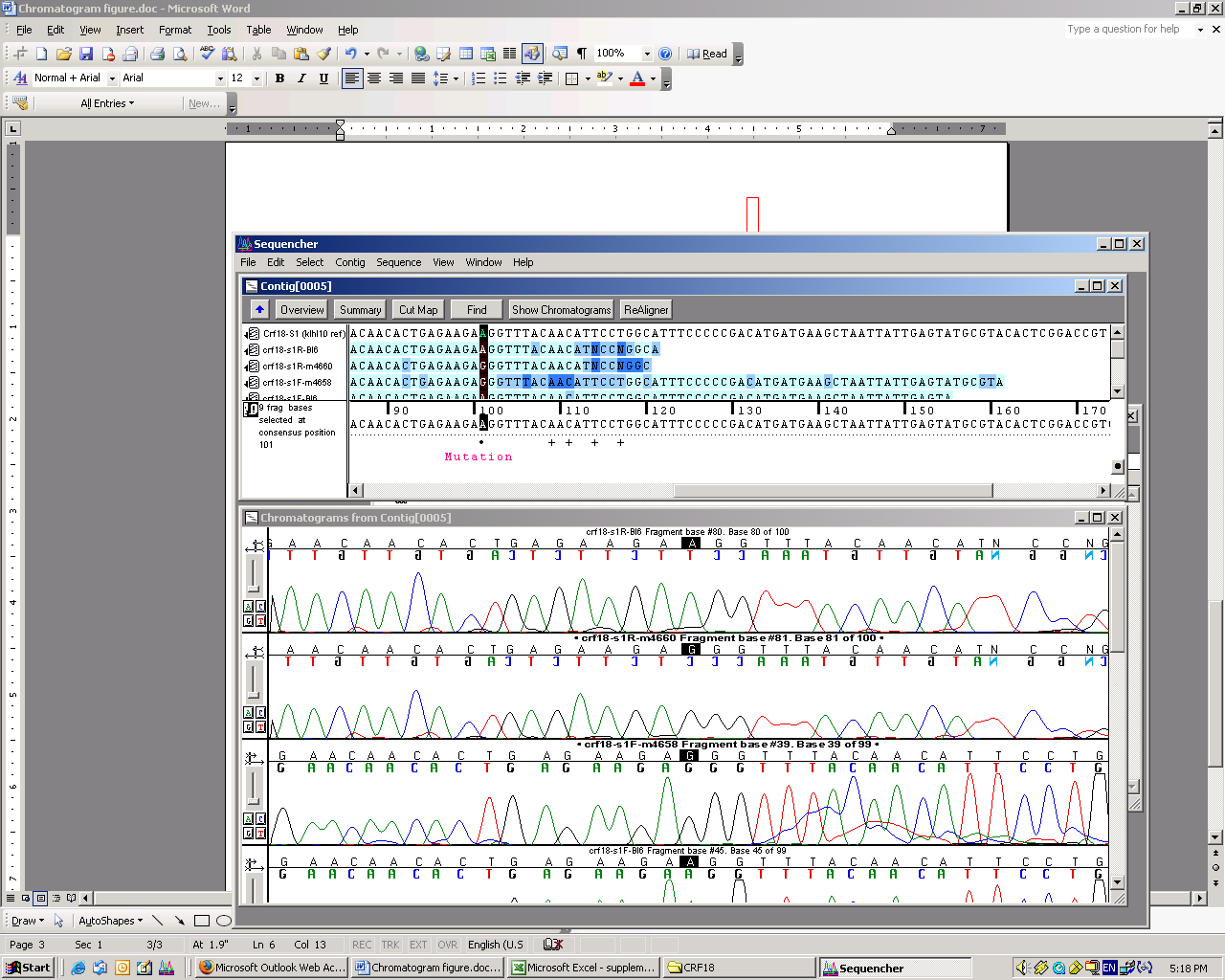
**Mutant Line:** *crf18*

**Gene Name:** *Klhl10*

**Chr. 11 base #:** 100266509

**Base Change:** A to G^F^

**
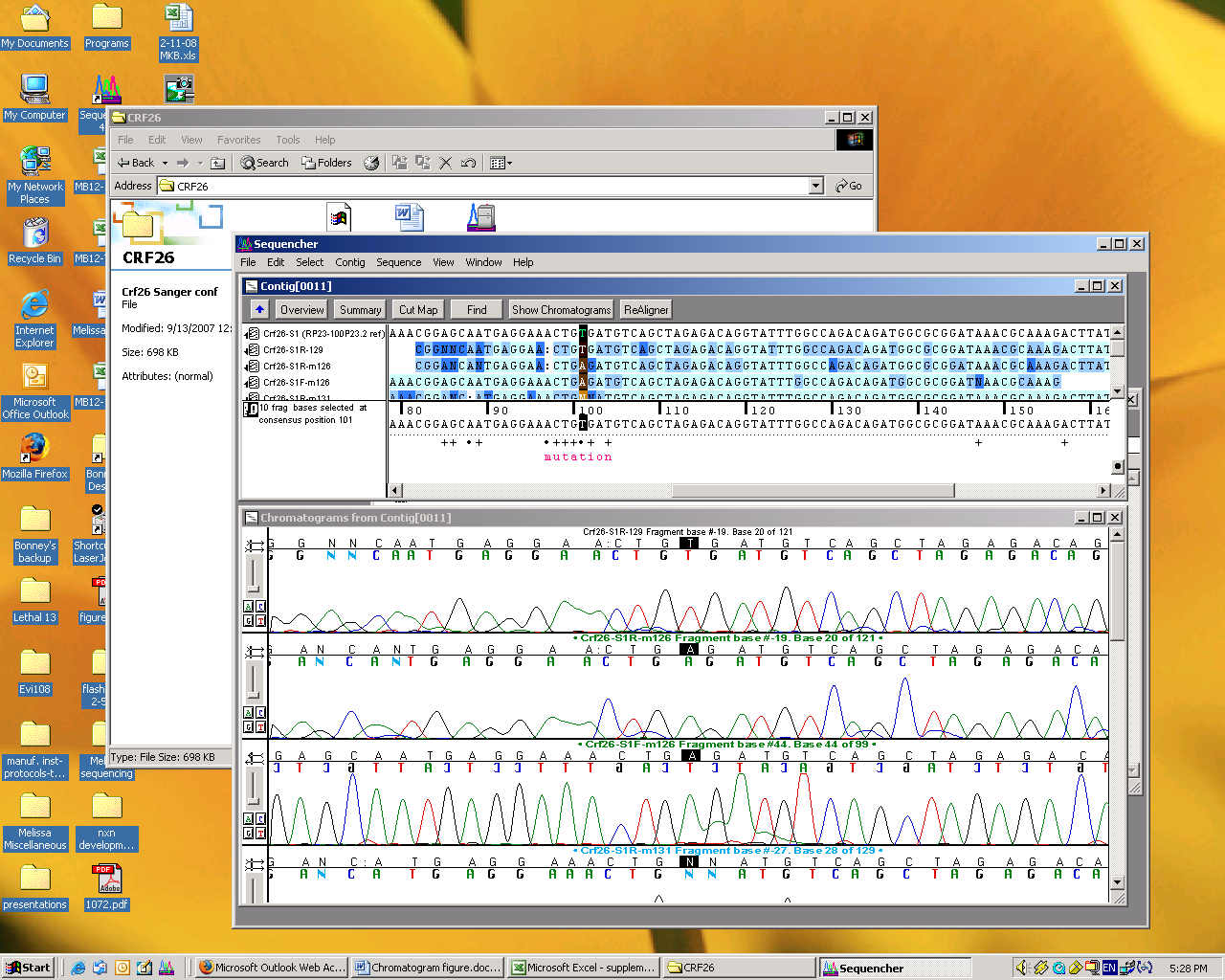
**

**Mutant Line:** *crf26*

**Gene Name:** *Ccdc55*

**Chr. 11 base #:** 76771482

**Base Change:** T to A^R^

**
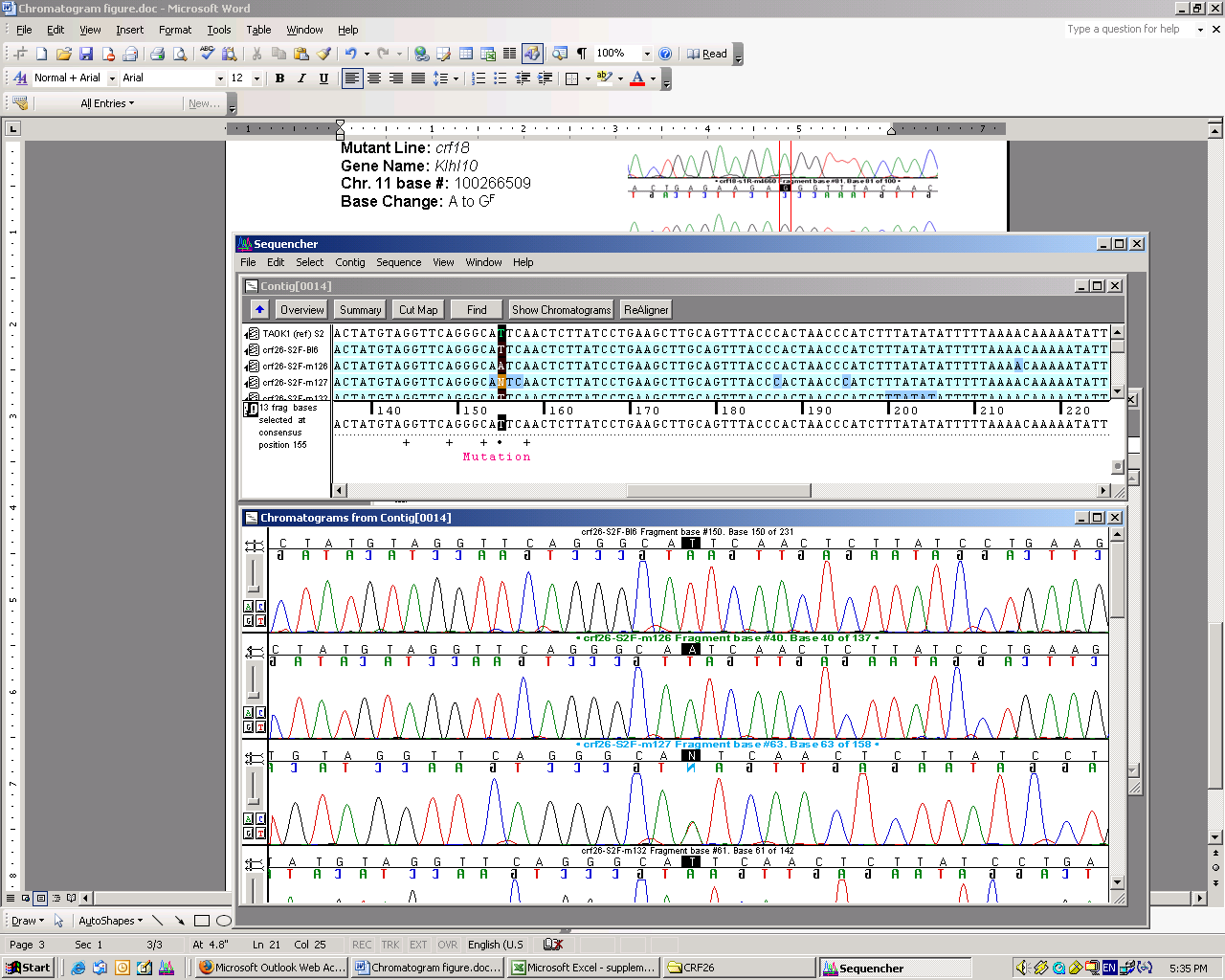
**

**Mutant Line:** *crf26*

**Gene Name:** *Taok1*

**Chr. 11 base #:** 77301436

**Base Change:** T to A^R^

**
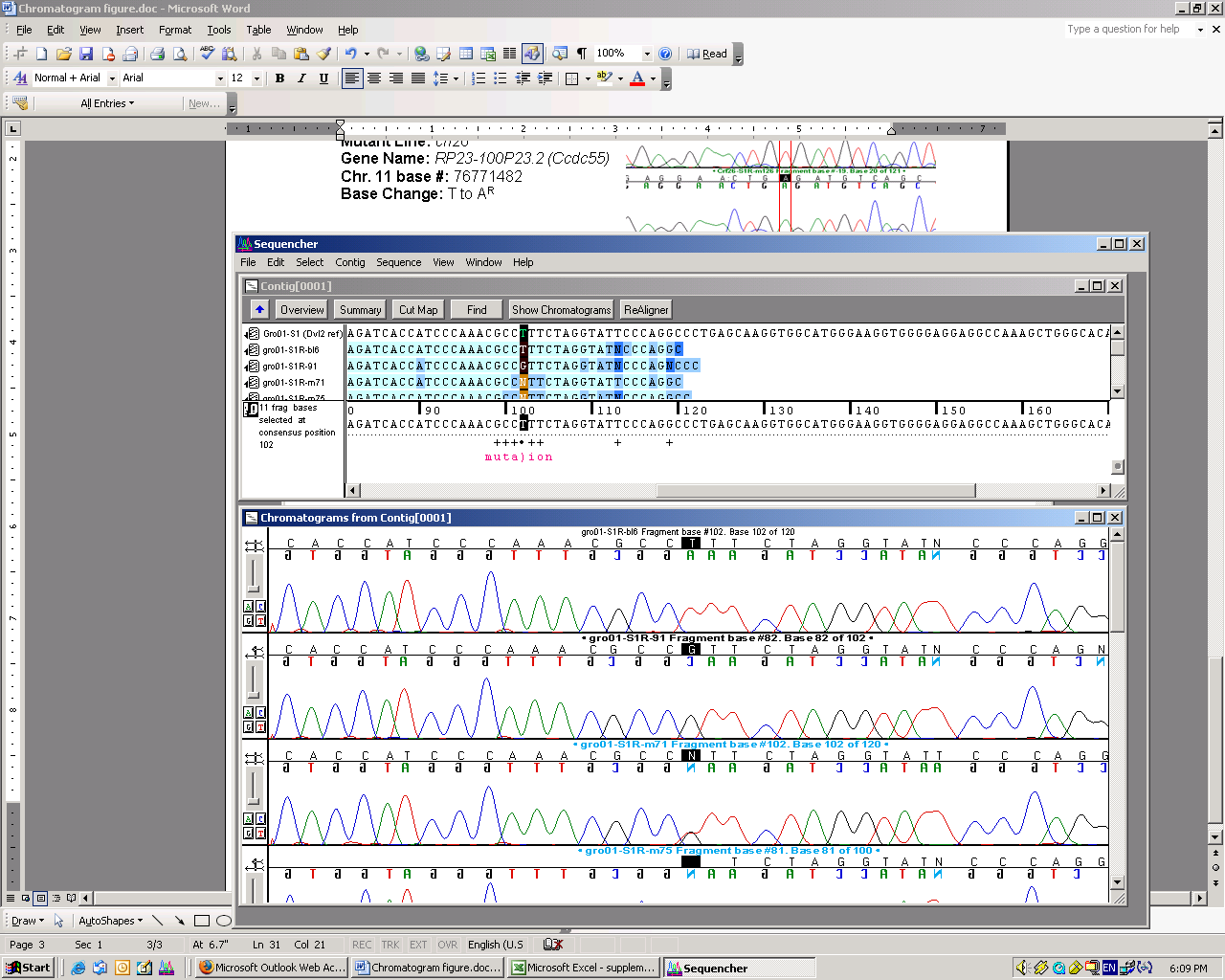
**

**Mutant Line:** *gro01*

**Gene Name:** *Dvl2*

**Chr. 11 base #:** 69733804

**Base Change:** T to G^F^

**
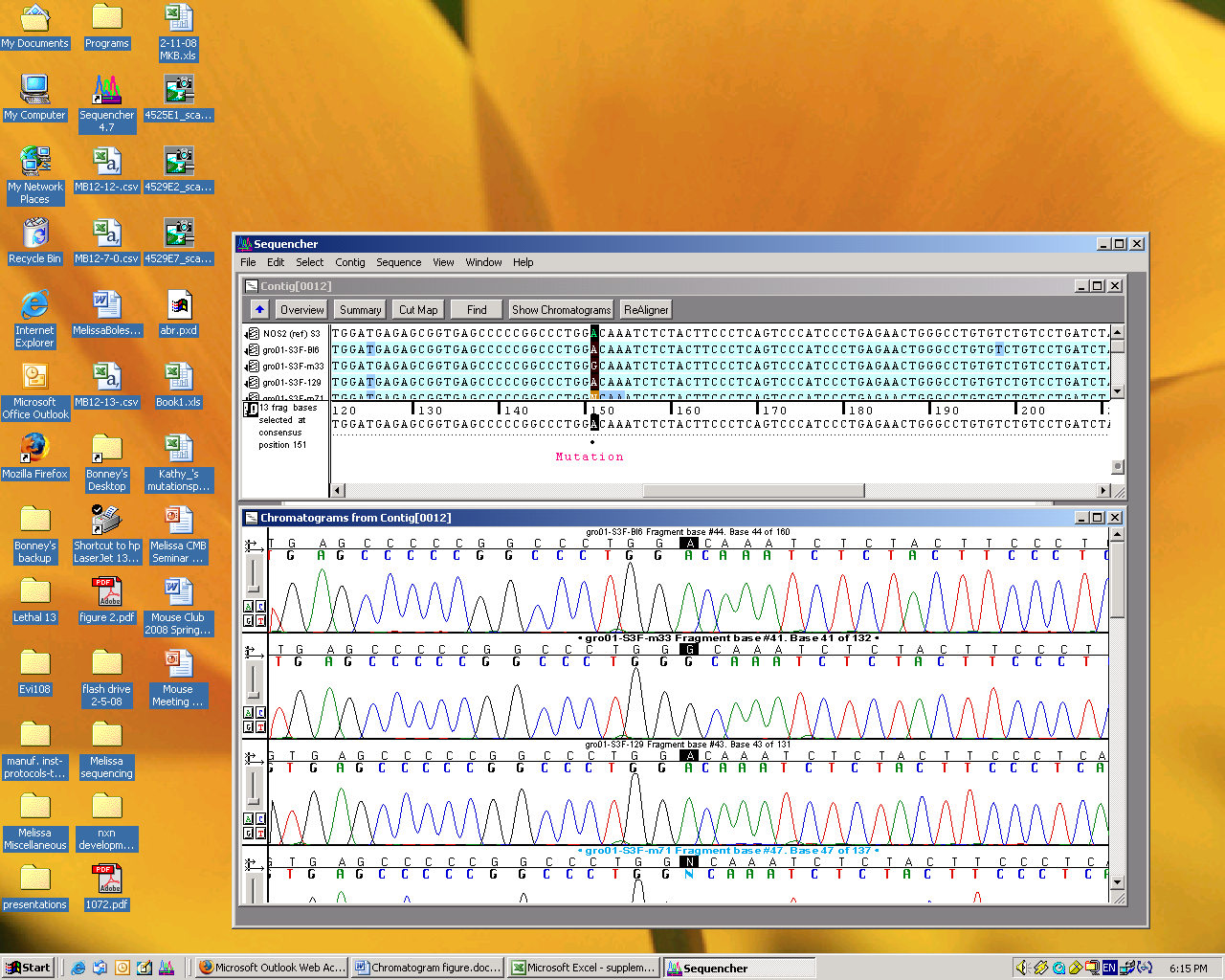
**

**Mutant Line:** *gro01*

**Gene Name:** *Nos2*

**Chr. 11 base #:** 78675816

**Base Change:** A to G^F^

**
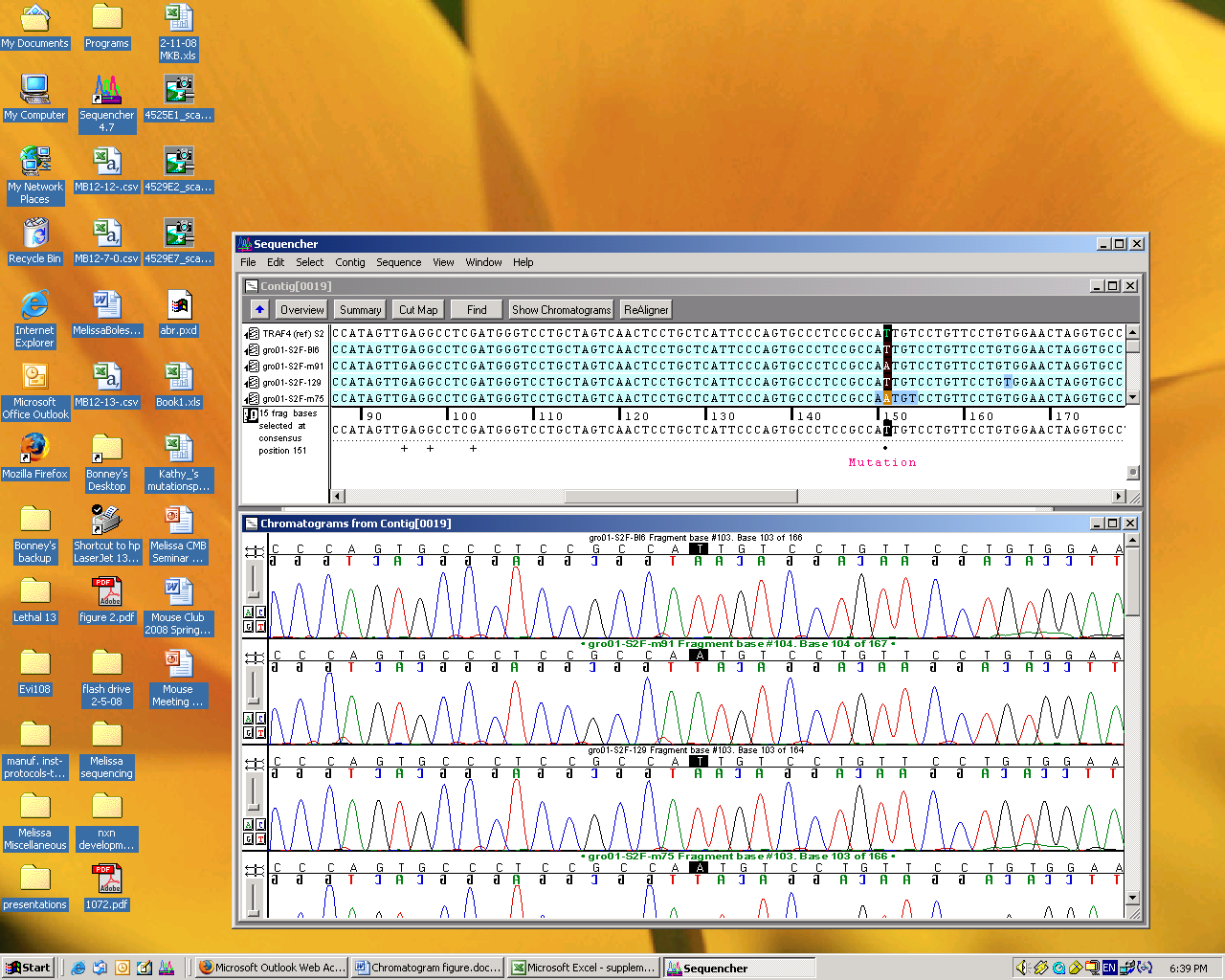
**

**Mutant Line:** *gro01*

**Gene Name:** *Traf4*

**Chr. 11 base #:** 77886324

**Base Change:** T to A^R^

**
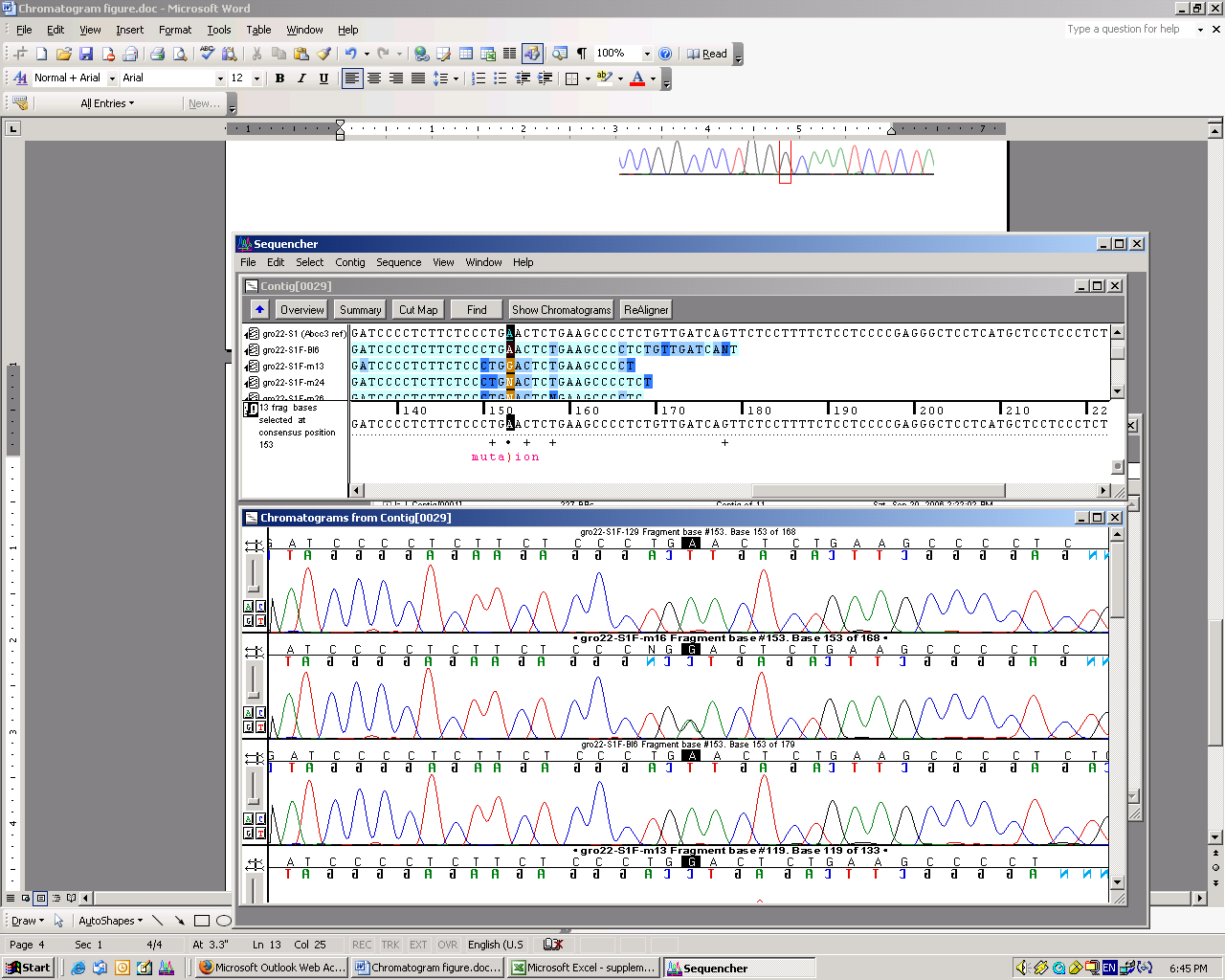
**

**Mutant Line:** *gro22*

**Gene Name:** *Abcc3*

**Chr. 11 base #:** 94185363

**Base Change:** A to G^R^

**
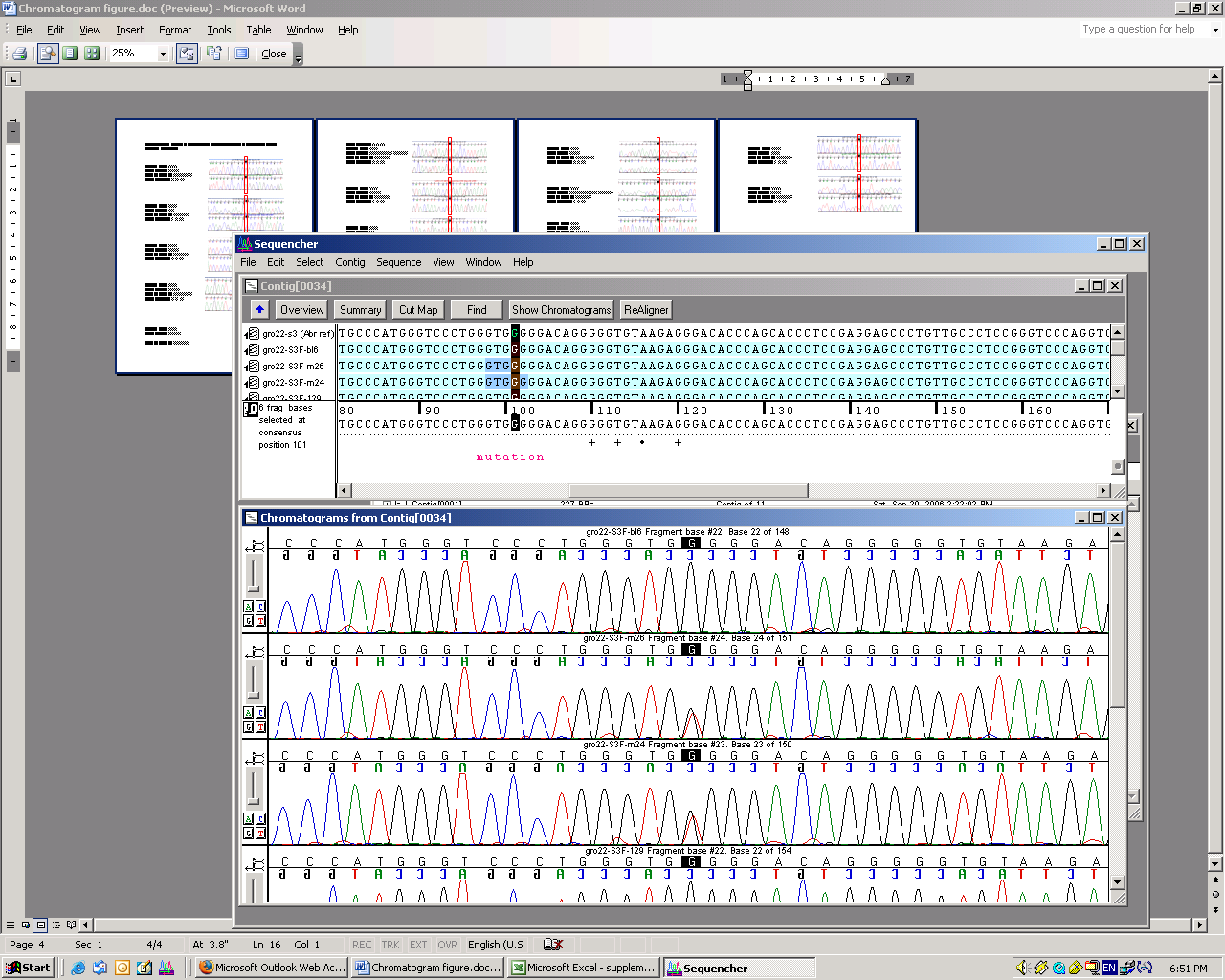
**

**Mutant Line:** *gro22*

**Gene Name:** *Abr*

**Chr. 11 base #:** 76296662

**Base Change:** G to T^R^

**
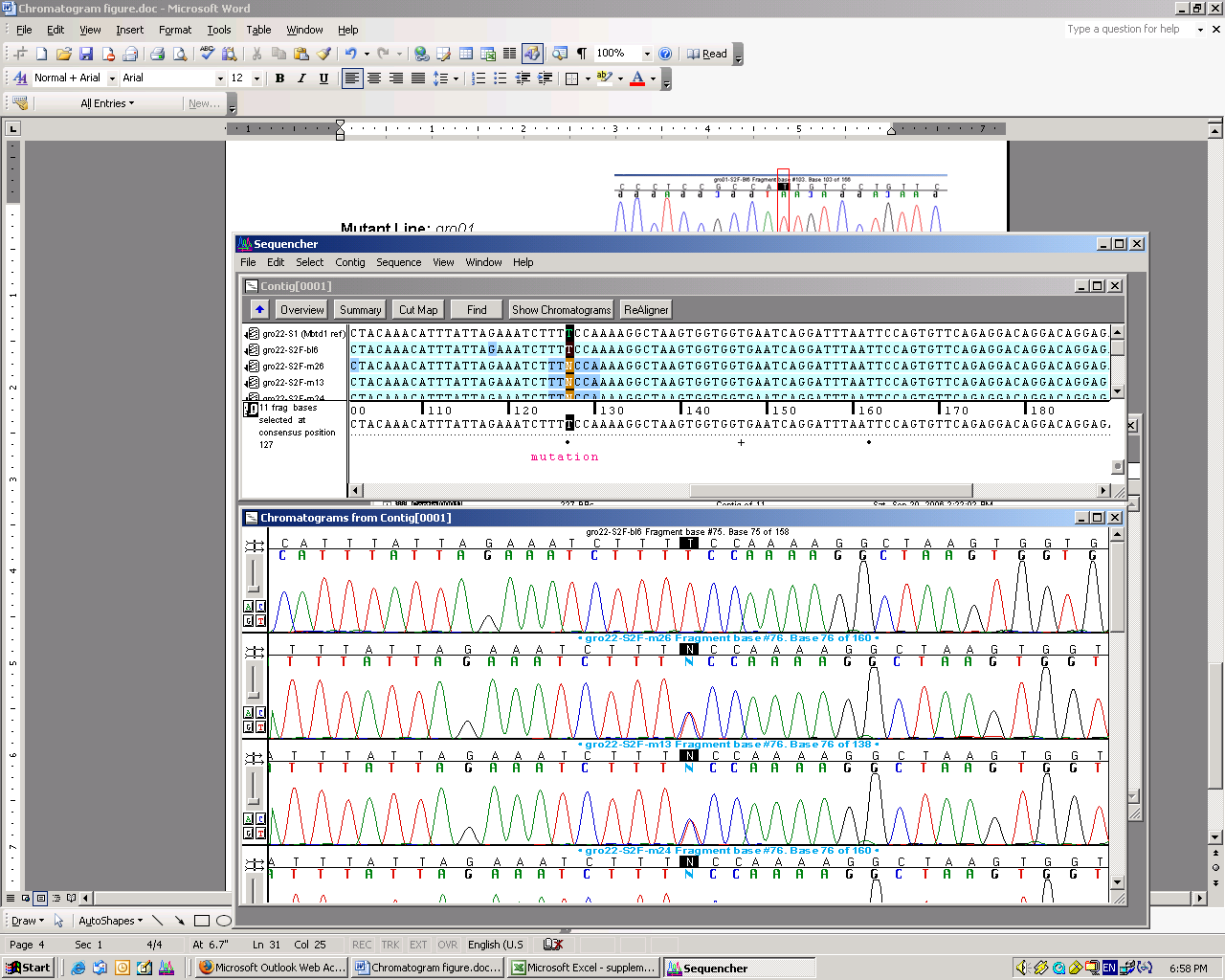
**

**Mutant Line:** *gro22*

**Gene Name:** *Mbtd1*

**Chr. 11 base #:** 93758035

**Base Change:** T to C^F^

**
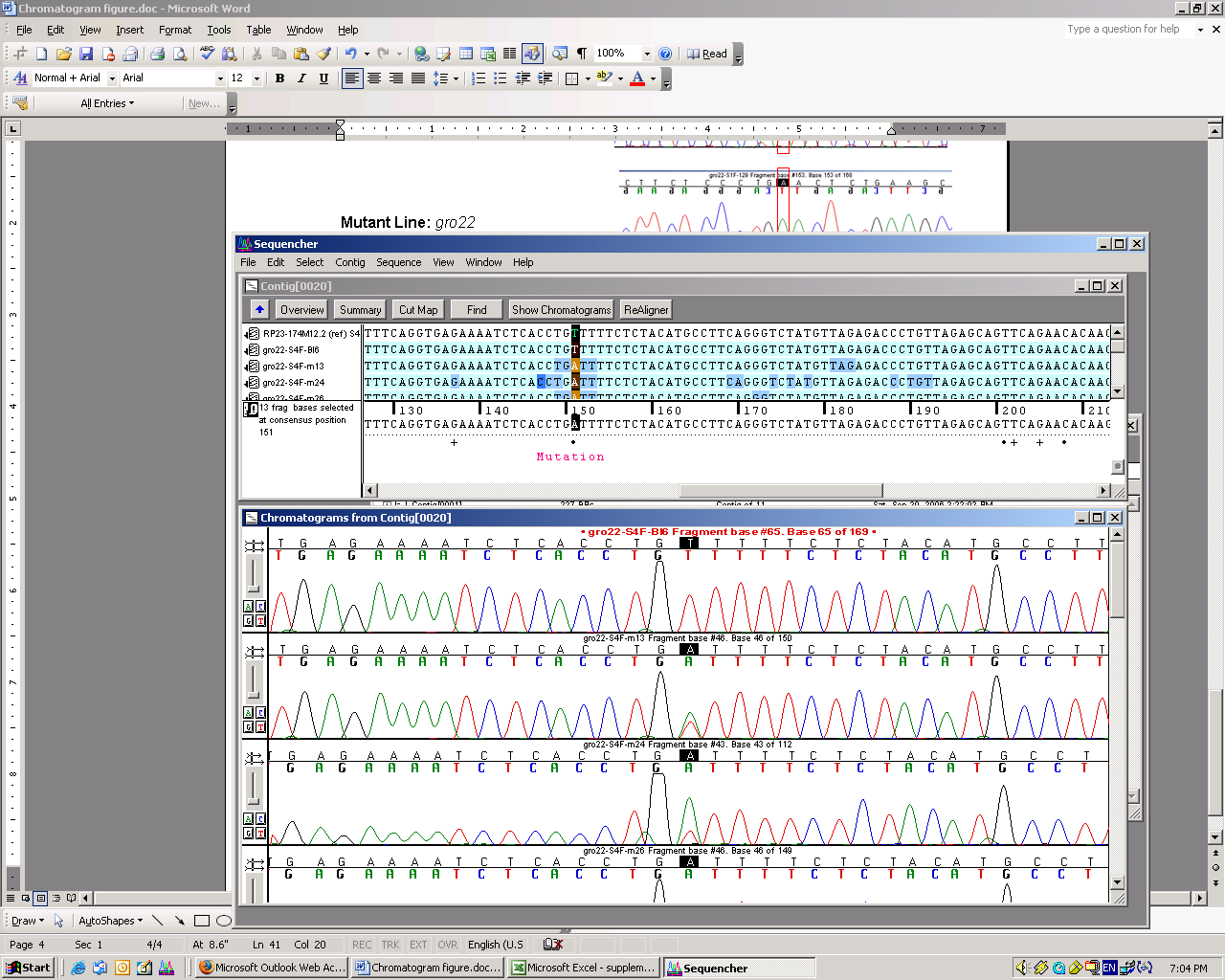
**

**Mutant Line:** *gro22*

**Gene Name:** *Mett10d*

**Chr. 11 base #:** 74528690

**Base Change:** T to A^F^


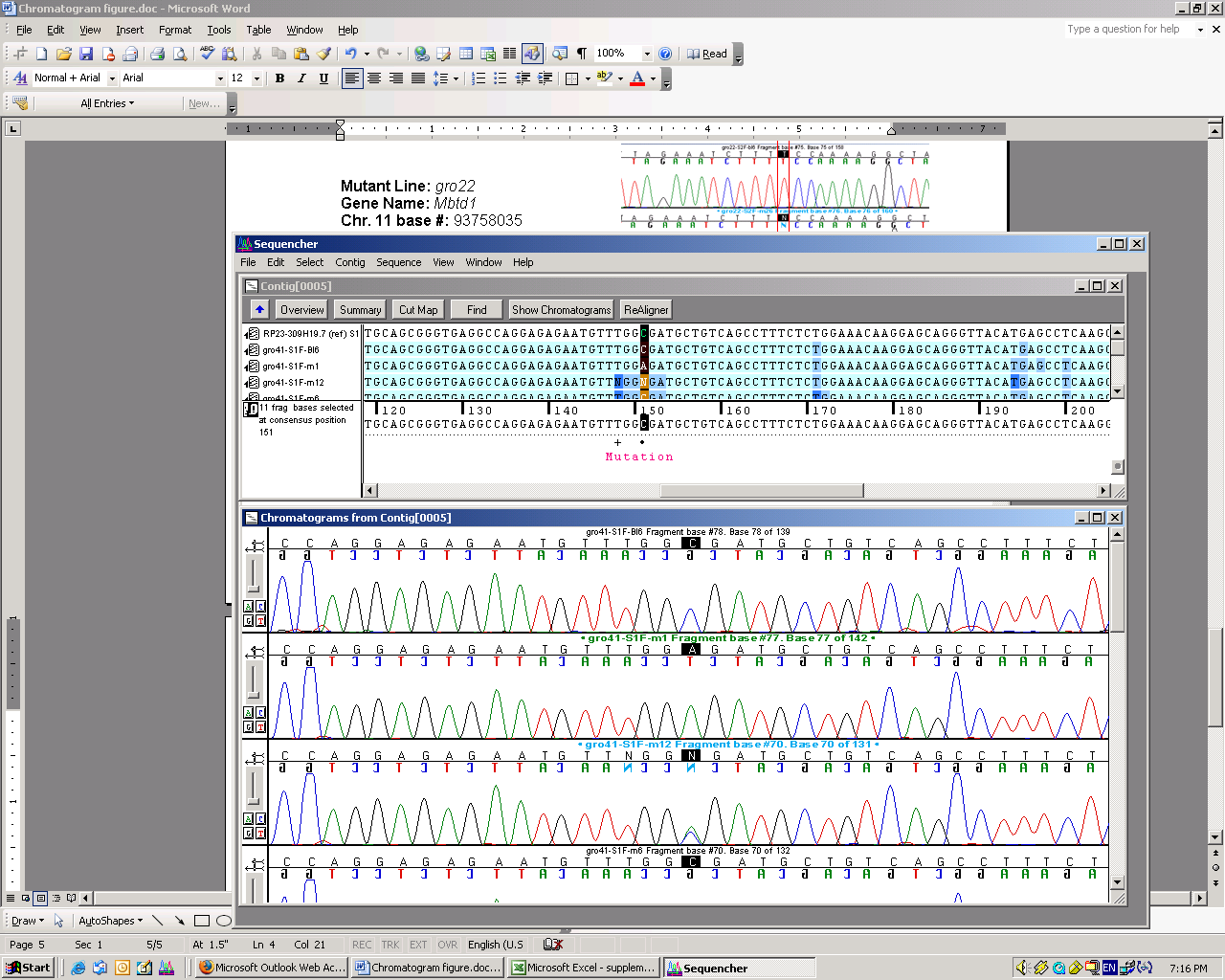
**Mutant Line:** *gro41*

**Gene Name:** *Stac2*

**Chr. 11 base #:** 97860769

**Base Change:** C to A^R^

**
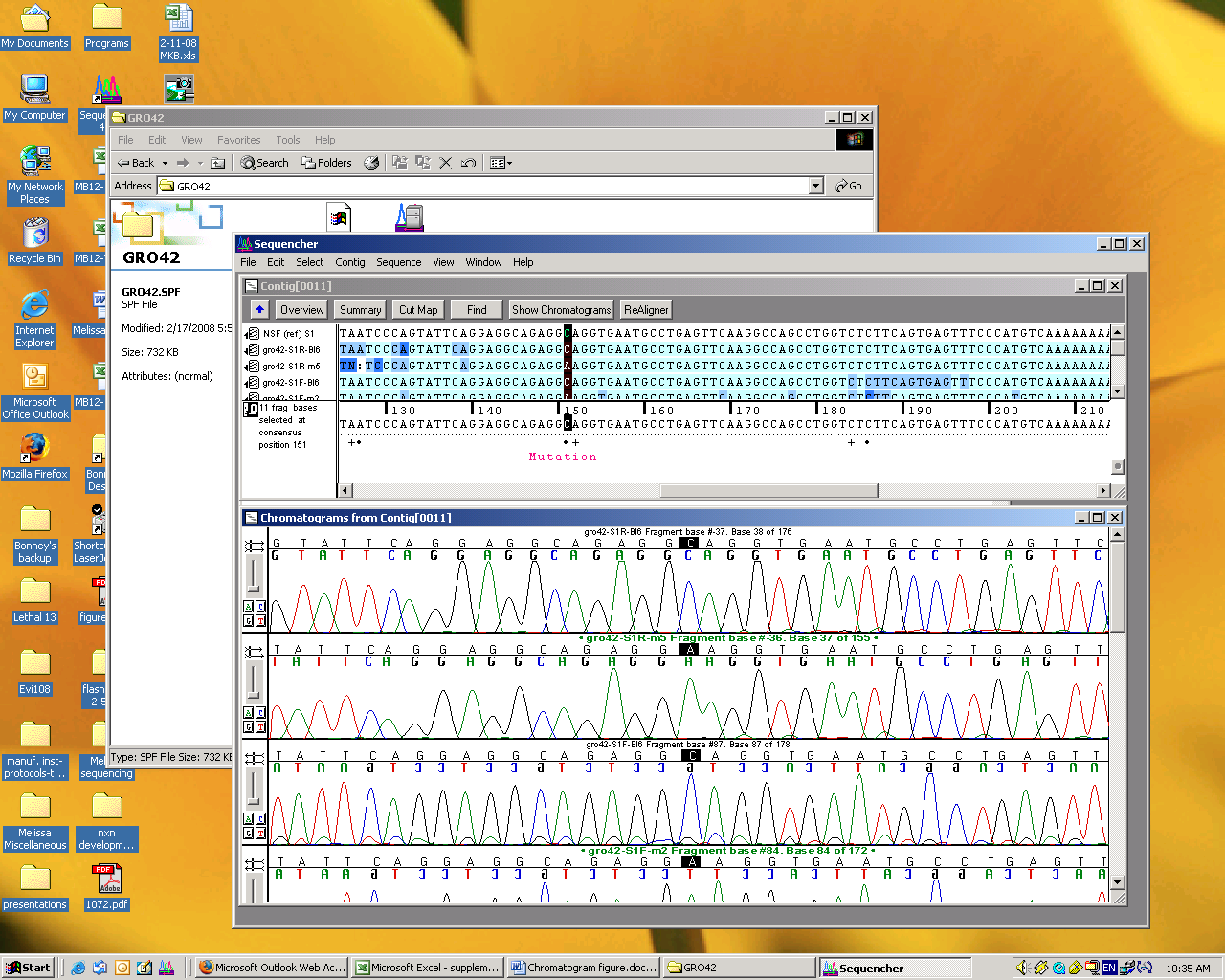
**

**Mutant Line:** *gro42*

**Gene Name:** *Nsf*

**Chr. 11 base #:** 103669305

**Base Change:** C to A^R^

**
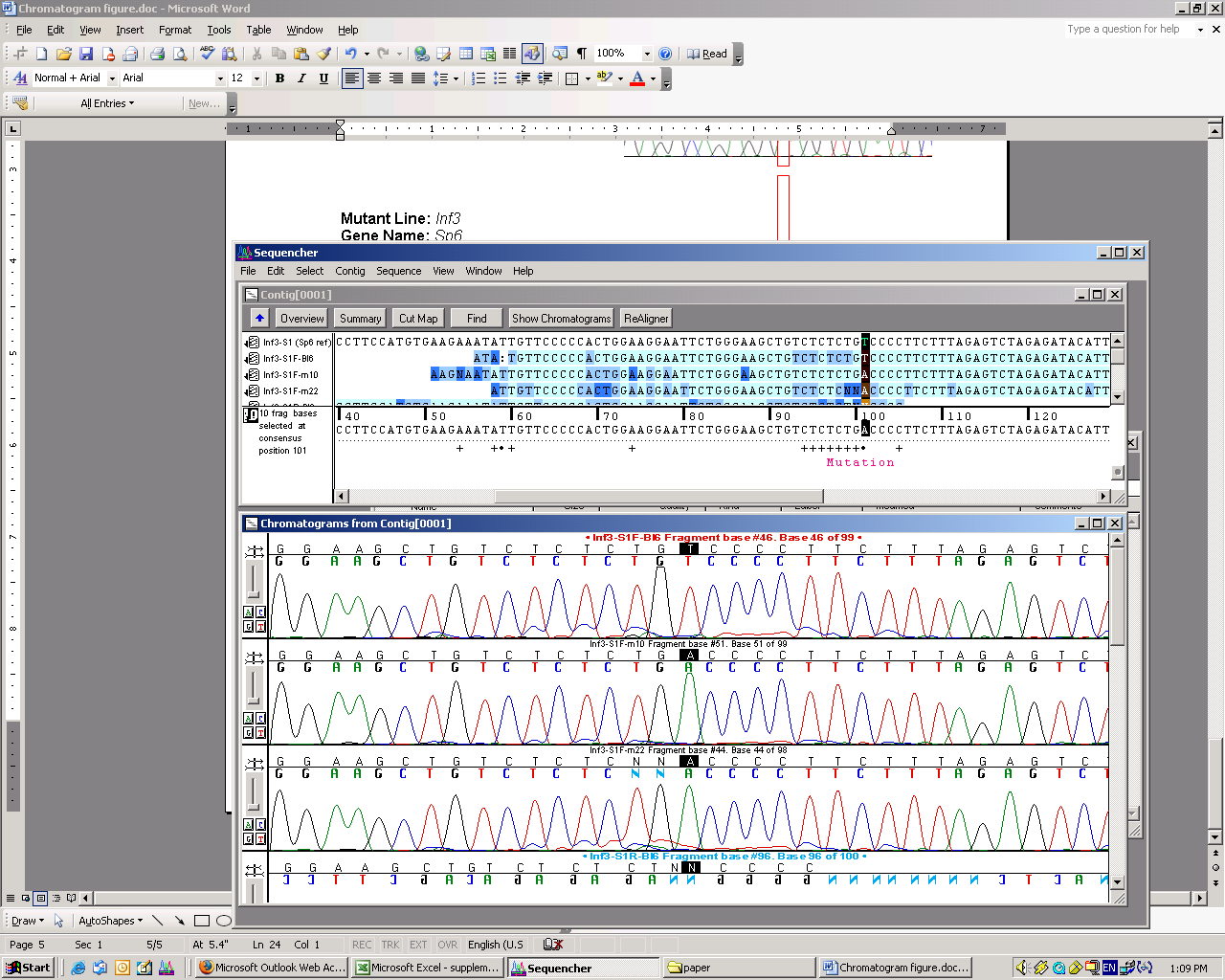
**

**Mutant Line:** *Inf3*

**Gene Name:** *Sp6*

**Chr. 11 base #:** 96844781

**Base Change:** T to A^F^

**
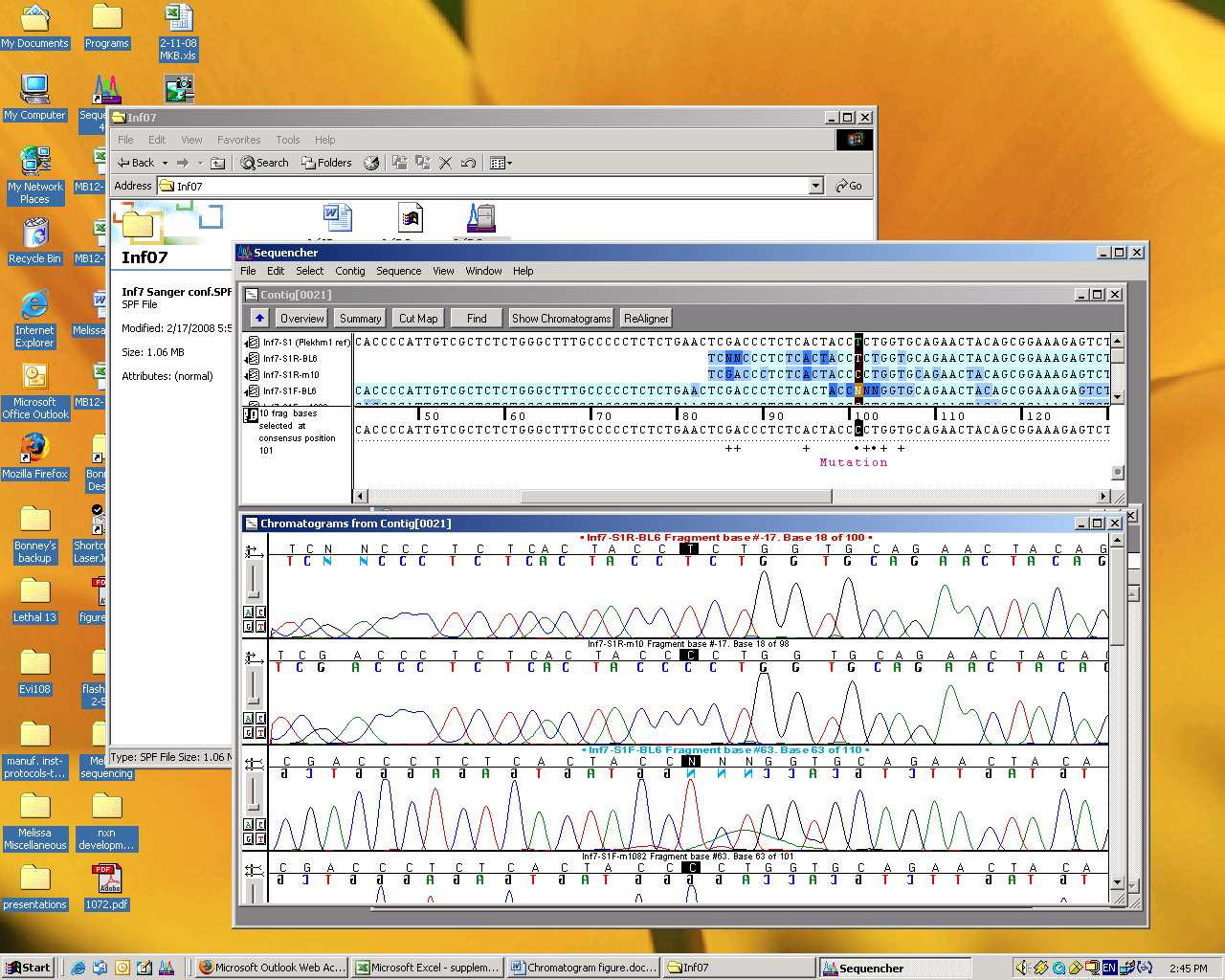
**

**Mutant Line:** *Inf7*

**Gene Name:** *Plekhm1*

**Chr. 11 base #:** 103216073

**Base Change:** T to C^R^


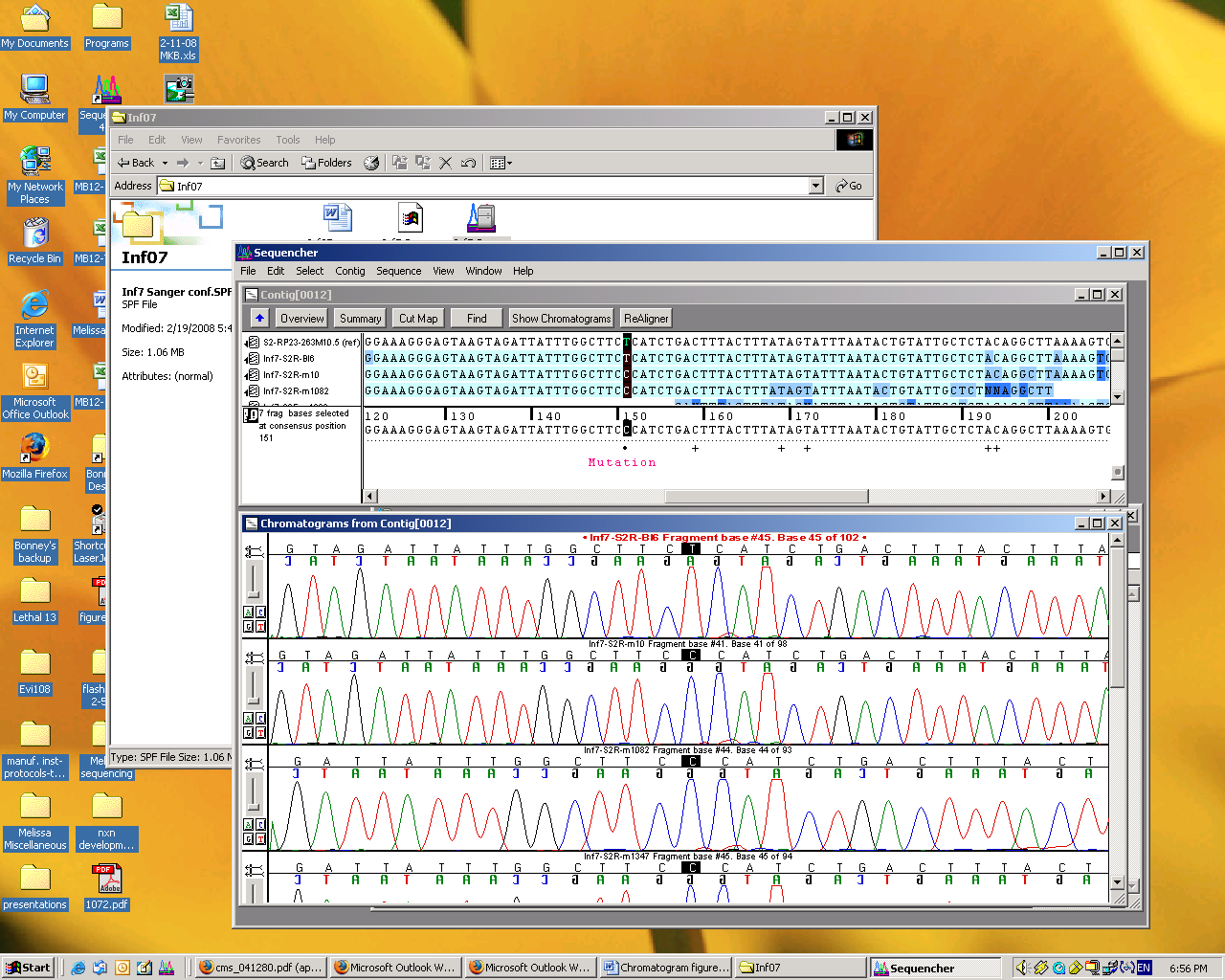
**Mutant Line:** *Inf7*

**Gene Name:** *RP23-263M10.5*

**Chr. 11 base #:** 72806018

**Base Change:** T to C^F^

**
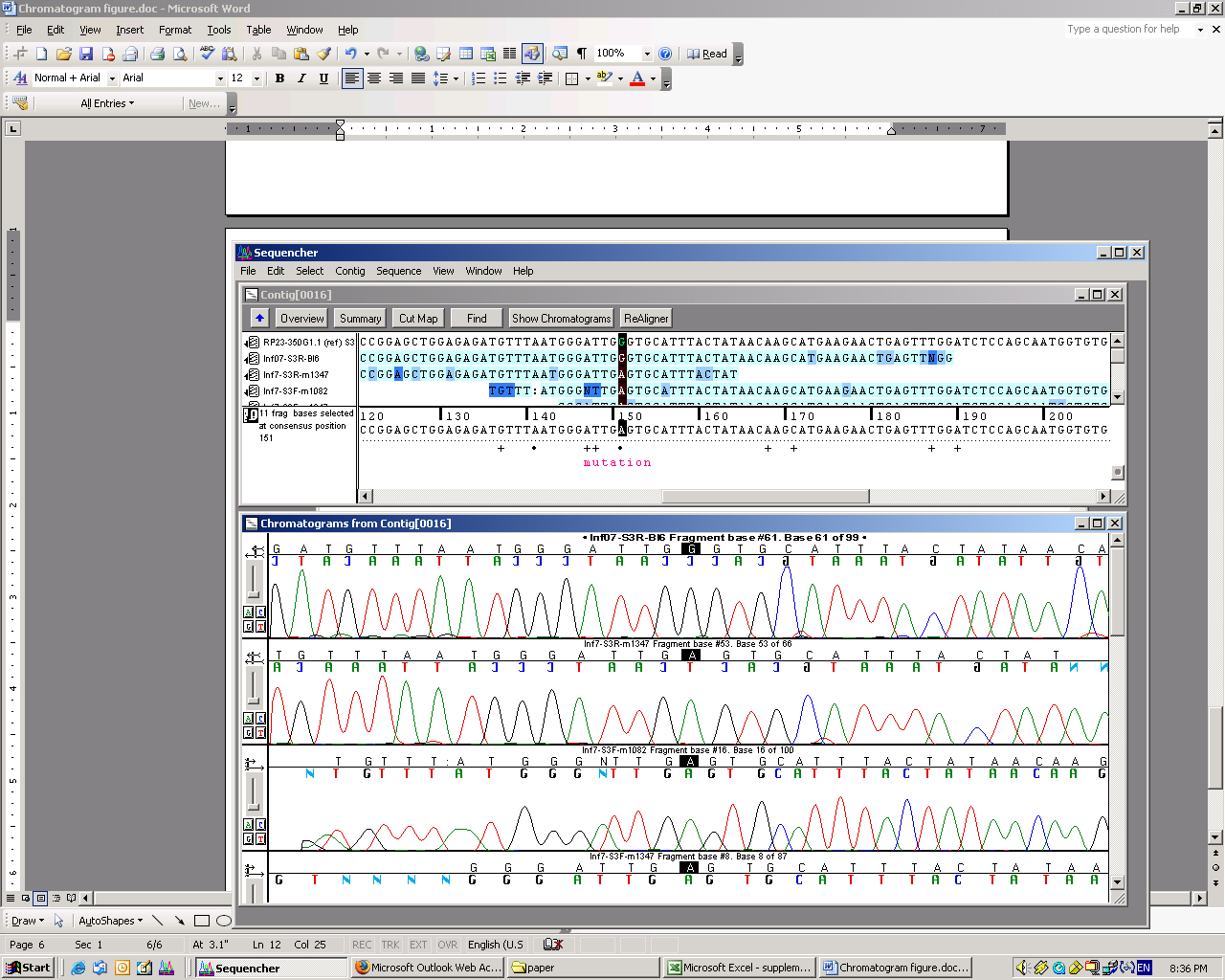
**

**Mutant Line:** *Inf7*

**Gene Name:** *RP23-350G1.1*

**Chr. 11 base #:** 81603955

**Base Change:** G to A

**
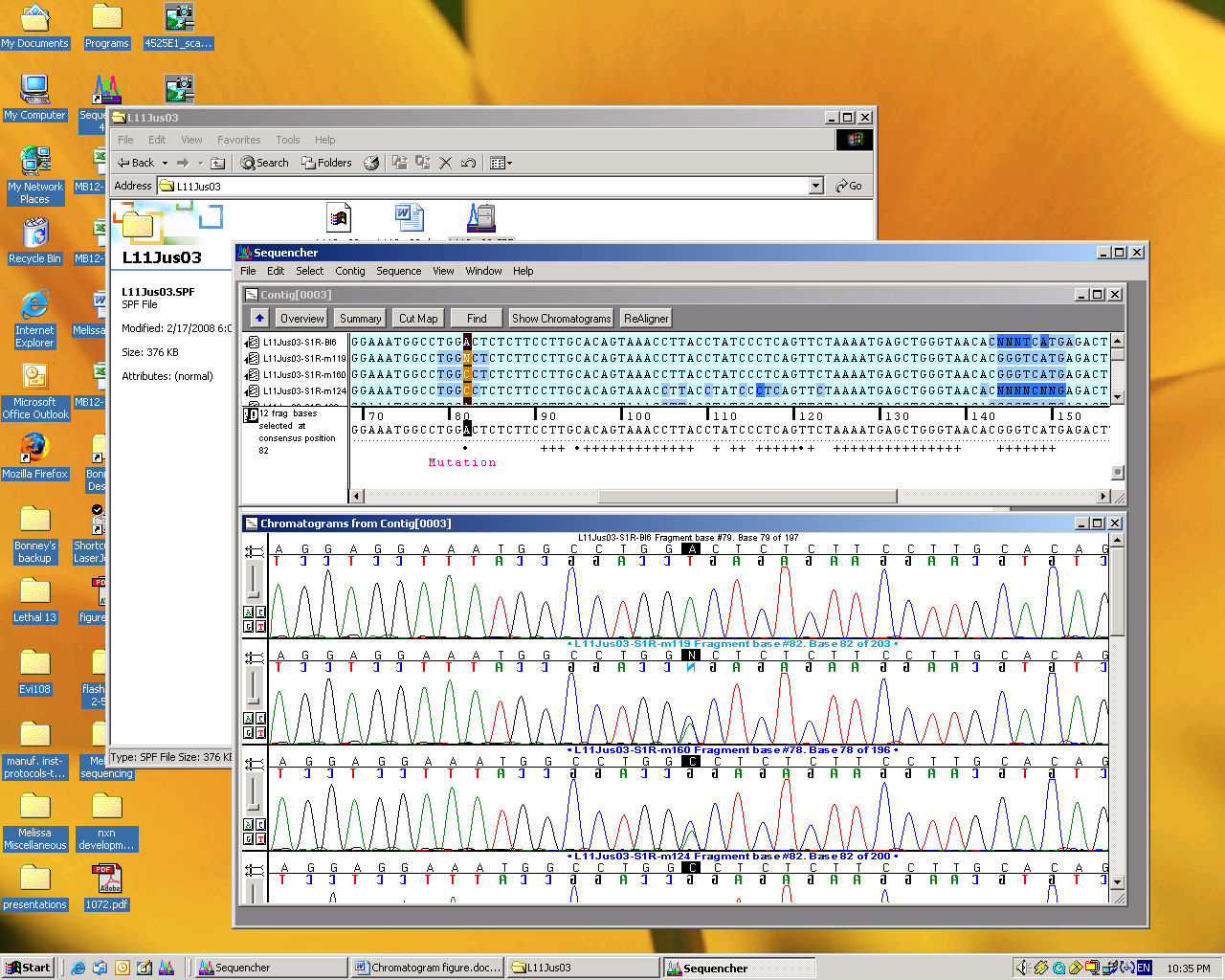
**

**Mutant Line:** *l11Jus03*

**Gene Name:** *Git1*

**Chr. 11 base #:** 77228888

**Base Change:** A to C^F^

**
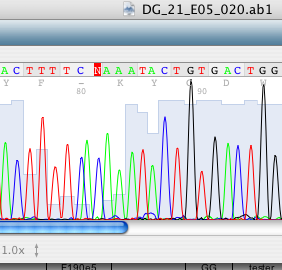
**

**Mutant Line:** *l11Jus05****Error: Reference source not found***


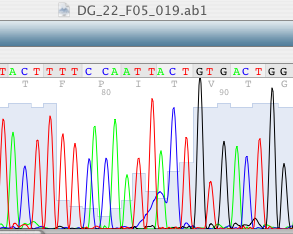
**Gene Name:** *Med13*

**Chr. 11 base #:** 85998715

**Base Change:** T to A^R^

**
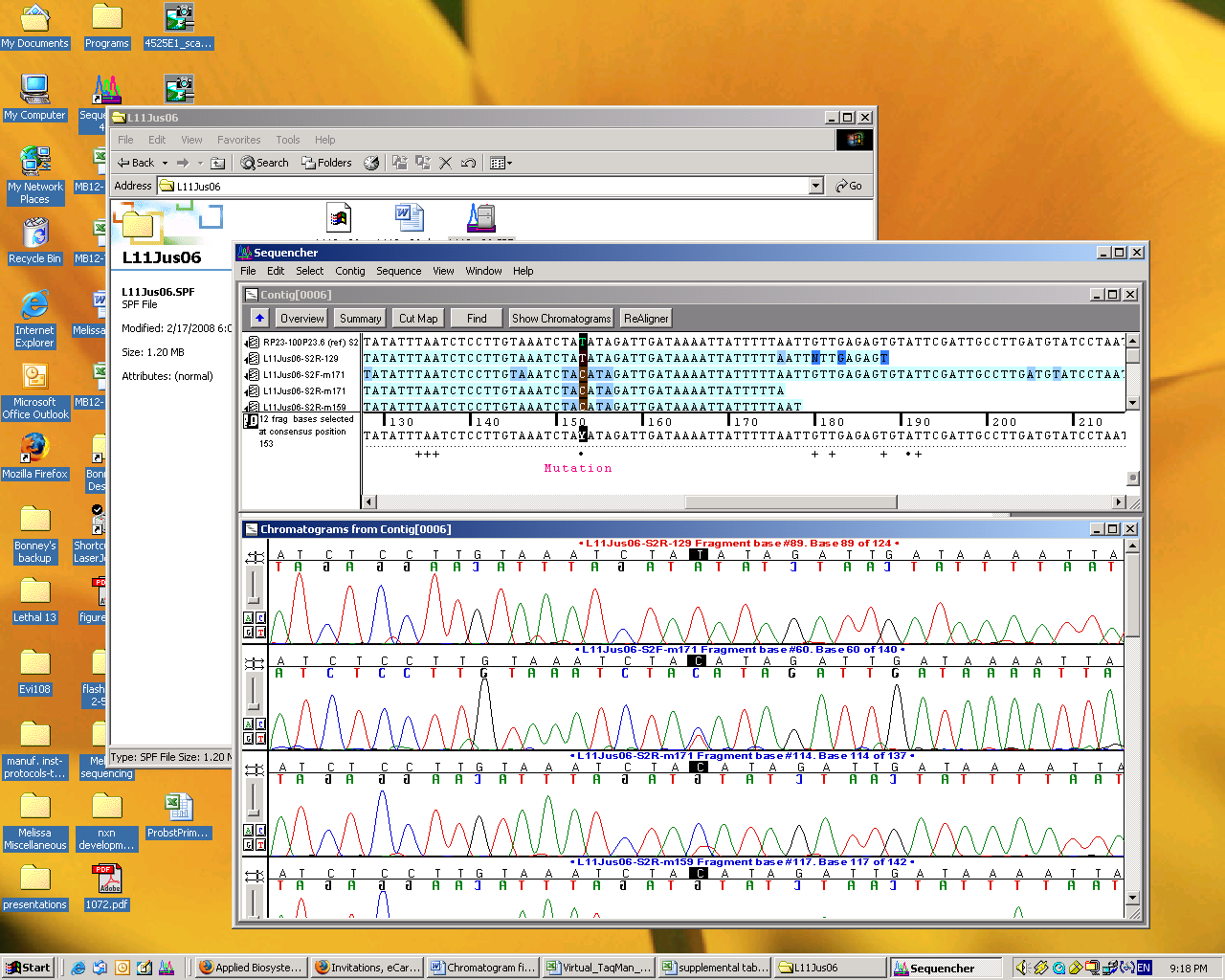
**

**Mutant Line:** *l11Jus06*

**Gene Name:** *Tmigd1*

**Chr. 11 base #:** 76632430

**Base Change:** T to C^F^

**
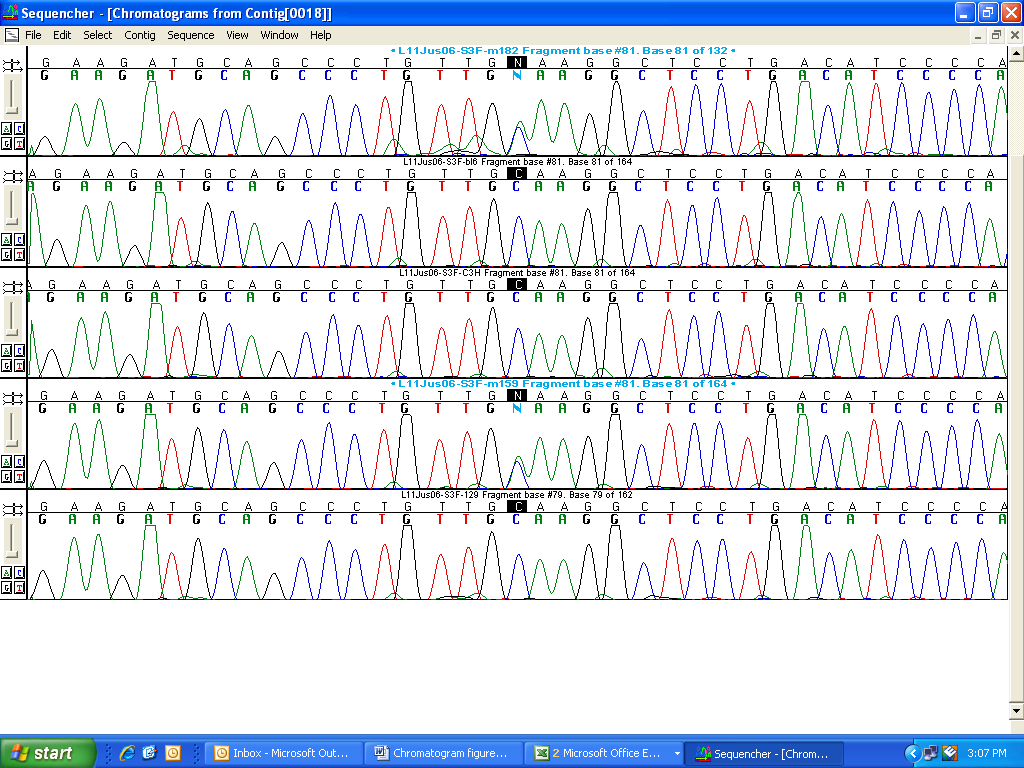
**

**Mutant Line:** *l11Jus06*

**Gene Name:** *RP23-185A18.9*

**Chr. 11 base #:** 77995272

**Base Change:** C to A^F^

**
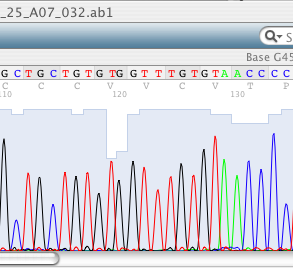
**

**Mutant Line:** *l11Jus08*

**Gene Name:** *Zzef1*


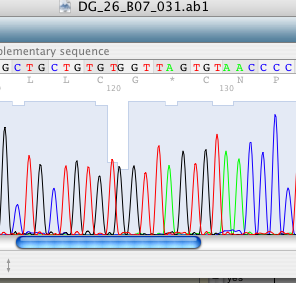
**Chr. 11 base #:** 72616638

**Base Change:** T to A^F^

**
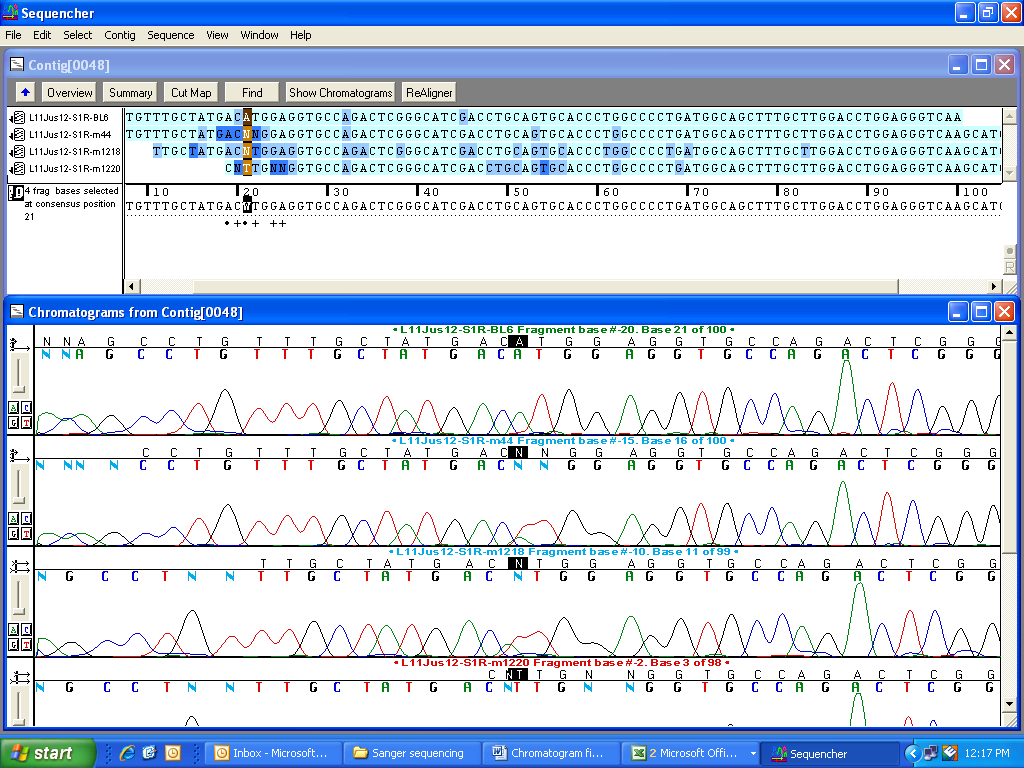
**

**Mutant Line:** *l11Jus12*

**Gene Name:** *Map3k14*

**Chr. 11 base #:** 103042222

**Base Change:** A to T^R^

**
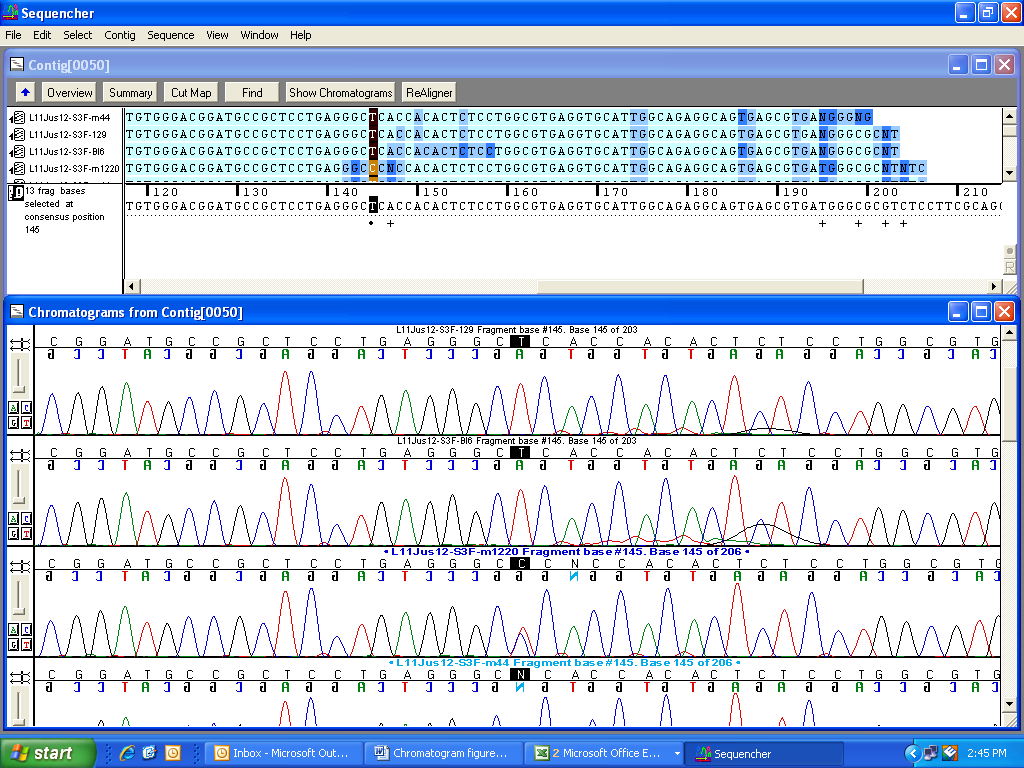
**

**Mutant Line:** *l11Jus12*

**Gene Name:** *P140*

**Chr. 11 base #:** 97357664

**Base Change:** T to C^R^

**
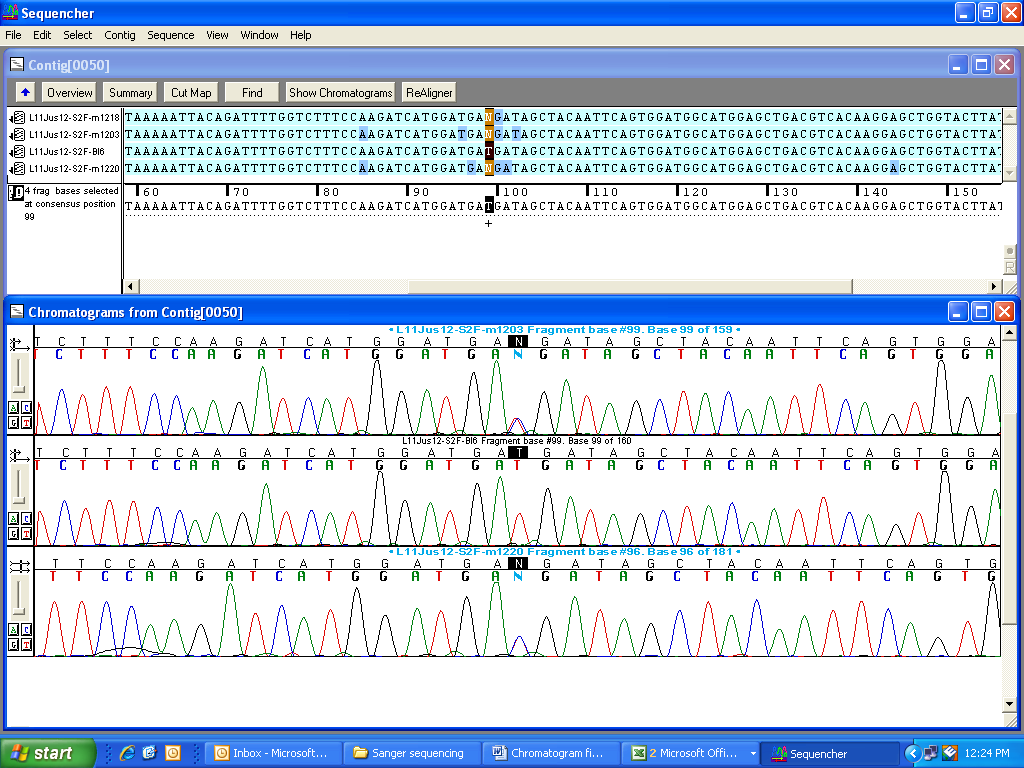
**

**Mutant Line:** *l11Jus12*

**Gene Name:** *Tlk2*

**Chr. 11 base #:** 105092268

**Base Change:** T to C^F^


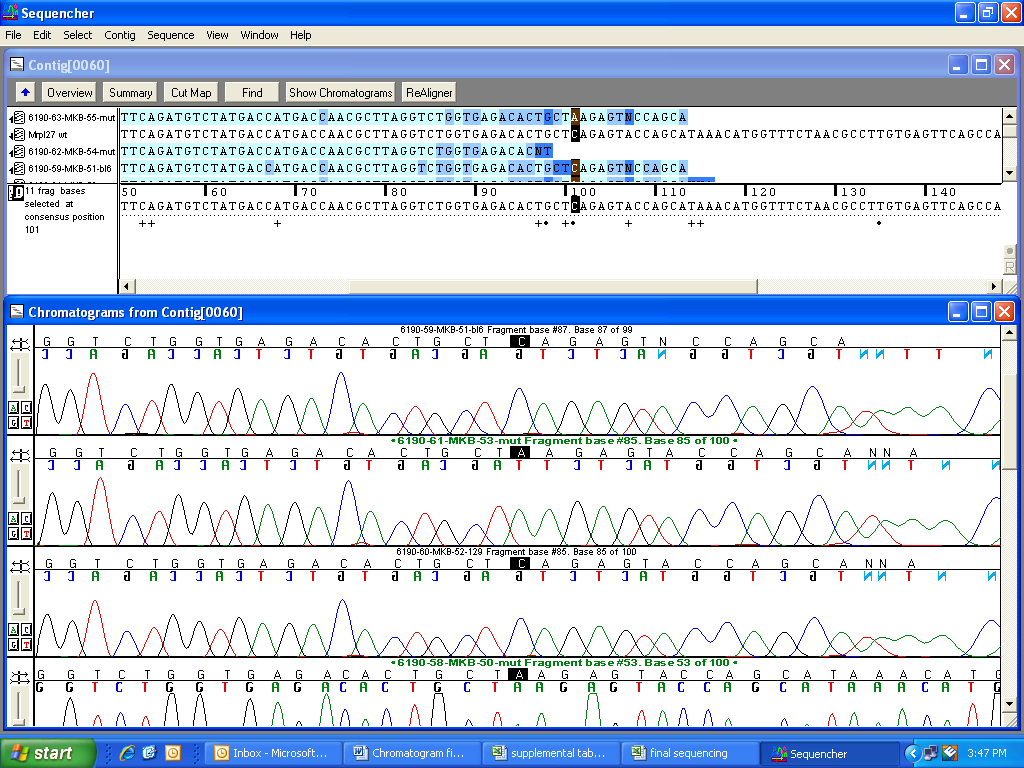


**Mutant Line:** *L11Jus13*

**Gene Name:** *Mrpl27*

**Chr. 11 base #:** 94475340

**Base Change:** C to A^F^


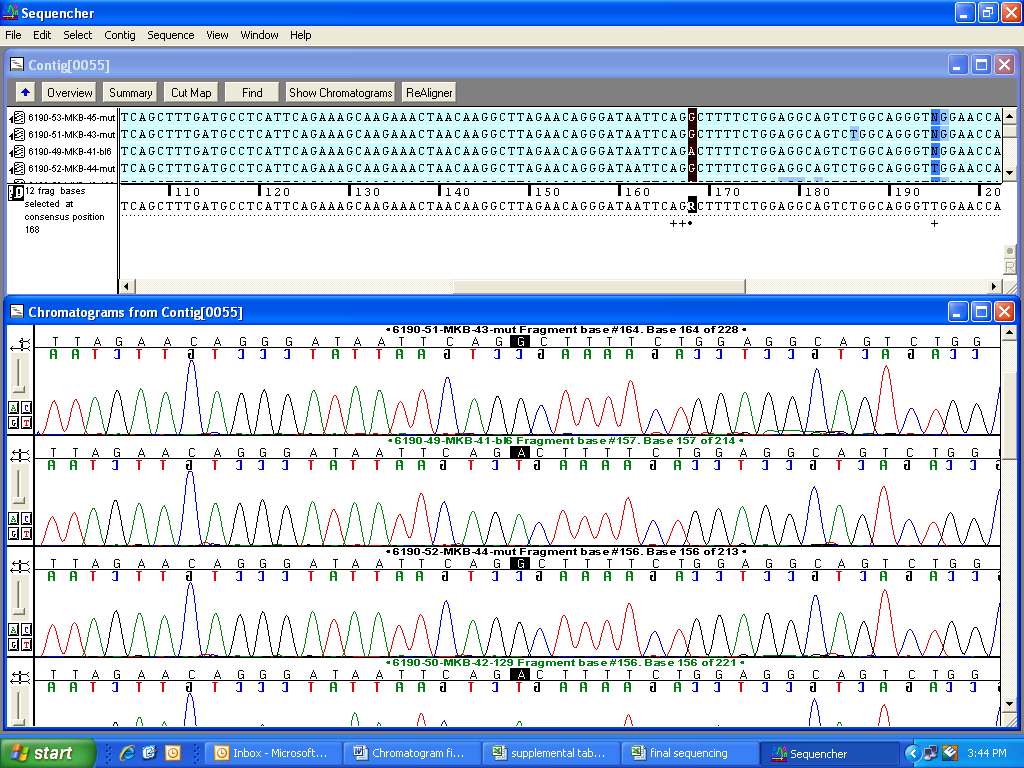


**Mutant Line:** *l11Jus13*

**Gene Name:** *Nbr1*

**Chr. 11 base #:** 101389913

**Base Change:** A to G^F^

**
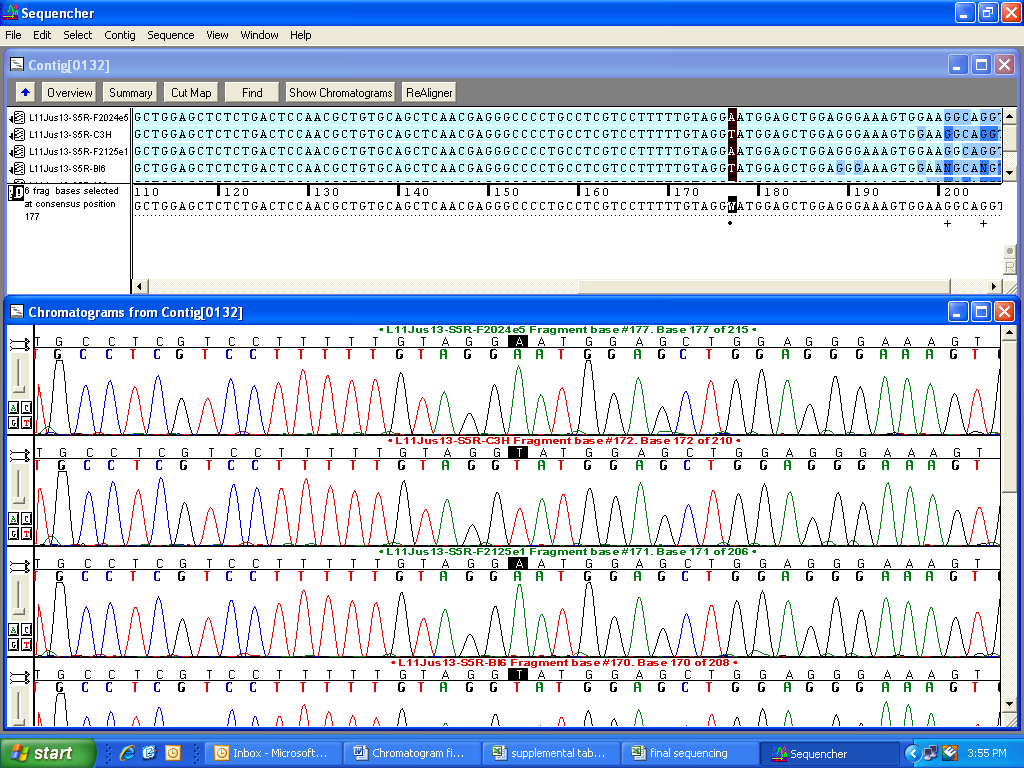
**

**Mutant Line:** *l11Jus13*

**Gene Name:** *Nxn*

**Chr. 11 base #:** 75988665

**Base Change:** T to A^R^


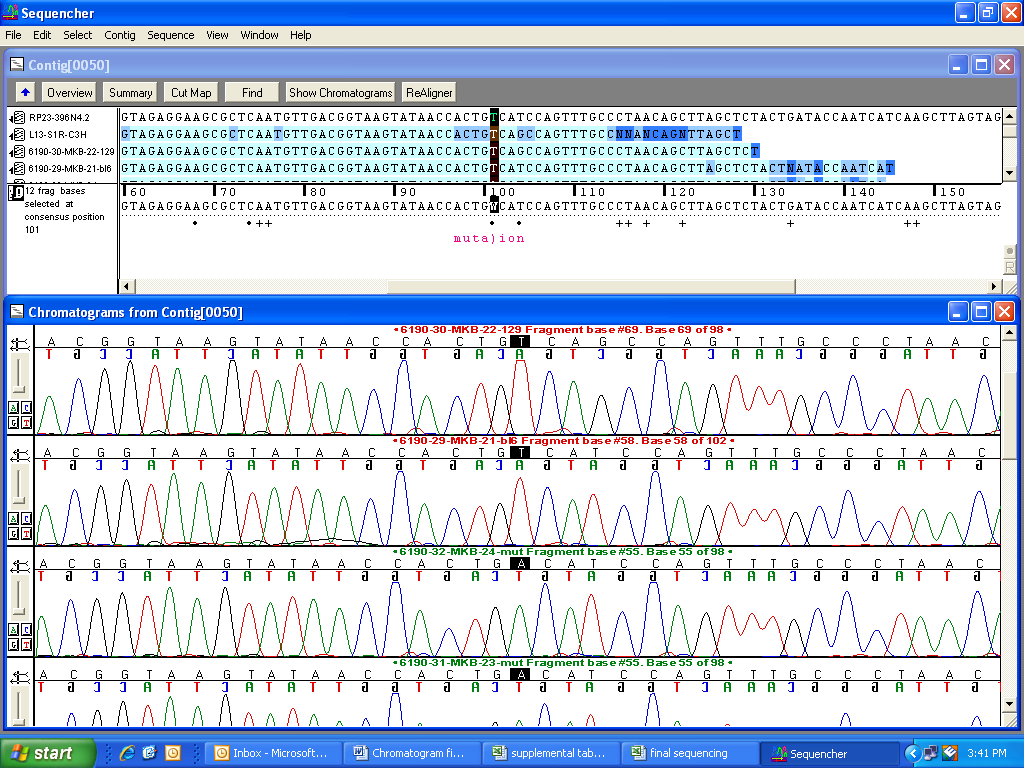
**Mutant Line:** *l11Jus13*

**Gene Name:** *RP23-396N4.2*

**Chr. 11 base #:** 104597480

**Base Change:** T to A^F^


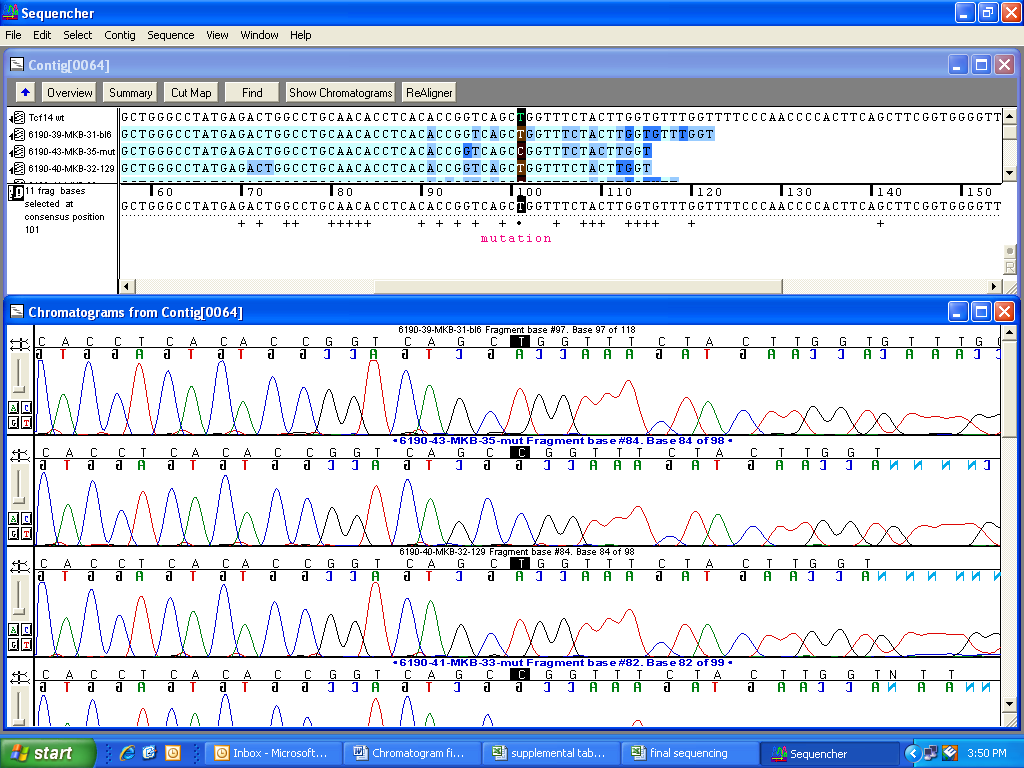


**Mutant Line:** *l11Jus13*

**Gene Name:** *Mlx*

**Chr. 11 base #:** 100912363

**Base Change:** T to C^F^

**
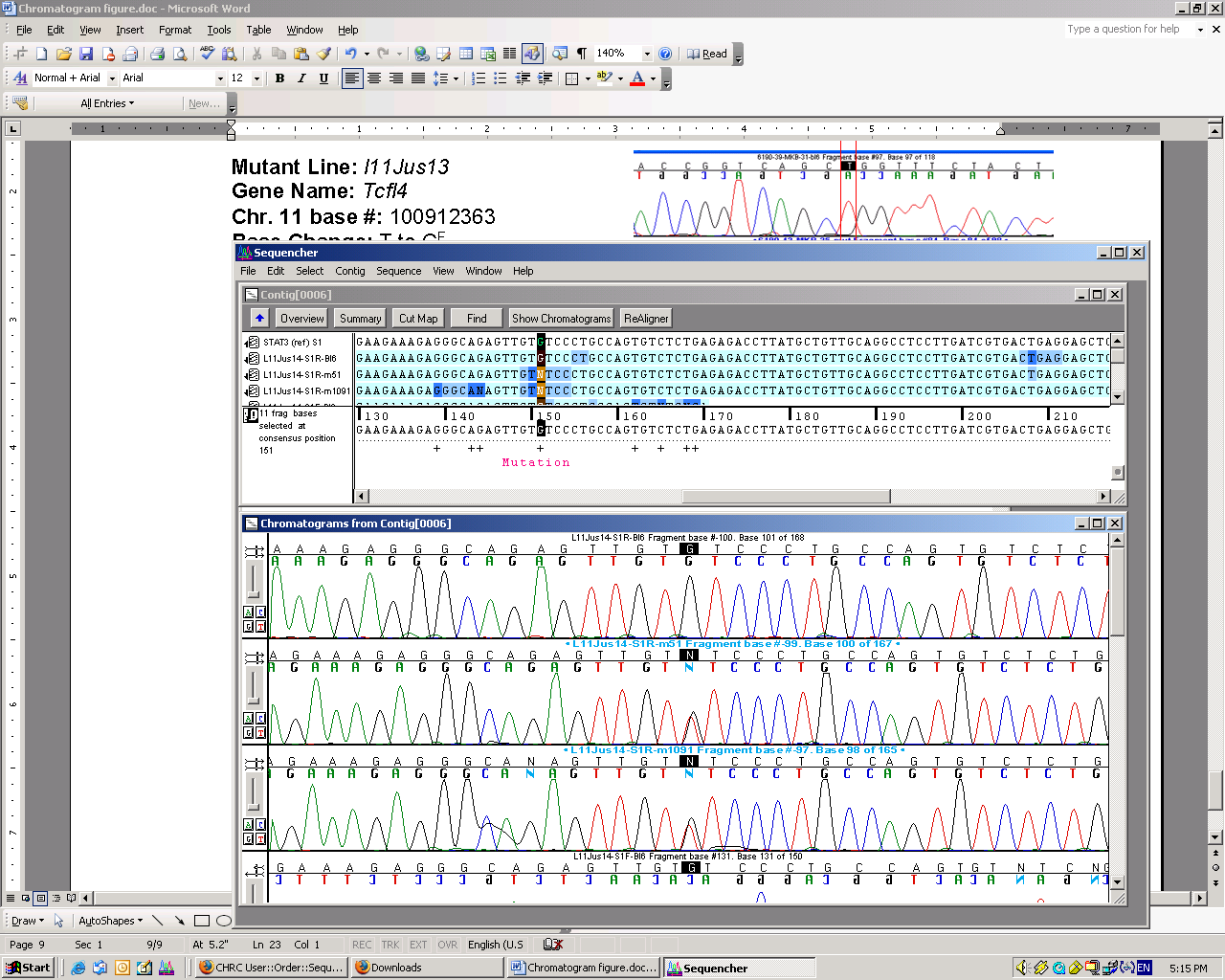
**

**Mutant Line:** *l11Jus14*

**Gene Name:** *Stat3*

**Chr. 11 base #:** 100717534

**Base Change:** G to T^R^


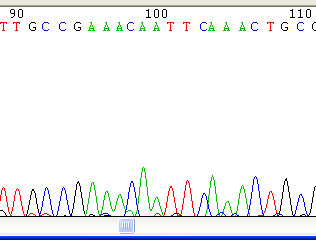

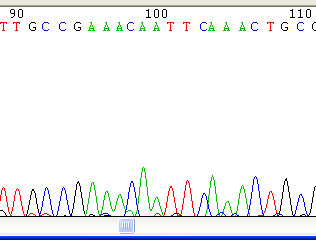


**Mutant Line:** *l11Jus15*

**Gene Name:** *Car10*

**Chr. 11 base #:** 92921139


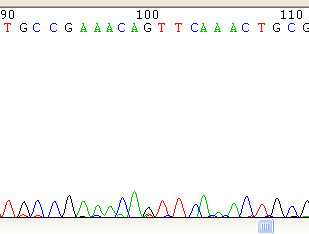

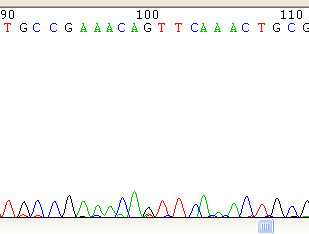


**Base Change:** A to G^F^

**Mutant Line:** *l11Jus15*


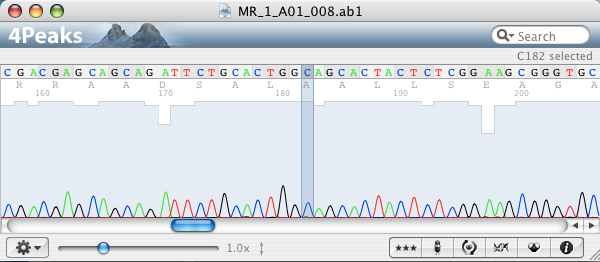

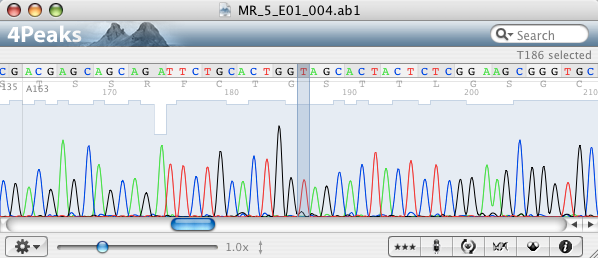


**Gene Name:** *Med31*

**Chr. 11 base #:** 71937624

**Base Change:** C to T^R^

**
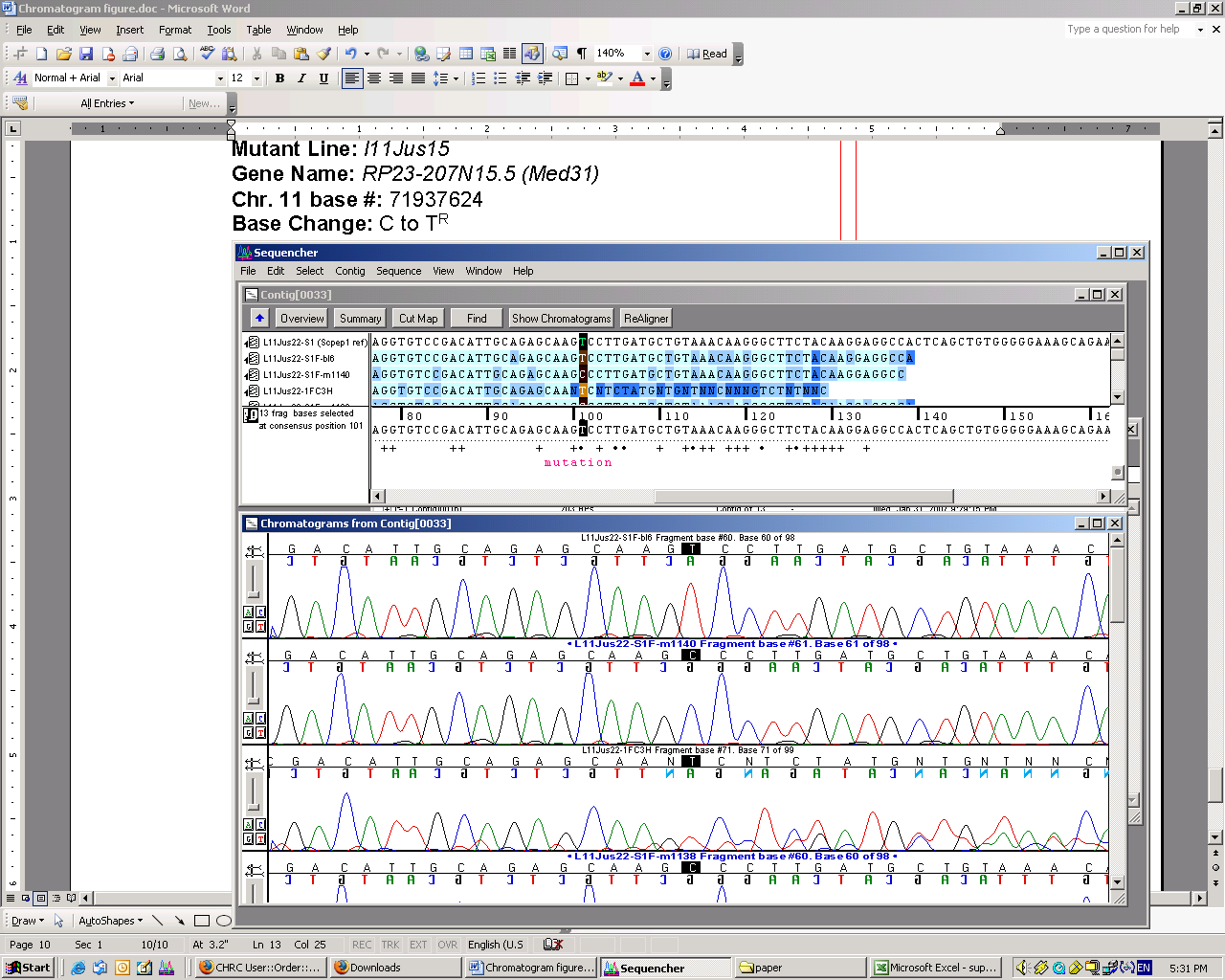
**

**Mutant Line:** *l11Jus22*

**Gene Name:** *Scpep1*

**Chr. 11 base #:** 88756987

**Base Change:** T to C^R^

**
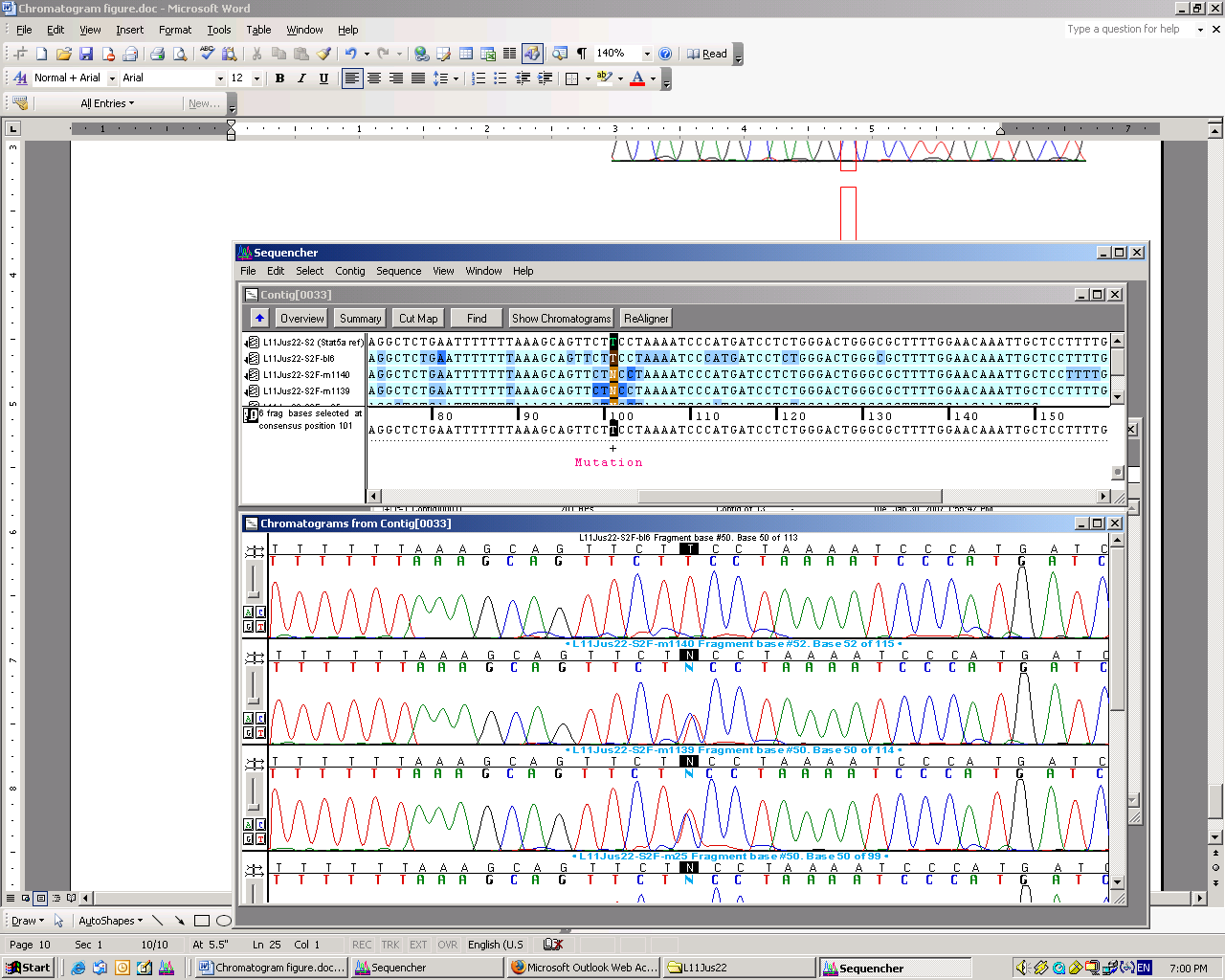
**

**Mutant Line:** *l11Jus22*

**Gene Name:** *Stat5a*

**Chr. 11 base #:** 88756987

**Base Change:** T to C^F^


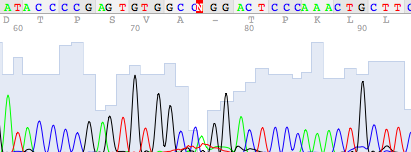


**Mutant Line:** *l11Jus27**[[2]](#endnote-3)*


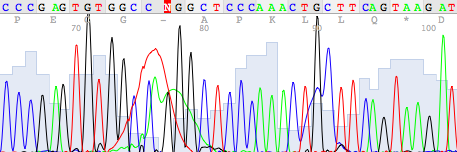
**Gene Name:** *Dhx58*

**Chr. 11 base #:** 100520453

**Base Change:** T to C^R^


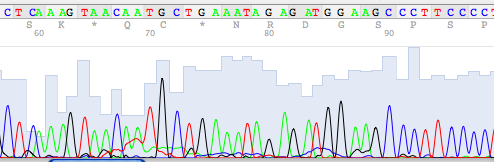

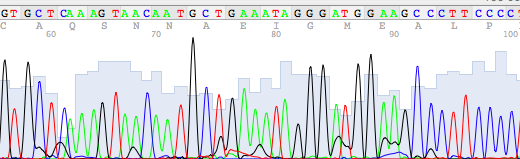


**Mutant Line:** *l11Jus27****Error: Reference source not found***

**Gene Name:** *Usp32*

**Chr. 11 base #:** 84720424

**Base Change:** T to C^R^


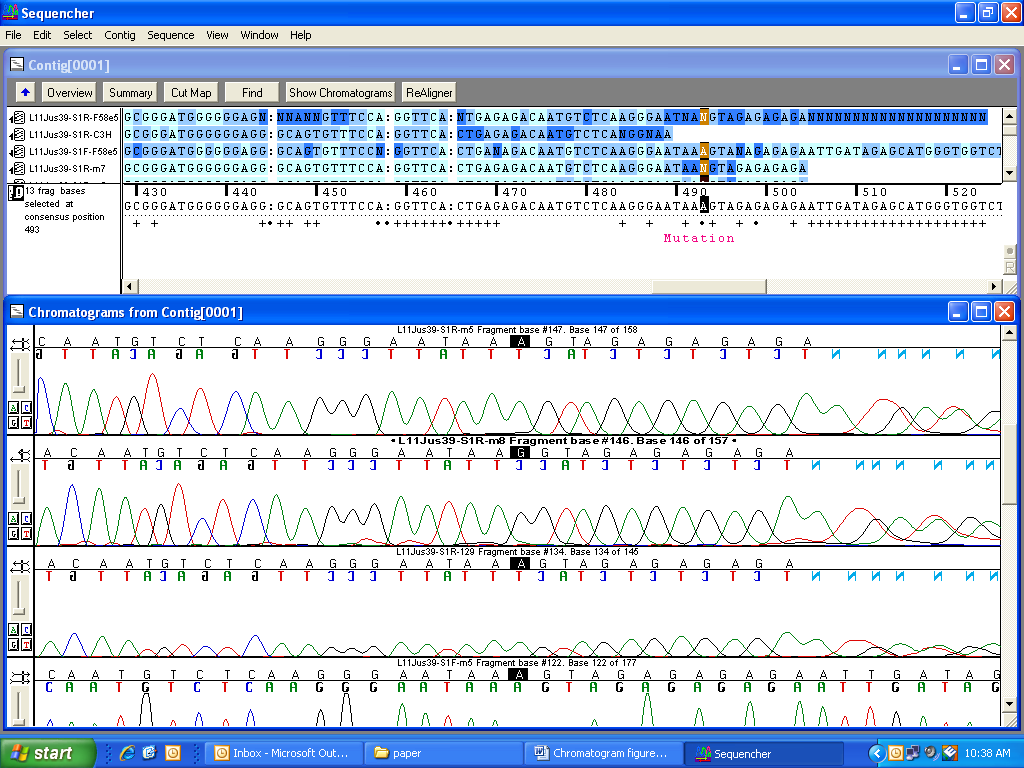


**Mutant Line:** *l11Jus39*

**Gene Name:** *RP23-350G1.1*

**Chr. 11 base #:** 81604157

**Base Change:** A to G^F^


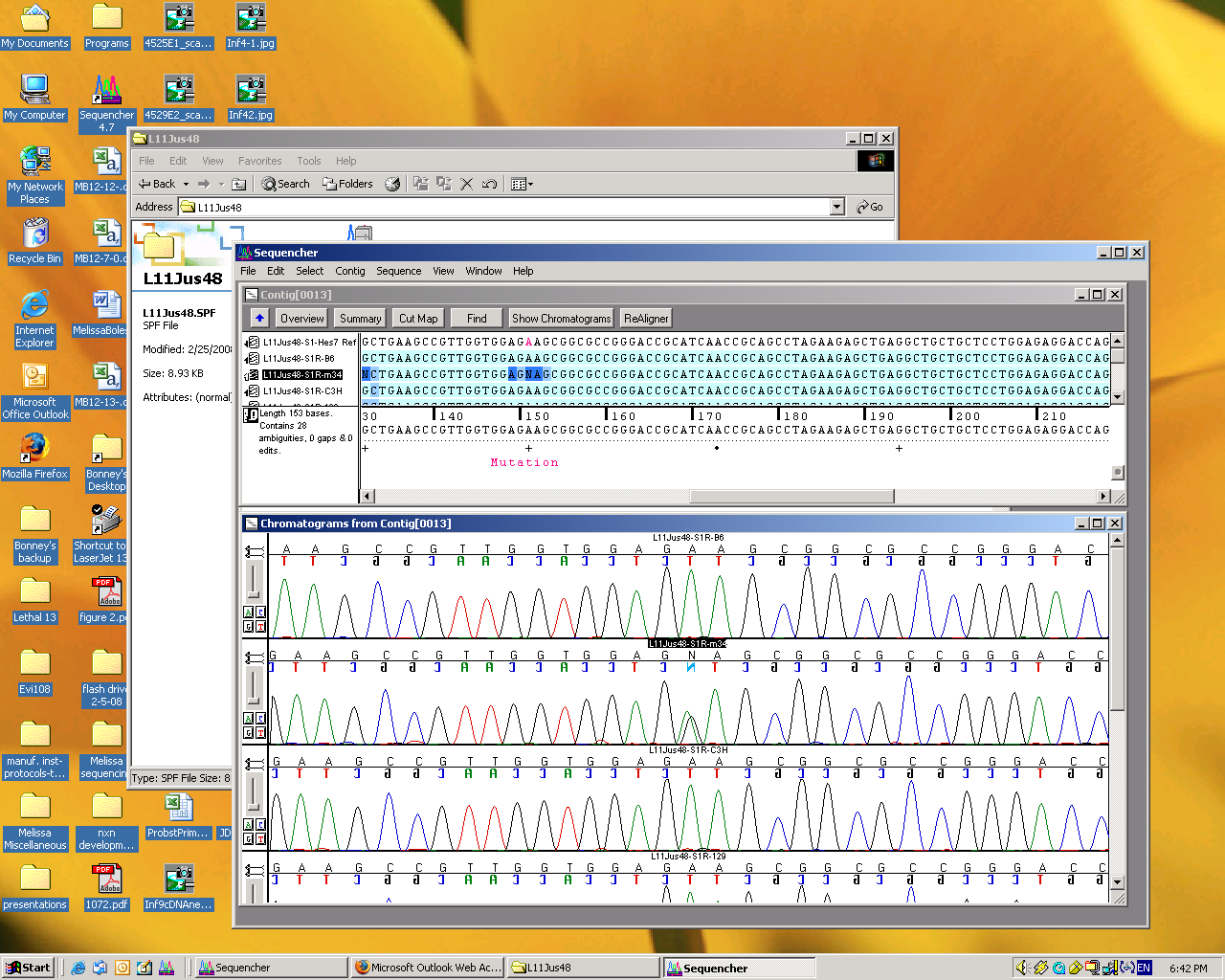


**Mutant Line:** *l11Jus48*

**Gene Name:** *Hes7*

**Chr. 11 base #:** 68847190

**Base Change:** A to G^F^

**
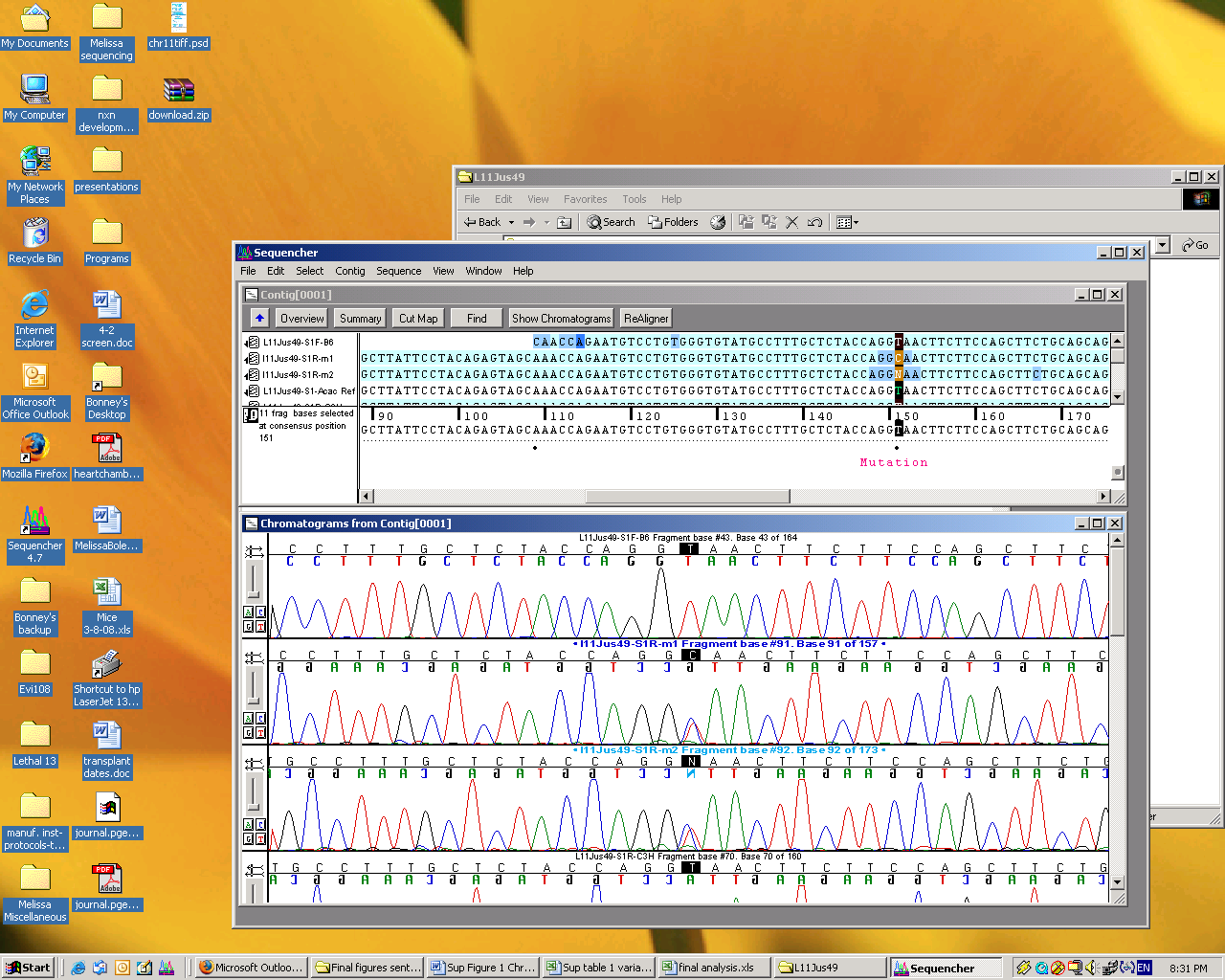
**

**Mutant Line:** *l11Jus49*

**Gene Name:** *Acaca*

**Chr. 11 base #:** 84022865

**Base Change:** T to C^F^


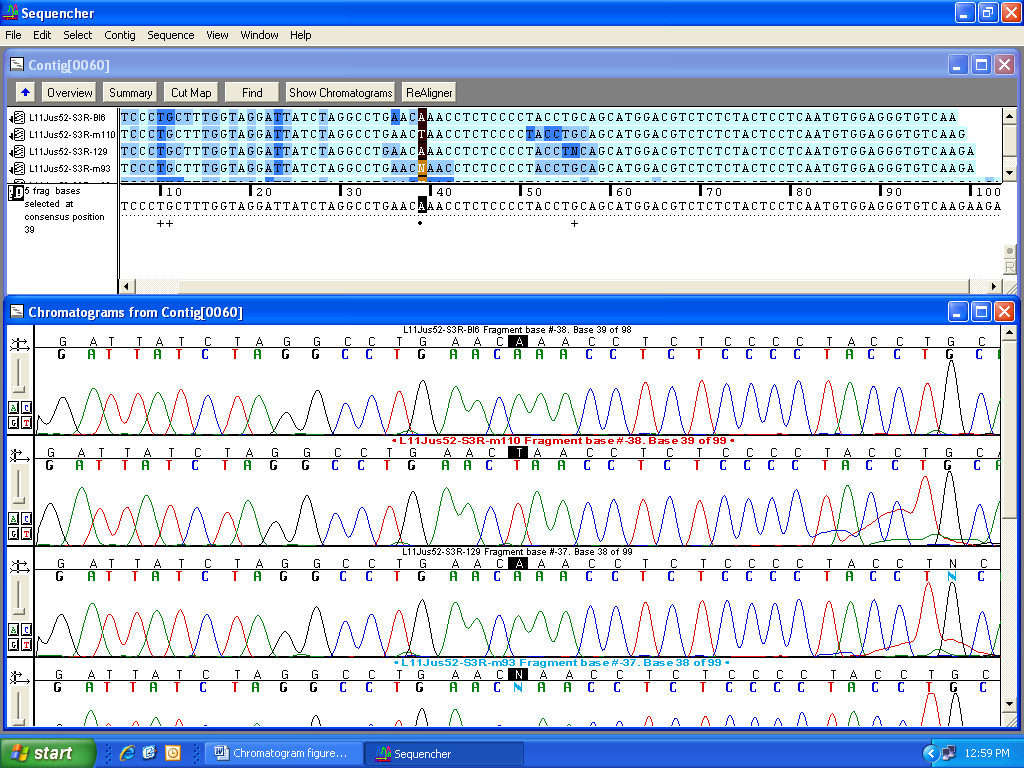


**Mutant Line:** *l11Jus52*

**Gene Name:** *Aipl1*

**Chr. 11 base #:** 71763139

**Base Change:** A to T^R^

**
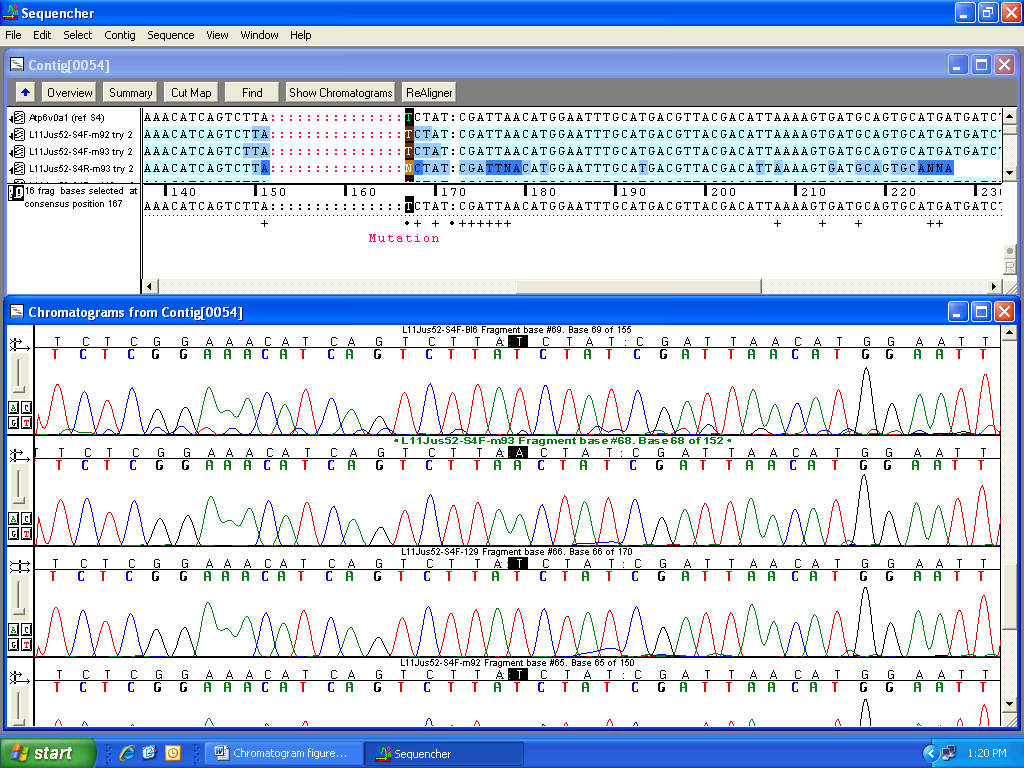
**

**Mutant Line:** *l11Jus52*

**Gene Name:** *Atp6v0a1*

**Chr. 11 base #:** 100831549

**Base Change:** T to A^F^


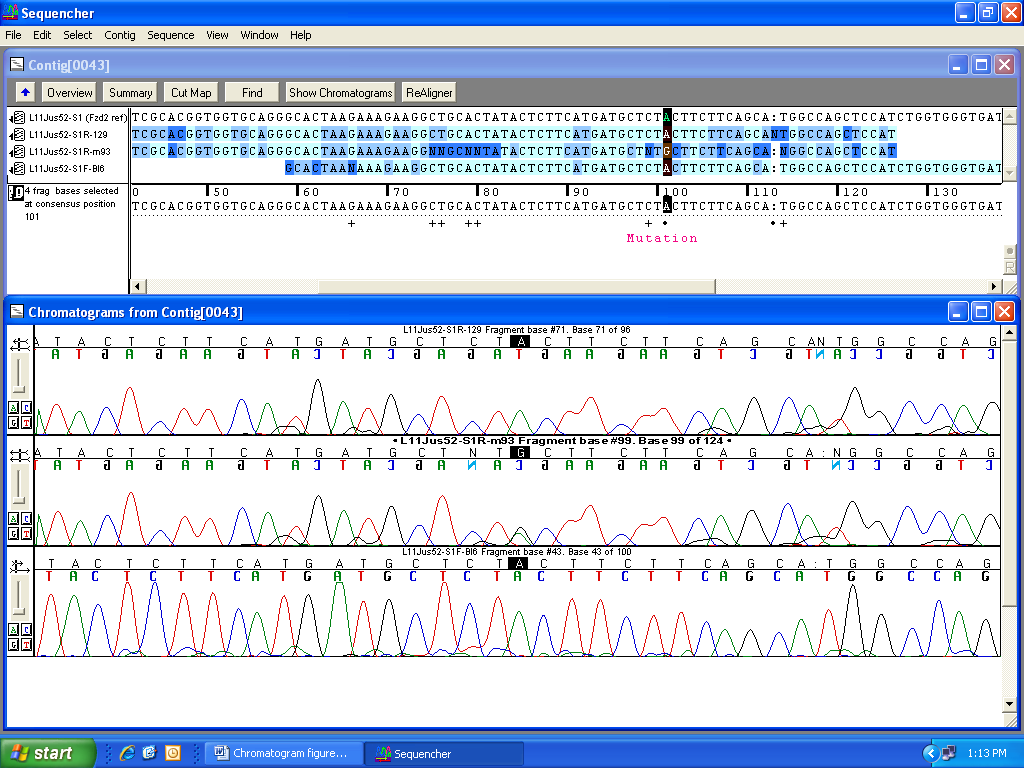


**Mutant Line:** *l11Jus52*

**Gene Name:** *Fzd2*

**Chr. 11 base #:** 102426840

**Base Change:** A to G^F^


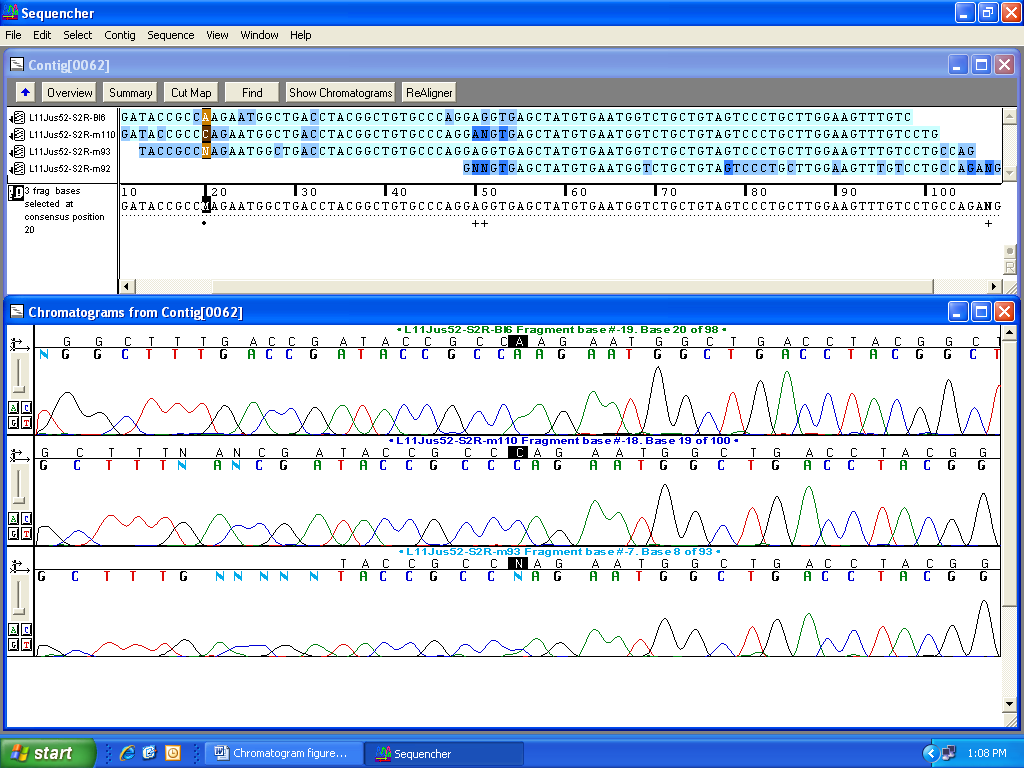


**Mutant Line:** *l11Jus52*

**Gene Name:** *Plxdc1*

**Chr. 11 base #:** 97755094

**Base Change:** A to C^R^


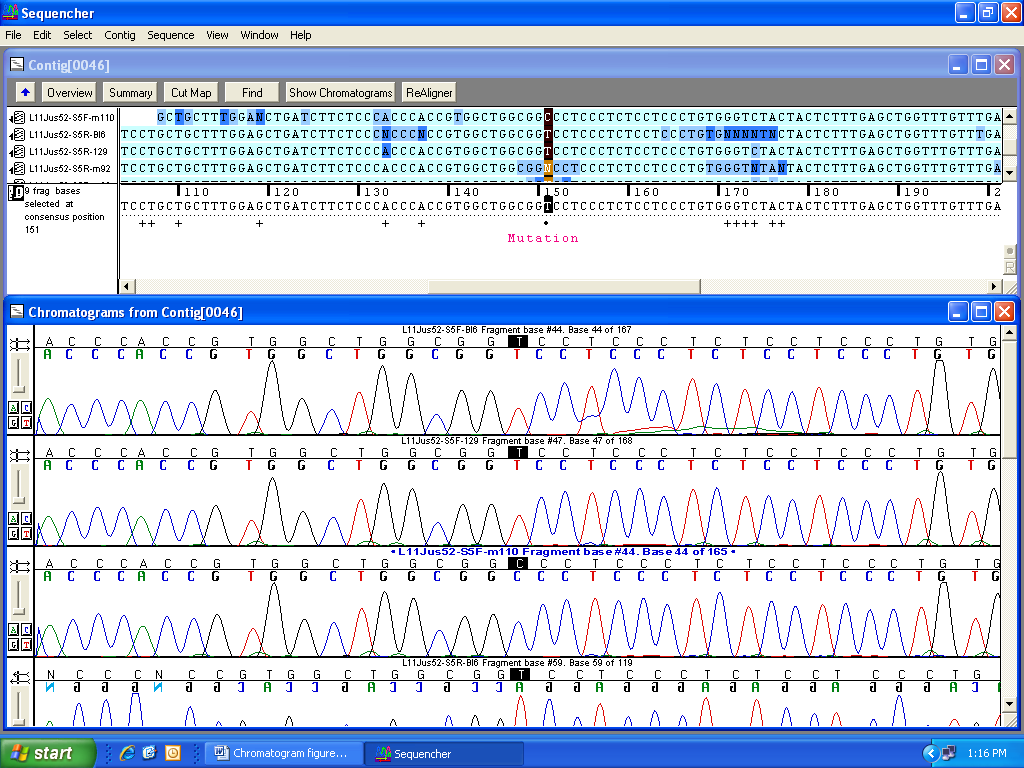


**Mutant Line:** *l11Jus52*

**Gene Name:** *Socs7*

**Chr. 11 base #:** 97214405

**Base Change:** T to C^F^


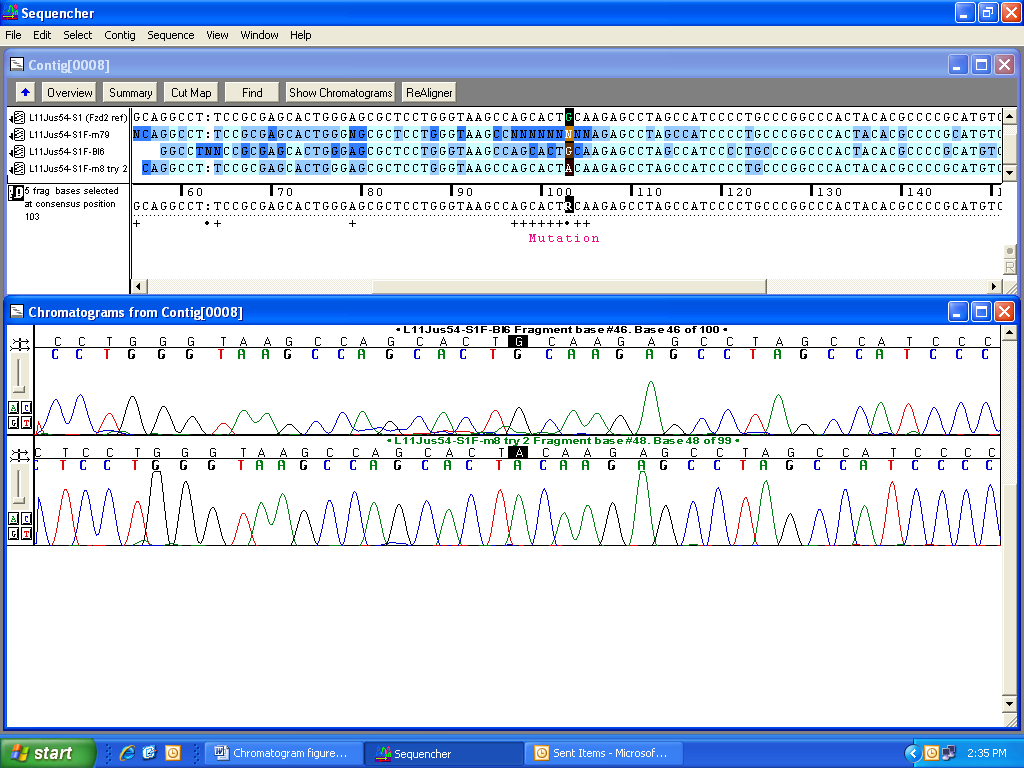


**Mutant Line:** *l11Jus54*

**Gene Name:** *Fzd2*

**Chr. 11 base #:** 102427332

**Base Change:** G to A^F^


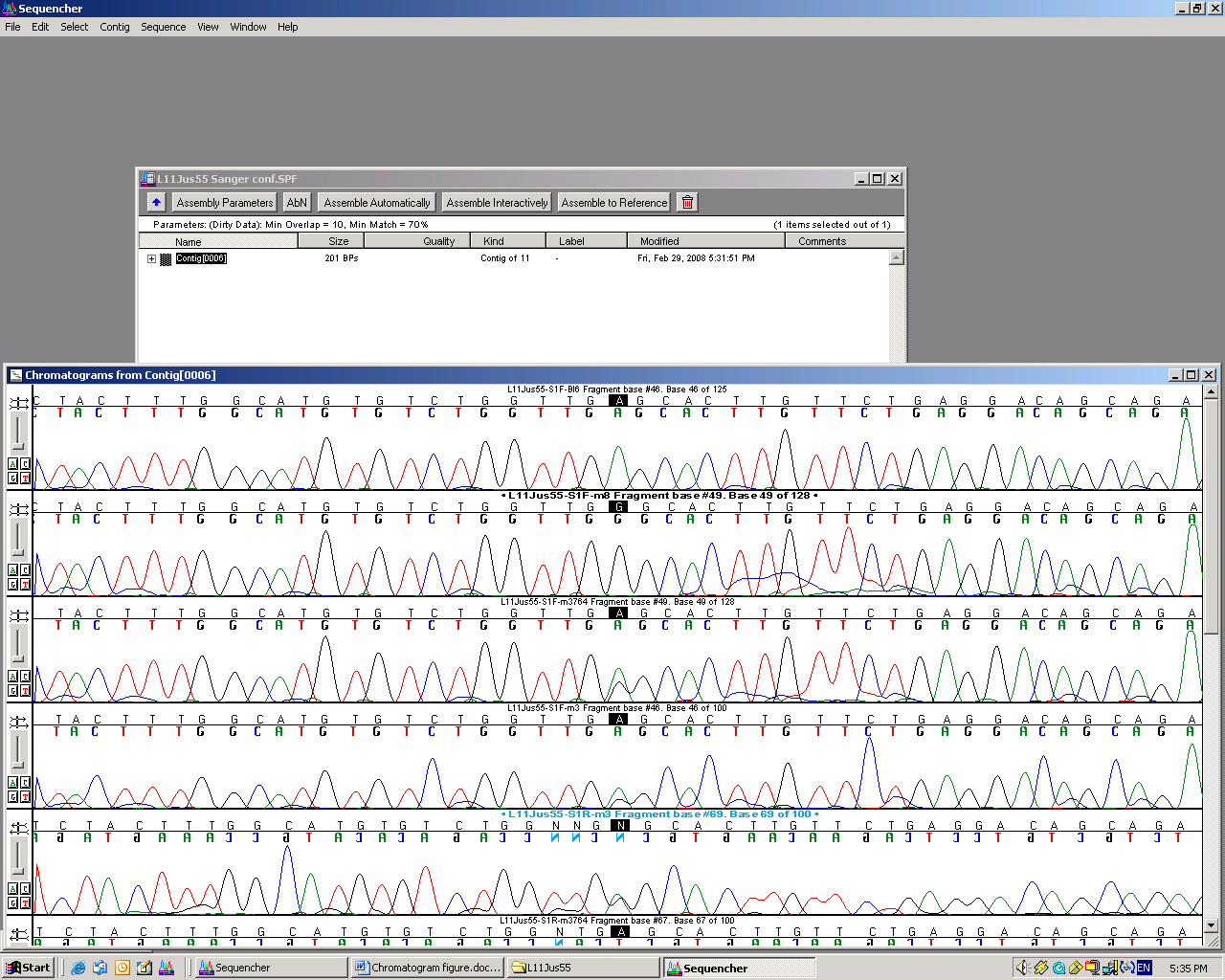


**Mutant Line:** *l11Jus55*

**Gene Name:** *Nf1*

**Chr. 11 base #:** 79304255

**Base Change:** A to G^F^


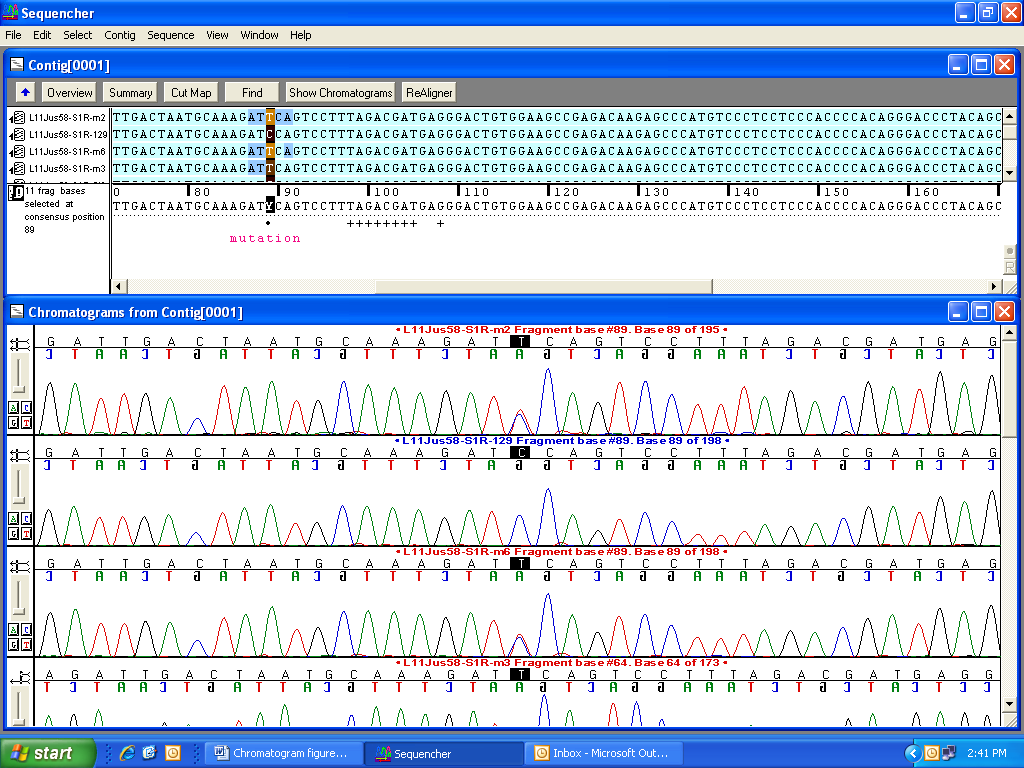
**Mutant Line:** *l11Jus58*

**Gene Name:** *Cntnap1*

**Chr. 11 base #:** 101008018

**Base Change:** C to T^F^

**
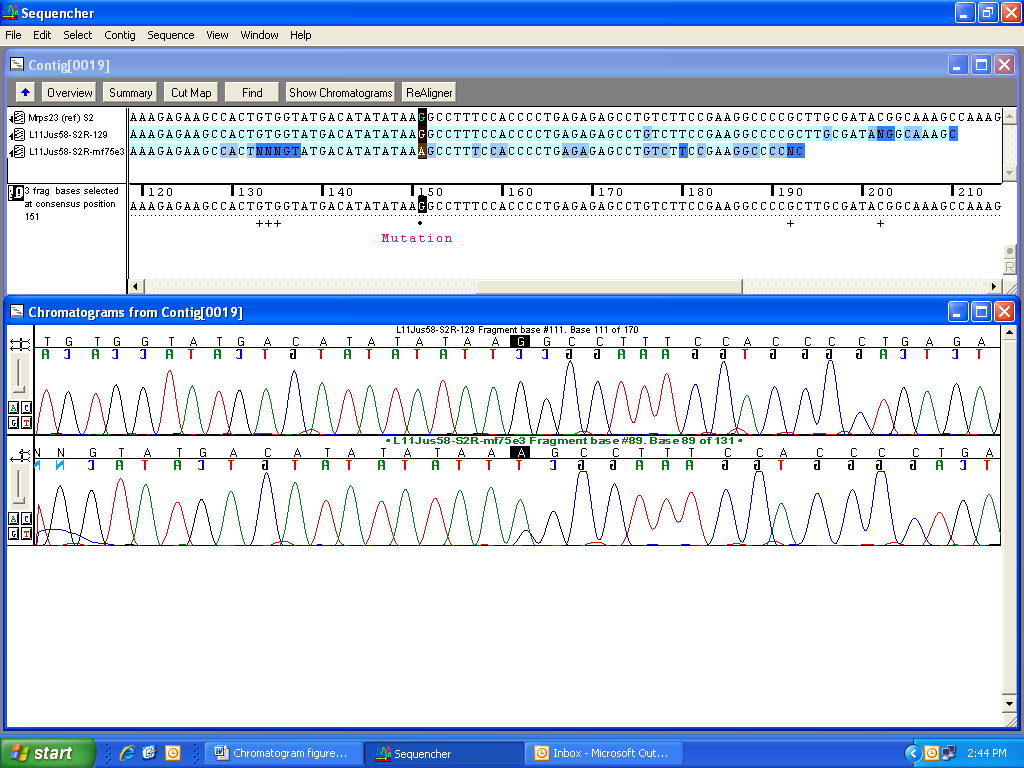
**

**Mutant Line:** *l11Jus58*

**Gene Name:** *Mrps23*

**Chr. 11 base #:** 87933245

**Base Change:** G to A^F^


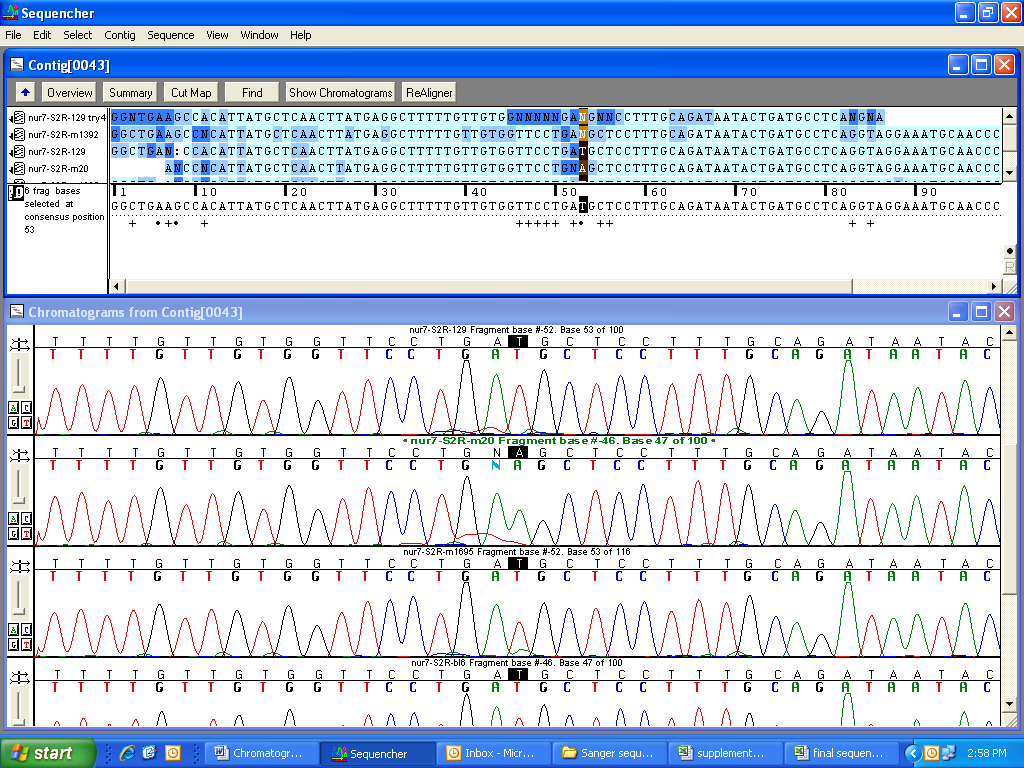


**Mutant Line:** *nur07*

**Gene Name:** *RP23-352L3.2*

**Chr. 11 base #:** 86808189

**Base Change:** T to A^R^


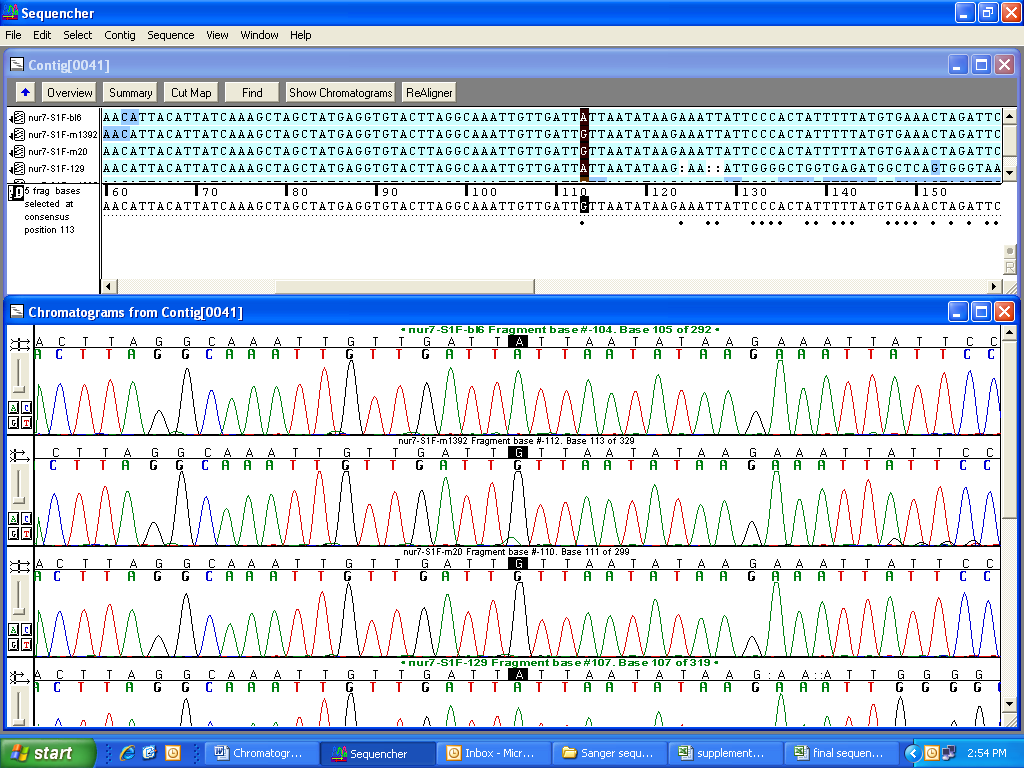


**Mutant Line:** *nur07*

**Gene Name:** *RP23-467J12.1*

**Chr. 11 base #:** 86226660

**Base Change:** A to G^F^

**
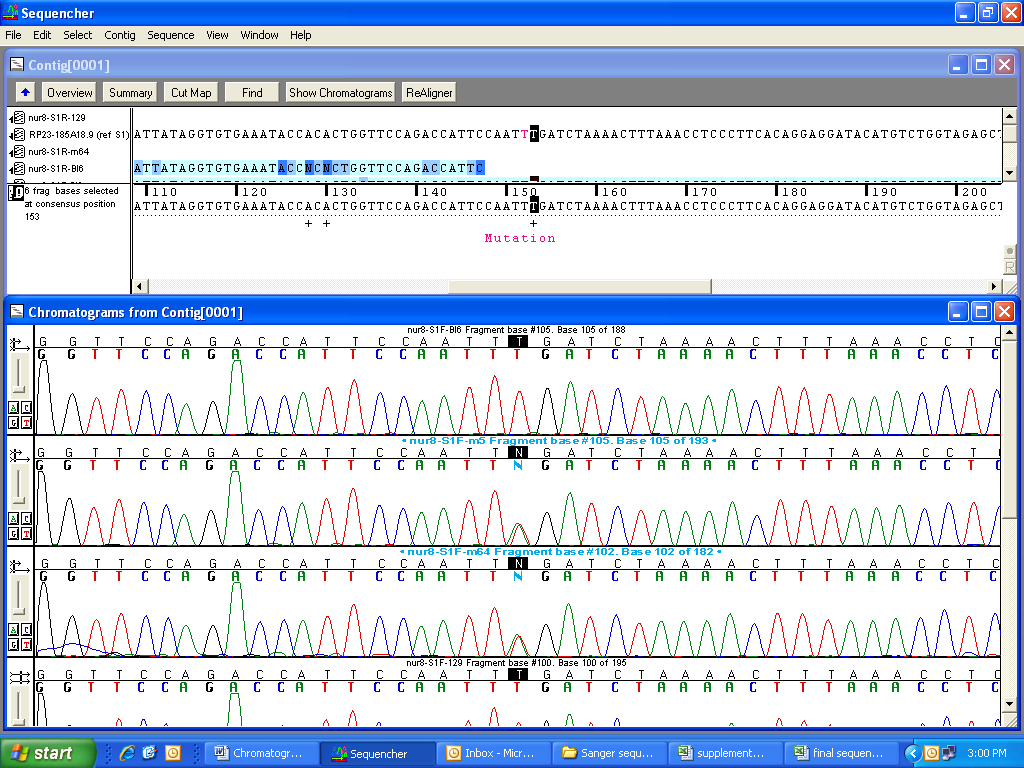
**

**Mutant Line:** *nur08*

**Gene Name:** *RP23-185A18.9*

**Chr. 11 base #:** 77991203

**Base Change:** T to A^F^


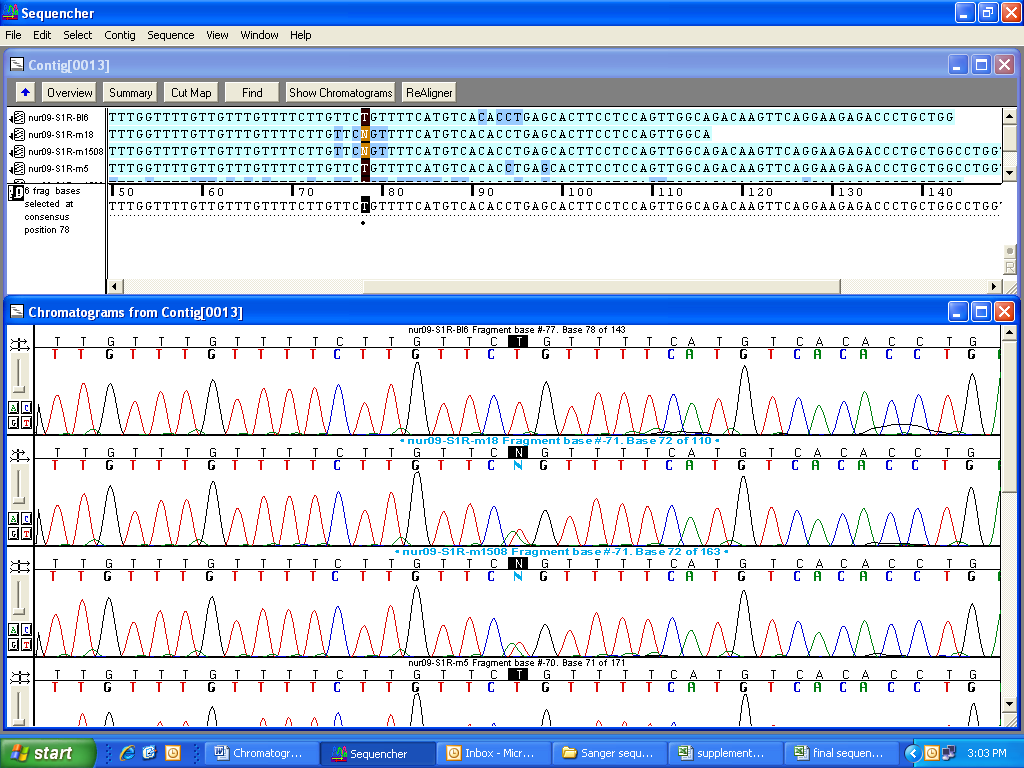
**Mutant Line:** *nur09*

**Gene Name:** *RP23-136D4.2*

**Chr. 11 base #:** 104155899

**Base Change:** T to A^R^

1. [↑](#endnote-ref-2)
2. [↑](#endnote-ref-3)
