# Supplementary figures and images for: Discovery of Candidate Disease Genes in ENU–Induced Mouse Mutants by Large-Scale Sequencing, Including a Splice-Site Mutation in Nucleoredoxin
Source: PLoS Genet. 2009 Dec 11;5(12):e1000759. doi: 10.1371/journal.pgen.1000759 (PMC2782131; doi:10.1371/journal.pgen.1000759)

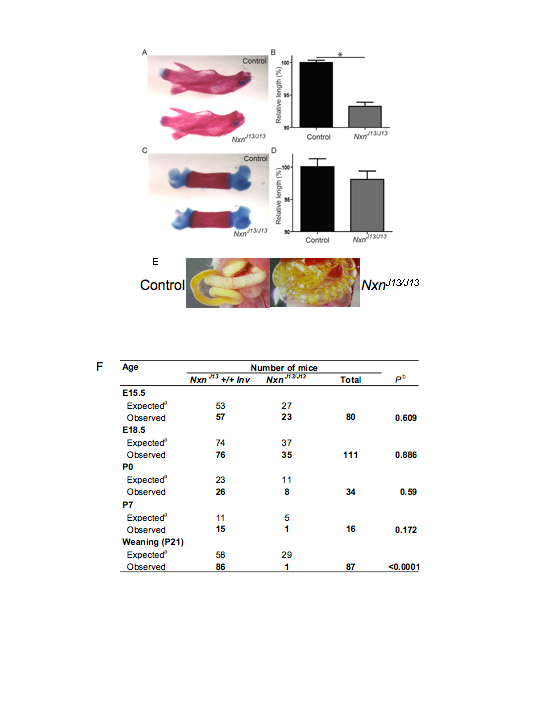

Supplement: Figure S2 — NxnJ13 mutants. (A–D) Comparison of femur length with mandible length in NxnJ13/J13 mutants. At the top are the mandibles showing a seven percent difference in length (also shown in Figure 4). At the bottom are the femurs showing no significant difference in length (p = 0.29). Error bars show the 95% confidence interval around the mean (n = 10 per genotype, mandibles and femurs). Controls were all heterozygous animals. (E) Intestines in control and mutant animals at P0. Homozygous mutants have air in the intestines, indicating a suckling defect. (F) Genotypes of offspring from NxnJ13+/+Inv×NxnJ13+/+Inv matings. (A) Homozygous Inv(11)8Brd disrupts Wnt3, and no mice were obtained with this genotype, therefore the expected ratio is 2∶1. (B) Fisher exact tests with two-tailed p values. (0.14 MB TIF) [file pgen.1000759.s002.tif]
