# Supplementary material for: Discovery of Candidate Disease Genes in ENU–Induced Mouse Mutants by Large-Scale Sequencing, Including a Splice-Site Mutation in Nucleoredoxin
Source: PLoS Genet. 2009 Dec 11;5(12):e1000759. doi: 10.1371/journal.pgen.1000759 (PMC2782131; doi:10.1371/journal.pgen.1000759)
Supplement: Table S1 — Sequence contigs on mouse Chromosome 11. The cumulative gap size is approximately 100 kb and centromere (3 Mb allocated in Accessioned Golden Pathy) (AGP). Note: Telmeric sequence reached. * Optical Map is a technique for generating a high resolution map of the structure of a chromosome or genome. (0.02 MB DOC) [file pgen.1000759.s003.doc]

Table S1: Sequence contigs on mouse chromosome 11

**Contig**  **Size (Mb) Gaps (kb)**

1: CR954170 to AL669849 31,433,409 bp ctg1-ctg2 = approx. 90kb by FIBER FISH

2: CT005230 to CR405681 53,910,979 bp ctg2-ctg3 = 6.8kb (specifically 6755 bp) - sequenced in a different mouse strain

3: BX664717 to CR556721 33,435,024 bp & ctg2-ctg3 has gap size of 6.7 kb (6692bp) by Optical Map*
