# Supplementary material for: Discovery of Candidate Disease Genes in ENU–Induced Mouse Mutants by Large-Scale Sequencing, Including a Splice-Site Mutation in Nucleoredoxin
Source: PLoS Genet. 2009 Dec 11;5(12):e1000759. doi: 10.1371/journal.pgen.1000759 (PMC2782131; doi:10.1371/journal.pgen.1000759)
Supplement: Table S2 — Gene content differences between mouse and human. (0.03 MB DOC) [file pgen.1000759.s004.doc]

**Table S2: Gene content differences between mouse and human**

**A: Genes present in mouse, but not found or found as pseudogenes, or of a significantly different size, in human**

**Mouse Vega ID Gene name Description Human Vega ID Note**

OTTMUSG00000000137 *Poldip2*  polymerase (DNA-directed), delta interacting protein 2 OTTHUMG00000132065 polymorphic pseudogene

OTTMUSG00000000970 *Expi*  extracellular proteinase inhibitor

OTTMUSG00000000971 (RP23-430I21.1) (novel protein similar to Expi)

OTTMUSG00000001044 (RP23-351G6.1) (putative novel protein)

OTTMUSG00000002038 (RP23-290B5.6) (putative novel protein)

OTTMUSG00000002039 (RP23-290B5.7) (novel transcript)

OTTMUSG00000003383 (RP23-175A18.3) (putative novel protein) OTTHUMG00000156148 unitary pseudogene

OTTMUSG00000003416 *Tcam1* testicular cell adhesion molecule 1 OTTHUMG00000154404 transcribed unitary pseudogene

OTTMUSG00000003612 *Myo15b*  myosin XVB OTTHUMG00000142197

OTTMUSG00000003730 *Tha1*  threonine aldolase 1 OTTHUMG00000132125 unitary pseudogene

OTTMUSG00000004130 (RP23-37J21.9) (novel transcript)

OTTMUSG00000004146 (RP23-82I5.2) (putative novel protein) OTTHUMG00000156169 unitary pseudogene

OTTMUSG00000004290 *Tex19.1* testis expressed gene 19.1 OTTHUMG00000132857 187aa shorter CDS (164aa vs 351aa)

OTTMUSG00000005873 *Zfp286* zinc finger protein 286

OTTMUSG00000005900 (RP23-219K7.4) (putative novel protein)

OTTMUSG00000006031 (RP23-172M21.16) (novel protein similar to Ppp1r2) OTTHUMG00000132361 unitary pseudogene

**B: Genes present in human but not found or found as pseudogenes in mouse**

**Vega ID Gene name Description Mouse Vega ID Note**

OTTHUMG00000058996 *ZNF624*  zinc finger protein 624

OTTHUMG00000059178 *CCDC144A* coiled-coil domain containing 144A CCDC cluster smaller in mouse

OTTHUMG00000059513 *CCDC144C*  coiled-coil domain containing 144C CCDC cluster smaller in mouse

OTTHUMG00000132223 (novel transcript)

OTTHUMG00000141762 *C17orf55*  chromosome 17 open reading frame 55

OTTHUMG00000141763 *TMEM105* transmembrane protein 105

OTTHUMG00000141788 *DBF4B* DBF4 homolog B (*S. cerevisiae*) OTTMUSG0000033937 unitary pseudogene

OTTHUMG00000141804 *STH* saitohin

OTTHUMG00000141921 (putative novel protein)

OTTHUMG00000141995 (novel transcript)

OTTHUMG00000142203 *ZACN* zinc activated ligand-gated ion channel

OTTHUMG00000142244 *C17orf54* chromosome 17 open reading frame 54

OTTHUMG00000154861 *CDK3* cyclin-dependent kinase 3
