# Supplementary material for: Discovery of Candidate Disease Genes in ENU–Induced Mouse Mutants by Large-Scale Sequencing, Including a Splice-Site Mutation in Nucleoredoxin
Source: PLoS Genet. 2009 Dec 11;5(12):e1000759. doi: 10.1371/journal.pgen.1000759 (PMC2782131; doi:10.1371/journal.pgen.1000759)
Supplement: Table S3 — Structural characteristics of annotated genes structures from mouse Chromosome 11. 1 Of these, 450 were processed transcripts that were not likely to encode a protein. (0.04 MB DOC) [file pgen.1000759.s005.doc]

**Table S3: Structural characteristics of annotated genes structures from mouse chromosome 11**

| **Gene Structure Class** | **Number of genes** | **Total transcribed length (bp)** | **Mean gene length (bp)** | **Mean transcripts/gene** | **Mean exons/gene** |
| --- | --- | --- | --- | --- | --- |
| **Novel and known protein coding genes and processed transcripts^1^**  **(*Trp53 – Wnt3*)** | 2,047  (905) | 60,461,569  (8,707,000 Mb) | 30,520 | 3.30 | 8.01 |
| Novel protein coding | 113 | 1,693,212 | 14,984 | 1.80 | 3.60 |
| Known protein coding | 1,484 | 54,176,557 | 14,625 | 3.90 | 3.60 |
| **Pseudogenes** | 498 | 501,274 | 1,007 | 1.01 | 1.13 |
| **Total structures** | 2,545 | 60,891,301 | 24,735 | 2.80 | 6.80 |
