# Supplementary material for: Discovery of Candidate Disease Genes in ENU–Induced Mouse Mutants by Large-Scale Sequencing, Including a Splice-Site Mutation in Nucleoredoxin
Source: PLoS Genet. 2009 Dec 11;5(12):e1000759. doi: 10.1371/journal.pgen.1000759 (PMC2782131; doi:10.1371/journal.pgen.1000759)
Supplement: Table S4 — Variants found by resequencing exons and their flanking 125 bp in the Trp53-Wnt3 interval. Lesions found only once in a single line are shown. Each lesion was confirmed by re-sequencing 2–4 DNAs from additional mutant individuals from each line. The B6/129 SNPs are likely to be new SNPs that arose in our substrain, or may not have been identified. No mutations were found or confirmed in 8 lines (crf05, inf4, gro40, l11Jus38, l11Jus45, nur01, nur05, and skc1). Hom: Indicates that DNA from a homozygous mutant was used for re-sequencing. Het: Indicates that DNA from a heterozygous mutant was used for re-sequencing. F: Denotes forward gene, forward strand sequence provided (coding sequence). R: Denotes reverse gene, reverse complement of forward strand sequence provided (coding sequence). 1 Twelve lesions did not confirm in any animals giving an error rate for re-sequencing of 15% (12/80). 2 Mutation was discovered independently of chr 11 resequencing effort. 3 Unable to successfully PCR and amplify and sequence the genomic region flanking this mutation so this mutation was not confirmed. 4 Kile, B.T. et. al. Nature 2003 [5]. 5 Traka, M. et. al. J. Neurosci. 2008 [19]. (0.22 MB DOC) [file pgen.1000759.s006.doc]

Table S4: Variants found by resequencing exons and their flanking 125 bp in the *Trp53-Wnt3* interval

| **Mutant Line** | **Mutation** | **Gene name** | **MGI Symbol** | **ENS#** | **Surrounding Sequence** | **Base Change** | **Type of Lesion** | **Confirmation^1^** |
| --- | --- | --- | --- | --- | --- | --- | --- | --- |
| *crf02^Hom^* | exon 1 | Heat shock protein, alpha-crystallin-related, B9 *(Hspb9)* | 1922732 | ENSMUSG00000017832 | TTTCTCCACC**G**GGCAGCGCGA^F^ | G to A | G to R | 3/3 |
| *crf05^Hom^* | no mutations |  |  |  |  |  |  |  |
| *crf06^Hom^* | 3' UTR | Max binding protein *(Mnt)* | 109150 | ENSMUSG00000000282 | CCAGCGAAGG**T**CCTGAATACA^F^ | T to C | noncoding | 2/3 |
|  | intron 6 | Phospholipid scramblase 3 *(Plscr3)* | 1917560 | ENSMUSG00000019461 | TCTCCCATTG**A**CGGTGACCTT^F^ | A to G | noncoding | 2/3 |
|  | downstream | Receptor (calcitonin) activity modifying protein 2 *(Ramp2)* | 1859650 | ENSMUSG00000001240 | AAGAATCTAG**A**TGGCCCTGCT^F^ | A to T | noncoding | 2/3 |
| *crf08^Hom^* | exon 1 | Olfactory receptor 394 *(Olfr394)* | 3030228 | ENSMUSG00000056921 | CATCATTGTT**A**TTCCATTCCT^R^ | A to G | I to V | 2/2 |
|  | exon 10 | Mediator complex subunit 13 *(Med13)* | 3029632 | [ENSMUSG00000034297](http://www.ensembl.org/Mus_musculus/geneview?gene=ENSMUSG00000034297) | GCAGTGTAAG**A**AGCCTTTAAA^R^ | A to G | K to E | 2/2 |
| *crf12^Hom^* | exon 14 | Centaurin, beta 1 *(Centb1)* | 2388270 | ENSMUSG00000001588 | GTACAAAGCG**T**GGATGGAAAT^R^ | T to A | V to E | 3/3 |
|  | intron 4 | Gastric inhibitory polypeptide *(Gip)* | 107504 | ENSMUSG00000014351 | CTAGTGAGTA**A**TGTCCTAGAG^F^ | A to C | noncoding | 3/3 |
|  | intron 3 | Mannose-P-dolichol utilization defect 1 *(Mpdu1)* | 1346040 | ENSMUSG00000018761 | CCAGGCATGG**T**GGGAGTGGCA^R^ | T to A | noncoding | 3/3 |
|  | 3' UTR | Mediator complex subunit 13 *(Med13)* | 3029632 | [ENSMUSG00000034297](http://www.ensembl.org/Mus_musculus/geneview?gene=ENSMUSG00000034297) | TCTCAATGTA**C**AGTTTAACAG^R^ | C to A | noncoding | 3/3 |
| *crf18^Hom^* | exon 2 | Kelch-like 10 *(Klhl10)* | 2181067 | ENSMUSG00000001558 | ACTGAGAAGA**A**GGTTTACAAC^F^ | A to G | K to R | 2/2 |
| *crf26^Hom^* | exon 7 | Coiled-coil domain containing 55 *(Ccdc55)* | 2144305 | [ENSMUSG00000037958](http://www.ensembl.org/Mus_musculus/geneview?gene=ENSMUSG00000037958) | GAGGAAACTG**T**GATGTCAGCT^R^ | T to A | V to E | 3/3 |
|  | intron 4 | TAO kinase 1 *(Taok1)* | 1914490 | ENSMUSG00000017291 | GTTCAGGGCA**T**TCAACTCTTA^R^ | T to A | noncoding | 3/4 |
| *gro01^Hom^* | exon 12 | Dishevelled 2 *(Dvl2)* | 106613 | ENSMUSG00000020888 | CCCAAACGCC**T**TTCTAGGTAT^F^ | T to G | F to V | 3/3 |
|  | intron 20 | Nitric oxide synthase 2, inducible, macrophage *(Nos2)* | 97361 | ENSMUSG00000020826 | CCGGCCCTGG**A**CAAATCTCTA^F^ | A to G | noncoding | 3/3 |
|  | intron 6 | Tnf receptor associated factor 4 *(Traf4)* | 1202880 | ENSMUSG00000017386 | CCCTCCGCCA**T**TGTCCTGTTC^R^ | T to A | noncoding | 3/3 |
| *gro22^Hom^* | intron 14 | ATP-binding cassette, sub-family C (CFTR/MRP), member 3 *(Abcc3)* | 1923658 | ENSMUSG00000020865 | CTTCTCCCTG**A**ACTCTGAAGC^R^ | A to G | noncoding | 4/4 |
|  | intron 1 | Active BCR-related gene *(Abr)* | 107771 | ENSMUSG00000017631 | TCCCTGGGTG**G**GGGACAGGGG^R^ | G to T | noncoding | 3/3 |
|  | intron 17 | Mbt domain containing 1 *(Mbtd1)* | 2143977 | ENSMUSG00000059474 | AGAAATCTTT**T**CCAAAAGGCT^F^ | T to C | noncoding | 3/3 |
|  | intron 6 | Methyltransferase 10 domain containing *(Mett10d)* | 1914743 | ENSMUSG00000010554 | ATCTCACCTG**T**TTTTCTCTAC^F^ | T to A | noncoding | 4/4 |
| *gro40^Hom^* | no mutations |  |  |  |  |  |  |  |
| *gro41^Hom^* | exon 10 | SH3 and cysteine rich domain 2 *(Stac2)* | 2144518 | ENSMUSG00000017400 | GAATGTTTGG**C**GATGCTGTCA^R^ | C to A | R to R | 3/3 |
| *gro42^Hom^* | intron 15 | N-ethylmaleimide sensitive fusion protein *(Nsf)* | 104560 | ENSMUSG00000034187 | GAGGCAGAGG**C**AGGTGAATGC^R^ | C to A | noncoding | 3/3 |
|  | upstream | WD repeat domain 79 *(Wdr79)* | 2384933 | ENSMUSG00000041346 | CCCACTGCCC**C**ACCCCCAAAC^R^ | C to T | noncoding | 0/3 |
| *inf3^Hom^* | 3' UTR | Carboxypeptidase D *(Cpd)* | 107265 | ENSMUSG00000020841 | GTAAGACTCA**C**ACTTACAAAA^R^ | C to A | noncoding | B6/129 SNP |
|  | 3' UTR | Trans-acting transcription factor 6 *(Sp6)* | 1932575 | ENSMUSG00000038560 | TGTCTCTCTG**T**CCCCTTCTTT^F^ | T to A | noncoding | 3/3 |
| *inf4^Hom^* | intron 12 | V-erb-b2 erythroblastic leukemia viral oncogene homolog 2 *(Erbb2)* | 95410 | ENSMUSG00000062312 | TGCATGTTCT**A**GCACCAACCC^F^ | A to G | noncoding | 0/3 |
|  | intron 1 | Transmembrane protein 100 *(Tmem100)* | 1915138 | ENSMUSG00000069763 | CCTTGTCTGC**A**GTTAAGTATT^F^ | A to T | noncoding | 0/3 |
| *inf7^Hom^* | exon 4 | Pleckstrin homology domain containing, family M (with RUN domain) member 1 *(Plekhm1)* | 2443207 | ENSMUSG00000034247 | TCTCACTACC**T**CTGGTGCAGA^R^ | T to C | S to P | 3/3 |
|  | downstream | RP23-263M10.5 *(Novel)* | [1200014J11Rik](http://www.informatics.jax.org/searches/accession_report.cgi?id=MGI%3A1914124) | [ENSMUSG00000020783](http://www.ensembl.org/mus_musculus/geneview?gene=ENSMUSG00000020783) | ATTTGGCTTC**T**CATCTGACTT^F^ | T to C | noncoding | 3/3 |
|  | intron 2 | RP23-350G1.1 *(Novel)* | 5530401A14Rik | [ENSMUST00000021044](http://www.ensembl.org/Mus_musculus/transview?transcript=ENSMUST00000021044) | AATGGGATTG**G**GTGCATTTAC^F^ | G to A | noncoding | 3/3 |
| *l11Jus03^Het^* | intron 9 | G protein-coupled receptor kinase-interactor 1 *(Git1)* | 1927140 | ENSMUSG00000011877 | AATGGCCTGG**A**CTCTCTTCCT^F^ | A to C | noncoding | 3/3 |
| *l11Jus05^Het^* | 3' UTR | Mediator complex subunit 13 *(Med13)* | 3029632 | ENSMUSG00000034297 | AGTCACAGTA**T**TTGTGAAAAG^R^ | T to A | noncoding | 3/3 |
| *l11Jus06^Het^* | downstream | RNA-binding protein Musashi homolog 2 *(Msi2h)* | 1923876 | ENSMUSG00000069769 | AAAACTCAGA**C**ATCGGGCAAA^R^ | C to T | noncoding | B6/129 SNP |
|  | intron 2 | Transmembrane and immunoglobulin domain containing 1 *(Tmigd1)* | 1913851 | ENSMUSG00000020839 | TGTAAATCTA**T**ATAGATTGAT^F^ | T to C | noncoding | 3/3 |
|  | exon 15 | RP23-185A18.9 *(Novel)* | [2610507B11Rik](http://www.informatics.jax.org/searches/accession_report.cgi?id=MGI%3A1919753) | [ENSMUSG00000010277](http://www.ensembl.org/mus_musculus/geneview?gene=ENSMUSG00000010277) | AGCCCTGTTG**C**AAGGCTCCTG^F^ | C to A | C toSTOP | 3/3 |
| *l11Jus08^Het^* | intron 36 | Zinc finger, ZZ-type with EF hand domain 1 *(Zzef1)* | 2444286 | ENSMUSG00000055670 | CTGTGTGGTT**T**GTGTAACCCC^F^ | T to A | noncoding | 3/3 |
| *l11Jus12^Het^* | upstream | F-box and leucine-rich repeat protein 20 *(Fbxl20)* | 1919444 | ENSMUSG00000020883 | CCAGCCCCTC**C**CCCGGCCCAA^R^ | C to T | noncoding | 0/4 |
|  | exon 16 | Mitogen-activated protein kinase kinase kinase 14 *(Map3k14)* | 1858204 | ENSMUSG00000020941 | TTGCTATGAC**A**TGGAGGTGCC^R^ | A to T | M to L | 3/3 |
|  | intron 5 | P130Cas-associated protein *(P140)* | 1933179 | ENSMUSG00000038453 | TCCTGAGGGC**T**CACCACACTC^R^ | T to C | noncoding | 4/4 |
|  | exon 19 | Tousled-like kinase 2 *(Tlk2)* | 1346023 | ENSMUSG00000020694 | TCATGGATGA**T**GATAGCTACA^F^ | T to C | D to D | 4/4 |
| *l11Jus13^Hom^* | intron 1 | Mitochondrial ribosomal protein L27 *(Mrpl27)* | 2137224 | ENSMUSG00000024414 | AGACACTGCT**C**AGAGTACCAG^F^ | C to A | noncoding | 3/3 |
|  | intron 12 | Neighbor of Brca1 gene 1 *(Nbr1)* | 108498 | ENSMUSG00000017119 | GATAATTCAG**A**CTTTTCTGGA^F^ | A to G | noncoding | 3/3 |
|  | intron 6 | Nucleoredoxin *(Nxn)* | 109331 | ENSMUSG00000020844 | TTTTTGTAGG**T**ATGGAGCTGG^R^ | T to A | noncoding | 3/3 |
|  | downstream | RP23-396N4.2 *(Novel)* | [OTTMUSG00000002987](http://www.informatics.jax.org/searches/accession_report.cgi?id=MGI%3A3651790) | ENSMUSG00000040838 | ATAACCACTG**T**CATCCAGTTT^F^ | T to A | noncoding | 3/3 |
|  | 3' UTR | Max-like protein X (*Mlx*) | 108398 | ENSMUSG00000017801 | ACCGGTCAGC**T**GGTTTCTACT^F^ | T to C | noncoding | 3/3 |
| *l11Jus14^Het^* | intron 14 | Signal transducer and activator of transcription 3 *(Stat3)* | 103038 | ENSMUSG00000004040 | GCAGAGTTGT**G**TCCCTGCCAG^R^ | G to T | noncoding | 2/2 |
| *l11Jus15^Het^* | 5' UTR | Benzodiazapine receptor associated protein 1 *(Bzrap1)* | 2450877 | ENSMUSG00000034156 | GGAACTCCTG**C**GGACCCTTTC^F^ | C to A | noncoding | B6/129 SNP |
|  | 5' UTR | Carbonic anhydrase 10 *(Car10)* | 1919855 | ENSMUSG00000056158 | TGCCGAAACA**A**TTCAAACTGC^F^ | A to G | noncoding | 3/3 |
|  | exon 4 | Mediator of RNA polymerase II transcription, subunit 31 homolog *(Med31)* | 1914529 | ENSMUSG00000020801 | TCTGCACTGG**C**AGCACTACTC^R^ | C to T | Q to stop | 3/3 |
| *l11Jus22^Het^* | intron 4 | Membrane protein palmitoylated 2 *(Mpp2)* | 1858257 | ENSMUSG00000017314 | TTCCGGTCCC**T**CCTTTAGTCC^R^ | T to A | noncoding | 0/4 |
|  | exon 8 | Serine carboxypeptidase 1 *(Scpep1)* | 1921867 | ENSMUSG00000000278 | GCAGAGCAAG**T**CCTTGATGCT^R^ | T to C | V to A | 3/3 |
|  | intron 16 | Signal transducer and activator of transcription 5A *(Stat5a)* | 103036 | ENSMUSG00000004043 | AAGCAGTTCT**T**CCTAAAATCC^F^ | T to C | noncoding | 3/3 |
| *l11Jus27^Het^* | upstream | F-box and leucine-rich repeat protein 20 *(Fbxl20)* | 1919444 | ENSMUSG00000020883 | CCGCCCCCAC**C**CGATTGACGC^R^ | C to T | noncoding | N/A^3^ |
|  | exon 8 | DEXH (Asp-Glu-X-His) box polypeptide 58 *(Dhx58 )* | 1931560 | ENSMUSG00000017830 | AGTTTGGGAG**T**CCTGGCCACA^R^ | T to C | S to S | 3/3 |
|  | intron 31 | Ubiquitin specific protease 32 *(Usp32)* | 2144475 | ENSMUSG00000000804 | GGCTTCCATC**T**CTATTTCAGC^R^ | T to C | noncoding | 3/3 |
| *l11Jus38^Het^* | 3' UTR | Microtubule-associated protein tau *(Mapt)* | 97180 | ENSMUSG00000018411 | CAAAAATCAT**G**ATTTGGAGTG^F^ | G to T | noncoding | B6/C3H SNP |
| *l11Jus39^Het^* | intron 2 | RP23-350G1.1 *(Novel)* | 5530401A14Rik | [ENSMUST00000021044](http://www.ensembl.org/Mus_musculus/transview?transcript=ENSMUST00000021044) | AAGGGAATAA**A**GTAGAGAGAG^F^ | A to G | noncoding | 2/3 |
| *l11Jus45^Het^* | intron 2 | RNA methyltransferase like 1 *(Rnmtl1)* | 1914640 | ENSMUSG00000038046 | GTAATGCAAG**C**CTTTAAAATT^R^ | C to T | noncoding | B6/129 SNP |
|  | 5' UTR | Cyclin N-terminal domain containing 1 *(Cntd1)* | 1923965 | ENSMUSG00000035112 | AATAAGCTAA**G**GAGGCCAGTT^F^ | G to A | noncoding | 0/2 |
|  | downstream | Solute carrier family 25, member 35 *(Slc25a35)* | 1919248 | ENSMUSG00000018740 | GCCAGAAAGG**G**ACCAGGCATC^F^ | G to A | noncoding | 0/2 |
|  | intron 9 | Seizure related gene 6 *(Sez6)* | 104745 | ENSMUSG00000000632 | TAGTCCCGCC**C**ACCTCGACCC^F^ | C to T | noncoding | 0/2 |
|  | upstream | Sp2 transcription factor *(Sp2)* | 1926162 | ENSMUSG00000018678 | AGAGCTTTAA**G**GGGAGGGGGT^R^ | G to A | noncoding | 0/2 |
| *l11Jus48^Hom^* | exon 2 | Hairy and enhancer of split 7 *(Hes7)* | 2135679 | ENSMUSG00000023781 | GTTGGTGGAG**A**AGCGGCGCCG^F^ | A to G | K to E | 3/3^2^ |
| *l11Jus49^Het^* | intron 32 | Acetyl-Coenzyme A carboxylase alpha *(Acaca)* | 108451 | ENSMUSG00000020532 | CTCTACCAGG**T**AACTTCTTCC^F^ | T to C | noncoding | 2/2 |
| *l11Jus51^Hom^* | upstream | G protein pathway suppressor 2 *(Gps2)* | 1891751 | ENSMUSG00000023170 | AAAGCGCCGA**C**AAGGCGGGTT^F^ | C to T | noncoding | 0/3 |
|  | exon 13 | Solute carrier family 4 member 1 *(Slc4a1)* | 109393 | ENSMUSG00000006574 | TACACCCAGG**A**GATCTTCTCC^R^ | A to T | E to V | previously reported^4^ |
| *l11Jus52^Hom^* | 5' UTR | Aryl hydrocarbon receptor-interacting protein-like 1 *(Aipl1)* | 2148800 | ENSMUSG00000040554 | AGGCCTGAAC**A**AACCTCTCCC^R^ | A to T | noncoding | 3/3 |
|  | intron 1 | ATPase, H+ transporting, lysosomal V0 subunit a isoform 1 *(Atp6v0a1)* | 103286 | ENSMUSG00000019302 | ATCAGTCTTA**T**CTATCGATTA^F^ | T to A | noncoding | 3/3 |
|  | exon 1 | Frizzled homolog 2 *(Fzd2)* | 1888513 | ENSMUSG00000050288 | ATGATGCTCT**A**CTTCTTCAGC^F^ | A to G | Y to C | 3/3 |
|  | exon 10 | Plexin domain containing 1 *(Plxdc1)* | 1919574 | ENSMUSG00000017417 | CGATACCGCC**A**AGAATGGCTG^R^ | A to C | Q to P | 3/3 |
|  | intron 9 | Suppressor of cytokine signaling 7 *(Socs7)* | 2651588 | ENSMUSG00000038485 | TGGCTGGCGG**T**CCTCCCTCTC^F^ | T to C | noncoding | 3/3 |
| *l11Jus54^Hom^* | exon 1 | Frizzled homolog 2 *(Fzd2)* | 1888513 | ENSMUSG00000050288 | AGCCAGCACT**G**CAAGAGCCTA^F^ | G to A | C to Y | 3/3 |
| *l11Jus55^Hom^* | 3' UTR | Neurofibromatosis 1 *(Nf1)* | 97306 | ENSMUSG00000020716 | TGTCTGGTTG**A**GCACTTGTTC^F^ | A to G | noncoding | 3/3 |
| *l11Jus58^Het^* | intron 11 | Rho GTPase activating protein 23 *(Arhgap23)* | 3697726 | ENSMUSG00000049807 | AGGGGGAGAG**G**GGCTGCCTCC^F^ | G to A | noncoding | 0/2 |
|  | intron 19 | Contactin associated protein 1 *(Cntnap1)* | 1858201 | ENSMUSG00000017167 | ATGCAAAGAT**C**CAGTCCTTTA^F^ | C to T | noncoding | 3/3 |
|  | exon 2 | Mitochondrial ribosomal protein S23 *(Mrps23)* | 1928138 | ENSMUSG00000023723 | ACATATATAA**G**GCCTTTCCAC^F^ | G to A | K to K | 3/3 |
|  | 5' UTR | RP23-96I9.2 (Novel) | [1700016K19Rik](http://www.informatics.jax.org/searches/accession_report.cgi?id=MGI%3A1921480) | ENSMUSG00000053783 | GCGCCTGGAG**C**CCGCCAGGTG^F^ | C to T | noncoding | 0/3 |
| *nur01^Hom^* | no mutations |  |  |  |  |  |  |  |
| *nur05^Hom^* | no mutations |  |  |  |  |  |  |  |
| *nur07^Hom^* | exon 4 | Aspartoacylase (aminoacylase) 2 *(Aspa)* | 87914 | ENSMUSG00000020774 | TATTTTAGAC**C**AAATGAGAAA^F^ | C to T | Q to stop | previously reported^1,5^ |
|  | exon 3 | RP23-352L3.2 (Novel) | [1200011M11Rik](http://www.informatics.jax.org/searches/accession_report.cgi?id=MGI%3A1921383) | ENSMUSG00000020495 | TGGTTCCTGA**T**GCTCCTTTGC^R^ | T to A | D to E | 3/3 |
|  | 3' UTR | RP23-467J12.1(Novel) | [0610013E23Rik](http://www.informatics.jax.org/searches/accession_report.cgi?id=MGI%3A1924142) | ENSMUSG00000020521 | ATTGTTGATT**A**TTAATATAAG^F^ | A to G | noncoding | 3/3 |
| *nur08^Hom^* | intron 6 | RP23-185A18.9 (Novel) | [2610507B11Rik](http://www.informatics.jax.org/searches/accession_report.cgi?id=MGI%3A1919753) | ENSMUSG00000010277 | CATTCCAATT**T**GATCTAAAAC^F^ | T to A | noncoding | 3/3 |
| *nur09^Hom^* | 3' UTR | RP23-136D4.2 (Novel) | [1700081L11Rik](http://www.informatics.jax.org/searches/accession_report.cgi?id=MGI%3A1923969) | ENSMUSG00000018412 | TTTCTTGTTC**T**GTTTTCATGT^R^ | T to A | noncoding | 3/3 |
| *skc1^Hom^* | no mutations |  |  |  |  |  |  |  |
