# Supplementary material for: Discovery of Candidate Disease Genes in ENU–Induced Mouse Mutants by Large-Scale Sequencing, Including a Splice-Site Mutation in Nucleoredoxin
Source: PLoS Genet. 2009 Dec 11;5(12):e1000759. doi: 10.1371/journal.pgen.1000759 (PMC2782131; doi:10.1371/journal.pgen.1000759)
Supplement: Table S5 — Allelic series found. Exon number, intron numbers, and the size of the genomic loci are taken from Ensembl version 34. 1 Four additional lesions found in Aipl1, Atp6v0a1,Plxdc1, and Socs7. 2 One additional lesion found in Tmigd1. 3 Two additional lesions found in Plekhm1, RP23-263M10.5. 4 One additional lesion found in Olfr394. 5 Three additional lesions found in Centb1, Gip, Mpdu1. (0.05 MB DOC) [file pgen.1000759.s007.doc]

Table S5: Allelic series found

| **Gene name** | **Size of Genomic Locus** | **Location of Mutation** | **Base Change** | **Type of Lesion** | **Confirmation** | **Mutant Line** |
| --- | --- | --- | --- | --- | --- | --- |
| *Fzd2* | 3521 bp | exon 1 | A to G | Y to C | 3/3 | *l11Jus52^1^* |
|  |  | exon 1 | G to A | C to Y | 3/3 | *l11Jus54* |
| *RP23-185A18.9* | 28871 bp | exon 15 | C to A | C to stop | 3/3 | *l11Jus06^2^* |
|  |  | intron 6 | T to A | noncoding | 3/3 | *nur08* |
| *RP23-350G1.1* | 33909 bp | intron 2 | G to A | noncoding | 3/3 | *Inf7^3^* |
|  |  | intron 2 | A to G | noncoding | 2/3 | *l11Jus39* |
| *Med13* | 90569 bp | exon 10 | A to G | K to E | 2/2 | *crf08^4^* |
|  |  | 3' UTR | C to A | noncoding | 3/3 | *crf12^5^* |
|  |  | 3' UTR | T to A | noncoding | 2/2 | *l11Jus05* |
