# Supplementary material for: Discovery of Candidate Disease Genes in ENU–Induced Mouse Mutants by Large-Scale Sequencing, Including a Splice-Site Mutation in Nucleoredoxin
Source: PLoS Genet. 2009 Dec 11;5(12):e1000759. doi: 10.1371/journal.pgen.1000759 (PMC2782131; doi:10.1371/journal.pgen.1000759)
Supplement: Table S6 — Known genes associated with mutant alleles that lie in the Trp53-Wnt3 interval. (1.05 MB DOC) [file pgen.1000759.s008.doc]

| **Table S6: Known genes associated with mutant alleles that lie in the Trp53-Wnt3 interval** | |  |
| --- | --- | --- |
|  |  |  |
| **Gene Name** | **Type of Mutation** | **Phenotype?** |
| [Aarsd1, alanyl-tRNA synthetase domain containing 1](http://www.informatics.jax.org/javawi2/servlet/WIFetch?page=markerDetail&key=53664) |  |  |
| [Aatf, apoptosis antagonizing transcription factor](http://www.informatics.jax.org/javawi2/servlet/WIFetch?page=markerDetail&key=49781) | gene trap | yes |
| [Abcc3, ATP-binding cassette, sub-family C (CFTR/MRP), member 3](http://www.informatics.jax.org/javawi2/servlet/WIFetch?page=markerDetail&key=60388) | targeted knockout | yes |
| [Abi3, ABI gene family, member 3](http://www.informatics.jax.org/javawi2/servlet/WIFetch?page=markerDetail&key=50590) | targeted knockout | Cell line only |
| [Abr, active BCR-related gene](http://www.informatics.jax.org/javawi2/servlet/WIFetch?page=markerDetail&key=29080) | targeted knockout | yes |
| [Acaca, acetyl-Coenzyme A carboxylase alpha](http://www.informatics.jax.org/javawi2/servlet/WIFetch?page=markerDetail&key=30040) | targeted knockout | yes |
| [Acadvl, acyl-Coenzyme A dehydrogenase, very long chain](http://www.informatics.jax.org/javawi2/servlet/WIFetch?page=markerDetail&key=33032) | targeted knockout | yes |
| [Acap1, ArfGAP with coiled-coil, ankyrin repeat and PH domains 1](http://www.informatics.jax.org/javawi2/servlet/WIFetch?page=markerDetail&key=83023) |  |  |
| [Acbd4, acyl-Coenzyme A binding domain containing 4](http://www.informatics.jax.org/javawi2/servlet/WIFetch?page=markerDetail&key=51111) |  |  |
| [Accn1, amiloride-sensitive cation channel 1, neuronal (degenerin)](http://www.informatics.jax.org/javawi2/servlet/WIFetch?page=markerDetail&key=34662) | targeted knockout | yes |
| [Acly, ATP citrate lyase](http://www.informatics.jax.org/javawi2/servlet/WIFetch?page=markerDetail&key=23214) | gene trap | yes |
| [Acsf2, acyl-CoA synthetase family member 2](http://www.informatics.jax.org/javawi2/servlet/WIFetch?page=markerDetail&key=91604) |  |  |
| [Adam11, a disintegrin and metallopeptidase domain 11](http://www.informatics.jax.org/javawi2/servlet/WIFetch?page=markerDetail&key=40297) | targeted knockout | yes |
| [Adap2, ArfGAP with dual PH domains 2](http://www.informatics.jax.org/javawi2/servlet/WIFetch?page=markerDetail&key=90751) |  |  |
| [Aipl1, aryl hydrocarbon receptor-interacting protein-like 1](http://www.informatics.jax.org/javawi2/servlet/WIFetch?page=markerDetail&key=75799) | targeted knockout | yes |
| [Akap1, A kinase (PRKA) anchor protein 1](http://www.informatics.jax.org/javawi2/servlet/WIFetch?page=markerDetail&key=24967) | targeted knockout | yes |
| [Alcp2, alcohol preference locus 2, female specific](http://www.informatics.jax.org/javawi2/servlet/WIFetch?page=markerDetail&key=28595) | QTL |  |
| [Aldoc, aldolase C, fructose-bisphosphate](http://www.informatics.jax.org/javawi2/servlet/WIFetch?page=markerDetail&key=17734) |  |  |
| [Aliq1, acute lung injury QTL 1](http://www.informatics.jax.org/javawi2/servlet/WIFetch?page=markerDetail&key=42548) | QTL |  |
| [Alox12, arachidonate 12-lipoxygenase](http://www.informatics.jax.org/javawi2/servlet/WIFetch?page=markerDetail&key=36620) | targeted knockout | yes |
| [Alox12e, arachidonate lipoxygenase, epidermal](http://www.informatics.jax.org/javawi2/servlet/WIFetch?page=markerDetail&key=36630) |  |  |
| [Alox15, arachidonate 15-lipoxygenase](http://www.informatics.jax.org/javawi2/servlet/WIFetch?page=markerDetail&key=36616) | targeted knockout | yes |
| [Amac1, acyl-malonyl condensing enzyme 1](http://www.informatics.jax.org/javawi2/servlet/WIFetch?page=markerDetail&key=48740) |  |  |
| [Ankfn1, ankyrin-repeat and fibronectin type III domain containing 1](http://www.informatics.jax.org/javawi2/servlet/WIFetch?page=markerDetail&key=98393) |  |  |
| [Ankfy1, ankyrin repeat and FYVE domain containing 1](http://www.informatics.jax.org/javawi2/servlet/WIFetch?page=markerDetail&key=40458) |  |  |
| [Ankrd13b, ankyrin repeat domain 13b](http://www.informatics.jax.org/javawi2/servlet/WIFetch?page=markerDetail&key=71941) |  |  |
| [Ankrd40, ankyrin repeat domain 40](http://www.informatics.jax.org/javawi2/servlet/WIFetch?page=markerDetail&key=55432) |  |  |
| [Aoc2, amine oxidase, copper containing 2 (retina-specific)](http://www.informatics.jax.org/javawi2/servlet/WIFetch?page=markerDetail&key=91771) |  |  |
| [Aoc3, amine oxidase, copper containing 3](http://www.informatics.jax.org/javawi2/servlet/WIFetch?page=markerDetail&key=37893) | targeted knockout | yes |
| [Ap1gbp1, AP1 gamma subunit binding protein 1](http://www.informatics.jax.org/javawi2/servlet/WIFetch?page=markerDetail&key=46009) |  |  |
| [Ap2b1, adaptor-related protein complex 2, beta 1 subunit](http://www.informatics.jax.org/javawi2/servlet/WIFetch?page=markerDetail&key=55750) | targeted knockout | Cell line only |
| [Appbp2, amyloid beta precursor protein (cytoplasmic tail) binding protein 2](http://www.informatics.jax.org/javawi2/servlet/WIFetch?page=markerDetail&key=50864) |  |  |
| [Arhgap23, Rho GTPase activating protein 23](http://www.informatics.jax.org/javawi2/servlet/WIFetch?page=markerDetail&key=306678) |  |  |
| [Arhgap27, Rho GTPase activating protein 27](http://www.informatics.jax.org/javawi2/servlet/WIFetch?page=markerDetail&key=53633) |  |  |
| [Arl4d, ADP-ribosylation factor-like 4D](http://www.informatics.jax.org/javawi2/servlet/WIFetch?page=markerDetail&key=75873) | gene trap | Cell line only |
| [Arl5c, ADP-ribosylation factor-like 5C](http://www.informatics.jax.org/javawi2/servlet/WIFetch?page=markerDetail&key=105294) |  |  |
| [Arrb2, arrestin, beta 2](http://www.informatics.jax.org/javawi2/servlet/WIFetch?page=markerDetail&key=15286) | targeted knockout | yes |
| [Asb16, ankyrin repeat and SOCS box-containing 16](http://www.informatics.jax.org/javawi2/servlet/WIFetch?page=markerDetail&key=88487) |  |  |
| [Asgr1, asialoglycoprotein receptor 1](http://www.informatics.jax.org/javawi2/servlet/WIFetch?page=markerDetail&key=419) | targeted knockout | yes |
| [Asgr2, asialoglycoprotein receptor 2](http://www.informatics.jax.org/javawi2/servlet/WIFetch?page=markerDetail&key=420) | targeted knockout | yes |
| [Aspa, aspartoacylase](http://www.informatics.jax.org/javawi2/servlet/WIFetch?page=markerDetail&key=103) | ENU, targeted knockout | yes |
| [Atad4, ATPase family, AAA domain containing 4](http://www.informatics.jax.org/javawi2/servlet/WIFetch?page=markerDetail&key=82636) |  |  |
| [Atad5, ATPase family, AAA domain containing 5](http://www.informatics.jax.org/javawi2/servlet/WIFetch?page=markerDetail&key=84103) |  |  |
| [Atp1b2, ATPase, Na+/K+ transporting, beta 2 polypeptide](http://www.informatics.jax.org/javawi2/servlet/WIFetch?page=markerDetail&key=463) | targeted knockout, targeted mutation | yes |
| [Atp2a3, ATPase, Ca++ transporting, ubiquitous](http://www.informatics.jax.org/javawi2/servlet/WIFetch?page=markerDetail&key=34832) | targeted knockout | yes |
| [Atp5g1, ATP synthase, H+ transporting, mitochondrial F0 complex, subunit c (subunit 9), isoform 1](http://www.informatics.jax.org/javawi2/servlet/WIFetch?page=markerDetail&key=28925) |  |  |
| [Atp5l2, ATP synthase, H+ transporting, mitochondrial FO complex, subunit G2, pseudogene](http://www.informatics.jax.org/javawi2/servlet/WIFetch?page=markerDetail&key=198923) |  |  |
| [Atp6v0a1, ATPase, H+ transporting, lysosomal V0 subunit A1](http://www.informatics.jax.org/javawi2/servlet/WIFetch?page=markerDetail&key=23266) |  |  |
| [Atxn7l3, ataxin 7-like 3](http://www.informatics.jax.org/javawi2/servlet/WIFetch?page=markerDetail&key=104576) |  |  |
| [Azdm5, Alzheimer's disease modifier 5](http://www.informatics.jax.org/javawi2/servlet/WIFetch?page=markerDetail&key=201956) | QTL |  |
| [B4galnt2, beta-1,4-N-acetyl-galactosaminyl transferase 2](http://www.informatics.jax.org/javawi2/servlet/WIFetch?page=markerDetail&key=41753) | targeted knockout | Cell line only |
| [Bbaa4, B.burgdorferi-associated arthritis 4](http://www.informatics.jax.org/javawi2/servlet/WIFetch?page=markerDetail&key=75981) | QTL |  |
| [Bcas3, breast carcinoma amplified sequence 3](http://www.informatics.jax.org/javawi2/servlet/WIFetch?page=markerDetail&key=81744) |  |  |
| [Bcl6b, B-cell CLL/lymphoma 6, member B](http://www.informatics.jax.org/javawi2/servlet/WIFetch?page=markerDetail&key=37376) | targeted knockout | yes |
| [Becn1, beclin 1, autophagy related](http://www.informatics.jax.org/javawi2/servlet/WIFetch?page=markerDetail&key=48103) | targeted knockout | yes |
| [Bgeq9, body growth early QTL 9](http://www.informatics.jax.org/javawi2/servlet/WIFetch?page=markerDetail&key=30093) | QTL |  |
| [Bhlha9, basic helix-loop-helix family, member a9](http://www.informatics.jax.org/javawi2/servlet/WIFetch?page=markerDetail&key=85183) | targeted knockout | Cell line only |
| [Blmh, bleomycin hydrolase](http://www.informatics.jax.org/javawi2/servlet/WIFetch?page=markerDetail&key=42396) | targeted knockout | yes |
| [Bomd7, bone mineral density 7](http://www.informatics.jax.org/javawi2/servlet/WIFetch?page=markerDetail&key=155873) | QTL |  |
| [Brca1, breast cancer 1](http://www.informatics.jax.org/javawi2/servlet/WIFetch?page=markerDetail&key=24690) | targeted knockout | yes |
| [Brip1, BRCA1 interacting protein C-terminal helicase 1](http://www.informatics.jax.org/javawi2/servlet/WIFetch?page=markerDetail&key=84031) |  |  |
| [Brwq1, brain weight QTL 1](http://www.informatics.jax.org/javawi2/servlet/WIFetch?page=markerDetail&key=306633) | QTL |  |
| [Bw4, body weight QTL 4](http://www.informatics.jax.org/javawi2/servlet/WIFetch?page=markerDetail&key=38627) | QTL |  |
| [Bwtn3, body weight at necropsy 3](http://www.informatics.jax.org/javawi2/servlet/WIFetch?page=markerDetail&key=185890) | QTL |  |
| [Bzrap1, benzodiazapine receptor associated protein 1](http://www.informatics.jax.org/javawi2/servlet/WIFetch?page=markerDetail&key=87174) |  |  |
| [C10bw3, castaneus 10 week body weight 3](http://www.informatics.jax.org/javawi2/servlet/WIFetch?page=markerDetail&key=65219) |  |  |
| [C1qbp, complement component 1, q subcomponent binding protein](http://www.informatics.jax.org/javawi2/servlet/WIFetch?page=markerDetail&key=34834) | gene trap | Cell line only |
| [C1ql1, complement component 1, q subcomponent-like 1](http://www.informatics.jax.org/javawi2/servlet/WIFetch?page=markerDetail&key=42210) | targeted knockout | Cell line only |
| [Cacna1g, calcium channel, voltage-dependent, T type, alpha 1G subunit](http://www.informatics.jax.org/javawi2/servlet/WIFetch?page=markerDetail&key=35657) | targeted knockout | yes |
| [Cacnb1, calcium channel, voltage-dependent, beta 1 subunit](http://www.informatics.jax.org/javawi2/servlet/WIFetch?page=markerDetail&key=32910) | targeted knockout | yes |
| [Calcoco2, calcium binding and coiled-coil domain 2](http://www.informatics.jax.org/javawi2/servlet/WIFetch?page=markerDetail&key=42002) |  |  |
| [Camkk1, calcium/calmodulin-dependent protein kinase kinase 1, alpha](http://www.informatics.jax.org/javawi2/servlet/WIFetch?page=markerDetail&key=48084) | targeted knockout | yes |
| [Camta2, calmodulin binding transcription activator 2](http://www.informatics.jax.org/javawi2/servlet/WIFetch?page=markerDetail&key=64767) | targeted knockout | yes |
| [Car10, carbonic anhydrase 10](http://www.informatics.jax.org/javawi2/servlet/WIFetch?page=markerDetail&key=56585) |  |  |
| [Car4, carbonic anhydrase 4](http://www.informatics.jax.org/javawi2/servlet/WIFetch?page=markerDetail&key=33482) | targeted knockout | yes |
| [Casc3, cancer susceptibility candidate 3](http://www.informatics.jax.org/javawi2/servlet/WIFetch?page=markerDetail&key=80379) |  |  |
| [Cbx1, chromobox homolog 1 (Drosophila HP1 beta)](http://www.informatics.jax.org/javawi2/servlet/WIFetch?page=markerDetail&key=25867) | gene trap | No phenotype upon observation |
| [Ccdc103, coiled-coil domain containing 103](http://www.informatics.jax.org/javawi2/servlet/WIFetch?page=markerDetail&key=57273) |  |  |
| [Ccdc43, coiled-coil domain containing 43](http://www.informatics.jax.org/javawi2/servlet/WIFetch?page=markerDetail&key=37586) |  |  |
| [Ccdc49, coiled-coil domain containing 49](http://www.informatics.jax.org/javawi2/servlet/WIFetch?page=markerDetail&key=51460) |  |  |
| [Ccdc55, coiled-coil domain containing 55](http://www.informatics.jax.org/javawi2/servlet/WIFetch?page=markerDetail&key=71745) |  |  |
| [Ccdc56, coiled-coil domain containing 56](http://www.informatics.jax.org/javawi2/servlet/WIFetch?page=markerDetail&key=34135) |  |  |
| [Ccl1, chemokine (C-C motif) ligand 1](http://www.informatics.jax.org/javawi2/servlet/WIFetch?page=markerDetail&key=13362) | targeted knockout | yes |
| [Ccl11, chemokine (C-C motif) ligand 11](http://www.informatics.jax.org/javawi2/servlet/WIFetch?page=markerDetail&key=23564) | targeted knockout | yes |
| [Ccl12, chemokine (C-C motif) ligand 12](http://www.informatics.jax.org/javawi2/servlet/WIFetch?page=markerDetail&key=29740) | targeted knockout | Cell line only |
| [Ccl2, chemokine (C-C motif) ligand 2](http://www.informatics.jax.org/javawi2/servlet/WIFetch?page=markerDetail&key=13363) | targeted knockout | yes |
| [Ccl3, chemokine (C-C motif) ligand 3](http://www.informatics.jax.org/javawi2/servlet/WIFetch?page=markerDetail&key=13364) | targeted knockout | yes |
| [Ccl4, chemokine (C-C motif) ligand 4](http://www.informatics.jax.org/javawi2/servlet/WIFetch?page=markerDetail&key=13365) |  |  |
| [Ccl5, chemokine (C-C motif) ligand 5](http://www.informatics.jax.org/javawi2/servlet/WIFetch?page=markerDetail&key=13366) | targeted knockout | yes |
| [Ccl6, chemokine (C-C motif) ligand 6](http://www.informatics.jax.org/javawi2/servlet/WIFetch?page=markerDetail&key=13367) |  |  |
| [Ccl7, chemokine (C-C motif) ligand 7](http://www.informatics.jax.org/javawi2/servlet/WIFetch?page=markerDetail&key=15327) | targeted knockout | yes |
| [Ccl8, chemokine (C-C motif) ligand 8](http://www.informatics.jax.org/javawi2/servlet/WIFetch?page=markerDetail&key=17750) |  |  |
| [Ccl9, chemokine (C-C motif) ligand 9](http://www.informatics.jax.org/javawi2/servlet/WIFetch?page=markerDetail&key=24686) |  |  |
| [Ccr10, chemokine (C-C motif) receptor 10](http://www.informatics.jax.org/javawi2/servlet/WIFetch?page=markerDetail&key=33364) | targeted knockout | yes |
| [Ccr7, chemokine (C-C motif) receptor 7](http://www.informatics.jax.org/javawi2/servlet/WIFetch?page=markerDetail&key=32508) | targeted knockout | yes |
| [Cct6b, chaperonin containing Tcp1, subunit 6b (zeta)](http://www.informatics.jax.org/javawi2/servlet/WIFetch?page=markerDetail&key=39122) |  |  |
| [Cd300lg, CD300 antigen like family member G](http://www.informatics.jax.org/javawi2/servlet/WIFetch?page=markerDetail&key=37436) |  |  |
| [Cd68, CD68 antigen](http://www.informatics.jax.org/javawi2/servlet/WIFetch?page=markerDetail&key=871) |  |  |
| [Cdc34-ps, cell division cycle 34 homolog, pseudogene (S. cerevisiae)](http://www.informatics.jax.org/javawi2/servlet/WIFetch?page=markerDetail&key=46461) |  |  |
| [Cdc6, cell division cycle 6 homolog (S. cerevisiae)](http://www.informatics.jax.org/javawi2/servlet/WIFetch?page=markerDetail&key=42360) |  |  |
| [Cdk5r1, cyclin-dependent kinase 5, regulatory subunit 1 (p35)](http://www.informatics.jax.org/javawi2/servlet/WIFetch?page=markerDetail&key=17615) |  |  |
| [Cdk5rap3, CDK5 regulatory subunit associated protein 3](http://www.informatics.jax.org/javawi2/servlet/WIFetch?page=markerDetail&key=63768) |  |  |
| [Chad, chondroadherin](http://www.informatics.jax.org/javawi2/servlet/WIFetch?page=markerDetail&key=33523) |  |  |
| [Chrnb1, cholinergic receptor, nicotinic, beta polypeptide 1 (muscle)](http://www.informatics.jax.org/javawi2/servlet/WIFetch?page=markerDetail&key=64) | targeted knockout | yes |
| [Chrne, cholinergic receptor, nicotinic, epsilon polypeptide](http://www.informatics.jax.org/javawi2/servlet/WIFetch?page=markerDetail&key=70) | targeted knockout | yes |
| [Cia40, collagen induced arthritis 40](http://www.informatics.jax.org/javawi2/servlet/WIFetch?page=markerDetail&key=171580) | QTL |  |
| [Cisd3, CDGSH iron sulfur domain 3](http://www.informatics.jax.org/javawi2/servlet/WIFetch?page=markerDetail&key=17642) |  |  |
| [Cldn7, claudin 7](http://www.informatics.jax.org/javawi2/servlet/WIFetch?page=markerDetail&key=46705) |  |  |
| [Cltc, clathrin, heavy polypeptide (Hc)](http://www.informatics.jax.org/javawi2/servlet/WIFetch?page=markerDetail&key=82161) |  |  |
| [Cnp, 2',3'-cyclic nucleotide 3' phosphodiesterase](http://www.informatics.jax.org/javawi2/servlet/WIFetch?page=markerDetail&key=1044) | targeted knockout | yes |
| [Cntd1, cyclin N-terminal domain containing 1](http://www.informatics.jax.org/javawi2/servlet/WIFetch?page=markerDetail&key=60695) |  |  |
| [Cntnap1, contactin associated protein-like 1](http://www.informatics.jax.org/javawi2/servlet/WIFetch?page=markerDetail&key=46341) | targeted knockout | yes |
| [Coasy, Coenzyme A synthase](http://www.informatics.jax.org/javawi2/servlet/WIFetch?page=markerDetail&key=55723) |  |  |
| [Coil, coilin](http://www.informatics.jax.org/javawi2/servlet/WIFetch?page=markerDetail&key=25108) | targeted knockout | yes |
| [Col1a1, collagen, type I, alpha 1](http://www.informatics.jax.org/javawi2/servlet/WIFetch?page=markerDetail&key=1092) | targeted knockout, ENU | yes |
| [Copz2, coatomer protein complex, subunit zeta 2](http://www.informatics.jax.org/javawi2/servlet/WIFetch?page=markerDetail&key=49433) |  |  |
| [Coro6, coronin 6](http://www.informatics.jax.org/javawi2/servlet/WIFetch?page=markerDetail&key=89989) |  |  |
| [Cox11, COX11 homolog, cytochrome c oxidase assembly protein (yeast)](http://www.informatics.jax.org/javawi2/servlet/WIFetch?page=markerDetail&key=53782) |  |  |
| [Cpd, carboxypeptidase D](http://www.informatics.jax.org/javawi2/servlet/WIFetch?page=markerDetail&key=28363) |  |  |
| [Crhq1, compensatory renal hypertrophy QTL 1](http://www.informatics.jax.org/javawi2/servlet/WIFetch?page=markerDetail&key=37780) | QTL |  |
| [Crk, v-crk sarcoma virus CT10 oncogene homolog (avian)](http://www.informatics.jax.org/javawi2/servlet/WIFetch?page=markerDetail&key=1154) | targeted knockout, gene trap | yes |
| [Crkrs, CDC2-related kinase, arginine/serine-rich](http://www.informatics.jax.org/javawi2/servlet/WIFetch?page=markerDetail&key=34180) |  |  |
| [Crlf3, cytokine receptor-like factor 3](http://www.informatics.jax.org/javawi2/servlet/WIFetch?page=markerDetail&key=46945) |  |  |
| [Cryba1, crystallin, beta A1](http://www.informatics.jax.org/javawi2/servlet/WIFetch?page=markerDetail&key=1170) | ENU | yes |
| [Csf3, colony stimulating factor 3 (granulocyte)](http://www.informatics.jax.org/javawi2/servlet/WIFetch?page=markerDetail&key=41067) | targeted knockout | yes |
| [Ctns, cystinosis, nephropathic](http://www.informatics.jax.org/javawi2/servlet/WIFetch?page=markerDetail&key=63730) | targeted knockout |  |
| [Cuedc1, CUE domain containing 1](http://www.informatics.jax.org/javawi2/servlet/WIFetch?page=markerDetail&key=71721) |  |  |
| [Cxcl16, chemokine (C-X-C motif) ligand 16](http://www.informatics.jax.org/javawi2/servlet/WIFetch?page=markerDetail&key=64290) | targeted knockout | yes |
| [Cyb5d2, cytochrome b5 domain containing 2](http://www.informatics.jax.org/javawi2/servlet/WIFetch?page=markerDetail&key=97220) | gene trap | Cell line only |
| [Dautb4, dopamine uptake transporter binding 4](http://www.informatics.jax.org/javawi2/servlet/WIFetch?page=markerDetail&key=76640) | QTL |  |
| [Dbil5, diazepam binding inhibitor-like 5](http://www.informatics.jax.org/javawi2/servlet/WIFetch?page=markerDetail&key=29442) |  |  |
| [Dcakd, dephospho-CoA kinase domain containing](http://www.informatics.jax.org/javawi2/servlet/WIFetch?page=markerDetail&key=52067) |  |  |
| [Ddx52, DEAD (Asp-Glu-Ala-Asp) box polypeptide 52](http://www.informatics.jax.org/javawi2/servlet/WIFetch?page=markerDetail&key=62374) |  |  |
| [Derl2, Der1-like domain family, member 2](http://www.informatics.jax.org/javawi2/servlet/WIFetch?page=markerDetail&key=76969) |  |  |
| [Dgke, diacylglycerol kinase, epsilon](http://www.informatics.jax.org/javawi2/servlet/WIFetch?page=markerDetail&key=47683) | targeted knockout | yes |
| [Dhrs11, dehydrogenase/reductase (SDR family) member 11](http://www.informatics.jax.org/javawi2/servlet/WIFetch?page=markerDetail&key=90912) |  |  |
| [Dhrs13, dehydrogenase/reductase (SDR family) member 13](http://www.informatics.jax.org/javawi2/servlet/WIFetch?page=markerDetail&key=54431) |  |  |
| [Dhx33, DEAH (Asp-Glu-Ala-His) box polypeptide 33](http://www.informatics.jax.org/javawi2/servlet/WIFetch?page=markerDetail&key=85933) |  |  |
| [Dhx40, DEAH (Asp-Glu-Ala-His) box polypeptide 40](http://www.informatics.jax.org/javawi2/servlet/WIFetch?page=markerDetail&key=51467) |  |  |
| [Dhx58, DEXH (Asp-Glu-X-His) box polypeptide 58](http://www.informatics.jax.org/javawi2/servlet/WIFetch?page=markerDetail&key=63441) |  |  |
| [Dhx8, DEAH (Asp-Glu-Ala-His) box polypeptide 8](http://www.informatics.jax.org/javawi2/servlet/WIFetch?page=markerDetail&key=37919) |  |  |
| [Dlg4, discs, large homolog 4 (Drosophila)](http://www.informatics.jax.org/javawi2/servlet/WIFetch?page=markerDetail&key=37283) | targeted knockout, gene trap | yes |
| [Dlx3, distal-less homeobox 3](http://www.informatics.jax.org/javawi2/servlet/WIFetch?page=markerDetail&key=7872) | targeted knockout | yes |
| [Dlx4, distal-less homeobox 4](http://www.informatics.jax.org/javawi2/servlet/WIFetch?page=markerDetail&key=7873) | targeted knockout | Cell line only |
| [Dnajc7, DnaJ (Hsp40) homolog, subfamily C, member 7](http://www.informatics.jax.org/javawi2/servlet/WIFetch?page=markerDetail&key=49949) |  |  |
| [Doc2b, double C2, beta](http://www.informatics.jax.org/javawi2/servlet/WIFetch?page=markerDetail&key=34562) |  |  |
| [Dph1, DPH1 homolog (S. cerevisiae)](http://www.informatics.jax.org/javawi2/servlet/WIFetch?page=markerDetail&key=76817) | targeted knockout | yes |
| [Dullard, Dullard homolog (Xenopus laevis)](http://www.informatics.jax.org/javawi2/servlet/WIFetch?page=markerDetail&key=51161) | targeted knockout | Cell line only |
| [Dusp14, dual specificity phosphatase 14](http://www.informatics.jax.org/javawi2/servlet/WIFetch?page=markerDetail&key=48768) |  |  |
| [Dusp3, dual specificity phosphatase 3 (vaccinia virus phosphatase VH1-related)](http://www.informatics.jax.org/javawi2/servlet/WIFetch?page=markerDetail&key=56329) |  |  |
| [Dvl2, dishevelled 2, dsh homolog (Drosophila)](http://www.informatics.jax.org/javawi2/servlet/WIFetch?page=markerDetail&key=27479) | targeted knockout | yes |
| [Dynll2, dynein light chain LC8-type 2](http://www.informatics.jax.org/javawi2/servlet/WIFetch?page=markerDetail&key=52077) |  |  |
| [Eae22, experimental allergic encephalomyelitis susceptibility 22](http://www.informatics.jax.org/javawi2/servlet/WIFetch?page=markerDetail&key=75783) | QTL |  |
| [Eae7, experimental allergic encephalomyelitis susceptibility 7](http://www.informatics.jax.org/javawi2/servlet/WIFetch?page=markerDetail&key=38038) | QTL |  |
| [Efcab5, EF-hand calcium binding domain 5](http://www.informatics.jax.org/javawi2/servlet/WIFetch?page=markerDetail&key=83686) |  |  |
| [Eftud2, elongation factor Tu GTP binding domain containing 2](http://www.informatics.jax.org/javawi2/servlet/WIFetch?page=markerDetail&key=40409) |  |  |
| [Eif1, eukaryotic translation initiation factor 1](http://www.informatics.jax.org/javawi2/servlet/WIFetch?page=markerDetail&key=25572) |  |  |
| [Eif4a1, eukaryotic translation initiation factor 4A1](http://www.informatics.jax.org/javawi2/servlet/WIFetch?page=markerDetail&key=8348) |  |  |
| [Eif5a, eukaryotic translation initiation factor 5A](http://www.informatics.jax.org/javawi2/servlet/WIFetch?page=markerDetail&key=27036) |  |  |
| [Eme1, essential meiotic endonuclease 1 homolog 1 (S. pombe)](http://www.informatics.jax.org/javawi2/servlet/WIFetch?page=markerDetail&key=138123) |  |  |
| [Eno3, enolase 3, beta muscle](http://www.informatics.jax.org/javawi2/servlet/WIFetch?page=markerDetail&key=8519) |  |  |
| [Epn3, epsin 3](http://www.informatics.jax.org/javawi2/servlet/WIFetch?page=markerDetail&key=55869) |  |  |
| [Epx, eosinophil peroxidase](http://www.informatics.jax.org/javawi2/servlet/WIFetch?page=markerDetail&key=28800) | targeted knockout | Cell line only |
| [Eral1, Era (G-protein)-like 1 (E. coli)](http://www.informatics.jax.org/javawi2/servlet/WIFetch?page=markerDetail&key=47693) |  |  |
| [Erbb2, v-erb-b2 erythroblastic leukemia viral oncogene homolog 2, neuro/glioblastoma derived oncogene homolog (avian)](http://www.informatics.jax.org/javawi2/servlet/WIFetch?page=markerDetail&key=8584) | targeted knockout | yes |
| [Estq3, estradiol regulated response QTL 3](http://www.informatics.jax.org/javawi2/servlet/WIFetch?page=markerDetail&key=42750) | QTL |  |
| [Ethm4, ethanol induced thermoregulation 4](http://www.informatics.jax.org/javawi2/servlet/WIFetch?page=markerDetail&key=106197) | QTL |  |
| [Etohcta9, ethanol conditioned taste aversion 9](http://www.informatics.jax.org/javawi2/servlet/WIFetch?page=markerDetail&key=75754) | QTL |  |
| [Etv4, ets variant gene 4 (E1A enhancer binding protein, E1AF)](http://www.informatics.jax.org/javawi2/servlet/WIFetch?page=markerDetail&key=15222) | targeted knockout | yes |
| [Evi2a, ecotropic viral integration site 2a](http://www.informatics.jax.org/javawi2/servlet/WIFetch?page=markerDetail&key=8682) |  |  |
| [Evi2b, ecotropic viral integration site 2b](http://www.informatics.jax.org/javawi2/servlet/WIFetch?page=markerDetail&key=94071) |  |  |
| [Expi, extracellular proteinase inhibitor](http://www.informatics.jax.org/javawi2/servlet/WIFetch?page=markerDetail&key=28681) |  |  |
| [Ezh1, enhancer of zeste homolog 1 (Drosophila)](http://www.informatics.jax.org/javawi2/servlet/WIFetch?page=markerDetail&key=33716) |  |  |
| [Fam101b, family with sequence similarity 101, member B](http://www.informatics.jax.org/javawi2/servlet/WIFetch?page=markerDetail&key=60546) | targeted knockout | No phenotype upon observation |
| [Fam117a, family with sequence similarity 117, memberA](http://www.informatics.jax.org/javawi2/servlet/WIFetch?page=markerDetail&key=72004) |  |  |
| [Fam134c, family with sequence similarity 134, member C](http://www.informatics.jax.org/javawi2/servlet/WIFetch?page=markerDetail&key=51978) |  |  |
| [Fam171a2, family with sequence similarity 171, member A2](http://www.informatics.jax.org/javawi2/servlet/WIFetch?page=markerDetail&key=90844) |  |  |
| [Fam57a, family with sequence similarity 57, member A](http://www.informatics.jax.org/javawi2/servlet/WIFetch?page=markerDetail&key=77153) |  |  |
| [Fbxl20, F-box and leucine-rich repeat protein 20](http://www.informatics.jax.org/javawi2/servlet/WIFetch?page=markerDetail&key=56174) | targeted knockout | yes |
| [Fbxo39, F-box protein 39](http://www.informatics.jax.org/javawi2/servlet/WIFetch?page=markerDetail&key=118421) |  |  |
| [Fbxo47, F-box protein 47](http://www.informatics.jax.org/javawi2/servlet/WIFetch?page=markerDetail&key=56953) |  |  |
| [Feml4, femur length in high growth mice 4](http://www.informatics.jax.org/javawi2/servlet/WIFetch?page=markerDetail&key=306635) | QTL |  |
| [Fgf11, fibroblast growth factor 11](http://www.informatics.jax.org/javawi2/servlet/WIFetch?page=markerDetail&key=30930) | targeted knockout | Cell line only |
| [Fkbp10, FK506 binding protein 10](http://www.informatics.jax.org/javawi2/servlet/WIFetch?page=markerDetail&key=25029) | targeted knockout | Cell line only |
| [Flot2, flotillin 2](http://www.informatics.jax.org/javawi2/servlet/WIFetch?page=markerDetail&key=35938) |  |  |
| [Fmnl1, formin-like 1](http://www.informatics.jax.org/javawi2/servlet/WIFetch?page=markerDetail&key=47609) |  |  |
| [Fndc8, fibronectin type III domain containing 8](http://www.informatics.jax.org/javawi2/servlet/WIFetch?page=markerDetail&key=62899) |  |  |
| [Foxn1, forkhead box N1](http://www.informatics.jax.org/javawi2/servlet/WIFetch?page=markerDetail&key=22844) | targeted knockout | yes |
| [Fxr2, fragile X mental retardation, autosomal homolog 2](http://www.informatics.jax.org/javawi2/servlet/WIFetch?page=markerDetail&key=42728) | targeted knockout | yes |
| [Fzd2, frizzled homolog 2 (Drosophila)](http://www.informatics.jax.org/javawi2/servlet/WIFetch?page=markerDetail&key=47487) |  |  |
| [G6pc, glucose-6-phosphatase, catalytic](http://www.informatics.jax.org/javawi2/servlet/WIFetch?page=markerDetail&key=36537) | targeted knockout | yes |
| [G6pc3, glucose 6 phosphatase, catalytic, 3](http://www.informatics.jax.org/javawi2/servlet/WIFetch?page=markerDetail&key=52381) | targeted knockout | yes |
| [Gabarap, gamma-aminobutyric acid receptor associated protein](http://www.informatics.jax.org/javawi2/servlet/WIFetch?page=markerDetail&key=47347) | gene trap | No phenotype upon observation |
| [Garnl4, GTPase activating RANGAP domain-like 4](http://www.informatics.jax.org/javawi2/servlet/WIFetch?page=markerDetail&key=100699) |  |  |
| [Gas2l2, growth arrest-specific 2 like 2](http://www.informatics.jax.org/javawi2/servlet/WIFetch?page=markerDetail&key=201607) |  |  |
| [Gast, gastrin](http://www.informatics.jax.org/javawi2/servlet/WIFetch?page=markerDetail&key=25028) | targeted knockout | yes |
| [Gdpd1, glycerophosphodiester phosphodiesterase domain containing 1](http://www.informatics.jax.org/javawi2/servlet/WIFetch?page=markerDetail&key=50549) |  |  |
| [Gemin4, gem (nuclear organelle) associated protein 4](http://www.informatics.jax.org/javawi2/servlet/WIFetch?page=markerDetail&key=86637) |  |  |
| [Gfap, glial fibrillary acidic protein](http://www.informatics.jax.org/javawi2/servlet/WIFetch?page=markerDetail&key=9069) | targeted knockout | yes |
| [Ggnbp2, gametogenetin binding protein 2](http://www.informatics.jax.org/javawi2/servlet/WIFetch?page=markerDetail&key=81786) | gene trap | Cell line only |
| [Ggt6, gamma-glutamyltransferase 6](http://www.informatics.jax.org/javawi2/servlet/WIFetch?page=markerDetail&key=55502) | targeted knockout |  |
| [Ghdc, GH3 domain containing](http://www.informatics.jax.org/javawi2/servlet/WIFetch?page=markerDetail&key=63439) |  |  |
| [Gip, gastric inhibitory polypeptide](http://www.informatics.jax.org/javawi2/servlet/WIFetch?page=markerDetail&key=28679) |  |  |
| [Git1, G protein-coupled receptor kinase-interactor 1](http://www.informatics.jax.org/javawi2/servlet/WIFetch?page=markerDetail&key=48750) | gene trap | No phenotype upon observation |
| [Gjc1, gap junction protein, gamma 1](http://www.informatics.jax.org/javawi2/servlet/WIFetch?page=markerDetail&key=9104) | targeted mutation | yes |
| [Gjd3, gap junction protein, delta 3](http://www.informatics.jax.org/javawi2/servlet/WIFetch?page=markerDetail&key=89491) | targeted knockout | yes |
| [Glod4, glyoxalase domain containing 4](http://www.informatics.jax.org/javawi2/servlet/WIFetch?page=markerDetail&key=51181) |  |  |
| [Gltpd2, glycolipid transfer protein domain containing 2](http://www.informatics.jax.org/javawi2/servlet/WIFetch?page=markerDetail&key=85457) |  |  |
| [Gm12, gene model 12, (NCBI)](http://www.informatics.jax.org/javawi2/servlet/WIFetch?page=markerDetail&key=97230) |  |  |
| [Gm1564, gene model 1564, (NCBI)](http://www.informatics.jax.org/javawi2/servlet/WIFetch?page=markerDetail&key=98782) |  |  |
| [Gm525, gene model 525, (NCBI)](http://www.informatics.jax.org/javawi2/servlet/WIFetch?page=markerDetail&key=97743) |  |  |
| [Gm53, gene model 53, (NCBI)](http://www.informatics.jax.org/javawi2/servlet/WIFetch?page=markerDetail&key=97271) |  |  |
| [Gm884, gene model 884, (NCBI)](http://www.informatics.jax.org/javawi2/servlet/WIFetch?page=markerDetail&key=98102) |  |  |
| [Gngt2, guanine nucleotide binding protein (G protein), gamma transducing activity polypeptide 2](http://www.informatics.jax.org/javawi2/servlet/WIFetch?page=markerDetail&key=32590) |  |  |
| [Gosr1, golgi SNAP receptor complex member 1](http://www.informatics.jax.org/javawi2/servlet/WIFetch?page=markerDetail&key=46400) |  |  |
| [Gosr2, golgi SNAP receptor complex member 2](http://www.informatics.jax.org/javawi2/servlet/WIFetch?page=markerDetail&key=48796) |  |  |
| [Gp1ba, glycoprotein 1b, alpha polypeptide](http://www.informatics.jax.org/javawi2/servlet/WIFetch?page=markerDetail&key=39791) | targeted knockout | yes |
| [Gpatch8, G patch domain containing 8](http://www.informatics.jax.org/javawi2/servlet/WIFetch?page=markerDetail&key=55397) |  |  |
| [Gpr179, G protein-coupled receptor 179](http://www.informatics.jax.org/javawi2/servlet/WIFetch?page=markerDetail&key=84521) |  |  |
| [Gps2, G protein pathway suppressor 2](http://www.informatics.jax.org/javawi2/servlet/WIFetch?page=markerDetail&key=48075) |  |  |
| [Grb7, growth factor receptor bound protein 7](http://www.informatics.jax.org/javawi2/servlet/WIFetch?page=markerDetail&key=18635) |  |  |
| [Grn, granulin](http://www.informatics.jax.org/javawi2/servlet/WIFetch?page=markerDetail&key=9286) | targeted knockout | yes |
| [Gsdma, gasdermin A](http://www.informatics.jax.org/javawi2/servlet/WIFetch?page=markerDetail&key=47760) |  |  |
| [Gsdma2, gasdermin A2](http://www.informatics.jax.org/javawi2/servlet/WIFetch?page=markerDetail&key=58220) |  |  |
| [Gsdma3, gasdermin A3](http://www.informatics.jax.org/javawi2/servlet/WIFetch?page=markerDetail&key=118516) | ENU | yes |
| [Gsg2, germ cell-specific gene 2](http://www.informatics.jax.org/javawi2/servlet/WIFetch?page=markerDetail&key=34827) |  |  |
| [Hap1, huntingtin-associated protein 1](http://www.informatics.jax.org/javawi2/servlet/WIFetch?page=markerDetail&key=36292) | targeted knockout | yes |
| [Hcrt, hypocretin](http://www.informatics.jax.org/javawi2/servlet/WIFetch?page=markerDetail&key=35754) | targeted knockout | yes |
| [Hdac5, histone deacetylase 5](http://www.informatics.jax.org/javawi2/servlet/WIFetch?page=markerDetail&key=39831) | targeted mutation | yes |
| [Heal10, wound healing/regeneration 10](http://www.informatics.jax.org/javawi2/servlet/WIFetch?page=markerDetail&key=92014) | QTL |  |
| [Heatr6, HEAT repeat containing 6](http://www.informatics.jax.org/javawi2/servlet/WIFetch?page=markerDetail&key=56520) |  |  |
| [Hexim1, hexamethylene bis-acetamide inducible 1](http://www.informatics.jax.org/javawi2/servlet/WIFetch?page=markerDetail&key=81506) | gene trap | yes |
| [Hexim2, hexamthylene bis-acetamide inducible 2](http://www.informatics.jax.org/javawi2/servlet/WIFetch?page=markerDetail&key=55039) |  |  |
| [Hic1, hypermethylated in cancer 1](http://www.informatics.jax.org/javawi2/servlet/WIFetch?page=markerDetail&key=40699) | targeted mutation | yes |
| [Higd1b, HIG1 domain family, member 1B](http://www.informatics.jax.org/javawi2/servlet/WIFetch?page=markerDetail&key=59669) |  |  |
| [Hils1, histone H1-like protein in spermatids 1](http://www.informatics.jax.org/javawi2/servlet/WIFetch?page=markerDetail&key=75641) | targeted knockout | Cell line only |
| [Hlf, hepatic leukemia factor](http://www.informatics.jax.org/javawi2/servlet/WIFetch?page=markerDetail&key=9756) | targeted mutation | yes |
| [Hnf1b, HNF1 homeobox B](http://www.informatics.jax.org/javawi2/servlet/WIFetch?page=markerDetail&key=13786) | targeted knockout, targeted mutation | yes |
| [Hoxb1, homeo box B1](http://www.informatics.jax.org/javawi2/servlet/WIFetch?page=markerDetail&key=9897) | targeted knockout, targeted mutation | yes |
| [Hoxb13, homeo box B13](http://www.informatics.jax.org/javawi2/servlet/WIFetch?page=markerDetail&key=29027) | targeted knockout, targeted mutation | yes |
| [Hoxb2, homeo box B2](http://www.informatics.jax.org/javawi2/servlet/WIFetch?page=markerDetail&key=9898) | targeted knockout, targeted mutation | yes |
| [Hoxb3, homeo box B3](http://www.informatics.jax.org/javawi2/servlet/WIFetch?page=markerDetail&key=9899) | targeted knockout | yes |
| [Hoxb4, homeo box B4](http://www.informatics.jax.org/javawi2/servlet/WIFetch?page=markerDetail&key=9900) | targeted knockout, targeted mutation | yes |
| [Hoxb5, homeo box B5](http://www.informatics.jax.org/javawi2/servlet/WIFetch?page=markerDetail&key=9901) | targeted knockout, targeted mutation | yes |
| [Hoxb6, homeo box B6](http://www.informatics.jax.org/javawi2/servlet/WIFetch?page=markerDetail&key=9902) | targeted knockout | yes |
| [Hoxb7, homeo box B7](http://www.informatics.jax.org/javawi2/servlet/WIFetch?page=markerDetail&key=9903) | targeted knockout | yes |
| [Hoxb8, homeo box B8](http://www.informatics.jax.org/javawi2/servlet/WIFetch?page=markerDetail&key=9904) | targeted knockout, targeted mutation | yes |
| [Hoxb9, homeo box B9](http://www.informatics.jax.org/javawi2/servlet/WIFetch?page=markerDetail&key=9905) | targeted knockout | yes |
| [Hsd17b1, hydroxysteroid (17-beta) dehydrogenase 1](http://www.informatics.jax.org/javawi2/servlet/WIFetch?page=markerDetail&key=25496) | targeted knockout | Cell line only |
| [Hsf5, heat shock transcription factor family member 5](http://www.informatics.jax.org/javawi2/servlet/WIFetch?page=markerDetail&key=97957) |  |  |
| [Hspb9, heat shock protein, alpha-crystallin-related, B9](http://www.informatics.jax.org/javawi2/servlet/WIFetch?page=markerDetail&key=59462) |  |  |
| [Idd4, insulin dependent diabetes susceptibility 4](http://www.informatics.jax.org/javawi2/servlet/WIFetch?page=markerDetail&key=10187) | QTL |  |
| [Ifi35, interferon-induced protein 35](http://www.informatics.jax.org/javawi2/servlet/WIFetch?page=markerDetail&key=54090) |  |  |
| [Ift20, intraflagellar transport 20 homolog (Chlamydomonas)](http://www.informatics.jax.org/javawi2/servlet/WIFetch?page=markerDetail&key=52315) | targeted knockout, targeted mutation | yes |
| [Igf2bp1, insulin-like growth factor 2 mRNA binding protein 1](http://www.informatics.jax.org/javawi2/servlet/WIFetch?page=markerDetail&key=77940) | gene trap | yes |
| [Igfbp4, insulin-like growth factor binding protein 4](http://www.informatics.jax.org/javawi2/servlet/WIFetch?page=markerDetail&key=10250) | targeted knockout | yes |
| [Ikzf3, IKAROS family zinc finger 3](http://www.informatics.jax.org/javawi2/servlet/WIFetch?page=markerDetail&key=41865) | targeted knockout | yes |
| [Inpp5k, inositol polyphosphate 5-phosphatase K](http://www.informatics.jax.org/javawi2/servlet/WIFetch?page=markerDetail&key=34888) | targeted mutation, gene trap | yes |
| [Ints2, integrator complex subunit 2](http://www.informatics.jax.org/javawi2/servlet/WIFetch?page=markerDetail&key=54402) |  |  |
| [Itga2b, integrin alpha 2b](http://www.informatics.jax.org/javawi2/servlet/WIFetch?page=markerDetail&key=10495) | targeted knockout, targeted mutation | yes |
| [Itga3, integrin alpha 3](http://www.informatics.jax.org/javawi2/servlet/WIFetch?page=markerDetail&key=10496) | targeted knockout, targeted mutation | yes |
| [Itgae, integrin alpha E, epithelial-associated](http://www.informatics.jax.org/javawi2/servlet/WIFetch?page=markerDetail&key=37772) | targeted knockout | yes |
| [Ity2b, immunity to S. typhimurium 2b](http://www.informatics.jax.org/javawi2/servlet/WIFetch?page=markerDetail&key=313819) | QTL |  |
| [Jup, junction plakoglobin](http://www.informatics.jax.org/javawi2/servlet/WIFetch?page=markerDetail&key=10559) | targeted knockout | yes |
| [Kat2a, K(lysine) acetyltransferase 2A](http://www.informatics.jax.org/javawi2/servlet/WIFetch?page=markerDetail&key=41926) | targeted knockout, targeted mutation | yes |
| [Kcnh4, potassium voltage-gated channel, subfamily H (eag-related), member 4](http://www.informatics.jax.org/javawi2/servlet/WIFetch?page=markerDetail&key=78922) |  |  |
| [Kctd11, potassium channel tetramerisation domain containing 11](http://www.informatics.jax.org/javawi2/servlet/WIFetch?page=markerDetail&key=86261) |  |  |
| [Kif18b, kinesin family member 18B](http://www.informatics.jax.org/javawi2/servlet/WIFetch?page=markerDetail&key=82870) |  |  |
| [Kif1c, kinesin family member 1C](http://www.informatics.jax.org/javawi2/servlet/WIFetch?page=markerDetail&key=33844) | targeted mutation | No phenotype upon observation |
| [Kif2b, kinesin family member 2B](http://www.informatics.jax.org/javawi2/servlet/WIFetch?page=markerDetail&key=57450) | targeted knockout | Cell line only |
| [Klhl10, kelch-like 10 (Drosophila)](http://www.informatics.jax.org/javawi2/servlet/WIFetch?page=markerDetail&key=80663) | targeted knockout | yes |
| [Klhl11, kelch-like 11 (Drosophila)](http://www.informatics.jax.org/javawi2/servlet/WIFetch?page=markerDetail&key=92774) | targeted knockout | Cell line only |
| [Kpnb1, karyopherin (importin) beta 1](http://www.informatics.jax.org/javawi2/servlet/WIFetch?page=markerDetail&key=28707) | gene trap | yes |
| [Krt10, keratin 10](http://www.informatics.jax.org/javawi2/servlet/WIFetch?page=markerDetail&key=10655) | targeted knockout, targeted mutation | yes |
| [Krt12, keratin 12](http://www.informatics.jax.org/javawi2/servlet/WIFetch?page=markerDetail&key=10657) | targeted knockout, targeted mutation | yes |
| [Krt13, keratin 13](http://www.informatics.jax.org/javawi2/servlet/WIFetch?page=markerDetail&key=17802) |  |  |
| [Krt14, keratin 14](http://www.informatics.jax.org/javawi2/servlet/WIFetch?page=markerDetail&key=10659) | targeted knockout, targeted mutation | yes |
| [Krt15, keratin 15](http://www.informatics.jax.org/javawi2/servlet/WIFetch?page=markerDetail&key=10660) |  |  |
| [Krt16, keratin 16](http://www.informatics.jax.org/javawi2/servlet/WIFetch?page=markerDetail&key=10661) | targeted knockout | Cell line only |
| [Krt17, keratin 17](http://www.informatics.jax.org/javawi2/servlet/WIFetch?page=markerDetail&key=10662) | targeted knockout | yes |
| [Krt19, keratin 19](http://www.informatics.jax.org/javawi2/servlet/WIFetch?page=markerDetail&key=10664) | targeted mutation | yes |
| [Krt20, keratin 20](http://www.informatics.jax.org/javawi2/servlet/WIFetch?page=markerDetail&key=50789) |  |  |
| [Krt222, keratin 222](http://www.informatics.jax.org/javawi2/servlet/WIFetch?page=markerDetail&key=83936) |  |  |
| [Krt23, keratin 23](http://www.informatics.jax.org/javawi2/servlet/WIFetch?page=markerDetail&key=75883) |  |  |
| [Krt24, keratin 24](http://www.informatics.jax.org/javawi2/servlet/WIFetch?page=markerDetail&key=59686) |  |  |
| [Krt25, keratin 25](http://www.informatics.jax.org/javawi2/servlet/WIFetch?page=markerDetail&key=54790) | ENU | yes |
| [Krt26, keratin 26](http://www.informatics.jax.org/javawi2/servlet/WIFetch?page=markerDetail&key=85776) |  |  |
| [Krt27, keratin 27](http://www.informatics.jax.org/javawi2/servlet/WIFetch?page=markerDetail&key=41155) | radiation induced mutation | yes |
| [Krt28, keratin 28](http://www.informatics.jax.org/javawi2/servlet/WIFetch?page=markerDetail&key=54823) |  |  |
| [Krt31, keratin 31](http://www.informatics.jax.org/javawi2/servlet/WIFetch?page=markerDetail&key=38112) |  |  |
| [Krt32, keratin 32](http://www.informatics.jax.org/javawi2/servlet/WIFetch?page=markerDetail&key=38114) |  |  |
| [Krt33a, keratin 33A](http://www.informatics.jax.org/javawi2/servlet/WIFetch?page=markerDetail&key=55868) | ENU | yes |
| [Krt33b, keratin 33B](http://www.informatics.jax.org/javawi2/servlet/WIFetch?page=markerDetail&key=38110) |  |  |
| [Krt34, keratin 34](http://www.informatics.jax.org/javawi2/servlet/WIFetch?page=markerDetail&key=38113) |  |  |
| [Krt35, keratin 35](http://www.informatics.jax.org/javawi2/servlet/WIFetch?page=markerDetail&key=46580) | targeted knockout | Cell line only |
| [Krt36, keratin 36](http://www.informatics.jax.org/javawi2/servlet/WIFetch?page=markerDetail&key=38103) |  |  |
| [Krt39, keratin 39](http://www.informatics.jax.org/javawi2/servlet/WIFetch?page=markerDetail&key=156436) |  |  |
| [Krt40, keratin 40](http://www.informatics.jax.org/javawi2/servlet/WIFetch?page=markerDetail&key=185648) |  |  |
| [Krt42, keratin 42](http://www.informatics.jax.org/javawi2/servlet/WIFetch?page=markerDetail&key=52219) |  |  |
| [Krt9, keratin 9](http://www.informatics.jax.org/javawi2/servlet/WIFetch?page=markerDetail&key=10667) |  |  |
| [Krtap1-3, keratin associated protein 1-3](http://www.informatics.jax.org/javawi2/servlet/WIFetch?page=markerDetail&key=200002) |  |  |
| [Krtap1-4, keratin associated protein 1-4](http://www.informatics.jax.org/javawi2/servlet/WIFetch?page=markerDetail&key=200788) |  |  |
| [Krtap1-5, keratin associated protein 1-5](http://www.informatics.jax.org/javawi2/servlet/WIFetch?page=markerDetail&key=53644) |  |  |
| [Krtap17-1, keratin associated protein 17-1](http://www.informatics.jax.org/javawi2/servlet/WIFetch?page=markerDetail&key=61894) |  |  |
| [Krtap2-4, keratin associated protein 2-4](http://www.informatics.jax.org/javawi2/servlet/WIFetch?page=markerDetail&key=55433) |  |  |
| [Krtap3-1, keratin associated protein 3-1](http://www.informatics.jax.org/javawi2/servlet/WIFetch?page=markerDetail&key=53453) |  |  |
| [Krtap3-2, keratin associated protein 3-2](http://www.informatics.jax.org/javawi2/servlet/WIFetch?page=markerDetail&key=50688) |  |  |
| [Krtap3-3, keratin associated protein 3-3](http://www.informatics.jax.org/javawi2/servlet/WIFetch?page=markerDetail&key=50360) |  |  |
| [Krtap31-1, keratin associated protein 31-1](http://www.informatics.jax.org/javawi2/servlet/WIFetch?page=markerDetail&key=54811) |  |  |
| [Krtap31-2, keratin associated protein 31-2](http://www.informatics.jax.org/javawi2/servlet/WIFetch?page=markerDetail&key=200348) |  |  |
| [Krtap4-1, keratin associated protein 4-1](http://www.informatics.jax.org/javawi2/servlet/WIFetch?page=markerDetail&key=171428) |  |  |
| [Krtap4-13, keratin associated protein 4-13](http://www.informatics.jax.org/javawi2/servlet/WIFetch?page=markerDetail&key=53444) |  |  |
| [Krtap4-16, keratin associated protein 4-16](http://www.informatics.jax.org/javawi2/servlet/WIFetch?page=markerDetail&key=200589) |  |  |
| [Krtap4-2, keratin associated protein 4-2](http://www.informatics.jax.org/javawi2/servlet/WIFetch?page=markerDetail&key=52653) |  |  |
| [Krtap4-6, keratin associated protein 4-6](http://www.informatics.jax.org/javawi2/servlet/WIFetch?page=markerDetail&key=52748) |  |  |
| [Krtap4-7, keratin associated protein 4-7](http://www.informatics.jax.org/javawi2/servlet/WIFetch?page=markerDetail&key=60424) |  |  |
| [Krtap4-8, keratin associated protein 4-8](http://www.informatics.jax.org/javawi2/servlet/WIFetch?page=markerDetail&key=201865) |  |  |
| [Krtap4-9, keratin associated protein 4-9](http://www.informatics.jax.org/javawi2/servlet/WIFetch?page=markerDetail&key=201619) |  |  |
| [Krtap9-1, keratin associated protein 9-1](http://www.informatics.jax.org/javawi2/servlet/WIFetch?page=markerDetail&key=38116) |  |  |
| [Krtap9-3, keratin associated protein 9-3](http://www.informatics.jax.org/javawi2/servlet/WIFetch?page=markerDetail&key=59566) |  |  |
| [Krtap9-5, keratin associated protein 9-5](http://www.informatics.jax.org/javawi2/servlet/WIFetch?page=markerDetail&key=199892) |  |  |
| [Ksr1, kinase suppressor of ras 1](http://www.informatics.jax.org/javawi2/servlet/WIFetch?page=markerDetail&key=25406) | targeted knockout | yes |
| [Kwq15, kidney weight QTL 15](http://www.informatics.jax.org/javawi2/servlet/WIFetch?page=markerDetail&key=306625) | QTL |  |
| [Lasp1, LIM and SH3 protein 1](http://www.informatics.jax.org/javawi2/servlet/WIFetch?page=markerDetail&key=31990) | targeted knockout | yes |
| [Lgals9, lectin, galactose binding, soluble 9](http://www.informatics.jax.org/javawi2/servlet/WIFetch?page=markerDetail&key=31646) | targeted knockout, gene trap | yes |
| [Lgth6, body length 6](http://www.informatics.jax.org/javawi2/servlet/WIFetch?page=markerDetail&key=89584) | QTL |  |
| [Lhx1, LIM homeobox protein 1](http://www.informatics.jax.org/javawi2/servlet/WIFetch?page=markerDetail&key=15613) | targeted knockout, targeted mutation | yes |
| [Lig3, ligase III, DNA, ATP-dependent](http://www.informatics.jax.org/javawi2/servlet/WIFetch?page=markerDetail&key=30915) | targeted knockout | yes |
| [Lpo, lactoperoxidase](http://www.informatics.jax.org/javawi2/servlet/WIFetch?page=markerDetail&key=60093) |  |  |
| [Lrrc37a, leucine rich repeat containing 37A](http://www.informatics.jax.org/javawi2/servlet/WIFetch?page=markerDetail&key=97469) |  |  |
| [Lrrc46, leucine rich repeat containing 46](http://www.informatics.jax.org/javawi2/servlet/WIFetch?page=markerDetail&key=53277) |  |  |
| [Lrrc59, leucine rich repeat containing 59](http://www.informatics.jax.org/javawi2/servlet/WIFetch?page=markerDetail&key=65573) |  |  |
| [Lsm12, LSM12 homolog (S. cerevisiae)](http://www.informatics.jax.org/javawi2/servlet/WIFetch?page=markerDetail&key=56322) |  |  |
| [Ltxs3, lethal factor toxin susceptibility 3](http://www.informatics.jax.org/javawi2/servlet/WIFetch?page=markerDetail&key=158090) | QTL |  |
| [Lwq9, liver weight QTL 9](http://www.informatics.jax.org/javawi2/servlet/WIFetch?page=markerDetail&key=306634) | QTL |  |
| [Lyzl6, lysozyme-like 6](http://www.informatics.jax.org/javawi2/servlet/WIFetch?page=markerDetail&key=53424) |  |  |
| [Map3k14, mitogen-activated protein kinase kinase kinase 14](http://www.informatics.jax.org/javawi2/servlet/WIFetch?page=markerDetail&key=46344) | targeted knockout, spontaneous | yes |
| [Mbtd1, mbt domain containing 1](http://www.informatics.jax.org/javawi2/servlet/WIFetch?page=markerDetail&key=71417) |  |  |
| [Mdk-ps1, midkine pseudogene 1](http://www.informatics.jax.org/javawi2/servlet/WIFetch?page=markerDetail&key=22931) |  |  |
| [Med1, mediator complex subunit 1](http://www.informatics.jax.org/javawi2/servlet/WIFetch?page=markerDetail&key=34641) | targeted knockout, targeted mutation | yes |
| [Med11, mediator of RNA polymerase II transcription, subunit 11 homolog (S. cerevisiae)](http://www.informatics.jax.org/javawi2/servlet/WIFetch?page=markerDetail&key=50152) |  |  |
| [Med13, mediator complex subunit 13](http://www.informatics.jax.org/javawi2/servlet/WIFetch?page=markerDetail&key=100930) |  |  |
| [Med24, mediator complex subunit 24](http://www.informatics.jax.org/javawi2/servlet/WIFetch?page=markerDetail&key=42195) | targeted mutation | yes |
| [Med31, mediator of RNA polymerase II transcription, subunit 31 homolog (yeast)](http://www.informatics.jax.org/javawi2/servlet/WIFetch?page=markerDetail&key=51259) |  |  |
| [Meox1, mesenchyme homeobox 1](http://www.informatics.jax.org/javawi2/servlet/WIFetch?page=markerDetail&key=23177) | Targeted KO, tarageted other, gene trapped, transgenic | yes |
| [Mett10d, methyltransferase 10 domain containing](http://www.informatics.jax.org/javawi2/servlet/WIFetch?page=markerDetail&key=51473) |  |  |
| [Mgl1, macrophage galactose N-acetyl-galactosamine specific lectin 1](http://www.informatics.jax.org/javawi2/servlet/WIFetch?page=markerDetail&key=11237) | Targeted KO | yes |
| [Mgl2, macrophage galactose N-acetyl-galactosamine specific lectin 2](http://www.informatics.jax.org/javawi2/servlet/WIFetch?page=markerDetail&key=81444) |  |  |
| [Mink1, misshapen-like kinase 1 (zebrafish)](http://www.informatics.jax.org/javawi2/servlet/WIFetch?page=markerDetail&key=46184) | Gene trap | Cell line only |
| [Mir10a, microRNA 10a](http://www.informatics.jax.org/javawi2/servlet/WIFetch?page=markerDetail&key=170812) |  |  |
| [Mir132, microRNA 132](http://www.informatics.jax.org/javawi2/servlet/WIFetch?page=markerDetail&key=93159) |  |  |
| [Mir142, microRNA 142](http://www.informatics.jax.org/javawi2/servlet/WIFetch?page=markerDetail&key=93169) |  |  |
| [Mir144, microRNA 144](http://www.informatics.jax.org/javawi2/servlet/WIFetch?page=markerDetail&key=93171) |  |  |
| [Mir152, microRNA 152](http://www.informatics.jax.org/javawi2/servlet/WIFetch?page=markerDetail&key=93179) |  |  |
| [Mirn193, microRNA 193](http://www.informatics.jax.org/javawi2/servlet/WIFetch?page=markerDetail&key=93225) |  |  |
| [Mirn195, microRNA 195](http://www.informatics.jax.org/javawi2/servlet/WIFetch?page=markerDetail&key=93227) |  |  |
| [Mirn196a-1, microRNA 196a-1](http://www.informatics.jax.org/javawi2/servlet/WIFetch?page=markerDetail&key=93228) |  |  |
| [Mirn21, microRNA 21](http://www.informatics.jax.org/javawi2/servlet/WIFetch?page=markerDetail&key=93192) |  |  |
| [Mirn212, microRNA 212](http://www.informatics.jax.org/javawi2/servlet/WIFetch?page=markerDetail&key=93250) |  |  |
| [Mirn22, microRNA 22](http://www.informatics.jax.org/javawi2/servlet/WIFetch?page=markerDetail&key=93193) |  |  |
| [Mirn301, microRNA 301](http://www.informatics.jax.org/javawi2/servlet/WIFetch?page=markerDetail&key=170837) |  |  |
| [Mirn324, microRNA 324](http://www.informatics.jax.org/javawi2/servlet/WIFetch?page=markerDetail&key=170848) |  |  |
| [Mirn365-2, microRNA 365-2](http://www.informatics.jax.org/javawi2/servlet/WIFetch?page=markerDetail&key=170877) |  |  |
| [Mirn423, microRNA 423](http://www.informatics.jax.org/javawi2/servlet/WIFetch?page=markerDetail&key=185797) |  |  |
| [Mirn451, microRNA 451](http://www.informatics.jax.org/javawi2/servlet/WIFetch?page=markerDetail&key=170905) |  |  |
| [Mirn467f, microRNA 467f](http://www.informatics.jax.org/javawi2/servlet/WIFetch?page=markerDetail&key=321357) |  |  |
| [Mirn497, microRNA 497](http://www.informatics.jax.org/javawi2/servlet/WIFetch?page=markerDetail&key=185807) |  |  |
| [Mis12, MIS12 homolog (yeast)](http://www.informatics.jax.org/javawi2/servlet/WIFetch?page=markerDetail&key=51119) |  |  |
| [Mks1, Meckel syndrome, type 1](http://www.informatics.jax.org/javawi2/servlet/WIFetch?page=markerDetail&key=156082) |  |  |
| [Mllt6, myeloid/lymphoid or mixed-lineage leukemia (trithorax homolog, Drosophila); translocated to, 6](http://www.informatics.jax.org/javawi2/servlet/WIFetch?page=markerDetail&key=80369) |  |  |
| [Mlx, MAX-like protein X](http://www.informatics.jax.org/javawi2/servlet/WIFetch?page=markerDetail&key=29985) |  |  |
| [Mmd, monocyte to macrophage differentiation-associated](http://www.informatics.jax.org/javawi2/servlet/WIFetch?page=markerDetail&key=51448) |  |  |
| [Mmp28, matrix metallopeptidase 28 (epilysin)](http://www.informatics.jax.org/javawi2/servlet/WIFetch?page=markerDetail&key=77785) | Gene trap | Cell line only |
| [Mnt, max binding protein](http://www.informatics.jax.org/javawi2/servlet/WIFetch?page=markerDetail&key=30913) | Targeted KO, targeted other | yes |
| [Motp1, modifier of Trp53 1](http://www.informatics.jax.org/javawi2/servlet/WIFetch?page=markerDetail&key=120015) | QTL |  |
| [Mpdu1, mannose-P-dolichol utilization defect 1](http://www.informatics.jax.org/javawi2/servlet/WIFetch?page=markerDetail&key=42694) |  |  |
| [Mpo, myeloperoxidase](http://www.informatics.jax.org/javawi2/servlet/WIFetch?page=markerDetail&key=11548) | Targeted KO | yes |
| [Mpp2, membrane protein, palmitoylated 2 (MAGUK p55 subfamily member 2)](http://www.informatics.jax.org/javawi2/servlet/WIFetch?page=markerDetail&key=46397) |  |  |
| [Mpp3, membrane protein, palmitoylated 3 (MAGUK p55 subfamily member 3)](http://www.informatics.jax.org/javawi2/servlet/WIFetch?page=markerDetail&key=39016) |  |  |
| [Mrm1, mitochondrial rRNA methyltransferase 1 homolog (S. cerevisiae)](http://www.informatics.jax.org/javawi2/servlet/WIFetch?page=markerDetail&key=84575) |  |  |
| [Mrpl10, mitochondrial ribosomal protein L10](http://www.informatics.jax.org/javawi2/servlet/WIFetch?page=markerDetail&key=39848) |  |  |
| [Mrpl27, mitochondrial ribosomal protein L27](http://www.informatics.jax.org/javawi2/servlet/WIFetch?page=markerDetail&key=65195) |  |  |
| [Mrpl45, mitochondrial ribosomal protein L45](http://www.informatics.jax.org/javawi2/servlet/WIFetch?page=markerDetail&key=51016) |  |  |
| [Mrps23, mitochondrial ribosomal protein S23](http://www.informatics.jax.org/javawi2/servlet/WIFetch?page=markerDetail&key=49093) |  |  |
| [Msi2, Musashi homolog 2 (Drosophila)](http://www.informatics.jax.org/javawi2/servlet/WIFetch?page=markerDetail&key=60606) |  |  |
| [Msl1, male-specific lethal 1 homolog (Drosophila)](http://www.informatics.jax.org/javawi2/servlet/WIFetch?page=markerDetail&key=58006) |  |  |
| [Mtmr4, myotubularin related protein 4](http://www.informatics.jax.org/javawi2/servlet/WIFetch?page=markerDetail&key=80577) |  |  |
| [Mybbp1a, MYB binding protein (P160) 1a](http://www.informatics.jax.org/javawi2/servlet/WIFetch?page=markerDetail&key=26913) |  |  |
| [Mycbpap, MYCBP associated protein](http://www.informatics.jax.org/javawi2/servlet/WIFetch?page=markerDetail&key=82180) |  |  |
| [Myo18a, myosin XVIIIA](http://www.informatics.jax.org/javawi2/servlet/WIFetch?page=markerDetail&key=91432) |  |  |
| [Myo19, myosin XIX](http://www.informatics.jax.org/javawi2/servlet/WIFetch?page=markerDetail&key=50176) |  |  |
| [Myo1c, myosin IC](http://www.informatics.jax.org/javawi2/servlet/WIFetch?page=markerDetail&key=27478) | Targeted other | yes |
| [Myo1d, myosin ID](http://www.informatics.jax.org/javawi2/servlet/WIFetch?page=markerDetail&key=29025) |  |  |
| [Myst2, MYST histone acetyltransferase 2](http://www.informatics.jax.org/javawi2/servlet/WIFetch?page=markerDetail&key=90321) |  |  |
| [Naglu, alpha-N-acetylglucosaminidase (Sanfilippo disease IIIB)](http://www.informatics.jax.org/javawi2/servlet/WIFetch?page=markerDetail&key=45225) | Targeted KO | yes |
| [Nags, N-acetylglutamate synthase](http://www.informatics.jax.org/javawi2/servlet/WIFetch?page=markerDetail&key=82199) |  |  |
| [Nbr1, neighbor of Brca1 gene 1](http://www.informatics.jax.org/javawi2/servlet/WIFetch?page=markerDetail&key=30087) |  |  |
| [Nek8, NIMA (never in mitosis gene a)-related expressed kinase 8](http://www.informatics.jax.org/javawi2/servlet/WIFetch?page=markerDetail&key=78832) | Spontaneous | yes |
| [Neurl4, neuralized homolog 4 (Drosophila)](http://www.informatics.jax.org/javawi2/servlet/WIFetch?page=markerDetail&key=57822) |  |  |
| [Neurod2, neurogenic differentiation 2](http://www.informatics.jax.org/javawi2/servlet/WIFetch?page=markerDetail&key=29063) | Targeted other | yes |
| [Nf1, neurofibromatosis 1](http://www.informatics.jax.org/javawi2/servlet/WIFetch?page=markerDetail&key=11841) | Targeted KO, targeted other | yes |
| [Nfe2l1, nuclear factor, erythroid derived 2,-like 1](http://www.informatics.jax.org/javawi2/servlet/WIFetch?page=markerDetail&key=15220) | Targeted KO, targeted other | yes |
| [Ngfr, nerve growth factor receptor (TNFR superfamily, member 16)](http://www.informatics.jax.org/javawi2/servlet/WIFetch?page=markerDetail&key=11861) | Targeted KO | yes |
| [Nilac2, nicotine induced locomotor activity 2](http://www.informatics.jax.org/javawi2/servlet/WIFetch?page=markerDetail&key=185653) | QTL |  |
| [Nkiras2, NFKB inhibitor interacting Ras-like protein 2](http://www.informatics.jax.org/javawi2/servlet/WIFetch?page=markerDetail&key=55946) |  |  |
| [Nle1, notchless homolog 1 (Drosophila)](http://www.informatics.jax.org/javawi2/servlet/WIFetch?page=markerDetail&key=82489) | Targeted KO | yes |
| [Nlgn2, neuroligin 2](http://www.informatics.jax.org/javawi2/servlet/WIFetch?page=markerDetail&key=94092) | Targeted KO | yes |
| [Nlk, nemo like kinase](http://www.informatics.jax.org/javawi2/servlet/WIFetch?page=markerDetail&key=35591) | Targeted other, gene trapped | yes |
| [Nlrp1a, NLR family, pyrin domain containing 1A](http://www.informatics.jax.org/javawi2/servlet/WIFetch?page=markerDetail&key=97233) |  |  |
| [Nlrp1b, NLR family, pyrin domain containing 1B](http://www.informatics.jax.org/javawi2/servlet/WIFetch?page=markerDetail&key=306159) |  |  |
| [Nlrp1c, NLR family, pyrin domain containing 1C](http://www.informatics.jax.org/javawi2/servlet/WIFetch?page=markerDetail&key=306160) |  |  |
| [Nme1, non-metastatic cells 1, protein (NM23A) expressed in](http://www.informatics.jax.org/javawi2/servlet/WIFetch?page=markerDetail&key=11923) | Targeted other, gene trapped | yes |
| [Nme2, non-metastatic cells 2, protein (NM23B) expressed in](http://www.informatics.jax.org/javawi2/servlet/WIFetch?page=markerDetail&key=11924) | Gene trap | Cell line only |
| [Nmt1, N-myristoyltransferase 1](http://www.informatics.jax.org/javawi2/servlet/WIFetch?page=markerDetail&key=18514) | Gene trap | yes |
| [Nog, noggin](http://www.informatics.jax.org/javawi2/servlet/WIFetch?page=markerDetail&key=24323) | Targeted other | yes |
| [Nos2, nitric oxide synthase 2, inducible](http://www.informatics.jax.org/javawi2/servlet/WIFetch?page=markerDetail&key=11935) | Targeted KO | yes |
| [Npepps, aminopeptidase puromycin sensitive](http://www.informatics.jax.org/javawi2/servlet/WIFetch?page=markerDetail&key=34713) | Targeted other, gene trapped | yes |
| [Nr1d1, nuclear receptor subfamily 1, group D, member 1](http://www.informatics.jax.org/javawi2/servlet/WIFetch?page=markerDetail&key=85194) | Targeted other | yes |
| [Nt5c3l, 5'-nucleotidase, cytosolic III-like](http://www.informatics.jax.org/javawi2/servlet/WIFetch?page=markerDetail&key=52086) |  |  |
| [Nufip2, nuclear fragile X mental retardation protein interacting protein 2](http://www.informatics.jax.org/javawi2/servlet/WIFetch?page=markerDetail&key=52544) |  |  |
| [Nup88, nucleoporin 88](http://www.informatics.jax.org/javawi2/servlet/WIFetch?page=markerDetail&key=25220) |  |  |
| [Nxn, nucleoredoxin](http://www.informatics.jax.org/javawi2/servlet/WIFetch?page=markerDetail&key=31356) |  |  |
| [Nxph3, neurexophilin 3](http://www.informatics.jax.org/javawi2/servlet/WIFetch?page=markerDetail&key=40335) | Targeted KO, targeted other | yes |
| [Olfr1, olfactory receptor 1](http://www.informatics.jax.org/javawi2/servlet/WIFetch?page=markerDetail&key=18651) |  |  |
| [Olfr139, olfactory receptor 139](http://www.informatics.jax.org/javawi2/servlet/WIFetch?page=markerDetail&key=89788) |  |  |
| [Olfr20, olfactory receptor 20](http://www.informatics.jax.org/javawi2/servlet/WIFetch?page=markerDetail&key=31340) |  |  |
| [Olfr22-ps1, olfactory receptor 22, pseudogene 1](http://www.informatics.jax.org/javawi2/servlet/WIFetch?page=markerDetail&key=31338) |  |  |
| [Olfr23, olfactory receptor 23](http://www.informatics.jax.org/javawi2/servlet/WIFetch?page=markerDetail&key=31337) |  |  |
| [Olfr376, olfactory receptor 376](http://www.informatics.jax.org/javawi2/servlet/WIFetch?page=markerDetail&key=101223) |  |  |
| [Olfr377-ps1, olfactory receptor 377, pseudogene 1](http://www.informatics.jax.org/javawi2/servlet/WIFetch?page=markerDetail&key=101224) |  |  |
| [Olfr378, olfactory receptor 378](http://www.informatics.jax.org/javawi2/servlet/WIFetch?page=markerDetail&key=101225) |  |  |
| [Olfr379-ps1, olfactory receptor 379, pseudogene 1](http://www.informatics.jax.org/javawi2/servlet/WIFetch?page=markerDetail&key=101226) |  |  |
| [Olfr380, olfactory receptor 380](http://www.informatics.jax.org/javawi2/servlet/WIFetch?page=markerDetail&key=101227) |  |  |
| [Olfr381, olfactory receptor 381](http://www.informatics.jax.org/javawi2/servlet/WIFetch?page=markerDetail&key=101228) |  |  |
| [Olfr382, olfactory receptor 382](http://www.informatics.jax.org/javawi2/servlet/WIFetch?page=markerDetail&key=101229) |  |  |
| [Olfr383-ps1, olfactory receptor 383, pseudogene 1](http://www.informatics.jax.org/javawi2/servlet/WIFetch?page=markerDetail&key=101230) |  |  |
| [Olfr385, olfactory receptor 385](http://www.informatics.jax.org/javawi2/servlet/WIFetch?page=markerDetail&key=101232) |  |  |
| [Olfr386, olfactory receptor 386](http://www.informatics.jax.org/javawi2/servlet/WIFetch?page=markerDetail&key=101233) |  |  |
| [Olfr387-ps1, olfactory receptor 387, pseudogene 1](http://www.informatics.jax.org/javawi2/servlet/WIFetch?page=markerDetail&key=101234) |  |  |
| [Olfr388-ps1, olfactory receptor 388, pseudogene 1](http://www.informatics.jax.org/javawi2/servlet/WIFetch?page=markerDetail&key=101235) |  |  |
| [Olfr389, olfactory receptor 389](http://www.informatics.jax.org/javawi2/servlet/WIFetch?page=markerDetail&key=101236) |  |  |
| [Olfr390, olfactory receptor 390](http://www.informatics.jax.org/javawi2/servlet/WIFetch?page=markerDetail&key=101237) |  |  |
| [Olfr391-ps1, olfactory receptor 391, pseudogene 1](http://www.informatics.jax.org/javawi2/servlet/WIFetch?page=markerDetail&key=101238) |  |  |
| [Olfr392, olfactory receptor 392](http://www.informatics.jax.org/javawi2/servlet/WIFetch?page=markerDetail&key=101239) |  |  |
| [Olfr393, olfactory receptor 393](http://www.informatics.jax.org/javawi2/servlet/WIFetch?page=markerDetail&key=101240) |  |  |
| [Olfr394, olfactory receptor 394](http://www.informatics.jax.org/javawi2/servlet/WIFetch?page=markerDetail&key=101241) |  |  |
| [Olfr395, olfactory receptor 395](http://www.informatics.jax.org/javawi2/servlet/WIFetch?page=markerDetail&key=101242) |  |  |
| [Olfr396-ps1, olfactory receptor 396, pseudogene 1](http://www.informatics.jax.org/javawi2/servlet/WIFetch?page=markerDetail&key=101243) |  |  |
| [Olfr397, olfactory receptor 397](http://www.informatics.jax.org/javawi2/servlet/WIFetch?page=markerDetail&key=101244) |  |  |
| [Olfr398, olfactory receptor 398](http://www.informatics.jax.org/javawi2/servlet/WIFetch?page=markerDetail&key=101245) |  |  |
| [Olfr399, olfactory receptor 399](http://www.informatics.jax.org/javawi2/servlet/WIFetch?page=markerDetail&key=101246) |  |  |
| [Olfr400-ps1, olfactory receptor 400, pseudogene 1](http://www.informatics.jax.org/javawi2/servlet/WIFetch?page=markerDetail&key=101247) |  |  |
| [Olfr401, olfactory receptor 401](http://www.informatics.jax.org/javawi2/servlet/WIFetch?page=markerDetail&key=101248) |  |  |
| [Olfr402, olfactory receptor 402](http://www.informatics.jax.org/javawi2/servlet/WIFetch?page=markerDetail&key=101249) |  |  |
| [Olfr403, olfactory receptor 403](http://www.informatics.jax.org/javawi2/servlet/WIFetch?page=markerDetail&key=101250) |  |  |
| [Olfr404-ps1, olfactory receptor 404, pseudogene 1](http://www.informatics.jax.org/javawi2/servlet/WIFetch?page=markerDetail&key=101251) |  |  |
| [Olfr405-ps1, olfactory receptor 405, pseudogene 1](http://www.informatics.jax.org/javawi2/servlet/WIFetch?page=markerDetail&key=101252) |  |  |
| [Olfr406, olfactory receptor 406](http://www.informatics.jax.org/javawi2/servlet/WIFetch?page=markerDetail&key=101253) |  |  |
| [Olfr407-ps1, olfactory receptor 407, pseudogene 1](http://www.informatics.jax.org/javawi2/servlet/WIFetch?page=markerDetail&key=101254) |  |  |
| [Olfr408-ps1, olfactory receptor 408, pseudogene 1](http://www.informatics.jax.org/javawi2/servlet/WIFetch?page=markerDetail&key=101255) |  |  |
| [Olfr409-ps1, olfactory receptor 409, pseudogene 1](http://www.informatics.jax.org/javawi2/servlet/WIFetch?page=markerDetail&key=101256) |  |  |
| [Olfr410, olfactory receptor 410](http://www.informatics.jax.org/javawi2/servlet/WIFetch?page=markerDetail&key=101257) |  |  |
| [Olfr411, olfactory receptor 411](http://www.informatics.jax.org/javawi2/servlet/WIFetch?page=markerDetail&key=101258) |  |  |
| [Olfr412, olfactory receptor 412](http://www.informatics.jax.org/javawi2/servlet/WIFetch?page=markerDetail&key=101259) |  |  |
| [Olfr43, olfactory receptor 43](http://www.informatics.jax.org/javawi2/servlet/WIFetch?page=markerDetail&key=39817) |  |  |
| [Olfr462, olfactory receptor 462](http://www.informatics.jax.org/javawi2/servlet/WIFetch?page=markerDetail&key=101309) |  |  |
| [Olfr463, olfactory receptor 463](http://www.informatics.jax.org/javawi2/servlet/WIFetch?page=markerDetail&key=101310) |  |  |
| [Olfr464, olfactory receptor 464](http://www.informatics.jax.org/javawi2/servlet/WIFetch?page=markerDetail&key=101311) |  |  |
| [Olfr59, olfactory receptor 59](http://www.informatics.jax.org/javawi2/servlet/WIFetch?page=markerDetail&key=39802) |  |  |
| [Olfr75-ps1, olfactory receptor 75, pseudogene 1](http://www.informatics.jax.org/javawi2/servlet/WIFetch?page=markerDetail&key=77888) |  |  |
| [Omg, oligodendrocyte myelin glycoprotein](http://www.informatics.jax.org/javawi2/servlet/WIFetch?page=markerDetail&key=27451) | Targeted KO, targeted other | yes |
| [Orch3, autoimmune orchitis resistance 3](http://www.informatics.jax.org/javawi2/servlet/WIFetch?page=markerDetail&key=29323) | QTL |  |
| [Ormdl3, ORM1-like 3 (S. cerevisiae)](http://www.informatics.jax.org/javawi2/servlet/WIFetch?page=markerDetail&key=50592) |  |  |
| [Osbpl7, oxysterol binding protein-like 7](http://www.informatics.jax.org/javawi2/servlet/WIFetch?page=markerDetail&key=55220) |  |  |
| [Ovca2, candidate tumor suppressor in ovarian cancer 2](http://www.informatics.jax.org/javawi2/servlet/WIFetch?page=markerDetail&key=80411) |  |  |
| [P140, P140 gene](http://www.informatics.jax.org/javawi2/servlet/WIFetch?page=markerDetail&key=63811) |  |  |
| [P2rx1, purinergic receptor P2X, ligand-gated ion channel, 1](http://www.informatics.jax.org/javawi2/servlet/WIFetch?page=markerDetail&key=33819) | Targeted other | yes |
| [P2rx5, purinergic receptor P2X, ligand-gated ion channel, 5](http://www.informatics.jax.org/javawi2/servlet/WIFetch?page=markerDetail&key=65161) |  |  |
| [Pafah1b1, platelet-activating factor acetylhydrolase, isoform 1b, beta1 subunit](http://www.informatics.jax.org/javawi2/servlet/WIFetch?page=markerDetail&key=36539) | Targeted KO, targeted other, gene trapped | yes |
| [Pas5, pulmonary adenoma susceptibility 5](http://www.informatics.jax.org/javawi2/servlet/WIFetch?page=markerDetail&key=42450) | QTL |  |
| [Pas5a, pulmonary adenoma susceptibility 5a](http://www.informatics.jax.org/javawi2/servlet/WIFetch?page=markerDetail&key=42473) | QTL |  |
| [Pas5b, pulmonary adenoma susceptibility 5b](http://www.informatics.jax.org/javawi2/servlet/WIFetch?page=markerDetail&key=42443) | QTL |  |
| [Pbd1, peak bone density 1](http://www.informatics.jax.org/javawi2/servlet/WIFetch?page=markerDetail&key=76119) | QTL |  |
| [Pcgf2, polycomb group ring finger 2](http://www.informatics.jax.org/javawi2/servlet/WIFetch?page=markerDetail&key=14945) | Targeted KO | yes |
| [Pcir2, periosteal circumference 2](http://www.informatics.jax.org/javawi2/servlet/WIFetch?page=markerDetail&key=92080) | QTL |  |
| [Pctp, phosphatidylcholine transfer protein](http://www.informatics.jax.org/javawi2/servlet/WIFetch?page=markerDetail&key=28476) | targeted KO, spontaneous | yes |
| [Pcyts3, plasmacytoma susceptibility 3](http://www.informatics.jax.org/javawi2/servlet/WIFetch?page=markerDetail&key=91237) | QTL |  |
| [Pdk2, pyruvate dehydrogenase kinase, isoenzyme 2](http://www.informatics.jax.org/javawi2/servlet/WIFetch?page=markerDetail&key=41912) |  |  |
| [Pelp1, proline, glutamic acid and leucine rich protein 1](http://www.informatics.jax.org/javawi2/servlet/WIFetch?page=markerDetail&key=59253) | Targeted other | Cell line only |
| [Perld1, per1-like domain containing 1](http://www.informatics.jax.org/javawi2/servlet/WIFetch?page=markerDetail&key=85400) |  |  |
| [Pex12, peroxisomal biogenesis factor 12](http://www.informatics.jax.org/javawi2/servlet/WIFetch?page=markerDetail&key=71617) |  |  |
| [Pfn1, profilin 1](http://www.informatics.jax.org/javawi2/servlet/WIFetch?page=markerDetail&key=12276) | Targeted KO | yes |
| [Pgia7, proteoglycan induced arthritis 7](http://www.informatics.jax.org/javawi2/servlet/WIFetch?page=markerDetail&key=76807) | QTL |  |
| [Phb, prohibitin](http://www.informatics.jax.org/javawi2/servlet/WIFetch?page=markerDetail&key=12317) | Gene trap | yes |
| [Phf12, PHD finger protein 12](http://www.informatics.jax.org/javawi2/servlet/WIFetch?page=markerDetail&key=60787) |  |  |
| [Phf23, PHD finger protein 23](http://www.informatics.jax.org/javawi2/servlet/WIFetch?page=markerDetail&key=62226) |  |  |
| [Phospho1, phosphatase, orphan 1](http://www.informatics.jax.org/javawi2/servlet/WIFetch?page=markerDetail&key=82908) |  |  |
| [Pigs, phosphatidylinositol glycan anchor biosynthesis, class S](http://www.informatics.jax.org/javawi2/servlet/WIFetch?page=markerDetail&key=100816) |  |  |
| [Pigw, phosphatidylinositol glycan anchor biosynthesis, class W](http://www.informatics.jax.org/javawi2/servlet/WIFetch?page=markerDetail&key=54305) |  |  |
| [Pip4k2b, phosphatidylinositol-5-phosphate 4-kinase, type II, beta](http://www.informatics.jax.org/javawi2/servlet/WIFetch?page=markerDetail&key=64232) | Gene trap | yes |
| [Pipox, pipecolic acid oxidase](http://www.informatics.jax.org/javawi2/servlet/WIFetch?page=markerDetail&key=35412) |  |  |
| [Pitpna, phosphatidylinositol transfer protein, alpha](http://www.informatics.jax.org/javawi2/servlet/WIFetch?page=markerDetail&key=15724) | Targeted KO, gene trapped, spontaneous | yes |
| [Pitpnm3, PITPNM family member 3](http://www.informatics.jax.org/javawi2/servlet/WIFetch?page=markerDetail&key=98098) |  |  |
| [Plcd3, phospholipase C, delta 3](http://www.informatics.jax.org/javawi2/servlet/WIFetch?page=markerDetail&key=28594) | Gene trap | Cell line only |
| [Pld2, phospholipase D2](http://www.informatics.jax.org/javawi2/servlet/WIFetch?page=markerDetail&key=32500) |  |  |
| [Plekhh3, pleckstrin homology domain containing, family H (with MyTH4 domain) member 3](http://www.informatics.jax.org/javawi2/servlet/WIFetch?page=markerDetail&key=91102) | Targeted KO | Cell line only |
| [Plekhm1, pleckstrin homology domain containing, family M (with RUN domain) member 1](http://www.informatics.jax.org/javawi2/servlet/WIFetch?page=markerDetail&key=84355) | Gene trap | Cell line only |
| [Plscr3, phospholipid scramblase 3](http://www.informatics.jax.org/javawi2/servlet/WIFetch?page=markerDetail&key=54290) | Targeted KO | yes |
| [Pltct2, platelet count 2](http://www.informatics.jax.org/javawi2/servlet/WIFetch?page=markerDetail&key=118650) | QTL |  |
| [Plxdc1, plexin domain containing 1](http://www.informatics.jax.org/javawi2/servlet/WIFetch?page=markerDetail&key=56304) | Targeted other, gene trapped | Reporter used to look at expression? |
| [Pnmt, phenylethanolamine-N-methyltransferase](http://www.informatics.jax.org/javawi2/servlet/WIFetch?page=markerDetail&key=12573) | Targeted other | yes |
| [Pnpo, pyridoxine 5'-phosphate oxidase](http://www.informatics.jax.org/javawi2/servlet/WIFetch?page=markerDetail&key=71591) |  |  |
| [Poldip2, polymerase (DNA-directed), delta interacting protein 2](http://www.informatics.jax.org/javawi2/servlet/WIFetch?page=markerDetail&key=51791) |  |  |
| [Polr2a, polymerase (RNA) II (DNA directed) polypeptide A](http://www.informatics.jax.org/javawi2/servlet/WIFetch?page=markerDetail&key=13140) | Targeted KO, targeted other | yes |
| [Ppm1d, protein phosphatase 1D magnesium-dependent, delta isoform](http://www.informatics.jax.org/javawi2/servlet/WIFetch?page=markerDetail&key=46354) | Targeted KO | yes |
| [Ppm1e, protein phosphatase 1E (PP2C domain containing)](http://www.informatics.jax.org/javawi2/servlet/WIFetch?page=markerDetail&key=85101) |  |  |
| [Ppp1r1b, protein phosphatase 1, regulatory (inhibitor) subunit 1B](http://www.informatics.jax.org/javawi2/servlet/WIFetch?page=markerDetail&key=37757) | Targeted KO, targeted other | yes |
| [Ppp1r9b, protein phosphatase 1, regulatory subunit 9B](http://www.informatics.jax.org/javawi2/servlet/WIFetch?page=markerDetail&key=82284) | Targeted KO | yes |
| [Ppy, pancreatic polypeptide](http://www.informatics.jax.org/javawi2/servlet/WIFetch?page=markerDetail&key=12623) | Targeted KO | yes |
| [Prdt3, prion disease incubation time 3](http://www.informatics.jax.org/javawi2/servlet/WIFetch?page=markerDetail&key=76756) | QTL |  |
| [Pregq1, pregnancy QTL 1](http://www.informatics.jax.org/javawi2/servlet/WIFetch?page=markerDetail&key=185948) | QTL |  |
| [Pregq2, pregnancy QTL 2](http://www.informatics.jax.org/javawi2/servlet/WIFetch?page=markerDetail&key=185949) | QTL |  |
| [Prpf8, pre-mRNA processing factor 8](http://www.informatics.jax.org/javawi2/servlet/WIFetch?page=markerDetail&key=80276) |  |  |
| [Prr11, proline rich 11](http://www.informatics.jax.org/javawi2/servlet/WIFetch?page=markerDetail&key=85431) |  |  |
| [Psmb3, proteasome (prosome, macropain) subunit, beta type 3](http://www.informatics.jax.org/javawi2/servlet/WIFetch?page=markerDetail&key=43395) |  |  |
| [Psmb5-ps, proteasome (prosome, macropain) subunit, beta type 5, pseudogene](http://www.informatics.jax.org/javawi2/servlet/WIFetch?page=markerDetail&key=34830) |  |  |
| [Psmb6, proteasome (prosome, macropain) subunit, beta type 6](http://www.informatics.jax.org/javawi2/servlet/WIFetch?page=markerDetail&key=25200) |  |  |
| [Psmc3ip, proteasome (prosome, macropain) 26S subunit, ATPase 3, interacting protein](http://www.informatics.jax.org/javawi2/servlet/WIFetch?page=markerDetail&key=33988) | Targeted KO | yes |
| [Psmd11, proteasome (prosome, macropain) 26S subunit, non-ATPase, 11](http://www.informatics.jax.org/javawi2/servlet/WIFetch?page=markerDetail&key=53057) | Gene trap | Cell line only |
| [Psmd3, proteasome (prosome, macropain) 26S subunit, non-ATPase, 3](http://www.informatics.jax.org/javawi2/servlet/WIFetch?page=markerDetail&key=14318) |  |  |
| [Psme3, proteaseome (prosome, macropain) 28 subunit, 3](http://www.informatics.jax.org/javawi2/servlet/WIFetch?page=markerDetail&key=33410) | Targeted KO | yes |
| [Pstc2, periosteal circumference 2](http://www.informatics.jax.org/javawi2/servlet/WIFetch?page=markerDetail&key=306093) | QTL |  |
| [Ptrf, polymerase I and transcript release factor](http://www.informatics.jax.org/javawi2/servlet/WIFetch?page=markerDetail&key=37292) | Targeted other | yes |
| [Ptrh2, peptidyl-tRNA hydrolase 2](http://www.informatics.jax.org/javawi2/servlet/WIFetch?page=markerDetail&key=85726) | Targeted KO, targeted other | yes |
| [Pyy, peptide YY](http://www.informatics.jax.org/javawi2/servlet/WIFetch?page=markerDetail&key=15770) | Targeted KO, targeted other | yes |
| [Qbis3, QTL for body weight independent of sex 3](http://www.informatics.jax.org/javawi2/servlet/WIFetch?page=markerDetail&key=158661) | QTL |  |
| [Rab11fip4, RAB11 family interacting protein 4 (class II)](http://www.informatics.jax.org/javawi2/servlet/WIFetch?page=markerDetail&key=84098) |  |  |
| [Rab34, RAB34, member of RAS oncogene family](http://www.informatics.jax.org/javawi2/servlet/WIFetch?page=markerDetail&key=24800) | Gene trap | Cell line only |
| [Rab5c, RAB5C, member RAS oncogene family](http://www.informatics.jax.org/javawi2/servlet/WIFetch?page=markerDetail&key=25785) |  |  |
| [Rabep1, rabaptin, RAB GTPase binding effector protein 1](http://www.informatics.jax.org/javawi2/servlet/WIFetch?page=markerDetail&key=46992) | Gene trap | Cell line only |
| [Rad51c, RAD51 homolog c (S. cerevisiae)](http://www.informatics.jax.org/javawi2/servlet/WIFetch?page=markerDetail&key=76203) | Targeted KO, targeted other, gene trapped | yes |
| [Rad51l3, RAD51-like 3 (S. cerevisiae)](http://www.informatics.jax.org/javawi2/servlet/WIFetch?page=markerDetail&key=36541) | Targeted KO | yes |
| [Rafar, retinoic acid induced forelimb autopod reduction](http://www.informatics.jax.org/javawi2/servlet/WIFetch?page=markerDetail&key=154985) | QTL |  |
| [Rai12, retinoic acid induced 12](http://www.informatics.jax.org/javawi2/servlet/WIFetch?page=markerDetail&key=46623) |  |  |
| [Ramp2, receptor (calcitonin) activity modifying protein 2](http://www.informatics.jax.org/javawi2/servlet/WIFetch?page=markerDetail&key=46835) | Targeted KO, targeted other | yes |
| [Rapgefl1, Rap guanine nucleotide exchange factor (GEF)-like 1](http://www.informatics.jax.org/javawi2/servlet/WIFetch?page=markerDetail&key=158514) |  |  |
| [Rara, retinoic acid receptor, alpha](http://www.informatics.jax.org/javawi2/servlet/WIFetch?page=markerDetail&key=12794) | Targeted KO, targeted other | yes |
| [Rasl10b, RAS-like, family 10, member B](http://www.informatics.jax.org/javawi2/servlet/WIFetch?page=markerDetail&key=97947) |  |  |
| [Rbcq3, red blood cell QTL 3](http://www.informatics.jax.org/javawi2/servlet/WIFetch?page=markerDetail&key=171438) | QTL |  |
| [Rdm1, RAD52 motif 1](http://www.informatics.jax.org/javawi2/servlet/WIFetch?page=markerDetail&key=50579) |  |  |
| [Rffl, ring finger and FYVE like domain containing protein](http://www.informatics.jax.org/javawi2/servlet/WIFetch?page=markerDetail&key=51318) |  |  |
| [Rhbdl3, rhomboid, veinlet-like 3 (Drosophila)](http://www.informatics.jax.org/javawi2/servlet/WIFetch?page=markerDetail&key=80254) |  |  |
| [Rhot1, ras homolog gene family, member T1](http://www.informatics.jax.org/javawi2/servlet/WIFetch?page=markerDetail&key=62808) |  |  |
| [Rilp, Rab interacting lysosomal protein](http://www.informatics.jax.org/javawi2/servlet/WIFetch?page=markerDetail&key=71711) | Targeted KO | Cell line only |
| [Rnasek, ribonuclease, RNase K](http://www.informatics.jax.org/javawi2/servlet/WIFetch?page=markerDetail&key=27158) |  |  |
| [Rnd2, Rho family GTPase 2](http://www.informatics.jax.org/javawi2/servlet/WIFetch?page=markerDetail&key=40832) |  |  |
| [Rnf135, ring finger protein 135](http://www.informatics.jax.org/javawi2/servlet/WIFetch?page=markerDetail&key=55936) |  |  |
| [Rnf167, ring finger protein 167](http://www.informatics.jax.org/javawi2/servlet/WIFetch?page=markerDetail&key=54490) |  |  |
| [Rnf43, ring finger protein 43](http://www.informatics.jax.org/javawi2/servlet/WIFetch?page=markerDetail&key=83833) |  |  |
| [Rnft1, ring finger protein, transmembrane 1](http://www.informatics.jax.org/javawi2/servlet/WIFetch?page=markerDetail&key=60872) |  |  |
| [Rnmtl1, RNA methyltransferase like 1](http://www.informatics.jax.org/javawi2/servlet/WIFetch?page=markerDetail&key=51370) |  |  |
| [Rpa1, replication protein A1](http://www.informatics.jax.org/javawi2/servlet/WIFetch?page=markerDetail&key=52255) | Targeted other | yes |
| [Rpain, RPA interacting protein](http://www.informatics.jax.org/javawi2/servlet/WIFetch?page=markerDetail&key=53703) |  |  |
| [Rph3al, rabphilin 3A-like (without C2 domains)](http://www.informatics.jax.org/javawi2/servlet/WIFetch?page=markerDetail&key=60222) | Targeted KO | yes |
| [Rpl19, ribosomal protein L19](http://www.informatics.jax.org/javawi2/servlet/WIFetch?page=markerDetail&key=13072) |  |  |
| [Rpl23, ribosomal protein L23](http://www.informatics.jax.org/javawi2/servlet/WIFetch?page=markerDetail&key=49699) |  |  |
| [Rpl23a, ribosomal protein L23A](http://www.informatics.jax.org/javawi2/servlet/WIFetch?page=markerDetail&key=105485) |  |  |
| [Rpl27, ribosomal protein L27](http://www.informatics.jax.org/javawi2/servlet/WIFetch?page=markerDetail&key=13088) |  |  |
| [Rprml, reprimo-like](http://www.informatics.jax.org/javawi2/servlet/WIFetch?page=markerDetail&key=71926) |  |  |
| [Rps6kb1, ribosomal protein S6 kinase, polypeptide 1](http://www.informatics.jax.org/javawi2/servlet/WIFetch?page=markerDetail&key=36557) | Targeted KO, gene trap | yes |
| [Rsad1, radical S-adenosyl methionine domain containing 1](http://www.informatics.jax.org/javawi2/servlet/WIFetch?page=markerDetail&key=105276) | Targeted KO | Cell line only |
| [Rtn4rl1, reticulon 4 receptor-like 1](http://www.informatics.jax.org/javawi2/servlet/WIFetch?page=markerDetail&key=90020) | Gene trap | yes |
| [Rundc1, RUN domain containing 1](http://www.informatics.jax.org/javawi2/servlet/WIFetch?page=markerDetail&key=71946) |  |  |
| [Rundc3a, RUN domain containing 3A](http://www.informatics.jax.org/javawi2/servlet/WIFetch?page=markerDetail&key=46561) |  |  |
| [Samd14, sterile alpha motif domain containing 14](http://www.informatics.jax.org/javawi2/servlet/WIFetch?page=markerDetail&key=81817) |  |  |
| [Sarm1, sterile alpha and HEAT/Armadillo motif containing 1](http://www.informatics.jax.org/javawi2/servlet/WIFetch?page=markerDetail&key=64924) | Targeted KO | yes |
| [Sat2, spermidine/spermine N1-acetyl transferase 2](http://www.informatics.jax.org/javawi2/servlet/WIFetch?page=markerDetail&key=53195) |  |  |
| [Sbmd4, spinal bone mineral density 4](http://www.informatics.jax.org/javawi2/servlet/WIFetch?page=markerDetail&key=90725) | QTL |  |
| [Scarf1, scavenger receptor class F, member 1](http://www.informatics.jax.org/javawi2/servlet/WIFetch?page=markerDetail&key=117733) | Targeted KO | yes only in double KO with Mrs1 |
| [Scpep1, serine carboxypeptidase 1](http://www.informatics.jax.org/javawi2/servlet/WIFetch?page=markerDetail&key=58597) | Gene trap | Cell line only |
| [Scrn2, secernin 2](http://www.informatics.jax.org/javawi2/servlet/WIFetch?page=markerDetail&key=41917) |  |  |
| [Sdf2, stromal cell derived factor 2](http://www.informatics.jax.org/javawi2/servlet/WIFetch?page=markerDetail&key=29422) |  |  |
| [Sebox, SEBOX homeobox](http://www.informatics.jax.org/javawi2/servlet/WIFetch?page=markerDetail&key=29413) | Targeted KO | No phenotype upon observation |
| [Senp3, SUMO/sentrin specific peptidase 3](http://www.informatics.jax.org/javawi2/servlet/WIFetch?page=markerDetail&key=79329) |  |  |
| [Sept4, septin 4](http://www.informatics.jax.org/javawi2/servlet/WIFetch?page=markerDetail&key=36512) | Targeted KO | yes |
| [Serpinf1, serine (or cysteine) peptidase inhibitor, clade F, member 1](http://www.informatics.jax.org/javawi2/servlet/WIFetch?page=markerDetail&key=29549) | Targeted other | yes |
| [Serpinf2, serine (or cysteine) peptidase inhibitor, clade F, member 2](http://www.informatics.jax.org/javawi2/servlet/WIFetch?page=markerDetail&key=28146) | Targeted KO | yes |
| [Sez6, seizure related gene 6](http://www.informatics.jax.org/javawi2/servlet/WIFetch?page=markerDetail&key=25005) | Targeted KO | yes |
| [Sfrs1, splicing factor, arginine/serine-rich 1 (ASF/SF2)](http://www.informatics.jax.org/javawi2/servlet/WIFetch?page=markerDetail&key=13401) | Targeted KO, targeted other | yes |
| [Sgca, sarcoglycan, alpha (dystrophin-associated glycoprotein)](http://www.informatics.jax.org/javawi2/servlet/WIFetch?page=markerDetail&key=32968) | Targeted KO, targeted other | yes |
| [Sgsm2, small G protein signaling modulator 2](http://www.informatics.jax.org/javawi2/servlet/WIFetch?page=markerDetail&key=72135) |  |  |
| [Shbg, sex hormone binding globulin](http://www.informatics.jax.org/javawi2/servlet/WIFetch?page=markerDetail&key=13431) |  |  |
| [Shpk, sedoheptulokinase](http://www.informatics.jax.org/javawi2/servlet/WIFetch?page=markerDetail&key=58617) |  |  |
| [Si5lq6, serum IGFBP-5 level QTL 6](http://www.informatics.jax.org/javawi2/servlet/WIFetch?page=markerDetail&key=117023) | QTL |  |
| [Skap1, src family associated phosphoprotein 1](http://www.informatics.jax.org/javawi2/servlet/WIFetch?page=markerDetail&key=62453) | Gene trap | yes |
| [Skl5, skeletal size (tail length) 5](http://www.informatics.jax.org/javawi2/servlet/WIFetch?page=markerDetail&key=75938) | QTL |  |
| [Skull16, skull morphology 16](http://www.informatics.jax.org/javawi2/servlet/WIFetch?page=markerDetail&key=76100) | QTL |  |
| [Slc13a2, solute carrier family 13 (sodium-dependent dicarboxylate transporter), member 2](http://www.informatics.jax.org/javawi2/servlet/WIFetch?page=markerDetail&key=39101) |  |  |
| [Slc13a5, solute carrier family 13 (sodium-dependent citrate transporter), member 5](http://www.informatics.jax.org/javawi2/servlet/WIFetch?page=markerDetail&key=104710) |  |  |
| [Slc16a11, solute carrier family 16 (monocarboxylic acid transporters), member 11](http://www.informatics.jax.org/javawi2/servlet/WIFetch?page=markerDetail&key=90895) |  |  |
| [Slc16a13, solute carrier family 16 (monocarboxylic acid transporters), member 13](http://www.informatics.jax.org/javawi2/servlet/WIFetch?page=markerDetail&key=53289) |  |  |
| [Slc25a11, solute carrier family 25 (mitochondrial carrier oxoglutarate carrier), member 11](http://www.informatics.jax.org/javawi2/servlet/WIFetch?page=markerDetail&key=51843) |  |  |
| [Slc25a39, solute carrier family 25, member 39](http://www.informatics.jax.org/javawi2/servlet/WIFetch?page=markerDetail&key=35256) |  |  |
| [Slc2a4, solute carrier family 2 (facilitated glucose transporter), member 4](http://www.informatics.jax.org/javawi2/servlet/WIFetch?page=markerDetail&key=33086) | Targeted KO, targeted other | yes |
| [Slc35b1, solute carrier family 35, member B1](http://www.informatics.jax.org/javawi2/servlet/WIFetch?page=markerDetail&key=41958) | Gene trap | Cell line only |
| [Slc43a2, solute carrier family 43, member 2](http://www.informatics.jax.org/javawi2/servlet/WIFetch?page=markerDetail&key=83950) |  |  |
| [Slc46a1, solute carrier family 46, member 1](http://www.informatics.jax.org/javawi2/servlet/WIFetch?page=markerDetail&key=34111) |  |  |
| [Slc4a1, solute carrier family 4 (anion exchanger), member 1](http://www.informatics.jax.org/javawi2/servlet/WIFetch?page=markerDetail&key=31435) | Targeted KO, targeted other, spontaneous, chemically induced | yes |
| [Slc6a4, solute carrier family 6 (neurotransmitter transporter, serotonin), member 4](http://www.informatics.jax.org/javawi2/servlet/WIFetch?page=markerDetail&key=10027) | Targeted KO, targeted other, gene trapped | yes |
| [Sle13, systematic lupus erythematosus susceptibility 13](http://www.informatics.jax.org/javawi2/servlet/WIFetch?page=markerDetail&key=76118) |  |  |
| [Slfn1, schlafen 1](http://www.informatics.jax.org/javawi2/servlet/WIFetch?page=markerDetail&key=38201) | Targeted KO | yes |
| [Slfn10, schlafen 10](http://www.informatics.jax.org/javawi2/servlet/WIFetch?page=markerDetail&key=118679) |  |  |
| [Slfn14, schlafen family member 14](http://www.informatics.jax.org/javawi2/servlet/WIFetch?page=markerDetail&key=97238) |  |  |
| [Slfn2, schlafen 2](http://www.informatics.jax.org/javawi2/servlet/WIFetch?page=markerDetail&key=38200) |  |  |
| [Slfn3, schlafen 3](http://www.informatics.jax.org/javawi2/servlet/WIFetch?page=markerDetail&key=39114) |  |  |
| [Slfn4, schlafen 4](http://www.informatics.jax.org/javawi2/servlet/WIFetch?page=markerDetail&key=39119) |  |  |
| [Slfn5, schlafen 5](http://www.informatics.jax.org/javawi2/servlet/WIFetch?page=markerDetail&key=39113) |  |  |
| [Slfn8, schlafen 8](http://www.informatics.jax.org/javawi2/servlet/WIFetch?page=markerDetail&key=105481) |  |  |
| [Slfn9, schlafen 9](http://www.informatics.jax.org/javawi2/servlet/WIFetch?page=markerDetail&key=85946) |  |  |
| [Slthsr, semilethal sex-restricted](http://www.informatics.jax.org/javawi2/servlet/WIFetch?page=markerDetail&key=75635) | QTL |  |
| [Smarce1, SWI/SNF related, matrix associated, actin dependent regulator of chromatin, subfamily e, member 1](http://www.informatics.jax.org/javawi2/servlet/WIFetch?page=markerDetail&key=48849) |  |  |
| [Smg6, Smg-6 homolog, nonsense mediated mRNA decay factor (C. elegans)](http://www.informatics.jax.org/javawi2/servlet/WIFetch?page=markerDetail&key=71557) | Gene trap | Cell line only |
| [Smtnl2, smoothelin-like 2](http://www.informatics.jax.org/javawi2/servlet/WIFetch?page=markerDetail&key=83968) |  |  |
| [Smyd4, SET and MYND domain containing 4](http://www.informatics.jax.org/javawi2/servlet/WIFetch?page=markerDetail&key=83994) |  |  |
| [Snf8, SNF8, ESCRT-II complex subunit, homolog (S. cerevisiae)](http://www.informatics.jax.org/javawi2/servlet/WIFetch?page=markerDetail&key=41986) |  |  |
| [Snora21, small nucleolar RNA, H/ACA box 21](http://www.informatics.jax.org/javawi2/servlet/WIFetch?page=markerDetail&key=329071) |  |  |
| [Snord42a, small nucleolar RNA, C/D box 42A](http://www.informatics.jax.org/javawi2/servlet/WIFetch?page=markerDetail&key=329110) |  |  |
| [Snord42b, small nucleolar RNA, C/D box 42B](http://www.informatics.jax.org/javawi2/servlet/WIFetch?page=markerDetail&key=329111) |  |  |
| [Snord4a, small nucleolar RNA, C/D box 4A](http://www.informatics.jax.org/javawi2/servlet/WIFetch?page=markerDetail&key=329118) |  |  |
| [Snord7, small nucleolar RNA, C/D box 7](http://www.informatics.jax.org/javawi2/servlet/WIFetch?page=markerDetail&key=329130) |  |  |
| [Snord91a, small nucleolar RNA, C/D box 91A](http://www.informatics.jax.org/javawi2/servlet/WIFetch?page=markerDetail&key=329142) |  |  |
| [Snx11, sorting nexin 11](http://www.informatics.jax.org/javawi2/servlet/WIFetch?page=markerDetail&key=58459) |  |  |
| [Socs7, suppressor of cytokine signaling 7](http://www.informatics.jax.org/javawi2/servlet/WIFetch?page=markerDetail&key=87304) | Targeted KO | yes |
| [Sost, sclerostin](http://www.informatics.jax.org/javawi2/servlet/WIFetch?page=markerDetail&key=58479) | Targeted KO | Cell line only |
| [Sox15, SRY-box containing gene 15](http://www.informatics.jax.org/javawi2/servlet/WIFetch?page=markerDetail&key=13533) | Targeted KO | yes |
| [Sox16, SRY-box containing gene 16](http://www.informatics.jax.org/javawi2/servlet/WIFetch?page=markerDetail&key=76998) |  |  |
| [Sp2, Sp2 transcription factor](http://www.informatics.jax.org/javawi2/servlet/WIFetch?page=markerDetail&key=62892) |  |  |
| [Sp6, trans-acting transcription factor 6](http://www.informatics.jax.org/javawi2/servlet/WIFetch?page=markerDetail&key=63711) | Targeted KO, targeted other | yes |
| [Spaca3, sperm acrosome associated 3](http://www.informatics.jax.org/javawi2/servlet/WIFetch?page=markerDetail&key=59602) |  |  |
| [Spag5, sperm associated antigen 5](http://www.informatics.jax.org/javawi2/servlet/WIFetch?page=markerDetail&key=48876) | Targeted KO | No phenotype upon observation |
| [Spag7, sperm associated antigen 7](http://www.informatics.jax.org/javawi2/servlet/WIFetch?page=markerDetail&key=28481) |  |  |
| [Spag9, sperm associated antigen 9](http://www.informatics.jax.org/javawi2/servlet/WIFetch?page=markerDetail&key=54814) | Targeted KO | yes |
| [Spata20, spermatogenesis associated 20](http://www.informatics.jax.org/javawi2/servlet/WIFetch?page=markerDetail&key=81011) |  |  |
| [Spata22, spermatogenesis associated 22](http://www.informatics.jax.org/javawi2/servlet/WIFetch?page=markerDetail&key=98100) |  |  |
| [Spem1, sperm maturation 1](http://www.informatics.jax.org/javawi2/servlet/WIFetch?page=markerDetail&key=58268) | Targeted KO | yes |
| [Spns2, spinster homolog 2 (Drosophila)](http://www.informatics.jax.org/javawi2/servlet/WIFetch?page=markerDetail&key=91806) |  |  |
| [Spns3, spinster homolog 3 (Drosophila)](http://www.informatics.jax.org/javawi2/servlet/WIFetch?page=markerDetail&key=61557) |  |  |
| [Spop, speckle-type POZ protein](http://www.informatics.jax.org/javawi2/servlet/WIFetch?page=markerDetail&key=41910) |  |  |
| [Srr, serine racemase](http://www.informatics.jax.org/javawi2/servlet/WIFetch?page=markerDetail&key=45220) | Targeted other, gene trapped | yes |
| [Ssh2, slingshot homolog 2 (Drosophila)](http://www.informatics.jax.org/javawi2/servlet/WIFetch?page=markerDetail&key=93570) |  |  |
| [Stac2, SH3 and cysteine rich domain 2](http://www.informatics.jax.org/javawi2/servlet/WIFetch?page=markerDetail&key=71958) |  |  |
| [Stard3, START domain containing 3](http://www.informatics.jax.org/javawi2/servlet/WIFetch?page=markerDetail&key=49790) | Targeted KO | yes |
| [Stat3, signal transducer and activator of transcription 3](http://www.informatics.jax.org/javawi2/servlet/WIFetch?page=markerDetail&key=22952) | Targeted KO, targeted other | yes |
| [Stat5a, signal transducer and activator of transcription 5A](http://www.informatics.jax.org/javawi2/servlet/WIFetch?page=markerDetail&key=22950) | Targeted KO, targeted other | yes |
| [Stat5b, signal transducer and activator of transcription 5B](http://www.informatics.jax.org/javawi2/servlet/WIFetch?page=markerDetail&key=22949) | Targeted KO, targeted other | yes |
| [Stxbp4, syntaxin binding protein 4](http://www.informatics.jax.org/javawi2/servlet/WIFetch?page=markerDetail&key=41812) |  |  |
| [Stzid, streptozotocin induced diabetes susceptibility](http://www.informatics.jax.org/javawi2/servlet/WIFetch?page=markerDetail&key=119520) |  |  |
| [Supt4h1, suppressor of Ty 4 homolog 1 (S. cerevisiae)](http://www.informatics.jax.org/javawi2/servlet/WIFetch?page=markerDetail&key=28557) |  |  |
| [Supt6h, suppressor of Ty 6 homolog (S. cerevisiae)](http://www.informatics.jax.org/javawi2/servlet/WIFetch?page=markerDetail&key=29023) |  |  |
| [Suz12, suppressor of zeste 12 homolog (Drosophila)](http://www.informatics.jax.org/javawi2/servlet/WIFetch?page=markerDetail&key=36219) | targeted KO, gene trap, chemically induced | yes |
| [Tac4, tachykinin 4](http://www.informatics.jax.org/javawi2/servlet/WIFetch?page=markerDetail&key=64688) |  |  |
| [Tada2l, transcriptional adaptor 2 (ADA2 homolog, yeast)-like](http://www.informatics.jax.org/javawi2/servlet/WIFetch?page=markerDetail&key=71911) |  |  |
| [Taf15, TAF15 RNA polymerase II, TATA box binding protein (TBP)-associated factor](http://www.informatics.jax.org/javawi2/servlet/WIFetch?page=markerDetail&key=54419) |  |  |
| [Tailq8, tail length QTL 8](http://www.informatics.jax.org/javawi2/servlet/WIFetch?page=markerDetail&key=306627) | QTL |  |
| [Taok1, TAO kinase 1](http://www.informatics.jax.org/javawi2/servlet/WIFetch?page=markerDetail&key=51220) |  |  |
| [Tax1bp3, Tax1 (human T-cell leukemia virus type I) binding protein 3](http://www.informatics.jax.org/javawi2/servlet/WIFetch?page=markerDetail&key=60261) |  |  |
| [Tbbmd5, total body bone mineral density 5](http://www.informatics.jax.org/javawi2/servlet/WIFetch?page=markerDetail&key=91856) | QTL |  |
| [Tbkbp1, TBK1 binding protein 1](http://www.informatics.jax.org/javawi2/servlet/WIFetch?page=markerDetail&key=57154) |  |  |
| [Tbx2, T-box 2](http://www.informatics.jax.org/javawi2/servlet/WIFetch?page=markerDetail&key=13763) | targeted KO | yes |
| [Tbx21, T-box 21](http://www.informatics.jax.org/javawi2/servlet/WIFetch?page=markerDetail&key=47595) | Targeted KO | yes |
| [Tbx4, T-box 4](http://www.informatics.jax.org/javawi2/servlet/WIFetch?page=markerDetail&key=18453) | Targeted KO, targeted other | yes |
| [Tcap, titin-cap](http://www.informatics.jax.org/javawi2/servlet/WIFetch?page=markerDetail&key=39219) |  |  |
| [Tekt1, tektin 1](http://www.informatics.jax.org/javawi2/servlet/WIFetch?page=markerDetail&key=39866) |  |  |
| [Tex14, testis expressed gene 14](http://www.informatics.jax.org/javawi2/servlet/WIFetch?page=markerDetail&key=63864) | Targeted KO | yes |
| [Thra, thyroid hormone receptor alpha](http://www.informatics.jax.org/javawi2/servlet/WIFetch?page=markerDetail&key=14123) | Targeted KO, targeted other | yes |
| [Timm22, translocase of inner mitochondrial membrane 22 homolog (yeast)](http://www.informatics.jax.org/javawi2/servlet/WIFetch?page=markerDetail&key=49869) |  |  |
| [Tlcd1, TLC domain containing 1](http://www.informatics.jax.org/javawi2/servlet/WIFetch?page=markerDetail&key=52302) |  |  |
| [Tlcd2, TLC domain containing 2](http://www.informatics.jax.org/javawi2/servlet/WIFetch?page=markerDetail&key=53871) |  |  |
| [Tm4sf5, transmembrane 4 superfamily member 5](http://www.informatics.jax.org/javawi2/servlet/WIFetch?page=markerDetail&key=59584) |  |  |
| [Tmc1m2, Tmc1 modifier 2](http://www.informatics.jax.org/javawi2/servlet/WIFetch?page=markerDetail&key=201960) | QTL |  |
| [Tmem100, transmembrane protein 100](http://www.informatics.jax.org/javawi2/servlet/WIFetch?page=markerDetail&key=51868) |  |  |
| [Tmem101, transmembrane protein 101](http://www.informatics.jax.org/javawi2/servlet/WIFetch?page=markerDetail&key=60527) |  |  |
| [Tmem102, transmembrane protein 102](http://www.informatics.jax.org/javawi2/servlet/WIFetch?page=markerDetail&key=58321) | targeted KO | Cell line only |
| [Tmem106a, transmembrane protein 106A](http://www.informatics.jax.org/javawi2/servlet/WIFetch?page=markerDetail&key=58786) |  |  |
| [Tmem132e, transmembrane protein 132E](http://www.informatics.jax.org/javawi2/servlet/WIFetch?page=markerDetail&key=97862) |  |  |
| [Tmem199, transmembrane protein 199](http://www.informatics.jax.org/javawi2/servlet/WIFetch?page=markerDetail&key=71553) |  |  |
| [Tmem49, transmembrane protein 49](http://www.informatics.jax.org/javawi2/servlet/WIFetch?page=markerDetail&key=59889) |  |  |
| [Tmem92, transmembrane protein 92](http://www.informatics.jax.org/javawi2/servlet/WIFetch?page=markerDetail&key=103408) |  |  |
| [Tmem93, transmembrane protein 93](http://www.informatics.jax.org/javawi2/servlet/WIFetch?page=markerDetail&key=50028) |  |  |
| [Tmem97, transmembrane protein 97](http://www.informatics.jax.org/javawi2/servlet/WIFetch?page=markerDetail&key=53051) |  |  |
| [Tmem98, transmembrane protein 98](http://www.informatics.jax.org/javawi2/servlet/WIFetch?page=markerDetail&key=60187) |  |  |
| [Tmevd5, Theiler's murine encephalomyelitis virus induced demyelinating disease susceptibility 5](http://www.informatics.jax.org/javawi2/servlet/WIFetch?page=markerDetail&key=65358) | QTL |  |
| [Tmigd1, transmembrane and immunoglobulin domain containing 1](http://www.informatics.jax.org/javawi2/servlet/WIFetch?page=markerDetail&key=50581) |  |  |
| [Tmub2, transmembrane and ubiquitin-like domain containing 2](http://www.informatics.jax.org/javawi2/servlet/WIFetch?page=markerDetail&key=56033) | Gene trap | Cell line only |
| [Tnfaip1, tumor necrosis factor, alpha-induced protein 1 (endothelial)](http://www.informatics.jax.org/javawi2/servlet/WIFetch?page=markerDetail&key=25297) | Gene trap | Cell line only |
| [Tnfsf12, tumor necrosis factor (ligand) superfamily, member 12](http://www.informatics.jax.org/javawi2/servlet/WIFetch?page=markerDetail&key=38372) | targeted KO | yes |
| [Tnfsf13, tumor necrosis factor (ligand) superfamily, member 13](http://www.informatics.jax.org/javawi2/servlet/WIFetch?page=markerDetail&key=53563) | targeted KO | yes |
| [Tnk1, tyrosine kinase, non-receptor, 1](http://www.informatics.jax.org/javawi2/servlet/WIFetch?page=markerDetail&key=64014) | targeted KO | yes |
| [Tns4, tensin 4](http://www.informatics.jax.org/javawi2/servlet/WIFetch?page=markerDetail&key=71817) |  |  |
| [Tob1, transducer of ErbB-2.1](http://www.informatics.jax.org/javawi2/servlet/WIFetch?page=markerDetail&key=43855) | targeted KO | yes |
| [Tom1l1, target of myb1-like 1 (chicken)](http://www.informatics.jax.org/javawi2/servlet/WIFetch?page=markerDetail&key=55923) |  |  |
| [Top2a, topoisomerase (DNA) II alpha](http://www.informatics.jax.org/javawi2/servlet/WIFetch?page=markerDetail&key=14201) |  |  |
| [Traf4, TNF receptor associated factor 4](http://www.informatics.jax.org/javawi2/servlet/WIFetch?page=markerDetail&key=35895) | targeted KO | yes |
| [Trim25, tripartite motif-containing 25](http://www.informatics.jax.org/javawi2/servlet/WIFetch?page=markerDetail&key=18703) | targeted KO | yes |
| [Trim37, tripartite motif-containing 37](http://www.informatics.jax.org/javawi2/servlet/WIFetch?page=markerDetail&key=77795) |  |  |
| [Trp53, transformation related protein 53](http://www.informatics.jax.org/javawi2/servlet/WIFetch?page=markerDetail&key=14279) | 44 alleles, targeted KO, targeted other, chemically induced | yes |
| [Trp53i13, transformation related protein 53 inducible protein 13](http://www.informatics.jax.org/javawi2/servlet/WIFetch?page=markerDetail&key=51855) |  |  |
| [Trpv1, transient receptor potential cation channel, subfamily V, member 1](http://www.informatics.jax.org/javawi2/servlet/WIFetch?page=markerDetail&key=41617) | targeted KO | yes |
| [Trpv3, transient receptor potential cation channel, subfamily V, member 3](http://www.informatics.jax.org/javawi2/servlet/WIFetch?page=markerDetail&key=80669) | targeted KO, spontaneous | yes |
| [Tshp10, tooth shape 10](http://www.informatics.jax.org/javawi2/servlet/WIFetch?page=markerDetail&key=81651) |  |  |
| [Tsr1, TSR1, 20S rRNA accumulation, homolog (yeast)](http://www.informatics.jax.org/javawi2/servlet/WIFetch?page=markerDetail&key=72006) |  |  |
| [Ttc25, tetratricopeptide repeat domain 25](http://www.informatics.jax.org/javawi2/servlet/WIFetch?page=markerDetail&key=58387) |  |  |
| [Ttll6, tubulin tyrosine ligase-like family, member 6](http://www.informatics.jax.org/javawi2/servlet/WIFetch?page=markerDetail&key=94288) |  |  |
| [Tubd1, tubulin, delta 1](http://www.informatics.jax.org/javawi2/servlet/WIFetch?page=markerDetail&key=48098) |  |  |
| [Tubg1, tubulin, gamma 1](http://www.informatics.jax.org/javawi2/servlet/WIFetch?page=markerDetail&key=17701) | targeted KO | yes |
| [Tubg2, tubulin, gamma 2](http://www.informatics.jax.org/javawi2/servlet/WIFetch?page=markerDetail&key=71648) | targeted KO | No phenotype upon observation |
| [Tusc5, tumor suppressor candidate 5](http://www.informatics.jax.org/javawi2/servlet/WIFetch?page=markerDetail&key=100883) |  |  |
| [Txndc17, thioredoxin domain containing 17](http://www.informatics.jax.org/javawi2/servlet/WIFetch?page=markerDetail&key=37516) |  |  |
| [Ube2g1, ubiquitin-conjugating enzyme E2G 1 (UBC7 homolog, C. elegans)](http://www.informatics.jax.org/javawi2/servlet/WIFetch?page=markerDetail&key=51108) |  |  |
| [Ube2z, ubiquitin-conjugating enzyme E2Z (putative)](http://www.informatics.jax.org/javawi2/servlet/WIFetch?page=markerDetail&key=41985) |  |  |
| [Ubtf, upstream binding transcription factor, RNA polymerase I](http://www.informatics.jax.org/javawi2/servlet/WIFetch?page=markerDetail&key=13797) |  |  |
| [Unc119, unc-119 homolog (C. elegans)](http://www.informatics.jax.org/javawi2/servlet/WIFetch?page=markerDetail&key=39019) | targeted KO | yes |
| [Unc45b, unc-45 homolog B (C. elegans)](http://www.informatics.jax.org/javawi2/servlet/WIFetch?page=markerDetail&key=84495) |  |  |
| [Usp32, ubiquitin specific peptidase 32](http://www.informatics.jax.org/javawi2/servlet/WIFetch?page=markerDetail&key=71915) |  |  |
| [Utp18, UTP18, small subunit (SSU) processome component, homolog (yeast)](http://www.informatics.jax.org/javawi2/servlet/WIFetch?page=markerDetail&key=60132) |  |  |
| [Utp6, UTP6, small subunit (SSU) processome component, homolog (yeast)](http://www.informatics.jax.org/javawi2/servlet/WIFetch?page=markerDetail&key=86013) |  |  |
| [Vat1, vesicle amine transport protein 1 homolog (T californica)](http://www.informatics.jax.org/javawi2/servlet/WIFetch?page=markerDetail&key=43801) |  |  |
| [Vezf1, vascular endothelial zinc finger 1](http://www.informatics.jax.org/javawi2/servlet/WIFetch?page=markerDetail&key=38233) | targeted KO | yes |
| [Vmbic6, ventral midbrain iron content 6](http://www.informatics.jax.org/javawi2/servlet/WIFetch?page=markerDetail&key=104624) | QTL |  |
| [Vmo1, vitelline membrane outer layer 1 homolog (chicken)](http://www.informatics.jax.org/javawi2/servlet/WIFetch?page=markerDetail&key=97959) |  |  |
| [Vps25, vacuolar protein sorting 25 (yeast)](http://www.informatics.jax.org/javawi2/servlet/WIFetch?page=markerDetail&key=27142) |  |  |
| [Vps53, vacuolar protein sorting 53 (yeast)](http://www.informatics.jax.org/javawi2/servlet/WIFetch?page=markerDetail&key=52279) |  |  |
| [Vtn, vitronectin](http://www.informatics.jax.org/javawi2/servlet/WIFetch?page=markerDetail&key=14450) | targeted KO | yes |
| [Wdr81, WD repeat domain 81](http://www.informatics.jax.org/javawi2/servlet/WIFetch?page=markerDetail&key=94229) |  |  |
| [Wfikkn2, WAP, follistatin/kazal, immunoglobulin, kunitz and netrin domain containing 2](http://www.informatics.jax.org/javawi2/servlet/WIFetch?page=markerDetail&key=91944) |  |  |
| [Wg7, weight gain in high growth mice 7](http://www.informatics.jax.org/javawi2/servlet/WIFetch?page=markerDetail&key=306621) | QTL |  |
| [Wipf2, WAS/WASL interacting protein family, member 2](http://www.informatics.jax.org/javawi2/servlet/WIFetch?page=markerDetail&key=61192) |  |  |
| [Wnk4, WNK lysine deficient protein kinase 4](http://www.informatics.jax.org/javawi2/servlet/WIFetch?page=markerDetail&key=53827) | targeted knockin | yes |
| [Wnt3, wingless-related MMTV integration site 3](http://www.informatics.jax.org/javawi2/servlet/WIFetch?page=markerDetail&key=14482) | targeted KO | yes |
| [Wnt9b, wingless-type MMTV integration site 9B](http://www.informatics.jax.org/javawi2/servlet/WIFetch?page=markerDetail&key=35426) | targeted KO | yes |
| [Wsb1, WD repeat and SOCS box-containing 1](http://www.informatics.jax.org/javawi2/servlet/WIFetch?page=markerDetail&key=62869) |  |  |
| [Wscd1, WSC domain containing 1](http://www.informatics.jax.org/javawi2/servlet/WIFetch?page=markerDetail&key=92772) |  |  |
| [Xaf1, XIAP associated factor 1](http://www.informatics.jax.org/javawi2/servlet/WIFetch?page=markerDetail&key=314830) |  |  |
| [Xylt2, xylosyltransferase II](http://www.informatics.jax.org/javawi2/servlet/WIFetch?page=markerDetail&key=85685) | targeted KO | yes |
| [Ybx2, Y box protein 2](http://www.informatics.jax.org/javawi2/servlet/WIFetch?page=markerDetail&key=33416) | targeted KO | yes |
| [Ypel2, yippee-like 2 (Drosophila)](http://www.informatics.jax.org/javawi2/servlet/WIFetch?page=markerDetail&key=61844) |  |  |
| [Ywhae, tyrosine 3-monooxygenase/tryptophan 5-monooxygenase activation protein, epsilon polypeptide](http://www.informatics.jax.org/javawi2/servlet/WIFetch?page=markerDetail&key=32959) | targeted KO | yes |
| [Zbtb4, zinc finger and BTB domain containing 4](http://www.informatics.jax.org/javawi2/servlet/WIFetch?page=markerDetail&key=59560) |  |  |
| [Zfp207, zinc finger protein 207](http://www.informatics.jax.org/javawi2/servlet/WIFetch?page=markerDetail&key=41201) |  |  |
| [Zfp3, zinc finger protein 3](http://www.informatics.jax.org/javawi2/servlet/WIFetch?page=markerDetail&key=14961) |  |  |
| [Zfp385c, zinc finger protein 385C](http://www.informatics.jax.org/javawi2/servlet/WIFetch?page=markerDetail&key=157976) |  |  |
| [Zfp652, zinc finger protein 652](http://www.informatics.jax.org/javawi2/servlet/WIFetch?page=markerDetail&key=83503) |  |  |
| [Zfp830, zinc finger protein 830](http://www.informatics.jax.org/javawi2/servlet/WIFetch?page=markerDetail&key=50963) | targetted KO | yes |
| [Zmynd15, zinc finger, MYND-type containing 15](http://www.informatics.jax.org/javawi2/servlet/WIFetch?page=markerDetail&key=157269) |  |  |
| [Znhit3, zinc finger, HIT type 3](http://www.informatics.jax.org/javawi2/servlet/WIFetch?page=markerDetail&key=117514) |  |  |
| [Zpbp2, zona pellucida binding protein 2](http://www.informatics.jax.org/javawi2/servlet/WIFetch?page=markerDetail&key=53356) |  |  |
| [Zzef1, zinc finger, ZZ-type with EF hand domain 1](http://www.informatics.jax.org/javawi2/servlet/WIFetch?page=markerDetail&key=85261) | targetted KO | Cell line only |
