# Supplementary material for: Discovery of Candidate Disease Genes in ENU–Induced Mouse Mutants by Large-Scale Sequencing, Including a Splice-Site Mutation in Nucleoredoxin
Source: PLoS Genet. 2009 Dec 11;5(12):e1000759. doi: 10.1371/journal.pgen.1000759 (PMC2782131; doi:10.1371/journal.pgen.1000759)
Supplement: Table S7 — Primers used to confirm Chromosome 11 ENU-induced mutations. (0.12 MB DOC) [file pgen.1000759.s009.doc]

Table S7: Primers used to amplify mutant DNA and confirm point mutations

| **Mutant Line** | **Gene name** | **Forward Primer** | **Reverse Primer** |
| --- | --- | --- | --- |
| *crf02* | *Hspb9* | CTCAGGCAGCAGTTGCACT | CTGAGCAGCCTCACTGGTAG |
| *crf06* | *Mnt* | TGCGAAGTTTGCACTGGG | GGAGGACCTGTGAGCTCTGA |
| *crf06* | *Plscr3* | TTCCCTGGACCCTTACTAGGT | ACTGGTGATGGCAGAGGGTC |
| *crf06* | *Ramp2* | TTGTACAATATTGCCTTCCTAGTGG | GAAAAAGTATAGGAACATGGCCTTC |
| *crf08* | *Olfr394* | AAGACCTTGTGGATGCCTTG | ACTTATCATGGGAGGGCTCA |
| *crf08* | *Med13* | ACTCACCTTGTGTTTCTTCCCAG | GTGGAGACTTGGGTTTTAGTTTCA |
| *crf12* | *Centb1* | CTTGTGCCTCGGGTCTTAG | TGGAGGAGTTGGTCAGGTGG |
| *crf12* | *Gip* | AGGCACAGGAGAGGTGAGTC | CAGCTCTGGTCTGAGGTTCC |
| *crf12* | *Mpdu1* | ACCCCAAGAGCTGCAGAGT | GCTGGAACGTAGGCAATCAG |
| *crf12* | *Med13* | ACATGTACTCTGCAACTACAGCTT | GCAGTAGGGTTGCTTTTAGAGT |
| *crf18* | *Klhl10* | AAGAGGAGGAGAATGTTTCCGT | GGTCCGAGTGTACGCATA |
| *crf26* | *Ccdc55* | TCCAGATATGGGCAGTCCTTAGT | AGGGCAGTGAGCAAGAGAGG |
| *crf26* | *Taok1* | TTGCTGCTATTTCCACTTCTTG | GTGTGGAAGCCAGAGGGTAA |
| *gro01* | *Dvl2* | CCACATGGACATGGCCTCTGT | GTGTTGTGCCCAGCTTTGG |
| *gro01* | *Nos2* | AGGGAATCTTGGAGCGAGTT | CACTACCAGTGAAGGCCAGA |
| *gro01* | *Traf4* | ACTGTTGGCAAAACCCAAAG | TTAGCCCTTGAGGTCCTTGA |
| *gro22* | *Abcc3* | GACAGAGGGAGGAGCATGAG | TGAAACGGATCCAGGATTTC |
| *gro22* | *Abr* | CCCCTCCCCCCTAAAATCTG | CCCTCTACAGCAGTACGTGT |
| *gro22* | *Mbtd1* | TTGAATGTTGGAATTGAAGAGCAGG | GCCGGCCTTGAGTTTGTA |
| *gro22* | *Mett10d* | GTTGCCATGCTGTCCACTC | CCCTAGGCACTACATTAAGAAAACA |
| *gro41* | *Stac2* | TGACGCCCTCATTCAGTACA | AGGCTCACTGAAACCCTCAC |
| *gro42* | *Nsf* | AACCATGTGTGACCACCTCA | GTTTTGTTCAGGCGTCGTTC |
| *gro42* | *Wdr79* | GTCACGCTTCTCCGAAGACT | CGTCGGTCCTAACATCATCA |
| *Inf3* | *Cpd* | AGCTAGTTTTGTGATCCCCTGAT | CAGTCAGGCCTAAAAGATTGGTAT |
| *Inf3* | *Sp6* | TCCCTCCATTGTTATAGAGTCCTCC | AAGTCTACCCTCTCATCCTATGATCAA |
| *Inf4* | *Erbb2* | GGCTACAGCCTGGTTCTTCA | GCAGCAAGGAGAAGGAAGTG |
| *Inf4* | *Tmem100* | AAAGAAGGGAGGGACATGCT | GCGTAGCTCCGTTCAACTTC |
| *Inf7* | *Plekhm1* | CTGCGGATCTTATGGCCTG | ACTCACCCCATTGTCGCT |
| *Inf7* | *RP23-263M10.5* | TGATTTTACCATGGAAAGGGAGT | CGAGCAGGTGAAGAGCTTG |
| *Inf7* | *RP23-350G1.1* | GCCAGCTTTTGCAATCATTA | GGTTTACAGCCACACACAGC |
| *l11Jus03* | *Git1* | TGCCCTTTTCTTTGATCCTG | ACATCCATGGCAAGTTCCTC |
| *l11Jus05* | *Med13* | TAATGGCGGATGGTGGGT | AAAGACAAGCCTCTTCCCAGC |
| *l11Jus06* | *Msi2h* | CCTTTTCCCATTCAAAGCAA | GGGCAGCTGTACCTGTCTGT |
| *l11Jus06* | *Tmigd1* | TCCTGCTCTCACACCTGACTT | TCCACAGGGGATTGCTTAAA |
| *l11Jus06* | *RP23-185A18.9* | GCCAGGTGCTTCAGAGCTAC | CCTCTAGAGAGCGGATGCTG |
| *l11Jus08* | *Zzef1* | CTGTTGCCTGTTGTATCTTC | CTGTTGCCTGTTGTATCTTC |
| *l11Jus12* | *Fbxl20* | GCGCTAAGAGGAGGACGTAG | CTCTCACGAACCAGCTCTCC |
| *l11Jus12* | *Map3k14* | GTTCTCCAGCTGACCATGCTT | TACCCACAGATCCCAGCCAC |
| *l11Jus12* | *P140* | ACGTCGTGTCTCCTCTCCAA | CTCTAGTTGCCTGGGGTCTG |
| *l11Jus12* | *Tlk2* | TTCCCTTCTTGTGTGTCTTTTC | GGCAGACTTCCTCCCTGATA |
| *l11Jus13* | *Mrpl27* | GCTTTTGAGGTAGGTTCCCAC | ACTCACGTGTTTACTGGCTG |
| *l11Jus13* | *Nbr1* | TGGCTTCCCATCCAGCTCT | GCCTAAAGGGTGATTATAAGTGTCC |
| *l11Jus13* | *Nxn* | CATTCTGACCTGCCTTCCACTT | TCAGGACAATGGCCAGCTAAG |
| *l11Jus13* | *RP23-396N4.2* | AAGACCTCAATATTCACTTAAGCCC | GACTGTCTCAAAAGCTCAAACAAAC |
| *l11Jus13* | *Mlx* | CTGCAGAGCAGCCAACAAG | GCTTTCACAAAACACTCAACCC |
| *l11Jus14* | *Stat3* | CAGCTCCTCAGTCACGATCA | AAGCTGGGTGGCTTATCCTT |
| *l11Jus15* | *Bzrap1* | GTGGATCACTGGGGATCATT | GGGGTAGGGGCTGTCTGTAT |
| *l11Jus15* | *Car10* | TTCTGCCACTGGTGAGAGG | CCCGACACGGTACACACATA |
| *l11Jus15* | *Med31* | ATGGCAGGCTTGAGATCATT | CCACTGTATGTCACCCCTATGTT |
| *l11Jus22* | *Mpp2* | TCTCCTGCAGTCTTCCGAAT | CCCCACTTCCAGGTTCAAG |
| *l11Jus22* | *Scpep1* | CACTTCAGCCCCCTACCTTT | CTCTGTTTATCTTCCAGTCTCTCCTT |
| *l11Jus22* | *Stat5a* | GTAGAATGTTCCAGCATGAGCTTC | CGCTGCAAAAGGAGCAAT |
| *l11Jus27* | *Dhx58* | ATGCCAACAGTCTGTAGGCAA | CCTGTTGCCTCTGTTTTGGTA |
| *l11Jus27* | *Usp32* | TCCTCTCGCTGTAACTTGGTA | TGCCAGCATAAACCACTCACA |
| *l11Jus38* | *Mapt* | TGGTTACCTGTCGTGGTTCTC | GTTGGAGAGGCATGGAAAGA |
| *l11Jus39* | *RP23-350G1.1* | GTTTGGATCTCCAGCAATGG | ACACAGGCAAGCACACATTC |
| *l11Jus45* | *Rnmtl1* | CATTCCTGTTTGCACCTCCT | CAATGTTTGCTCTGGCTGTC |
| *l11Jus45* | *Cntd1* | GAAGCCGTGGTGGACATTAT | CAGGGCGTTTTCGATAGTCT |
| *l11Jus45* | *Slc25a35* | CCTGATGCAGTGTGACATCC | GGTGAGAAAGCTAGCCAACG |
| *l11Jus45* | *Sez6* | AGGACTGCATCTGGGGTGT | TCCCCATCGTAGAAGGTCAG |
| *l11Jus45* | *Sp2* | TCACCATGAGCACATTTCCA | TTCCAGGTGAAGGAGCTCTG |
| *l11Jus48* | *Hes7* | GCTGGTCACAGATGCTGAAG | GTCCCCACCCCAGTAGAAAG |
| *l11Jus49* | *Acac* | GCTGTGACAGTTGGGCAGTA | GCAAGCCTGTCATCCTCAAT |
| *l11Jus51* | *Gps2* | CGTGACTCCGGAAGGATTTA | GCTCGCCACTTCGATCAC |
| *l11Jus52* | *Aipl1* | TCCCCCCATGCAGAATGGT | GGATTATCTAGGCTTGCACAGG |
| *l11Jus52* | *Atp6v0a1* | AACACCTAATGCCGAAAGGA | GCTTCGGTACAGCAGCATCT |
| *l11Jus52* | *Fzd2* | GAGCGCGTGGTATGCAAT | ACTTCATTCCGGCTGCCA |
| *l11Jus52* | *Plxdc1* | GGTAGAGTCCTCTGGCAGGA | TGAACCCCTCCCATCTCCTGT |
| *l11Jus52* | *Socs7* | ACGTCTCCTGTCCTGCAGTT | TGTGGTAGCCTCATTTGGTTC |
| *l11Jus54* | *Fzd2* | TCGGTGCTCTACACGGTA | TGTGAAGTCGGGCGACAT |
| *l11Jus55* | *Nf1* | GCCAGACGGTCATCTCTTGG | TTCATCCACACCCACGGTC |
| *l11Jus58* | *Arhgap23* | GGATCAGCAAAAAGCTCTGG | TCTTCATCCTCTCCCTGGAA |
| *l11Jus58* | *Cntnap1* | TGTGCTCATCAAGGAAGACG | CTATGGGGCTGACCATCAGT |
| *l11Jus58* | *Mrps23* | CAACCCTGTGGTAACCGACT | GCTCGAATCTGATCCTCCTG |
| *l11Jus58* | *RP23-96I9.2* | CGCATGCGTACATCTAGAGC | GCCCTCCTCACCATACTCC |
| *nur07* | *RP23-352L3.2* | AGTACTAGAAACCAAAACCATGCC | CCCGAGACATTGGAGCTATTT |
| *nur07* | *RP23-467J12.1* | ATTGCCATTTAAGGGAAGGGTT | TCAACAGTGACATGCCCCTGT |
| *nur08* | *RP23-185A18.9* | TGTGGGAGACGATTTTGTCA | CTGCTGACGCTGACCTGTAG |
| *nur09* | *RP23-136D4.2* | TGATCCAACAGTGCAGAGGA | GCTTCAGAAATCTCTGCGTTT |
