# Supplementary material for: Discovery of Candidate Disease Genes in ENU–Induced Mouse Mutants by Large-Scale Sequencing, Including a Splice-Site Mutation in Nucleoredoxin
Source: PLoS Genet. 2009 Dec 11;5(12):e1000759. doi: 10.1371/journal.pgen.1000759 (PMC2782131; doi:10.1371/journal.pgen.1000759)
Supplement: Table S8 — Microsatellite markers and SNPs used in mapping l11Jus13 (NxnJ13). PCR conditions are available upon request. F denotes forward primer, R denotes reverse primer. (0.06 MB DOC) [file pgen.1000759.s010.doc]

**Table S8:** Microsatellite markers and SNPs used in mapping *l11Jus13* (*Nxn^J13^*).

| **Marker** | **Assay** | **Forward and Reverse Primer Sequences** | **Restriction enzyme** |
| --- | --- | --- | --- |
| *D11Mit4* | PCR | F- 5’-CAGTGGGTCATCAGTACAGCA-3’  R- 5’-AAGCCAGCCCAGTCTTCATA-3’ | N/A |
| *DllMit219* | PCR | F-5’-TTGTATGTATAGATGCATTTGAATGG-3’  R-5’-GGTTTGTATAAATTCTCACCTGTG-3’ | N/A |
| *D11Bhm148* | PCR | F-5’-AGGGGAAGTCCTGTATGGACA-3’  R-5’-ACCAACCTCGATAGAGCCATC-3’ | N/A |
| *D11Mit245* | PCR | F-5’ATGAGACCATGCTCCTCCAC-3’  R-5’-TTGTCCTCTGACCTTCACACC-3’ | N/A |
| *D11Mit120* | PCR | F-5’-CTTCTGATTTCCTCTTGCACG-3’  R-5’-TGGCATAAGAGACAGGCTCA-3’ | N/A |
| *D11Mit324* | PCR | F-5’-CTGGTCTACGTTGAGTGCCA-3’  R-5’-AGAGAGAACAGCAAACATTCAGG-3’ | N/A |
| *D11Mit39* | PCR | F-5’-TTTCATGACCCCTAATTTCCC-3’  R-5’-GTGGGTGTGCCTGTCAATC-3’ | N/A |
| *D11Mit327* | PCR | F-5’-ATTACAGTTGACTGATACCAATCAGC-3’  R-5’-TCAGGCTCCACTGTGAAATG-3’ | N/A |
| *D11Mit132* | PCR | F-5’-GGTCAGAGGACAATCTTACATGC-3’  R-5’-GTTCCAAGACAATGAGAGACCC-3’ | N/A |
| *D11Mit333* | PCR | F-5’-CATGTGGTTATTTTCTAGCCCC-3’  R-5’-AGGCATCAATAACTATTTTTCAGTG-3’ | N/A |
| *rs3702197* | PCR + restriction digest | F-5’-CTCTCCTGGTGCTTTGGAAG-3’  R-5’-GGTGCAGCTCGACACAACTA-3’ | *Sau*96I |
| *rs13481111* | PCR + restriction digest | F-5’-GTAAGGACAAAGAGGACTGCCAAG-3’  R-5’-AATGACAGACAGGAGGAAATCCAT-3’ | *Sau*96I |
| *rs13481113* | PCR + restriction digest | F-5’-AAGGAGATTGGAGTTGCCTATTTG-3’  R-5’-TGTGTACTCCTGAGCCTTCTGTCT-3’ | *A*ciI |
| *rs13481117* | PCR + restriction digest | F-5’-CTCAAATCTGCTGAATGGTAGTGG-3’  R-5’-TGAGGTCATGAGGTCAAGATTGTT-3’ | *Aci*I |
| *rs13481125* | PCR + restriction digest | F-5’-TCCTCTTAAGGGCTATTAAGTTCCAA-3’  R-5’-ACTTCACCTGCCATCCCTGT-3’ | *Hae*III |
